# Supplementary material for: Cell-type-specific damage scores reveal kidney and liver disease trajectories in single-cell and spatial transcriptomics
Source: Cell Genom. 2026 Aug 20;6(10):101337. doi: 10.1016/j.xgen.2026.101337 (PMC13689510; doi:10.1016/j.xgen.2026.101337)
Supplement: Supplementary file 1 [file mmc1.pdf]

Cell Genomics, Volume *6*

Supplemental information

Cell-type-specific damage scores reveal kidney and liver disease trajectories in single-cell and spatial transcriptomics

Tsimafei Padvitski, Paula Unger Avila, He Chen, Cem Özel, Fabian Braun, Marie-Kristin Kroll, Christian Vossen, Peter Wienand, Linus Butt, David Unnersjö-Jess, Anna-Lena Schumacher, Kat Folz-Donahue, Heike Göbel, Christoph Kuppe, Roman-Ulrich Müller, Pål Olof Westermark, Paul Thomas Brinkkötter, Bernhard Schermer, Thomas Benzing, Frank Thomas Wunderlich, Martin Kann, and Andreas Beyer

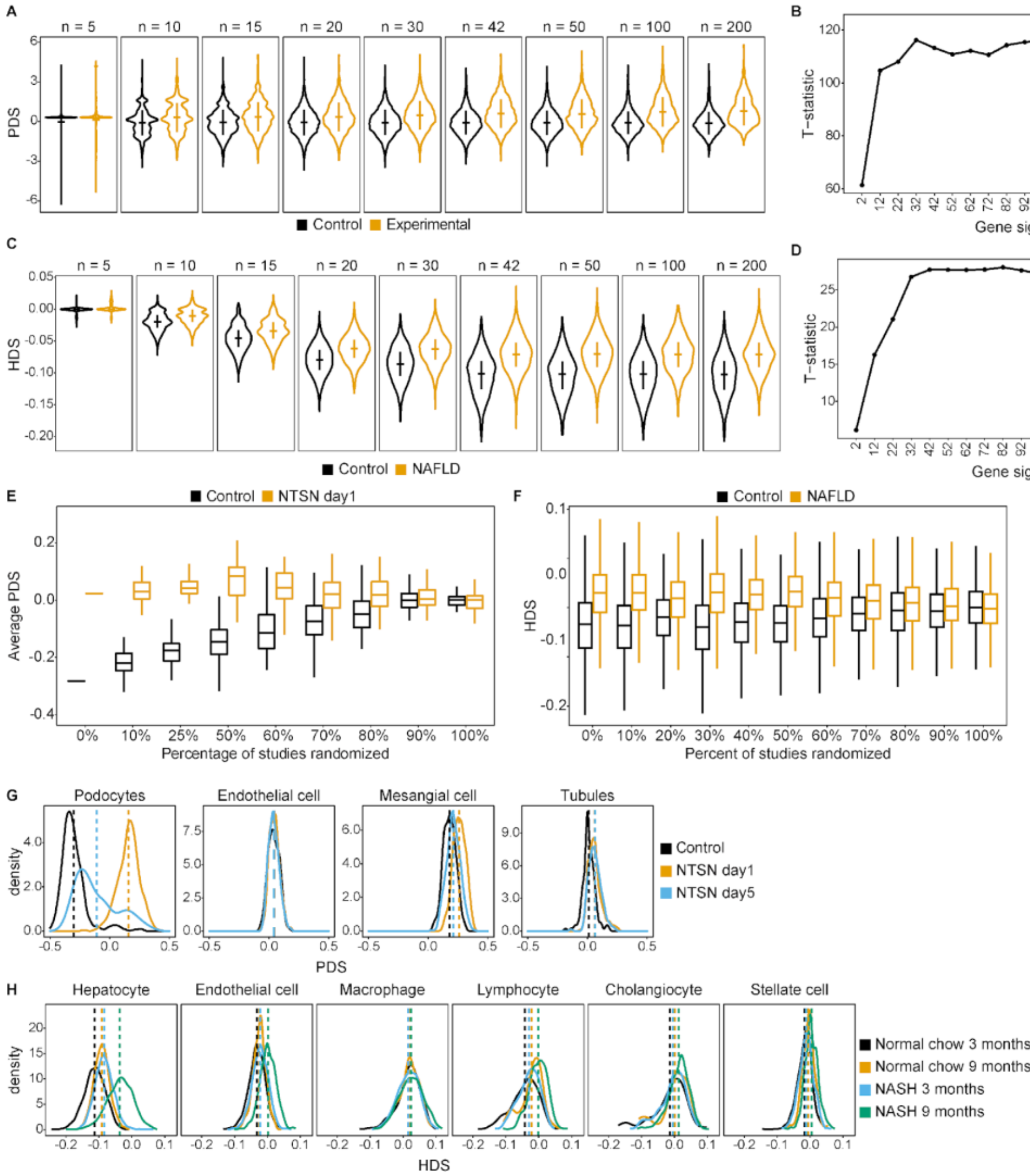

Figure S1: Robustness and specificity of PDS and HDS, related to Figure 1

(A) Signature size test for PDS. Three snRNA-seq ($Wt1^{ko/wt}$, $Nphs2^{mut}$, $Pdss2^{mut}$) and four scRNA-seq datasets from Chung et al.[1] were used to test for the number of genes in the PDS needed to robustly differentiate control (black) and experimental (orange) conditions. PDS were scaled per study (Y-axis). Number of top genes (*n*) used per signature is indicated above. Insets in violin plots show the mean plus/minus 2 standard deviations. (B) Signature size test shows absolute t-statistics of PDS comparisons between control and experimental samples in sc/snRNA-seq studies as a function of damage signature size (top *N* genes used; X-axis). Larger absolute t-statistics indicates stronger separation of experimental and control samples. (C) Same as in A for HDS, except HDS is not scaled. Median HDS per distribution are indicated; whiskers represent the inter quartile range (IQR). HDS calculated for snRNA-seq of hepatocytes from 24-week-old mice with and without NAFLD-inducing diet, *n* = 5 (data from Liver Cell Atlas, GSE192742[2,3]). (D) Same as in B for HDS. Signature size test shows absolute t-statistics of HDS comparisons between control and experimental samples in snRNA-seq data as a function of damage signature size (top *N* genes used; X-axis; same data as in C). Larger absolute t-statistics indicates stronger separation of experimental and control samples. (E) PDS robustness test against noise in training data. Varying fractions of the training data were randomized to test how this would reduce the ability of the PDS to successfully distinguish experimental from control conditions in independent test data (see Methods). ScRNA-seq data from nephrotoxic serum nephritis (NTSN) model of podocyte damage from Chung et al.[1] was used for the test. 50 rounds of randomization were conducted for each ‘percentage of studies randomized’. Box plots show first (Q1), second (Q2), and third quartiles (Q3) of the distribution of average PDS per cell across 50 randomization rounds per ‘percentage randomized’. Whiskers represent Q1 - 1.5 x interquartile range (IQR) and Q3 + 1.5 x IQR. (F) Same as in E, but for the HDS. Test was conducted on hepatocyte snRNA-seq (same data as in C). HDS centered per run. Median HDS per distribution is indicated; whiskers represent IQR. (G) Cell-type specificity test for the PDS. Panels show distribution of PDS in four major glomerular cell types (podocytes, endothelium, mesangium and tubules, color of lines reflect experimental condition (control and two damage time points). scRNA-seq data from the nephrotoxic nephritis model[1]. Vertical dashed lines represent the mean. (H) Cell-type specificity test for the HDS. Panels show distribution of HDS in hepatocytes and five major liver cell types from Xiao et al.[4] Cell type annotation was adopted from the authors. Colour of density lines represent the condition of the mouse (*n* = 1). Vertical dashed lines represent the mean.

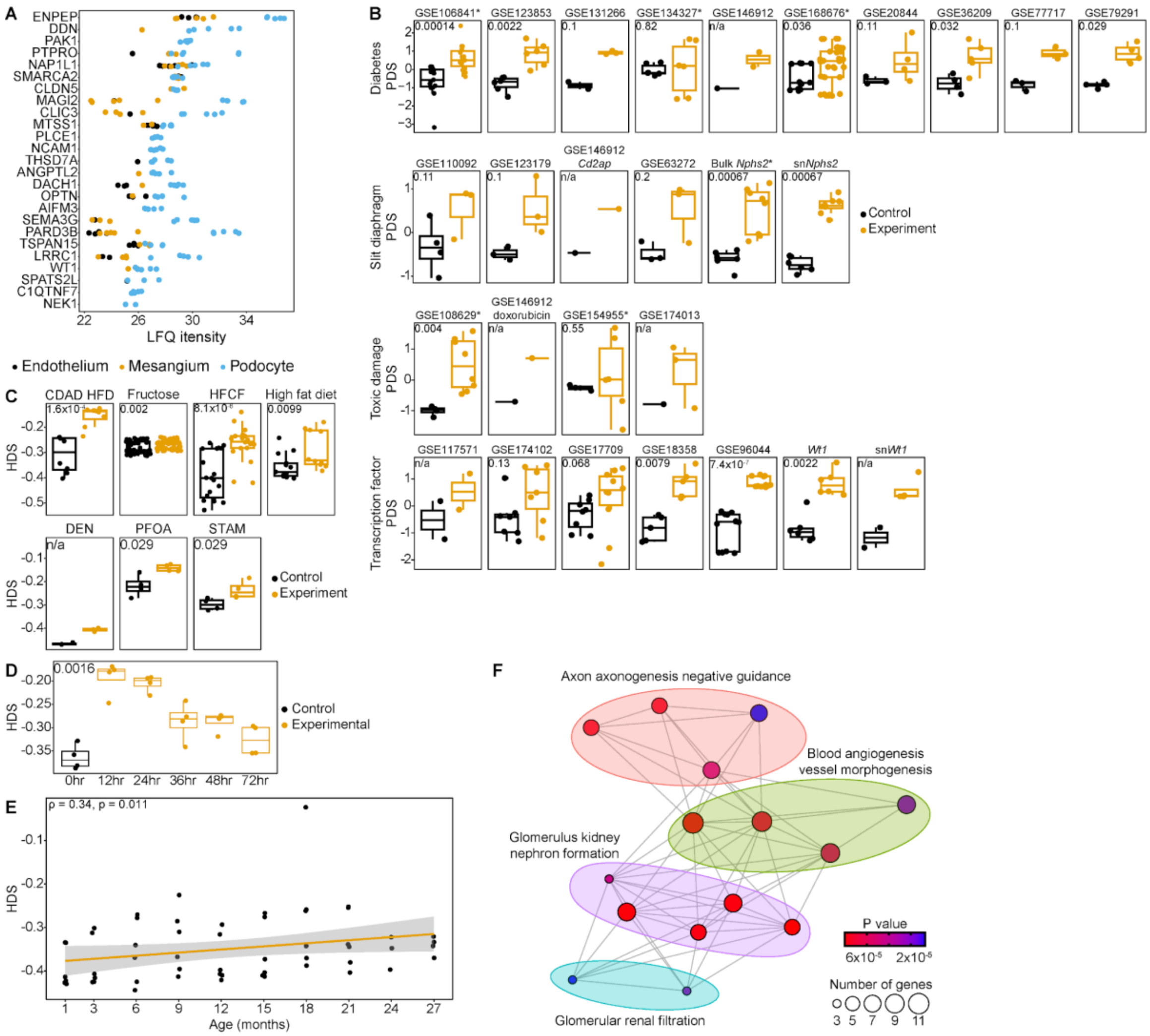

Figure S2: Cross validation of PDS and HDS, related to Figure 1.

(A) Protein expression of PDS marker genes in three types of mouse glomerular cells: podocytes (blue), endothelial cells (black) and mesangial cells (orange). Data is from glomerular proteomics as reported in PXD016238[5]. Non-scaled protein expression values (LFQ) are shown. (B) Leave-one-dataset-out cross validation of the PDS. Each dataset of the indicated group of disease models were left out during the training phase of the PDS and used for testing the indicated disease model afterwards. Each dot represents the mean PDS across all cells of a sample. PDS were scaled and centered per study. Boxplots show first (Q1), second (Q2), and third quartiles (Q3) of the distribution. Whiskers represent Q1 - 1.5 x interquartile range (IQR) and Q3 + 1.5 x IQR. Outlier cells are shown as dots. *P*-value of Wilcoxon test is shown for all studies with at least 3 samples in both conditions. For studies GSE106841[6] (ageing series in diabetes), GSE134327[8], GSE168676[10], Bulk *Nphs2*[14], GSE108629[12], and GSE154955[15] diverse experimental animals (different age, treatments etc.) were summarized as 'experiment'. (C) Leave-one-model-out cross validation of the HDS. Procedure is the same as in B. Each dot represents the average HDS of one sample. CDAD HFD: choline-deficient, L-amino acid-defined, high-fat diet (n = 8), Fructorse (n = 54), HFCF: high fat, cholesterol, and fructose diet (n: control = 19, treatment = 20), High Fat Diet (n: control = 13, treatment = 10), DEN: Diethylnitrosamine (DEN)-induced carcinogenic liver injury (n = 3), PFOA: perfluorooctanoic acid (n = 4), STAM: mouse model that recapitulates human NASH progression to HCC and uses a combination of streptozotocin and high fat diet (n = 4). Boxplots show the first (Q1), second (Q2), and third (Q3) quartiles of distribution. Whiskers show Q1 - 1.5 x IQR and Q3 + 1.5 x IQR. P-values of Wilcoxon rank test (experimental versus control conditions) are shown. (D) Cross validation of the HDS on a time course dataset. Procedure is the same as in B Each dot represents a sample in time series murine liver samples post hepatic injury induced by acetaminophen administration, n = 4 (GSE111828[7]). Labels on x-axis represent the time in hours (hr) after acetaminophen administration. Untreated control samples are labeled with 0hr. *P*-value of Kruskal Wallis test shown. (E) Cross validation of HDS on aging time course in murine liver. Each dot represents the HDS of a murine liver sample of an age group (in months on x-axis) from the Tabula Muris Senis data set, n = 6, except for 24 month (n = 3), and 27 months (n = 4) (GSE132040[9,11]). Regression line (orange with confidence interval as gray shading) shown as visual aid to highlight age-related trend. Correlation coefficient and *p*-value of Spearman correlation are indicated. (F) Functional annotation of the podocyte damage marker set confirms its relations to the key podocyte functions. GO terms were grouped by semantic similarity. Nodes are colored by *p*-value of Fisher's exact test and sized by the number of genes tested.

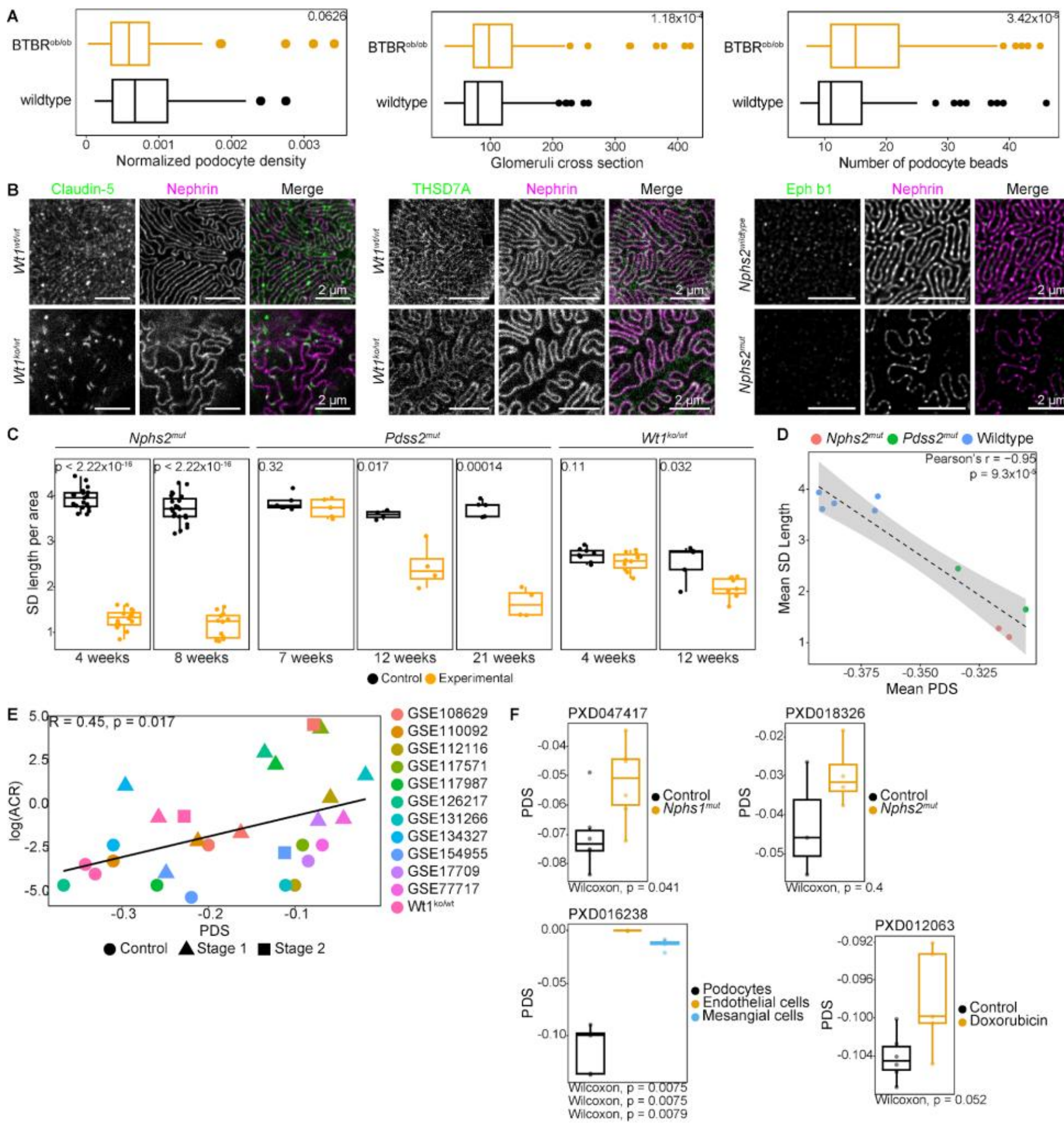

Figure S3: Functional validation of PDS, related to Figure 1.

(A) Boxplots showing glomerular size estimates, podocyte bead number, and normalized podocyte density for diabetic BTBR$^{ob/ob}$ (orange) and control mice (black) as calculated on Slide-seqV2 spatial transcriptomics data provided in GSE190094[13]. Podocyte KNN filtration, glomerular identification and size estimation was performed using the programming code of the original publication[13]. Boxplots show quartiles (Q), with whiskers representing Q1 - 1.5 x interquartile range (IQR) and Q3 + 1.5 x IQR. Outlier cells are represented by dots. Values in top right are *p*-values of Wilcoxon rank sum test of the difference between experimental and control groups. (B) STED of 12-week-old *Wt1*$^{ko/wt}$ or control mice co-stained for nephrin to visualize the slit diaphragm and PDS marker proteins including Claudin-5 and THSD7A. STED of 4-week-old *Nphs2*^{mut} or control mice was co-stained for nephrin and the Eph B1 receptor. All PDS marker proteins localize to the slit diaphragms. In *Wt1*$^{ko/wt}$ mice, Claudin-5 forms aggregates at the slit diaphragms. In *Nphs2*^{mut} mice, the expression of Eph B1 is downregulated upon podocyte damage. (C) Quantification of SD length per area for *Nphs2*^{mut} (n = 19 for 4-week control, n = 16 for 4-week experimental, n = 23 for 8-week control, n = 12 for 8-week experimental), *Pdss2*^{mut} (n = 10 for 7-week, n = 8 for 12-week, n = 9 for 21-week) and *Wt1*$^{ko/wt}$ (n = 9 for 4-week control, n = 10 for 4-week experimental, n = 5 for 12-week control, n = 7 for 12-week experimental) control and experimental animals at the indicated age. Boxplots show quartiles (Q), with whiskers representing Q1 - 1.5 x interquartile range (IQR) and Q3 + 1.5 x IQR. *P*-values of t-test are shown between control and experimental groups. (D) Correlation plot of average PDS and average SD length per area for wildtype and experimental *Nphs2*^{mut} (Grouped control: n = 2 for 4-week, n = 1 for 6-week, n = 1 for 8-week, n = 2 for 12-week; Grouped experimental: n = 2 for each of 4-week, 6-week, 8-week and 12-week) and *Pdss2*^{mut} (Grouped control: n = 2 for each of 6-week, 12-week and 21-week; Groupe experimental: n = 2 for 6-week, n = 2 for 12-week and n = 1 for 21-week) mouse groups. Linear regression of PDS correlated with SD length shown in dashed line with 95% confidence interval colored in grey. Coefficient and *p*-value for Pearson's correlation are shown. (E) Correlation of urinary albumin/creatinine ratio (ACR) and PDS in bulk microarray and bulk RNA-seq data. *Wt1*$^{ko/wt}$ represents bulk RNA-seq data published[14]. Stage 1 and 2 indicate datasets, in which longitudinal ACR data was available. Coefficient and *p*-value for Pearson's correlation are shown. (F) Boxplots show PDS calculated for various conditions and genotypes in 4 proteomics datasets. Individual samples are shown as dots. *P*-value of Willcoxon test of condition/genotype effect are provided below each panel. ProteomeXchange identifiers (PXDs) of the corresponding datasets are indicated[5,16–18]. Boxplots show quartiles (Q), with whiskers representing Q1 - 1.5 x interquartile range (IQR) and Q3 + 1.5 x IQR. Outlier cells are represented by dots. *P*-values of Wilcoxon rank sum test of the difference between experimental and control groups are shown.

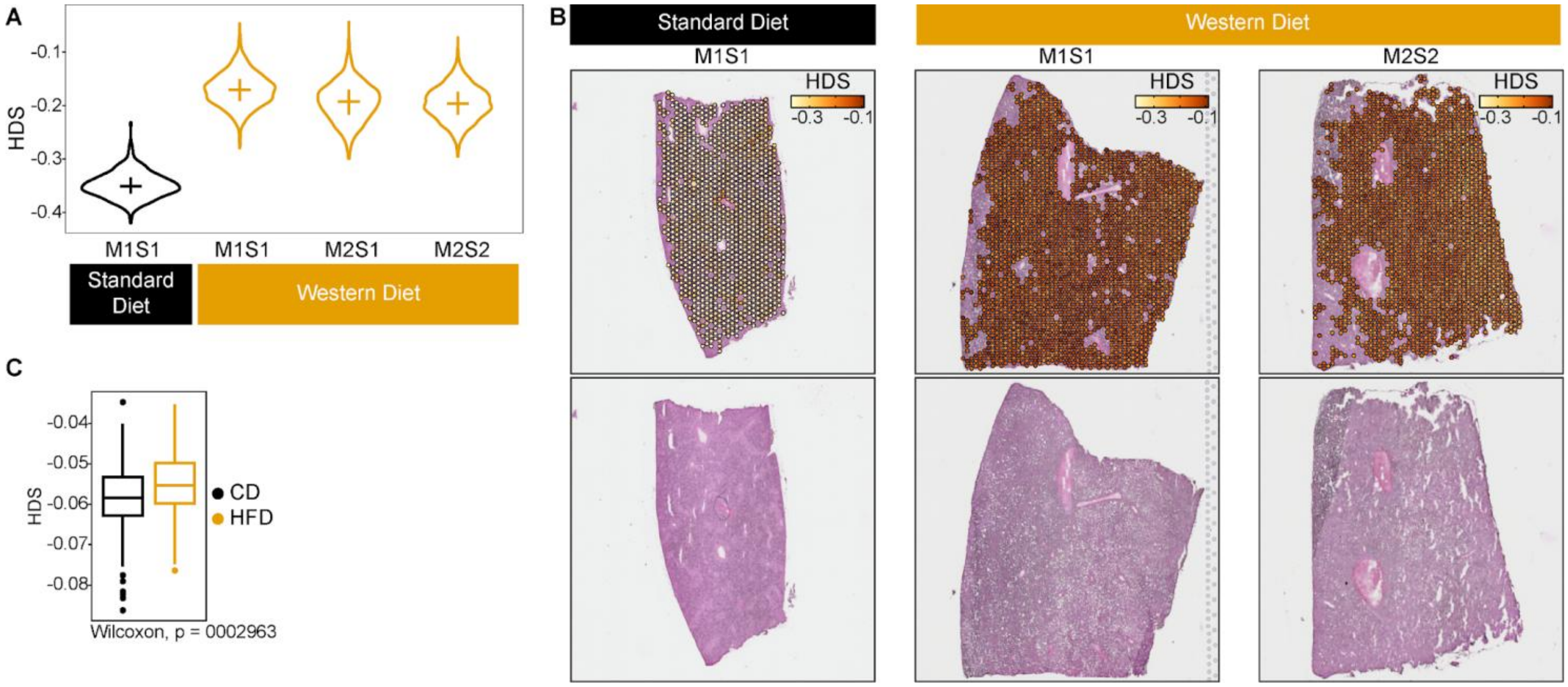

Figure S4: Functional validation of the HDS, related to Figure 1.

(A) Violin plots of HDS distribution in all murine spatial transcriptomic 10X Visium samples from the Liver Cell Atlas (GSE192742[2,3]) comparing western (n = 3) to standard diet (n = 1). M1S1 = Mouse 1 Sample 1, M2S1 = Mouse 2 Sample 1, M2S2 = Mouse 2 Sample 2. Horizontal lines show the median and vertical lines the IQR of the distributions. Statistical comparison not appropriate due to n = 1 per "Standard Diet" group. (B) 10X Visium spatial transcriptomics of murine liver from the Liver Cell Atlas (GSE192742[2,3]). Spatial plots of HDS distribution in spatial transcriptomic not previously shown in Figure 1. Color range: floor = -0.37, ceiling = -0.09. *P*-value for Wilcoxon test shown. (C) Confirmation of HDS on murine liver proteomics data[19]. Mice fed a high fat diet (HFD) compared to mice fed a control diet (CD), (n = 315 across conditions, different strains, age range between 157 and 785 days). *P*-value for one-sided Wilcoxon test is shown.

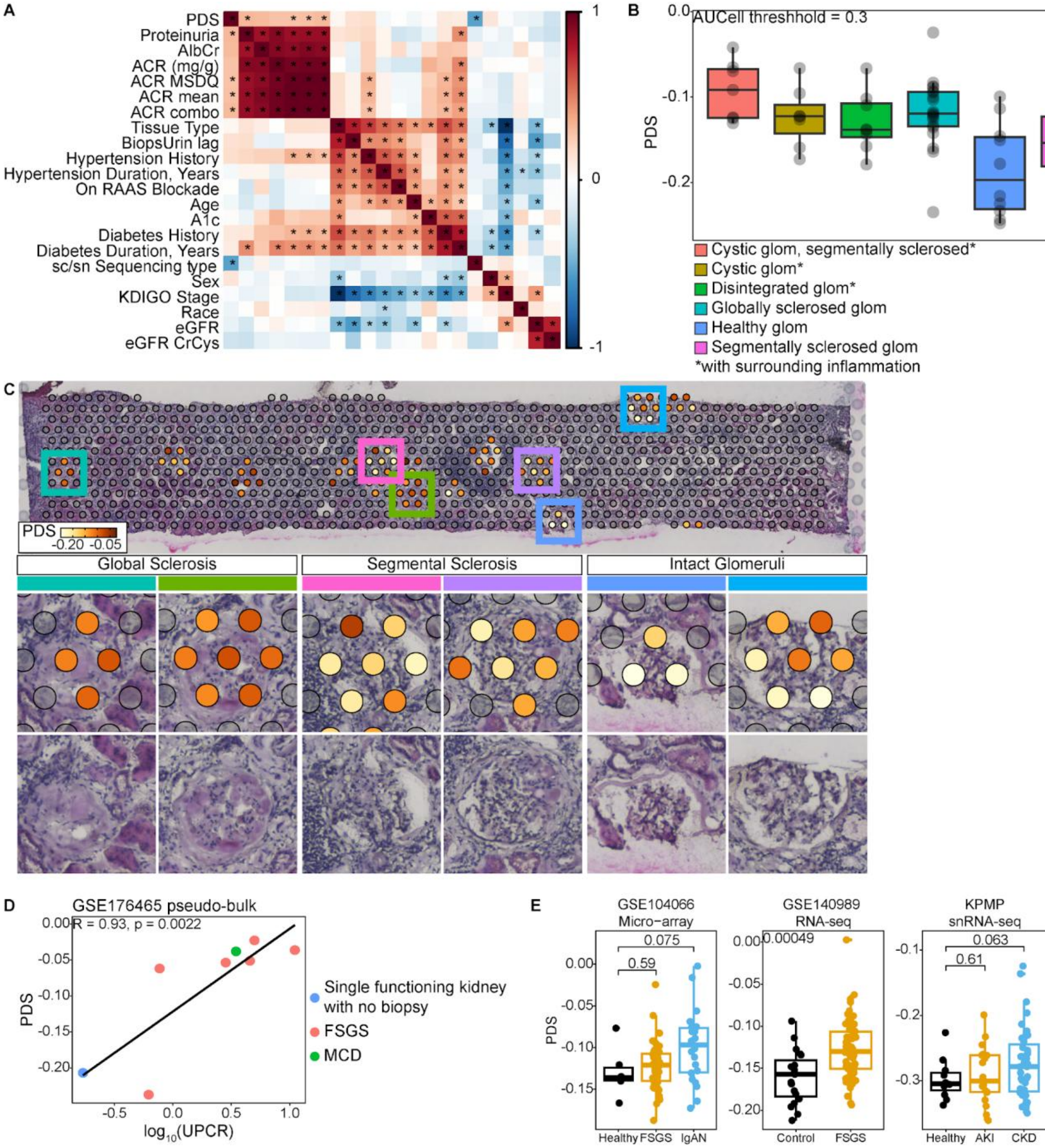

Figure S5: Validation of the PDS on human data, related to Figure 2.

(A) Heatmap of spearman correlation between PDS and detailed clinical traits from patients with acute kidney injury (AKI) and CKD. Anonymized patient data, sc/snRNA-seq data used in the analysis were retrieved from the KPMP database. Stars denote cells with *p*-value of Spearman correlation < 0.05. (B) Boxplot shows PDS in further glomeruli as annotated manually in the histology image shown in the next panel, PDS measurements based on KPMP spatial transcriptomics dataset,[20] patient ID 29-10282, accessed at https://atlas.kpmp.org/. (C) 10X Visium spatial transcriptomic data on a human kidney biopsy from the KPMP registry (patient ID 29-10282)[20] showing an FSGS phenotype. PDS values were computed only for Visium spots with podocyte signals as determined by *WT1* and *NPHS2* expression. Selected glomeruli are marked in the upper panel with respective colors. High magnification images show various degrees of glomerular sclerosis with PDS gradients corresponding to degree of sclerosis. See boxplots for PDS distribution as by glomerular damage in the previous panel. (D) Relationship between PDS calculated on human urinary podocyte snRNA-seq data and urinary protein creatinine ratio (UPCR) in patients suffering from either minimal change disease (MCD) or focal and segmental glomerulosclerosis (FSGS) as reported in GSE176465[21]. Data from 8 patients with at least three measured podocytes each was used. (E) Validation of PDS on human transcriptomic data obtained from healthy patients (CON) and from patients suffering from FSGS, IgA nephropathy (IgAN), acute kidney injury (AKI), and chronic kidney disease (CKD). Samples were acquired and reported in transcriptomic modalities and studies as indicated on top of the plots. Podocytes from snRNA-seq datasets were aggregated in pseudo-bulk samples. *P*-values of Wilcoxon rank sum test comparing experimental and control samples are shown.

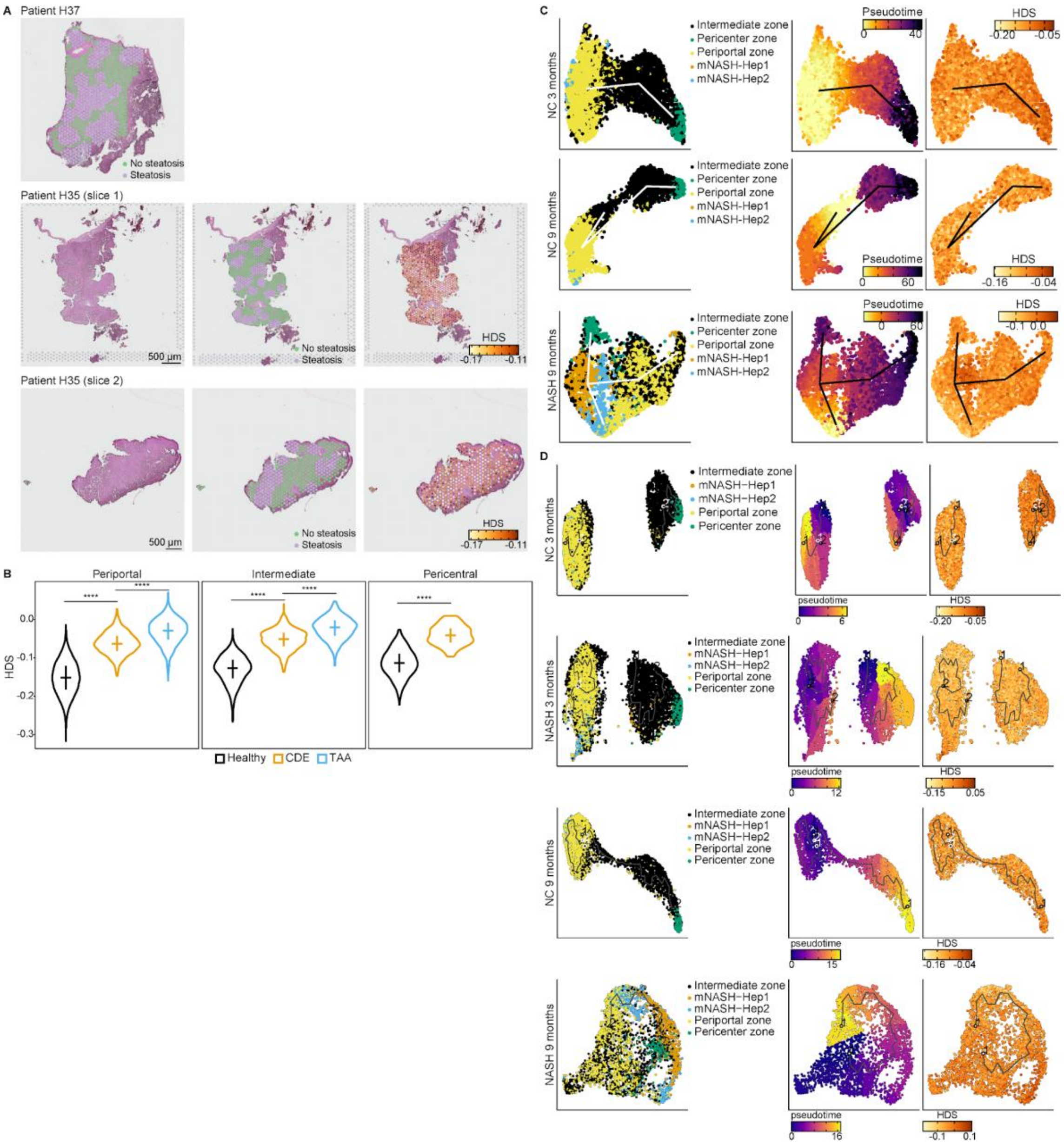

Figure S6: Validation of HDS using single nucleus and spatial transcriptomics, related to Figure 3.
(A) HDS calculation on 10X Visium human spatial transcriptomic data from the Liver Cell Atlas (GSE192742[2,3]). Only subjects with liver steatosis were analyzed. Top panel: manual assignment of steatosis vs non-steatosis areas as used for Figure 3D. Bottom two rows: Left panel: histology, Middle panel: assignment of steatosis vs n-steatosis areas, right panel: HDS per Visium spot overlay. Spots representing hepatocytes were manually annotated. Color scale represents the 10%- and 90%-quantiles of the HDS distribution across all samples. Spots in regions with steatosis: light purple, spots in no-steatosis regions: green. (B) HDS distributions by liver zonation in snRNA-seq data from mice subjected to three different treatments. Healthy: normal chow; CDE: choline-deficient, ethionine-supplemented (CDE) diet; TAA: thioacetamide (TAA) in the drinking water (GSE200366[22]) (n = 3 biological replicates per treatment). Same data is in Figure 6A. Hepatocyte zone annotation as provided by authors of data set. Pericentral hepatocytes were not detected in TAA-treated animals. Horizontal lines show median of the distribution and vertical lines represent the IQR. **** = $p < 2 \times 10^{-16}$ in one sided Wilcoxon rank-sum test. (C) Analysis of HDS versus pseudo-time trajectories with TSCAN. UMAPs for further samples from the same study as shown in Figure 3G. Each dot represents a hepatocyte colored either by liver zone (left), pseudo-time (PT) (middle) and HDS (right). Hepatocyte zone annotation from Xiao et al.[4] MNASH-Hep1/2: classification from original publication corresponding to damaged hepatocytes whose zone of origin could not be determined according to the authors. PT trajectory inference is based on clusters identified using Seurat after quality control cell filtering, normalization with Seurat's SCTransform and dimensionality reduction. PT is dominated by liver zonation, while the HDS is independent of it. Top: Normal Chow 3-month-old, middle: NASH-inducing diet 9-month-old (n = 1), bottom: Normal Chow 9-month-old mouse. (D) Analysis of HDS versus pseudo-time trajectories with Monocle3. Same data and legends as in C. Trajectory inference is based on clusters identified using Monocle3 default processing and clustering pipeline. PT trajectories and clustering are dominated by liver zonation also in Monocle3 analysis. HDS is not reflected by PT trajectories.

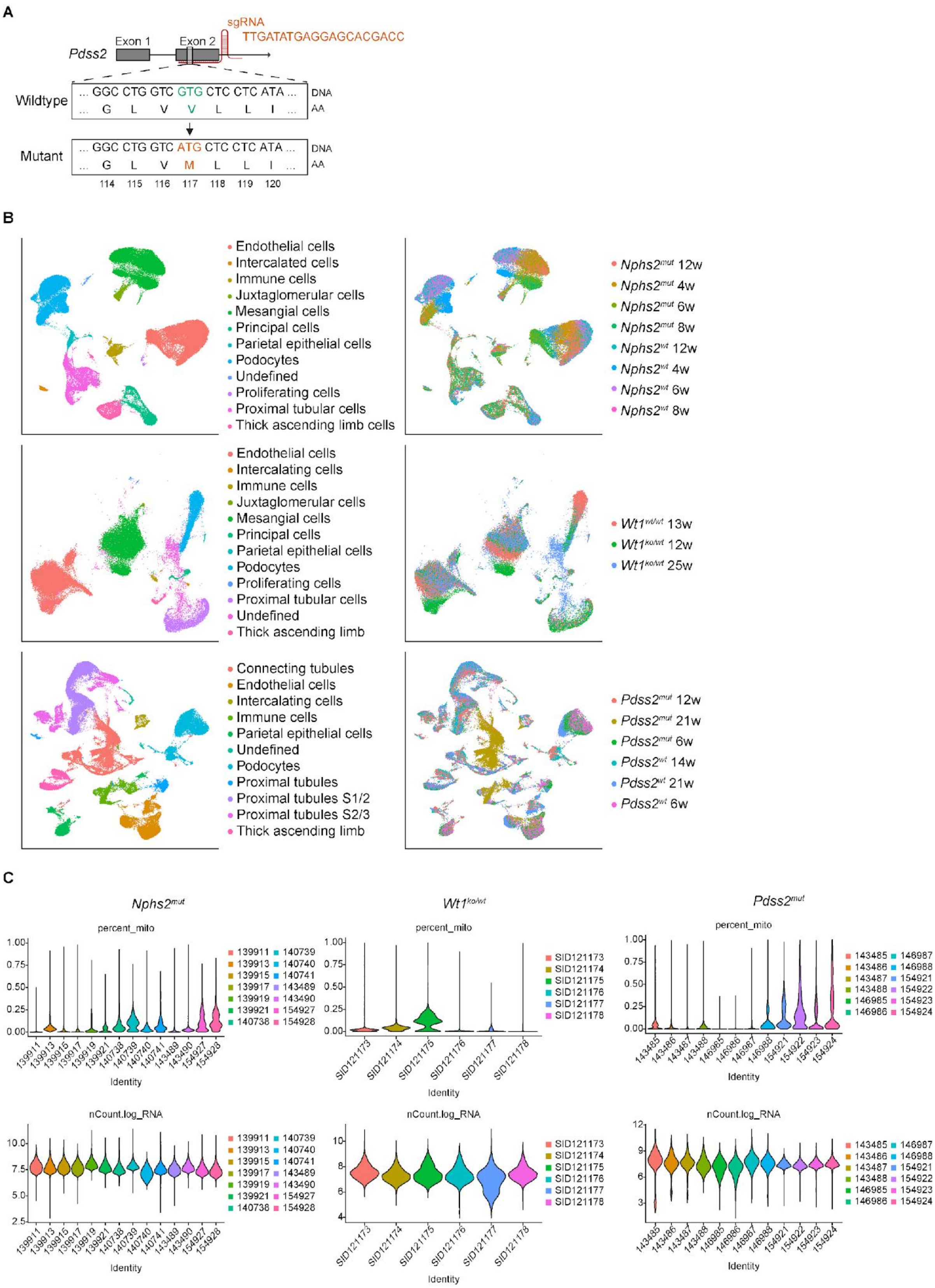

Figure S7: Glomerular snRNA-seq of *Nphs2^{mut}*, *Wt1^{ko/wt}* and *Pdss2^{mut}* mice, related to Figure 4.
(A) CRISPR strategy for the generation of the *Pdss2^{mut}* mice. Exon 2 of *Pdss2* is targeted using single guide RNA (sgRNA) to generate a point mutation which exchanges valine (V) at amino acid (AA) position 117 to methionine (M). (B-C) Overview of snRNA-seq data generated for this study. (B) UMAP of preprocessed snRNA-seq data colored by cell types (left) or condition (right) of *Nphs2^{mut}* (top), *Wt1$^{ko/wt}$* (middle) or *Pdss2^{mut}* (bottom) datasets. UMI counts corrected for ambient RNA, filtered and normalized (see Methods), are used for this and the next panels. (C) Percent mitochondria (top) and log

transformed number of RNA counts (bottom) for individual snRNA-seq samples of *Nphs2*^{mut} (left), *Wt1*$^{ko/wt}$ (middle) or *Pdss2*^{mut} (right) datasets.

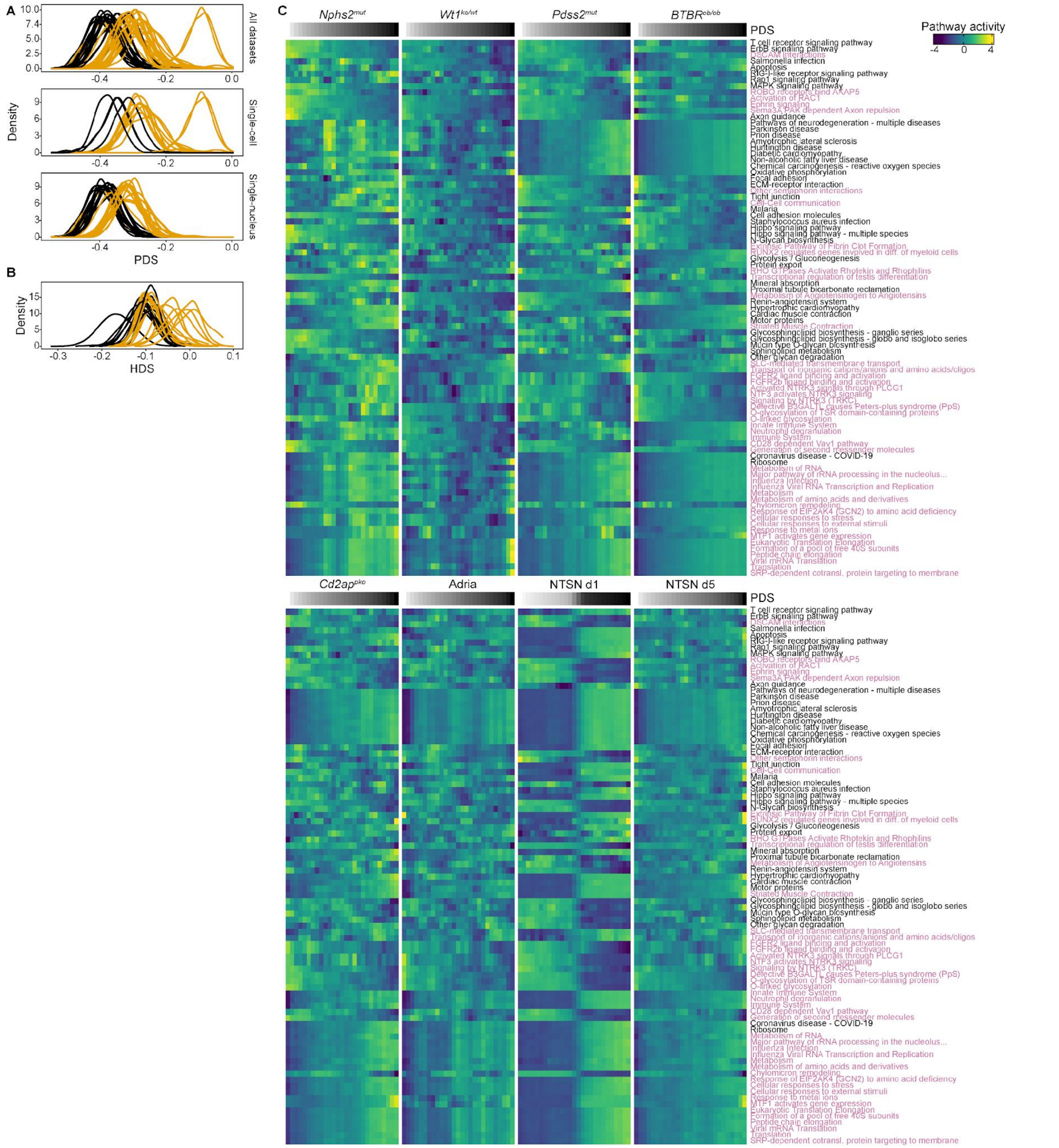

Figure S8: Pathway fingerprints of all FSGS sc/sn-RNA-seq models, related to Figure 4.

(A) Density plots show greater variation of PDS in experimental compared to control samples. PDS densities of individual samples are aligned by x-axis. Black: controls, orange: experimental conditions. Top panel shows results for all sc/snRNA-seq samples, bottom two panels show results for single-cell and single-nuclei datasets separately. Wilcoxon Rank Sum test confirms (p-value = 2.5×10^{-11}) that controls are significantly better aligned than experimental samples (see method section Damage score alignment test). (B) Density plots show greater variation of HDS in experimental compared to control samples. HDS densities from three sn/scRNA-seq studies across different liver damage models (NAFLD, NASH, ageing, GSE192742[2,3], GSE200366[22], GSE189600[4]). Black: controls, orange: experimental samples. Wilcoxon rank sum test confirms that control samples are aligned significantly better than experimental samples (p-value = 2.7×10^{-4}). (C) Full PDS pathway fingerprints for sn/scRNA-seq data from all studies analyzed. Cells/nuclei were sorted by increasing PDS (left to right, gray color scale). Pathway activities for KEGG (black) and Reactome (magenta) pathways were calculated using AUCell (see Methods). A total of 85 pathways - a union of 10 pathways that most significantly (adjusted p-value < 0.05) correlate with PDS in each model - is used.

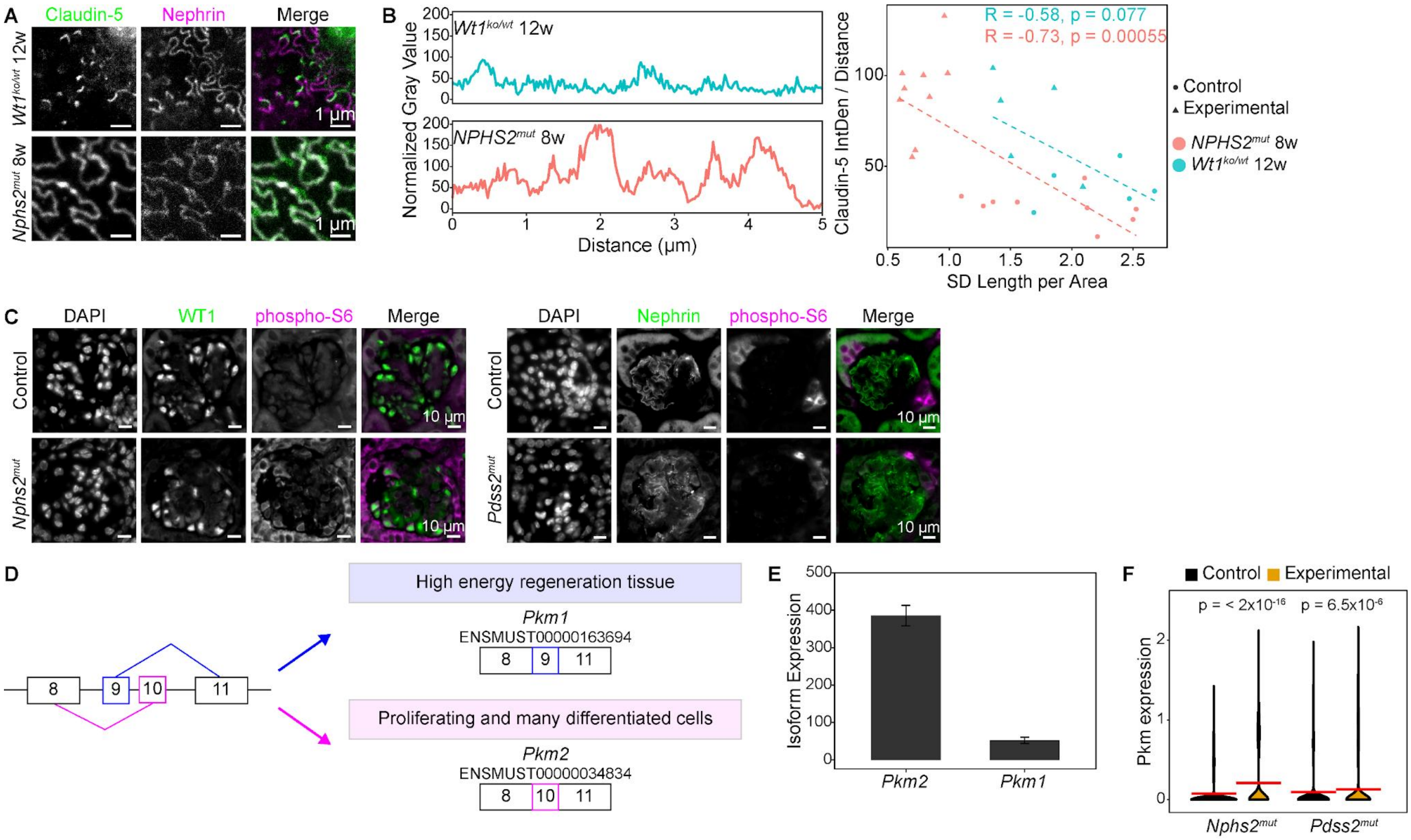

Figure S9: Validation of the PDS pathway fingerprints, related to Figure 4.
(A) Representative STED microscopy of 12-week-old *Wt1*$^{ko/wt}$ (*n* = 5 for control, *n* = 5 for experimental) or 8-week-old *Nphs2*^{mut} (*n* = 9 for control, *n* = 9 for experimental) kidneys co-stained for claudin-5 (green) and nephrin (magenta) to assess the aggregation of claudin-5 at the podocyte slit diaphragm. (B) Representative normalized line profiles of claudin-5 fluorescence intensity along manually traced slit diaphragms, illustrating differences in intensity and focal aggregation of claudin-5 along equal-length segments in *Nphs2*^{mut} (8-week-old) and *Wt1*$^{ko/wt}$ (12-week-old) experimental mice. Right: Correlation plot of normalized claudin-5 fluorescence intensity to average SD length per area for 8-week-old *Nphs2*^{mut} (*n* = 9 for control, *n* = 9 for experimental) and 12-week-old *Wt1*$^{ko/wt}$ (*n* = 5 for control, *n* = 5 for experimental) control and experimental animals. Correlation coefficient and *p*-values in Spearman are shown. (C) Representative immunofluorescence staining of control and experimental kidney tissue from either *Nphs2*^{mut} 8-week-old (left, *n* = 3 for control, *n* = 3 for experimental) or *Pdss2*^{mut} 21-week-old (right, *n* = 2 for control, *n* = 2 for experimental) showing DAPI (blue), nephrin (green) and phospho-S6 (magenta). Phospho-S6 expression is upregulated in podocytes in *Nphs2*^{mut} mice. (D) Cartoon of *Pkm1* and *Pkm2* isoforms and related dominant isoforms in respective cells or tissues. *Pkm2* is the dominant isoform in podocytes. (E) Barplot of the expression of *Pkm1* and *Pkm2* isoforms in healthy mouse glomeruli, from bulk glomerular RNA-seq of *Nphs2*^{mut} mice. Error-bars indicate 95% confidence intervals. (F) Violin plot of expression of *Pkm* in murine podocytes from *Nphs2*^{mut} and *Pdss2*^{mut} snRNA-seq experiments. Red horizontal lines indicate mean. *P*-value of Wilcoxon test of the genotype effect is displayed.

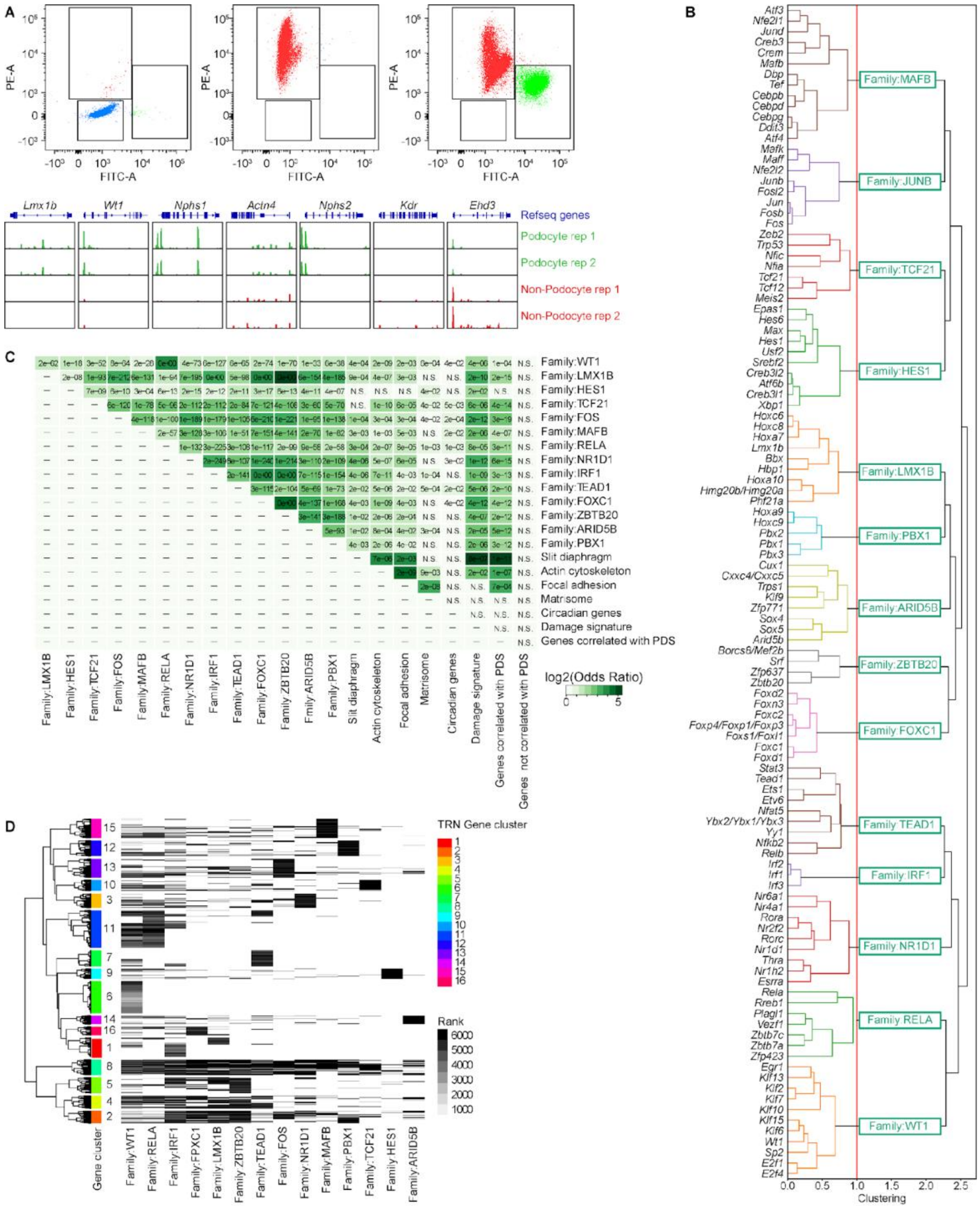

Figure S10: Development of the podocyte-specific transcription regulatory network and identification of podocyte damage pathways, related to Figure 5.

(A) Top: Flow-cytometry bivariate plots of glomerular nuclei extracted from nTnG;Pod:cre mice that express no nuclear fluorescence reporter (left), only tdTomato+ reporter (middle) or both GFP+/tdTomato+ reporter (right). Sorted nuclei were collected and used for the generation of cell type-specific ATAC-seq (see Methods). Bottom: genome browser tracks at podocyte-specific loci (*Lmx1b*, *Wt1*, *Nphs1*, *Actn4*, *Nphs2*) show accessible chromatin in podocytes, whereas those at other glomerular cell type-specific loci (*Kdr*, *Ehd3*) show accessible chromatin in non-podocytes. (B) Dendrogram of hierarchical clustering of TF motifs (PWMs). The dendrogram is cut (red line) to produce 14 TF clusters (families), each one is named by the representative motif. (C) Heatmap shows podocyte specific TRN: 14 regulatory TF-clusters (each representing a group of TFs with similar PWMs) are in columns and 6360 of their target genes expressed in podocytes are in rows. Shades of grey color shows ranks of predicted interaction score (from TFtargetCaller prediction), black and white color means the strongest and no predicted interaction, corresponding dendrogram on the left shows hierarchical clustering of genes. The dendrogram is cut to produce 16 clusters (annotated with rainbow colors), that represent common and unique targets of each of 14 regulatory modules. (D) Heatmap of overlaps of 14 TF regulons and podocyte gene sets used in Figure 5B. Color shows log2 odds ratio of the Fisher's exact test, which represents the strength of association, cell labels show labeled with adjusted P-values of the test (if *padj* < 0.05, otherwise "NS").

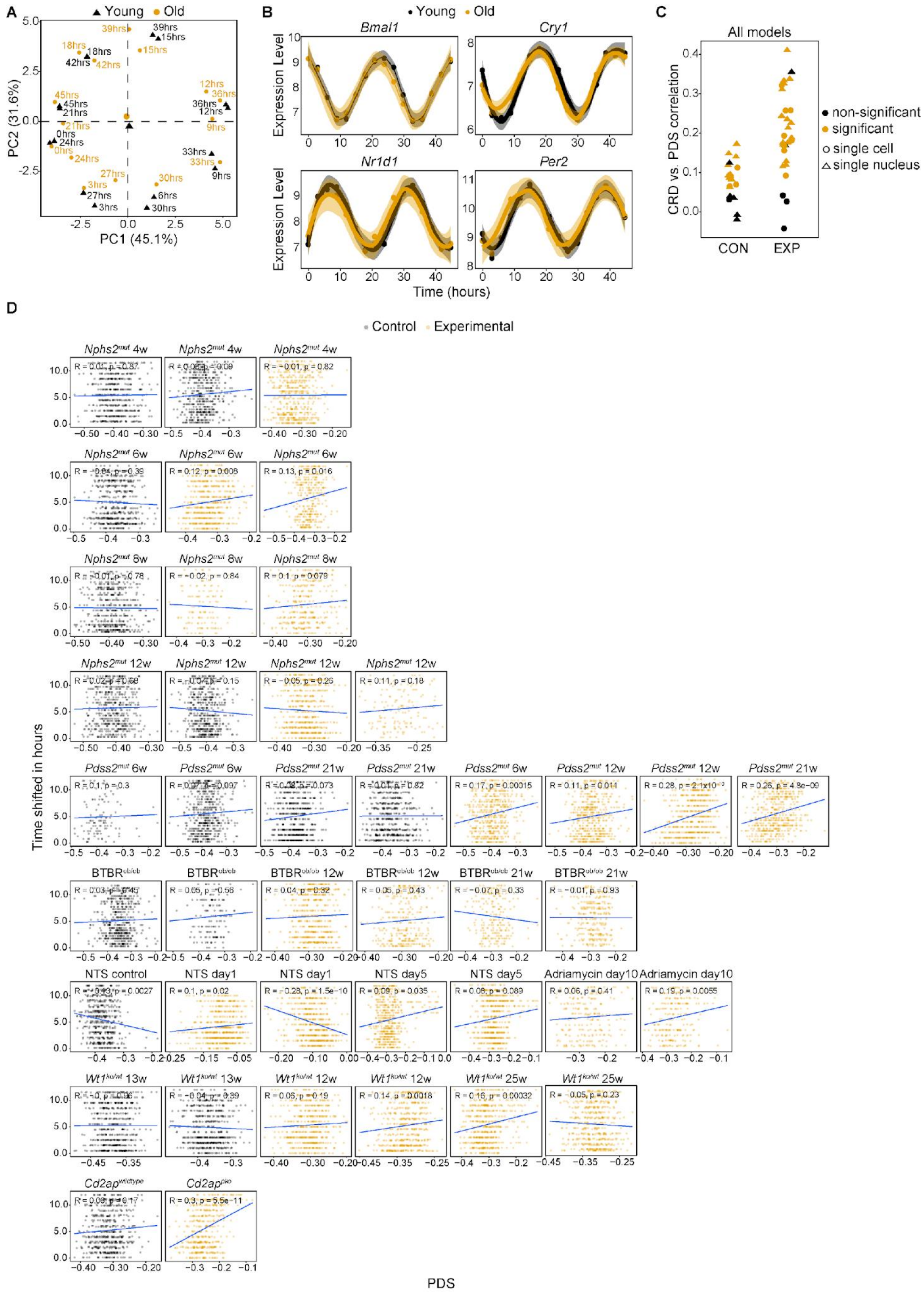

Figure S11: Transcriptome analysis of circadian gene expression, related to Figure 5.

(A) PCA of circadian bulk RNA-seq samples from whole kidneys. Each symbol represents a pooled replicate of 3 individual mice (yellow: age 78 weeks, black: age 8 weeks). Samples were taken at zeitgeber times 0, 3, ... 21 hours during 12h/12h light–dark cycles. Replicates per group (n = 2) are presented in serial fashion resulting in a 48h period. (B) Expression of circadian genes *Bmal1*, *Cry1*, *Nr1d1*, and *Per2* in the same dataset as in panel a. Y-axis - gene expression value normalized by DEseq2 rlog function, X-axis – zeitgeber time, in hours. Replicates per group (n = 2) are represented in serial fashion resulting in a 48h period. Solid line indicates an estimate of the conditional mean function, shades indicate 95% confidence interval of estimates. Yellow: age 78 weeks, black: age 8 wks. (C) Coefficients of Spearman rank correlation between PDS and CRD calculated based on podocyte-specific circadian genes in Preston et al.[23], for all individual control (CON) and experimental (EXP) samples of seven different mouse models of podocyte damage. Color distinguishes if the CRD to PDS correlation was significant ($p < 0.05$). (D) Absolute deviation between circadian time of each cell from the reference sample time (Y-axis) of the respective sample as a function of the PDS (X-axis). Regression line and parameters for Spearman rank correlation are shown. Black and orange color of dots distinguish cells of control and experimental samples. Circadian time was calculated using a Tempo algorithm,[24] *Clock* was used as a reference gene.

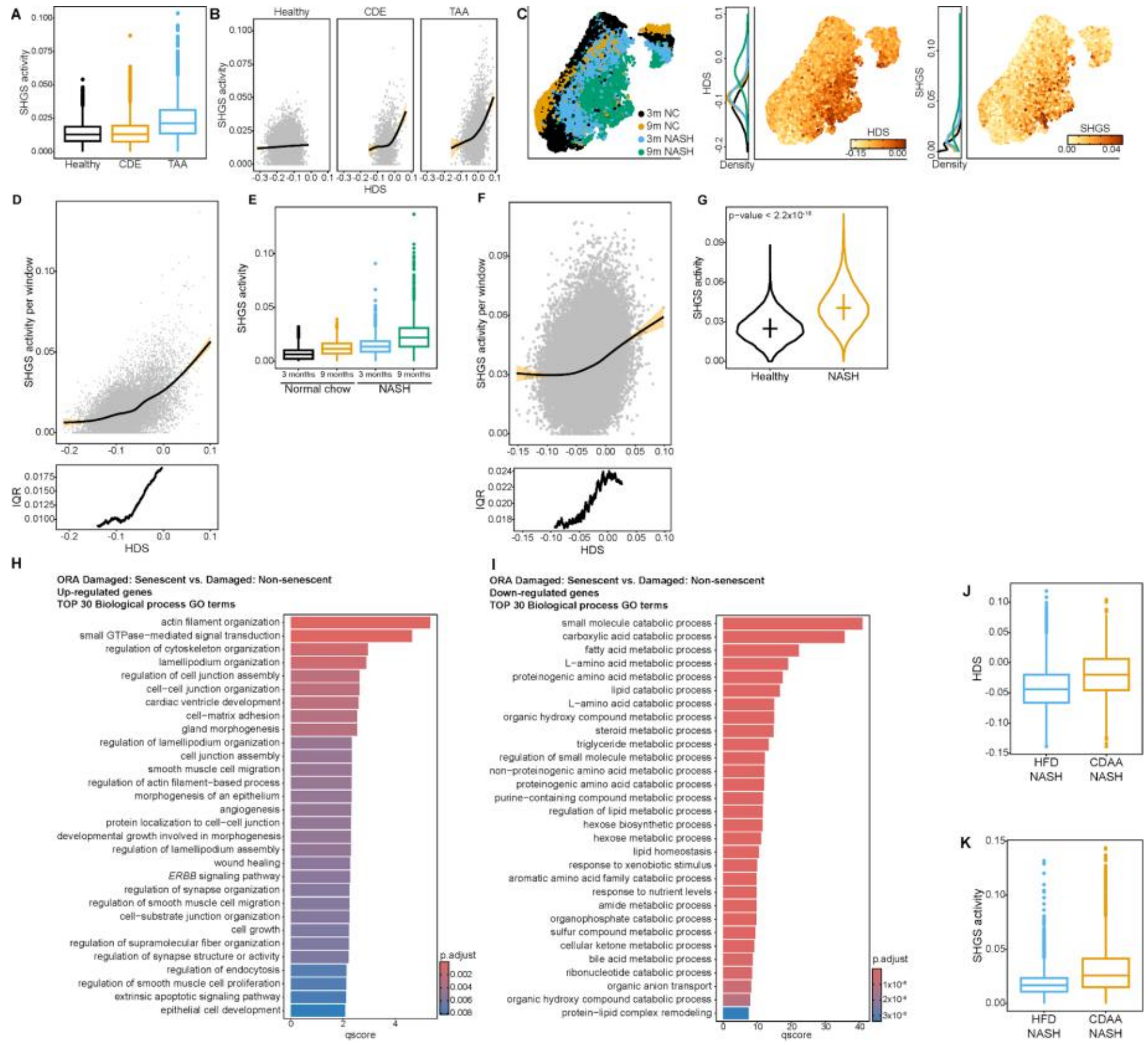

Figure S12: Analysis of hepatocyte cell state transitions, related to Figure 6.

(A) Boxplots showing activity distributions of the Senescent Hepatocyte Gene Signature (SHGS) in snRNA-seq data of mice subjected to three different treatments: Healthy: normal chow; CDE: choline-deficient, ethionine-supplemented (CDE) diet; TAA: thioacetamide (TAA) in the drinking water (GSE200366,[22] *n* = 3 biological replicates per treatment, same data as in Figure 6A). Wilcoxon rank sum test for both, comparison of TAA to controls and TAA to CDE $p < 2 \times 10^{-16}$. Wilcoxon rank sum test for comparison of CDE to controls was not significant (*p*-value = 0.052). (B) SHGS activity score versus HDS, separated by model. Same data as in Figure 6A-6B and panel A, but separated by condition. Each dot represents a hepatocyte. Solid line: Generalized linear model line showcases behavior of SHGS activity with increasing HDS. A tipping point pattern with consistent HDS threshold level is evident. Orange: confidence interval of regression line. X-axis represents the same range of HDS for all three plots. (C) UMAP of merged snRNA-seq samples from mice held in four different conditions (*n* = 1): Black: 3-month-old fed normal chow (3m NC); Orange: 9-month-old fed normal chow (9m NC): Light blue: 3-month-old fed NASH inducing diet (3m NASH); Green: 9-month-old fed NASH inducing diet (9m NASH). Left: UMAP of hepatocytes colored by condition. Middle: hepatocytes colored by HDS. The color range for the HDS has floor and ceiling values corresponding to the 1%- and 99%-quantiles of the HDS distribution across all samples. Right: Hepatocytes colored by SHGS activity score.[25] Density curves of HDS and SHGS activity score are shown per condition, color of line represents condition. Significance tests not appropriate because of sample size (*n* = 1). (D) SHGS activity score versus HDS in NASH murine snRNA-seq (same data as in panel C). Top: SHGS activity score for each hepatocyte. Solid line: Generalized linear model line showcases behavior of SHGS activity with increasing HDS. Orange: confidence interval of regression line. Bottom: IQR of SHGS activity calculated per 2000-hepatocytes sliding window (y-axis) plotted against mean HDS per sliding window. (E) Boxplots show distribution of SHGS activity per hepatocyte in each condition (*n* = 1). Same data and legend as in panel C. Statistical significance test not possible due to *n* = 1. (F) SHGS activity score versus HDS in human snRNA-seq data set of healthy and NASH patients (*n* = 3, same dataset as in Figure 3A). Top: SHGS activity score for each hepatocyte. Solid line: Generalized linear model line showcases behavior of SHGS activity with increasing HDS. Orange: confidence interval of regression line. Bottom: IQR of SHGS activity calculated per 2000-hepatocytes sliding window (y-axis) plotted against mean HDS per sliding window. (G) Violin plots show distribution of SHGS activity for all hepatocytes per patient health status (black: healthy, orange: NASH, *n* = 3). Same dataset as in Figure 3A. Horizontal lines mark the median, and vertical lines the IQR. (H) Biological Process (BP) Gene Ontology (GO) over-representation analysis (ORA) of genes significantly differentially up-regulated (*p*-value < 0.05 and LFC < -0.05) in damaged, senescent vs. Damaged, not-senescent hepatocytes. ORA results were summarized to avoid redundancy using the ClusterProfiler[26] simplify() function with default settings. Bar plot includes top 30 terms kept after filtering for adjusted *p*-value < 0.01. (I) BP-GO-ORA of genes significantly differentially down-regulated (*p*-value < 0.05 and LFC > 0.05) in damaged-senescent vs. damaged-not-senescent hepatocytes. ORA results were summarized, and bar plot created, as describe in H. (J) Boxplots of HDS distribution computed on snRNA-seq data from murine samples subject to either HFD-NASH or CDAA-NASH condition (*n* = 1).[27] (K) Boxplots of SHGS activity distribution computed on snRNA-seq data from murine samples subject to either HFD-NASH or CDAA-NASH condition (*n* = 1).[27]

Seq names	start	end	width	strand	RefSNP_id	Alleles As ambig	Genome compat	Ref allele	Alt alleles	hit	Gene symbol	Damage signature	withinGeneBody
13	71773564	71773564	1	-	rs626277	N	TRUE	A	C,G,T	TRUE	*DACH1*	PDS	TRUE
4	16155043	16155043	1	-	rs317865	D	TRUE	T	A,G	TRUE	*FGFBP1*	PDS	FALSE
6	53782834	53782834	1	+	rs149051137	H	TRUE	C	A,T	TRUE	*LRRC1*	PDS	FALSE
12	15172097	15172097	1	+	rs10846157	H	TRUE	A	C,T	TRUE	*PTPRO*	PDS	FALSE
3	77398225	77398225	1	+	rs28378063	R	TRUE	A	G	TRUE	*ROBO2*	PDS	FALSE
7	83414458	83414458	1	-	rs143538480	R	TRUE	A	G	TRUE	*SEMA3E*	PDS	FALSE
5	122438887	122438887	1	+	rs149641852	K	TRUE	G	T	TRUE	*SNCAIP*	PDS	TRUE
7	11578298	11578298	1	-	rs74614630	Y	TRUE	T	C	TRUE	*THSD7A*	PDS	TRUE
7	11586025	11586025	1	-	rs17574506	H	TRUE	C	A,T	TRUE	*THSD7A*	PDS	TRUE
11	116830406	116830406	1	-	rs2070669	V	TRUE	C	A,G	TRUE	*APOA4*	HDS	FALSE
11	116832955	116832955	1	-	rs4225	K	TRUE	G	T	TRUE	*APOA4*	HDS	FALSE
11	116937254	116937254	1	-	rs473172	R	TRUE	A	G	TRUE	*APOA4*	HDS	FALSE
7	80545045	80545045	1	+	rs139761834	Y	TRUE	T	C	TRUE	*CD36*	HDS	TRUE
7	80656687	80656687	1	+	rs75326924	Y	TRUE	C	T	TRUE	*CD36*	HDS	TRUE
1	196729333	196729333	1	+	rs6689009	R	TRUE	A	G	TRUE	*CFHR3*	HDS	FALSE
1	196752801	196752801	1	+	rs371960809	W	TRUE	A	T	TRUE	*CFHR3*	HDS	FALSE
1	196706226	196706226	1	+	rs2300429	R	TRUE	G	A	TRUE	*CFHR3*	HDS	FALSE
22	23992517	23992517	1	-	rs140287	V	TRUE	G	A,C	TRUE	*DDT*	HDS	TRUE
22	24126177	24126177	1	-	rs73167538	H	TRUE	A	C,T	TRUE	*DDT*	HDS	TRUE
22	24175884	24175884	1	-	rs75303441	R	TRUE	G	A	TRUE	*DDT*	HDS	TRUE
22	24143401	24143401	1	-	rs117001360	R	TRUE	G	A	TRUE	*DDT*	HDS	TRUE
22	24066229	24066229	1	-	rs112234981	R	TRUE	A	G	TRUE	*DDT*	HDS	TRUE
22	24132091	24132091	1	-	rs735863	Y	TRUE	T	C	TRUE	*DDT*	HDS	TRUE
22	24087164	24087164	1	-	rs78365374	D	TRUE	G	A,T	TRUE	*DDT*	HDS	TRUE
22	24072436	24072436	1	-	rs17854874	R	TRUE	G	A	TRUE	*DDT*	HDS	TRUE
22	24178086	24178086	1	-	rs144524781	R	TRUE	G	A	TRUE	*DDT*	HDS	TRUE
22	24008132	24008132	1	-	rs405597	M	TRUE	A	C	TRUE	*DDT*	HDS	TRUE
22	24114019	24114019	1	-	rs111736007	Y	TRUE	C	T	TRUE	*DDT*	HDS	TRUE
22	24142922	24142922	1	-	rs116954205	Y	TRUE	C	T	TRUE	*DDT*	HDS	TRUE
22	24209757	24209757	1	-	rs139650685	R	TRUE	A	G	TRUE	*DDT*	HDS	TRUE
22	24169000	24169000	1	-	rs149418007	R	TRUE	G	A	TRUE	*DDT*	HDS	TRUE
22	24101661	24101661	1	-	rs4820582	S	TRUE	G	C	TRUE	*DDT*	HDS	TRUE
22	24080848	24080848	1	-	rs529389498	Y	TRUE	T	C	TRUE	*DDT*	HDS	TRUE
22	24152950	24152950	1	-	rs531196227	D	TRUE	G	A,T	TRUE	*DDT*	HDS	TRUE

22	24099093	24099093	1	-	rs552616617	B	TRUE	T	C,G	TRUE	*DDT*	HDS	TRUE
22	24111668	24111668	1	-	rs78960173	Y	TRUE	T	C	TRUE	*DDT*	HDS	TRUE
13	113849020	113849020	1	-	rs6602909	B	TRUE	T	C,G	TRUE	*GAS6*	HDS	TRUE
13	113842344	113842344	1	-	rs9577924	R	TRUE	A	G	TRUE	*GAS6*	HDS	TRUE
13	113862741	113862741	1	-	rs6602904	Y	TRUE	C	T	TRUE	*GAS6*	HDS	TRUE
16	81826596	81826596	1	+	rs76424446	S	TRUE	C	G	TRUE	*HSD17B2*	HDS	FALSE
15	90228773	90228773	1	+	rs11547962	V	TRUE	G	A,C	TRUE	*IQGAP1*	HDS	FALSE
15	90211624	90211624	1	+	rs1533325	Y	TRUE	C	T	TRUE	*IQGAP1*	HDS	FALSE
15	90275237	90275237	1	+	rs12595409	Y	TRUE	C	T	TRUE	*IQGAP1*	HDS	FALSE
17	48065680	48065680	1	-	rs7209484	B	TRUE	T	C,G	TRUE	*SCRN2*	HDS	FALSE
5	172438076	172438076	1	-	rs10067250	S	TRUE	G	C	TRUE	*STK10*	HDS	FALSE
17	1921011	1921011	1	-	rs4790292	M	TRUE	C	A	TRUE	*TLCD2*	HDS	FALSE

Table S3: Kidney and liver disease GWAS associations of podocyte and hepatocyte damage marker genes, related to Figure 1. PDS and HDS marker gene loci were intersected with kidney- and liver-disease-associated variants from the NHGRI-EBI GWAS Catalog using canonical RefSeq gene bodies and extended regulatory regions. The table reports overlapping disease-associated variants, corresponding traits, affected marker genes, and enrichment statistics relative to expressed kidney or liver background genes.

**Abbreviation in figure**	**Explanation in figure caption**	**Full description for methods**
Nphs2^{mut}	*Nphs2*$^{R231Q/A286V}$ mutant	*Nphs2*$^{R231Q/A286V}$ compound heterozygous mutant mice (Butt et al.[16])
Wt1$^{ko/wt}$	*Wt1* heterozygous	*Wt1* heterozygous whole-body knockout (Kann et al.[28])
Pdss2^{mut}	*Pdss2*$^{V117M/V117M}$ mutant	*Pdss2*$^{V117M/V117M}$ homozygous mutant mice generated by CRISPR strategy.
BTBR$^{ob/ob}$	Diabetic nephropathy	BTBR.Cg-*Lep*^{ob}/WiscJ, Data taken from Chung et al. (Chung et al.[1])
Cd2ap^{pko}	Conditional podocyte *Cd2ap* knock-out	*Nphs2*^{Cre} x *Cd2ap*$^{fl/fl}$, data taken from Chung et al. (Chung et al.[1])
Adria	Adriamycin toxicity model	Data taken from Chung et al. (Chung et al.[1])
NTSN	Nephrotoxic serum nephritis	Data taken from Chung et al. (Chung et al.[1])

Table S4: Description of FSGS damage models and corresponding scRNA-seq datasets used in the manuscript, related to Figure 4.

Supplemental references

[S1] Chung, J.J., Goldstein, L., Chen, Y.J.J., Lee, J., Webster, J.D., Roose-Girma, M., Paudyal, S.C., Modrusan, Z., Dey, A., and Shaw, A.S. (2020). Single-cell transcriptome profiling of the kidney glomerulus identifies key cell types and reactions to injury. Journal of the American Society of Nephrology *31*, 2341–2354. https://doi.org/10.1681/ASN.2020020220/-/DCSUPPLEMENTAL.

[S2] Guilliams, M., Bonnardel, J., Haest, B., Vanderborght, B., Wagner, C., Remmerie, A., Bujko, A., Martens, L., Thoné, T., Browaeys, R., et al. (2022). Spatial proteogenomics reveals distinct and evolutionarily conserved hepatic macrophage niches. Cell *185*, 379-396.e38. https://doi.org/10.1016/J.CELL.2021.12.018.

[S3] Habenicht, L.K.L., Wang, Z., Zhang, X., Li, Y., Mogler, C., Huspenina, J.S., Schmid, R.M., Weber, C., Mohanta, S.K., Ma, Z., et al. (2022). The C1q-ApoE complex: A new hallmark pathology of viral hepatitis and nonalcoholic fatty liver disease. Front. Immunol. *13*. https://doi.org/10.3389/FIMMU.2022.970938.

[S4] Xiao, Y., Batmanov, K., Hu, W., Zhu, K., Tom, A.Y., Guan, D., Jiang, C., Cheng, L., McCright, S.J., Yang, E.C., et al. (2023). Hepatocytes demarcated by EphB2 contribute to the progression of nonalcoholic steatohepatitis. Sci. Transl. Med. *15*. https://doi.org/10.1126/SCITRANSLMED.ADC9653.

[S5] Hatje, F.A., Wedekind, U., Sachs, W., Loreth, D., Reichelt, J., Demir, F., Kosub, C., Heintz, L., Tomas, N.M., Huber, T.B., et al. (2021). Tripartite separation of glomerular cell types and proteomes from reporter-free mice. Journal of the American Society of Nephrology *32*, 2175–2193. https://doi.org/10.1681/ASN.2020091346/-/DCSUPPLEMENTAL.

[S6] Chittka, D., Banas, B., Lennartz, L., Putz, F.J., Eidenschink, K., Beck, S., Stempfl, T., Moehle, C., Reichelt-Wurm, S., and Banas, M.C. (2018). Long-term expression of glomerular genes in diabetic nephropathy. Nephrol. Dial. Transplant *33*, 1533–1544. https://doi.org/10.1093/ndt/gfx359.

[S7] Bird, T.G., M ller, M., Boulter, L., Vincent, D.F., Ridgway, R.A., Lopez-Guadamillas, E., Lu, W.Y., Jamieson, T., Govaere, O., Campbell, A.D., et al. (2018). TGFβ inhibition restores a regenerative response in acute liver injury by suppressing paracrine senescence. Sci. Transl. Med. *10*. https://doi.org/10.1126/SCITRANSLMED.AAN1230.

[S8] Zhong, Y., Lee, K., Deng, Y., Ma, Y., Chen, Y., Li, X., Wei, C., Yang, S., Wang, T., Wong, N.J., et al. (2019). Arctigenin attenuates diabetic kidney disease through the activation of PP2A in podocytes. Nat. Commun. *10*, 4523. https://doi.org/10.1038/s41467-019-12433-w.

[S9] Schaum, N., Karkanias, J., Neff, N.F., May, A.P., Quake, S.R., Wyss-Coray, T., Darmanis, S., Batson, J., Botvinnik, O., Chen, M.B., et al. (2018). Single-cell transcriptomics of 20 mouse organs creates a Tabula Muris. Nature *562*, 367–372. https://doi.org/10.1038/S41586-018-0590-4.

[S10] Cao, A., Li, J., Asadi, M., Basgen, J.M., Zhu, B., Yi, Z., Jiang, S., Doke, T., El Shamy, O., Patel, N., et al. (2021). DACH1 protects podocytes from experimental diabetic injury and modulates PTIP-H3K4Me3 activity. J. Clin. Invest. *131*. https://doi.org/10.1172/JCI141279.

[S11] Schaum, N., Lehallier, B., Hahn, O., Pálovics, R., Hosseinzadeh, S., Lee, S.E., Sit, R., Lee, D.P., Losada, P.M., Zardeneta, M.E., et al. (2020). Ageing hallmarks exhibit organ-specific temporal signatures. Nature *583*, 596–602. https://doi.org/10.1038/S41586-020-2499-Y.

[S12] Okabe, M., Motojima, M., Miyazaki, Y., Pastan, I., Yokoo, T., and Matsusaka, T. (2019). Global polysome analysis of normal and injured podocytes. Am. J. Physiol. Renal Physiol. *316*, F241–F252. https://doi.org/10.1152/ajprenal.00115.2018.

[S13] Marshall, J.L., Noel, T., Wang, Q.S., Chen, H., Murray, E., Subramanian, A., Vernon, K.A., Bazua-Valenti, S., Liguori, K., Keller, K., et al. (2022). High-resolution Slide-seqV2 spatial transcriptomics enables discovery of disease-specific cell neighborhoods and pathways. iScience *25*. https://doi.org/10.1016/J.ISCI.2022.104097.

[S14] Talyan, S., Filipów, S., Ignarski, M., Smieszek, M., Chen, H., Kühne, L., Butt, L., Göbel, H., Hoyer-Allo, K.J.R., Koehler, F.C., et al. (2021). CALINCA-A Novel Pipeline for the Identification of lncRNAs in Podocyte Disease. Cells *10*, 1–17. https://doi.org/10.3390/CELLS10030692.

[S15] Ettou, S., Jung, Y.L., Miyoshi, T., Jain, D., Hiratsuka, K., Schumacher, V., Taglienti, M.E., Morizane, R., Park, P.J., and Kreidberg, J.A. (2020). Epigenetic transcriptional reprogramming by WT1 mediates a repair response during podocyte injury. Sci. Adv. *6*, eabb5460. https://doi.org/10.1126/sciadv.abb5460.

[S16] Butt, L., Unnersjö-Jess, D., Höhne, M., Edwards, A., Binz-Lotter, J., Reilly, D., Hahnfeldt, R., Ziegler, V., Fremter, K., Rinschen, M.M., et al. (2020). A molecular mechanism explaining albuminuria in kidney disease. Nat. Metab. *2*, 461–474. https://doi.org/10.1038/S42255-020-0204-Y.

[S17] Hengel, F.E., Dehde, S., Lassé, M., Zahner, G., Seifert, L., Schnarre, A., Kretz, O., Demir, F., Pinnschmidt, H.O., Grahammer, F., et al. (2024). Autoantibodies Targeting Nephrin in Podocytopathies. N. Engl. J. Med. *391*, 422–433. https://doi.org/10.1056/NEJMoa2314471.

[S18] Koehler, S., Kuczkowski, A., Kuehne, L., Jüngst, C., Hoehne, M., Grahammer, F., Eddy, S., Kretzler, M., Beck, B.B., Höhfeld, J., et al. (2020). Proteome Analysis of Isolated Podocytes Reveals Stress Responses in Glomerular Sclerosis. J. Am. Soc. Nephrol. *31*, 544–559. https://doi.org/10.1681/ASN.2019030312.

[S19] Williams, E.G., Pfister, N., Roy, S., Statzer, C., Haverty, J., Ingels, J., Bohl, C., Hasan, M., Čuklina, J., Bühlmann, P., et al. (2022). Multiomic profiling of the liver across diets and age in a diverse mouse population. Cell Syst. *13*, 43-57.e6. https://doi.org/10.1016/j.cels.2021.09.005.

[S20] Lake, B.B., Menon, R., Winfree, S., Hu, Q., Ferreira, R.M., Kalhor, K., Barwinska, D., Otto, E.A., Ferkowicz, M., Diep, D., et al. (2023). An atlas of healthy and injured cell states and niches in the human kidney. Nature *619*, 585–594. https://doi.org/10.1038/S41586-023-05769-3.

[S21] Latt, K.Z., Heymann, J., Jessee, J.H., Rosenberg, A.Z., Berthier, C.C., Arazi, A., Eddy, S., Yoshida, T., Zhao, Y., Chen, V., et al. (2021). Urine Single-Cell RNA Sequencing in Focal Segmental Glomerulosclerosis Reveals Inflammatory Signatures. Kidney Int. Rep. *7*, 289–304. https://doi.org/10.1016/J.EKIR.2021.11.005.

[S22] Carlessi, R., Denisenko, E., Boslem, E., Köhn-Gaone, J., Main, N., Abu Bakar, N.D.B., Shirolkar, G.D., Jones, M., Beasley, A.B., Poppe, D., et al. (2023). Single-nucleus RNA sequencing of pre-malignant liver reveals disease-associated hepatocyte state with HCC prognostic potential. Cell genomics *3*, 100301. https://doi.org/10.1016/j.xgen.2023.100301.

[S23] Preston, R., Chrisp, R., Dudek, M., Morais, M.R.P.T., Tian, P., Williams, E., Naylor, R.W., Davenport, B., Pathiranage, D.R.J., Benson, E., et al. (2024). The glomerular circadian clock temporally regulates basement membrane dynamics and the podocyte glucocorticoid response. Kidney Int. https://doi.org/10.1016/J.KINT.2024.10.016.

[S24] Auerbach, B.J., FitzGerald, G.A., and Li, M. (2022). Tempo: an unsupervised Bayesian algorithm for circadian phase inference in single-cell transcriptomics. Nat. Commun. *13*. https://doi.org/10.1038/S41467-022-34185-W.

[S25] Du, K., Umbaugh, D.S., Wang, L., Jun, J.H., Dutta, R.K., Oh, S.H., Ren, N., Zhang, Q., Ko, D.C., Ferreira, A., et al. (2025). Targeting senescent hepatocytes for treatment of metabolic dysfunction-associated steatotic liver disease and multi-organ dysfunction. Nat. Commun. *16*, 3038. https://doi.org/10.1038/s41467-025-57616-w.

[S26] Yu, G., Wang, L.G., Han, Y., and He, Q.Y. (2012). clusterProfiler: an R package for comparing biological themes among gene clusters. OMICS *16*, 284–287. https://doi.org/10.1089/OMI.2011.0118.

[S27] Vesting, A.J., Jais, A., Klemm, P., Steuernagel, L., Wienand, P., Fog-Tonnesen, M., Hvid, H., Schumacher, A.-L., Kukat, C., Nolte, H., et al. (2022). NIK/MAP3K14 in hepatocytes orchestrates NASH to hepatocellular carcinoma progression via JAK2/STAT5 inhibition. Mol. Metab. *66*, 101626. https://doi.org/10.1016/j.molmet.2022.101626.

[S28] Kann, M., Ettou, S., Jung, Y.L., Lenz, M.O., Taglienti, M.E., Park, P.J., Schermer, B., Benzing, T., and Kreidberg, J.A. (2015). Genome-wide analysis of Wilms' tumor 1-controlled gene expression in podocytes reveals key regulatory mechanisms. Journal of the American Society of Nephrology *26*, 2097–2104. https://doi.org/10.1681/ASN.2014090940/-/DCSUPPLEMENTAL.
