# Supplementary material for: Cell-type-specific damage scores reveal kidney and liver disease trajectories in single-cell and spatial transcriptomics
Source: Cell Genom. 2026 Aug 20;6(10):101337. doi: 10.1016/j.xgen.2026.101337 (PMC13689510; doi:10.1016/j.xgen.2026.101337)
Supplement: Supplementary file 2 [file mmc7.pdf]

Cell Genomics

Article

Cell-type-specific damage scores reveal kidney and liver disease trajectories in single-cell and spatial transcriptomics

Graphical abstract

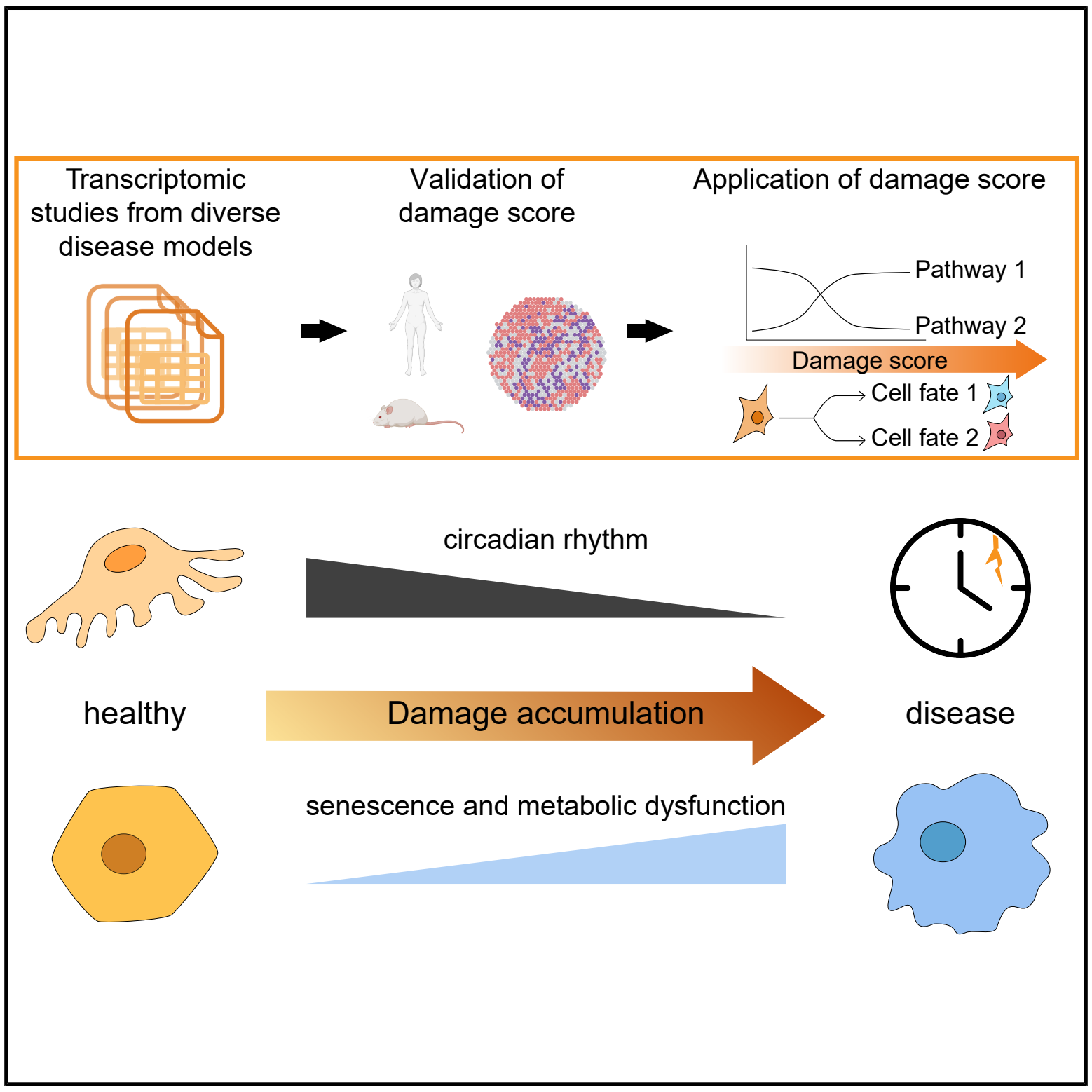

Authors

Tsimafei Padvitski, Paula Unger Avila, He Chen, ..., Frank Thomas Wunderlich, Martin Kann, Andreas Beyer

Correspondence

martin.kann@uk-koeln.de (M.K.), andreas.beyer@uni-koeln.de (A.B.)

In brief

Padvitski, Unger Avila, Chen et al. introduce a single-cell transcriptomic framework that quantifies progressive cellular damage across degenerative disease models. Applied to kidney and liver injury, the approach reveals conserved damage trajectories, circadian disruption in podocytes, and a hepatocyte damage threshold linked to senescence and metabolic dysfunction.

Highlights

- A generalizable concept for deriving single-cell damage scores
- Single-cell damage scores facilitate the analysis of degenerative disease progression
- In podocytes, injury associates with disruption in circadian gene expression
- A damage threshold in hepatocytes marks the onset of senescence

Padvitski et al., 2026, Cell Genomics *6*, 101337
October 14, 2026 © 2026 The Authors. Published by Elsevier Inc.
https://doi.org/10.1016/j.xgen.2026.101337

CellPress

Cell Genomics

CellPress OPEN ACCESS

Article

Cell-type-specific damage scores reveal kidney and liver disease trajectories in single-cell and spatial transcriptomics

Tsimafei Padvitski,[1,15] Paula Unger Avila,[1,15] He Chen,[2,3,15] Cem Özel,[2,3] Fabian Braun,[4,5,6] Marie-Kristin Kroll,[2,3] Christian Vossen,[7] Peter Wienand,[7] Linus Butt,[2,3] David Unnersjö-Jess,[2,3,8] Anna-Lena Schumacher,[9] Kat Folz-Donahue,[9] Heike Göbel,[10] Christoph Kuppe,[11] Roman-Ulrich Müller,[1,2,3] Pål Olof Westermark,[12,13] Paul Thomas Brinkkötter,[2,3] Bernhard Schermer,[1,2,3] Thomas Benzing,[1,2,3] Frank Thomas Wunderlich,[7] Martin Kann,[2,3,15,16,*] and Andreas Beyer[1,3,14,15,16,17,*]

[1]Cologne Excellence Cluster on Cellular Stress Responses in Age-Associated Diseases (CECAD), University of Cologne, 50931 Cologne, North Rhine-Westphalia, Germany

[2]Department II of Internal Medicine, University of Cologne, Faculty of Medicine and University Hospital Cologne, 50937 Cologne, North Rhine-Westphalia, Germany

[3]Center for Molecular Medicine Cologne, Faculty of Medicine and University Hospital Cologne, University of Cologne, 50931 Cologne, North Rhine-Westphalia, Germany

[4]III. Department of Medicine, University Medical Center Hamburg-Eppendorf, Hamburg, 20246 Hamburg, Germany

[5]Hamburg Center for Kidney Health, University Medical Center Hamburg-Eppendorf, Hamburg, 20246 Hamburg, Germany

[6]Martin Zeitz Center for Rare Diseases, University Medical Center Hamburg-Eppendorf, Hamburg, 20246 Hamburg, Germany

[7]Max Planck Institute for Metabolism Research, 50931 Cologne, North Rhine-Westphalia, Germany

[8]Science for Life Laboratory, Department of Applied Physics, Royal Institute of Technology, 17165 Solna, Stockholm County, Sweden

[9]Max Planck Institute for Biology of Aging, 50931 Cologne, North Rhine-Westphalia, Germany

[10]Institute of Pathology, University Hospital of Cologne, 50937 Cologne, North Rhine-Westphalia, Germany

[11]Division of Nephrology and Clinical Immunology, RWTH Aachen University, 52074 Aachen, North Rhine-Westphalia, Germany

[12]Working Group Husbandry & Health, Research Institute for Farm Animal Biology (FBN), 18196 Dummerstorf, Mecklenburg-Vorpommern, Germany

[13]Institute for Biostatistics and Informatics in Medicine and Ageing Research, Rostock University Medical Center, 18057 Rostock, Mecklenburg-Vorpommern, Germany

[14]Institute for Genetics, Faculty of Mathematics and Natural Sciences, University of Cologne, 50931 Cologne, North Rhine-Westphalia, Germany

[15]These authors contributed equally

[16]Senior author

[17]Lead contact

*Correspondence: martin.kann@uk-koeln.de (M.K.), andreas.beyer@uni-koeln.de (A.B.)

https://doi.org/10.1016/j.xgen.2026.101337

SUMMARY

Degenerative diseases progress through gradual cell-intrinsic damage that is difficult to resolve with bulk transcriptomics or discrete cell-state analysis. We introduce a generalizable single-cell and spatial transcriptomics framework that quantifies continuous damage trajectories *in vivo* using cell-type-specific scores. Applied to chronic kidney disease and metabolic dysfunction-associated steatotic liver disease, the podocyte damage score (PDS) and hepatocyte damage score (HDS) place individual podocytes and hepatocytes on a health-to-damage axis. Across mouse and human single-cell RNA sequencing (scRNA-seq), single-nucleus RNA-seq (snRNA-seq), spatial transcriptomics, proteomics, histology, and clinical datasets, PDS and HDS robustly detect disease-associated damage, align disease models as well as unperturbed cells, and reveal conserved pathways. The PDS links podocyte injury and FSGS progression to circadian gene-expression disruption. The HDS identifies a damage threshold in metabolic dysfunction-associated steatotic liver disease (MASLD)/NASH beyond which hepatocytes adopt senescent, metabolically dysfunctional states. These damage scores enable scalable mapping of degenerative disease progression and stage-specific therapeutic targets.

INTRODUCTION

Degenerative diseases arise when cells progressively accumulate molecular damage that ultimately compromises tissue and organ function. Degeneration underlies many chronic diseases as well as aging-related decline, yet the molecular trajectories that connect early perturbations to terminal cellular failure remain poorly understood. A central challenge is the ability to

This is an open access article under the CC BY license (http://creativecommons.org/licenses/by/4.0/).

quantify and track cellular deterioration as a continuous process rather than discrete states.

Chronic kidney disease (CKD) and metabolic dysfunction-associated steatotic liver disease (MASLD; formerly nonalcoholic fatty liver disease [NAFLD]) are prominent examples of disorders in which cellular injury occurs gradually over time.[1,2] Despite their clinical importance, how metabolic and molecular stress drives progressive cellular degeneration remains poorly understood. Early deviations occur in specific parenchymal cell types long before organ-level metrics or conventional histopathology capture them.[2,3] Conventional bulk analyses and histopathological analysis cannot resolve the continuum of cellular damage accumulation that underlies degenerative disease. Yet, detecting these deviations early—before damage becomes self-sustaining—is critical for defining intervention windows, stratifying patients, and monitoring treatment response. Moreover, existing animal models frequently mimic rare, monogenic forms of disease and fail to reflect the complexity of sporadic, multifactorial conditions in humans.

In CKD, the progressive decline of kidney function is tightly linked to injury to podocytes, terminally differentiated epithelial cells that maintain the integrity of the glomerular filtration barrier. Recent large-scale transcriptomic approaches in human kidneys have revealed disease-associated glomerular transcriptional programs; however, these snapshots provide limited insight into how podocytes gradually progress to degeneration.[1–5] Similarly, MASLD involves a complex interplay between liver cell types, but its defining criterion is damage to the predominant hepatic epithelial cell type, the hepatocyte.[6] Hepatocytes are key to metabolic functions of the liver and are tightly connected to inflammatory and immune responses. However, pathways of gradual degeneration and ultimately carcinogenesis are still elusive.[7,8]

Advances in single-cell RNA sequencing (scRNA-seq) and spatial transcriptomics have revolutionized our ability to profile gene expression at cellular resolution, offering new opportunities to dissect disease processes in unprecedented detail and study the function of disease-associated genes[9] and regulatory events.[10] However, current analytical strategies primarily classify cells into discrete states such as cell types or activation states. In contrast, many biological processes, including degeneration, unfold gradually along continuous trajectories. In chronic disease, the critical question is therefore often not which state a cell most closely resembles but how far it has progressed along a deterioration continuum and whether it is approaching a point beyond which recovery is unlikely. Because scRNA-seq datasets do not directly capture physiological damage, existing traditional clustering, differential expression analyses, and pseudotime trajectory methods struggle to distinguish pathological decline from normal physiology. As a result, reconstructing the entire continuum of disease-associated cellular states spanning from healthy to advanced degeneration remains a major challenge.

What is needed is a scalar measure of cell-intrinsic deterioration: a continuous coordinate that places each cell on a health-to-damage axis while remaining robust across datasets and compatible with limited tissue. For such a measure to be useful, it must meet several criteria. It should capture a damage-associated program that emerges across heterogeneous disease triggers while permitting discovery of etiology-specific mechanisms once cells are matched for comparable damage levels. It must generalize to settings not used during construction, and the resulting coordinate must relate to independent functional, structural, and spatial readouts. Finally, it should be compact enough to transfer across platforms and to support translation into targeted assays.

Here, we introduce a generalizable approach to quantify cellular damage at single-cell resolution. By integrating established single-cell scoring methods with a generalizable strategy for selecting molecular damage markers, we develop cell-type-specific damage scores—namely, the podocyte damage score (PDS) and hepatocyte damage score (HDS)—that systematically track molecular deterioration in kidney and liver disease. We evaluate damage scores as a measurement instrument rather than as a restatement of case-control differences. These scores represent *in vivo* cell states in chronic disease over extended time periods and enable the identification of molecular pathways associated with progressive damage, even within rare cell types or early-stage diseased cells. Using this framework, we uncover circadian rhythm disruption in podocytes and define a critical threshold of hepatocyte damage associated with senescence and metabolic dysfunction.

Our approach is transferable to other chronic diseases and offers a powerful tool for mapping cellular damage trajectories, providing critical insights into disease progression and potential therapeutic targets across degenerative conditions.

RESULTS

Cellular damage can be defined in different ways. Here, we operationally define "cellular damage" as a pathological cellular state that is not specific to any disease-causing mechanism. Instead, damage is a common pathological cellular state resulting from a broader spectrum of disease entities, disease-causing mechanisms, and/or a loss of homeostasis. Such cell-type-specific cellular damage states are well established in histopathology. Here, we develop an approach for the molecular characterization of such states. To demonstrate the general applicability of our concept, we developed two single-cell damage scores: one for podocytes (PDS), to track damage during the progression of CKD, and a second for hepatocytes (HDS), to explore progressive damage in chronic liver pathologies such as MASLD. In the following, we first motivate the selection criteria for damage marker genes and subsequently validate both scores using mouse and human single-cell and spatial omics data. Finally, we present applications of first the PDS and then the HDS to demonstrate their added value and derive mechanistic insight gained from these scores.

The key notion of our approach is to identify marker genes whose expression is associated with increasing damage across a wide range of disease models (Figure 1A). By combining diverse disease models in the selection process, we ensure that the marker genes are not just markers for one specific intervention, but report on damage of the target cell type in general. The damage scores combine information from dozens of marker genes, which increases robustness and reduces the impact of

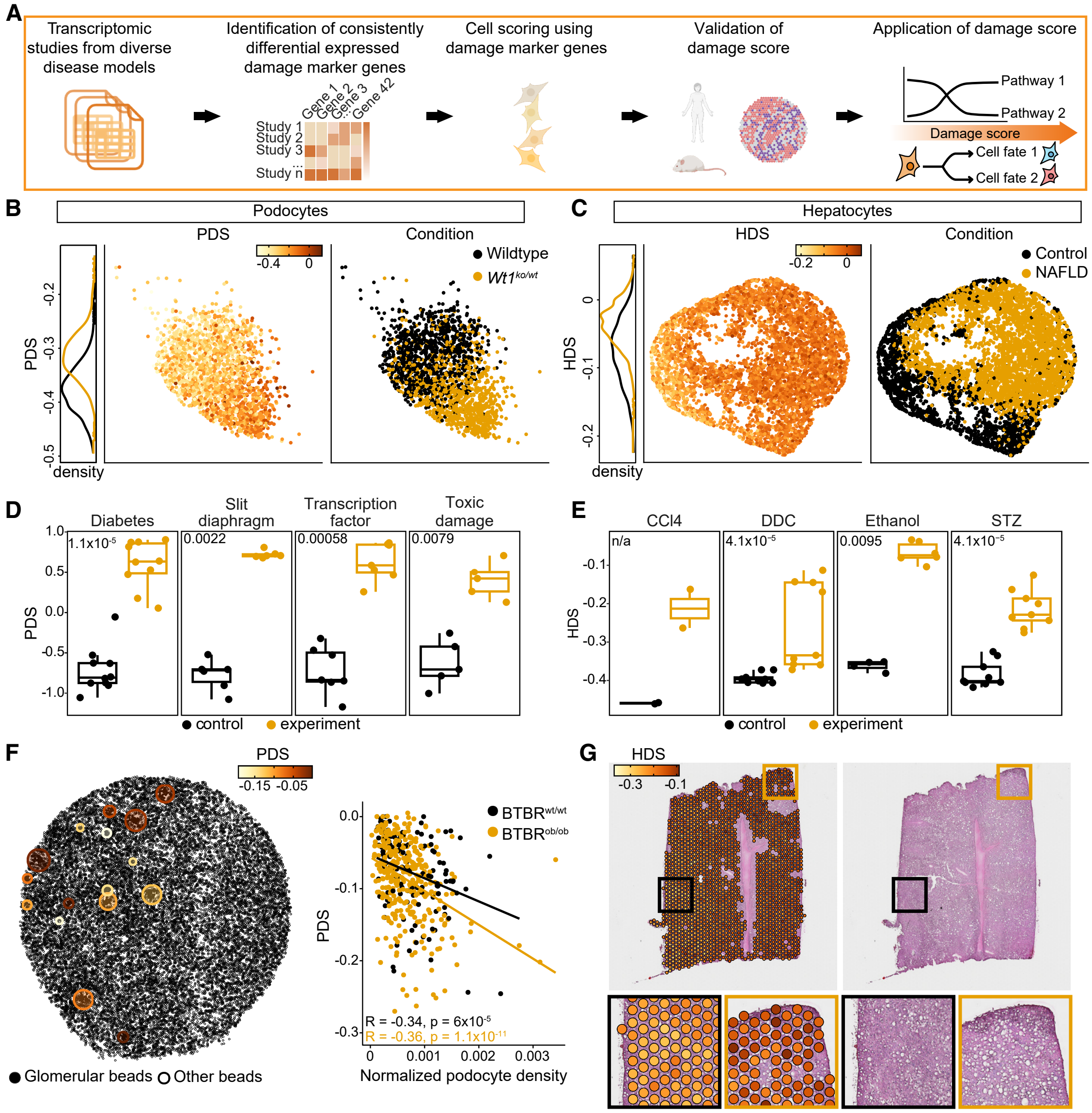

Figure 1. A strategy to quantify damage at the single-cell level based on transcriptomic data

(A) Robust damage marker genes are identified by compiling transcriptomic data from a great diversity of disease models. The top genes that change consistently across models are selected as damage markers. Damage of individual cells is quantified using a rank-based approach. After validation, the damage scores are used to analyze the molecular progression of chronic degenerative diseases *in vivo*.

(B) Application of the podocyte damage score (PDS) to podocytes from snRNA-seq data of the $Wt1^{ko/wt}$ mouse model (a podocyte damage model); $n = 6$. The left UMAP is colored by PDS (see color scale), and the right UMAP is colored by condition. The PDS density plot (left) shows the distribution of PDS values separated by experimental condition (same colors as the UMAP on the right).

(C) Application of the hepatocyte damage score (HDS) to hepatocytes of snRNA-seq data from 24-week-old mice fed with either a standard diet (control) or an NAFLD-inducing diet (NAFLD); $n = 5$.[8,12] The left UMAP is colored by HDS (see color scale), and the right UMAP is colored by diet. HDS density plot (left) shows the distribution of HDS values separated by experimental condition (same colors as the UMAP on the right).

(D) Leave-one-model-out cross-validation of the PDS. All data from the indicated disease models were left out during the creation of a variant damage signature and used afterward to test PDSs in the indicated disease models (see also Table S1 for grouping). Each dot represents the average damage score per sample. Scores are scaled and centered per study ($n = 20$, 12, 14, and 10). *p* values of the two-sided Wilcoxon rank test (experimental vs. control conditions) are shown. The boxplots show first (Q1), second (Q2), and third (Q3) quartiles of the distribution. Whiskers represent the Q1 $-$ 1.5$\times$ interquartile range (IQR) and Q3 + 1.5$\times$ IQR.

(E) Leave-one-model-out cross-validation of the HDS. The procedure is the same as that in (C). Each dot represents the HDS of one sample. CCl4: hepatic toxic injury by CCl4 injection and DDC: hepatotoxin-induced cholestatic liver injury via 3,5-diethoxycarbonyl-1,4-dihydrocollidine; $n = 9$. Ethanol: alcoholic liver injury via ethanol administration (control $n = 4$, experiment $n = 6$) and STZ: streptozotocin-induced liver injury ($n = 9$). Boxplots show first (Q1), second (Q2), and third (Q3) quartiles of distribution. Whiskers represent Q1 $-$ 1.5$\times$ IQR and Q3 + 1.5$\times$ IQR. *p* values of the two-sided Wilcoxon rank test (experimental vs. control conditions) are shown. n/a, statistics not applicable due to $n = 2$.

(legend continued on next page)

single (potentially noisy) outlier genes. Further, our approach ensures that selected marker genes are expressed in the target cell type, thereby excluding, for example, genes specific to tissue-resident immune cells that might be indicative of inflammation. Using gene expression data from 37 datasets covering 9 podocytopathy models and ~250 samples covering 13 liver disease models (Table S1), we first ranked genes by their *p* values for differential expression in each of these models (always comparing the diseased/experimental condition with the respective control). Those ranks were subsequently averaged across all disease models per cell type to obtain one global gene ranking for podocytes and one for hepatocytes. Next, we applied two additional filters: first, we removed genes that were not expressed in the target cell type (i.e., podocytes or hepatocytes; STAR Methods). Second, we only kept genes with a consistent direction of change (same sign) in at least 75% of all studies. Finally, we chose the top *n* genes as damage marker genes for each of the two cell types. To quantify the damage to an individual cell (or bulk sample), we tested how extreme the expression of PDS/HDS marker genes was in a given cell using AUCell.[11] Genes with positive and negative log fold changes were combined considering the direction of effects (STAR Methods), resulting in an algorithm capable of scoring cellular damage at single-cell resolution (Figures 1B and 1E).

Damage scores robustly characterize cellular states

To establish the robustness of our approach, we first confirmed that varying the number of top genes used for the damage scores had little influence on the outcome. Varying the number of marker genes between 30 and 200 only minimally affected the scores' ability to distinguish (diseased) experimental samples from (healthy) control samples (Figures S1A–S1D). Likewise, randomizing parts of the input data had little influence on the ability of the damage scores to distinguish healthy from diseased samples. Even when randomizing (shuffling) up to 70% of all input studies, the resulting damage scores were still able to distinguish experimental from control samples in disease models that were not used for developing the damage scores (Figures S1E and S1F). The choice of the top *n* genes used to compute the damage scores is, to an extent, arbitrary. We decided to continue all subsequent work with the top 42 marker genes for each of PDS and HDS (Table S2), based on an assessment of the statistical power to separate diseased from healthy samples (Figures S1B and S1D). Next, we confirmed that the PDS and HDS were indeed cell-type specific. By analyzing single-nucleus RNA-seq (snRNA-seq) data obtained from glomeruli and liver, we confirmed that only the targeted cell type showed significant variation in response to damage (Figures S1G and S1H). Furthermore, available glomerular proteomic data[14] confirmed that PDS marker proteins were more abundant in podocytes compared to mesangial and glomerular endothelial cells (Figure S2A).

A central aim of our study was to develop scores that measure cellular damage across disease conditions rather than reflect molecular adaptations in specific disease models. We tested this notion using a ''leave-one-model-out'' cross-validation: first, we removed all samples of one similar group of disease models from the training data; then, we performed the procedure described above to determine damage marker genes on the remaining samples; finally, we tested the resulting damage scores on the left-out disease model samples. In each of the cross-validation rounds, both the PDS and HDS were able to distinguish experimental samples from control samples in the unseen disease models (Figures 1C, 1F, and S2B–S2E). Altogether, these results demonstrated that the damage scores are remarkably robust against diverse variations in the data and that they capture generic molecular changes operating across disease models in a cell-type-specific fashion.

Damage coincides with declining cellular identity

Most marker genes for PDS and HDS were downregulated with increasing damage (PDS: 39/42, HDS: 30/42; Table S2), suggesting a loss of cell-type-specific gene expression. Downregulated PDS markers included genes with established key roles in podocyte biology, such as genes coding for the slit diaphragm (SD)-associated regulators *Magi2*,[15] *Thsd7a*,[16] *Robo2*,[17] and *Ddn*.[18] Further, mutations in the PDS genes *Plce1*,[19] *Wt1*,[20] *Magi2*,[15] and *Ptpro*[21] are known to cause podocytopathies in humans. Other PDS genes with established function in podocytes include the transcription factors (TFs) *Mafb*[22] and *Dach1*,[23] as well as *Sulf1*, *Cldn5*,[24] *Optn*,[25] *Pard3b*,[26] *Enpep*,[27] and *Sema3g*.[28] Overall, PDS genes were enriched for pathways with high relevance to podocyte biology (Table S2; Figure S2F).

Likewise, many HDS marker genes downregulated in the disease condition are involved in hepatocyte-specific functions such as bile acid metabolism (e.g., *Cyp7b1*[29]), detoxification (e.g., *Selenbp2*[30]), and factors secreted into the blood (e.g., *Serpinc1*, i.e., antithrombin and C8b).[31] Further, downregulated HDS marker genes are involved in the maintenance of cellular homeostasis, for example, maintaining genomic stability (e.g., *Mcm10*[32]), response to oxidative stress (e.g., *Mpv17L*[33]), and immunoregulation (e.g., *Ddt*[34]). Other downregulated HDS

(F) Spatial validation of the PDS using kidney Slide-seqV2 spatial transcriptomics data on diabetic BTBR$^{ob/ob}$ and control kidneys (GEO: GSE190094[13]). Representative section of a diabetic mouse kidney is shown. Circles represent glomerular areas and are colored by the mean PDS calculated for podocyte beads (as determined in Marshall et al.[13]) within one glomerulus. The right plot shows PDS measurements normalized for podocyte density after correcting for area variance caused by tissue sectioning and reveals increased podocyte damage corresponding to hyperfiltration in low podocyte density settings such as diabetes or glomerular hypertrophy (see also Figure S3A for density calculation). Dots are colored by genotype, and Spearman correlation (r) and *p* value are provided for each genotype.

(G) Spatial validation of HDS using 10× Visium mouse spatial transcriptomic data.[8,12] Liver tissue section of a mouse with NAFLD. Left: HDS computed for each spot that passed filtering in spatial transcriptomics data. Right: corresponding H&E staining. Indicated areas are enlarged as insets, in which HDS correlates with the degree of lipid accumulation. Black boxes indicate areas with little steatosis and low HDSs, and yellow boxes indicate areas with marked steatosis and high HDSs.

See also Figure S4B for additional data.

CellPress
OPEN ACCESS

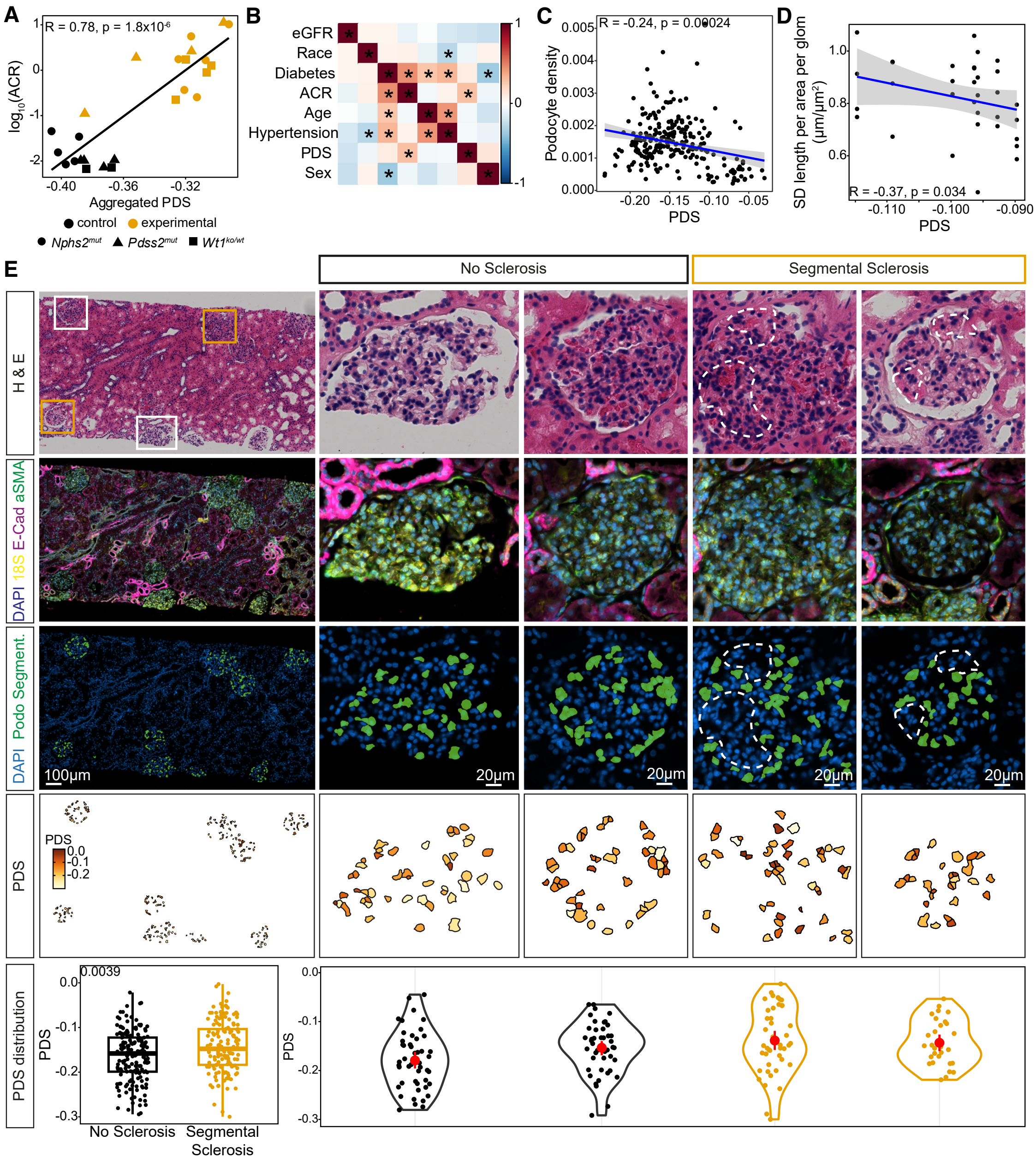

Figure 2. Functional validation of the PDS on mouse and human datasets

(A) Correlation between albuminuria (logarithm of the albumin/creatinine ratio [ACR] in mg/mg), an established clinical measure of podocyte damage, and the PDS for three different murine disease models (*Nphs2*^{mut}, *Pdss2*^{mut}, and *Wt1*$^{ko/wt}$); n = 25. Cells of each snRNA-seq sample were aggregated (pseudo-bulk) and represented as one symbol. The shape and color of symbols represent the study and experimental status of the sample, respectively. The Spearman correlation (r) and p value are provided.

(B) Spearman rank correlation (color scale) between the PDS and clinical attributes of patients with acute kidney injury (AKI) and patients with chronic kidney disease (CKD) as provided in the KPMP kidney tissue atlas (https://atlas.kpmp.org/). Asterisks denote correlations with $p < 0.05$. scRNA-seq (n = 17) and snRNA-seq (n = 37) data from patients with available albuminuria measurements were used. (eGFR, estimated glomerular filtration rate; ACR, urinary albumin/creatinine ratio).

(C) Scatterplot of PDS and podocyte density per glomerulus—calculated as the number of podocytes per glomerular area—in human Xenium spatial transcriptomic data. PDS is calculated per individual podocyte and aggregated per glomerulus. The Spearman correlation coefficient and p value are shown in the image. The blue line indicates linear regression, and the gray area shows a 0.95 confidence interval around the regression line. 238 gloms across 8 patients (each in 2 replicates) were analyzed.

(legend continued on next page)

genes have previously been associated with the progression of liver disease (e.g., *Nudt7*[35] and *Hes6*[36]) and with impaired liver regeneration (e.g., *Lifr*[37] and *Tm7sf2*[38]; Table S2).

Upregulated HDS genes were active in fatty acid uptake (e.g., *Cd36*[39]), suppression of gluconeogenesis (e.g., *Nrg1*[40]), and lipid transport (e.g., *Apoa4*[41]) and were significantly enriched for pathways relevant to cell-adhesion and wound-healing processes, indicating responses to tissue damage (Table S2). Examples include Reticulon 4 (RTN4), which activates TGF-β-mediated fibrosis,[42] and IQGAP1,[43,44] which has already been shown to positively correlate with damage. In line with this evidence, silencing IQGAP1 ameliorates hepatic fibrogenesis[43] and suppresses liver cancer.[44] Taken together, these results suggest that cellular damage in podocytes and hepatocytes proceeds in conjunction with cell identity loss.[45,46]

Lastly, we checked podocyte and liver damage markers for associations with known genome-wide association study (GWAS) variants (see STAR Methods). Eight PDS and ten HDS marker genes were associated with kidney and liver disease-related GWAS traits, respectively (Table S3). A significant enrichment for GWAS variants was detected in the gene bodies of podocyte damage marker genes ($p = 0.031$). This enrichment was enhanced upon inclusion of flanking, non-coding genomic regions ($p = 0.002$, Fisher's exact test). While HDS marker genes were not significantly enriched for GWAS variants (Fisher's exact test $p = 0.1$ for both tests as described above), they showed a trend toward preferentially containing genes associated with liver disease.

PDS and HDS report on pathologic cellular states

To validate the association of the PDS with histologically detectable damage, we used two morphological features. First, we used glomerular size, since glomerular hyperfiltration such as in diabetic kidney disease results in glomerular hypertrophy and consequent podocyte stress, as podocytes are unable to proliferate for compensation.[13,47] Thus, larger glomeruli with lower podocyte densities are indicative of greater podocyte damage. We used spatial transcriptomic data from diabetic and control mouse kidneys to correlate glomerular podocyte density and glomerular PDS.[13] We observed a significant correlation ($p < 1 \times 10^{-4}$ in controls and $p < 1 \times 10^{-10}$ in diabetic mice) between the two measures (Figures 1D and S3A), showing that the PDS captures within-sample variability of cellular damage. Importantly, PDS indicates that glomerular size in wild-type mice is associated with podocyte stress presumably attributable to increased filtration load. Second, we used the SD length, a proxy for damage to the podocyte filtration barrier, with shorter length indicating greater damage.[48] We used stimulated emission depletion (STED) imaging of SDs to assess the protein expression of three PDS signature members localizing to the SD, namely THSD7A,[16] CLDN5,[49] and EPHB1.[50] This confirmed decreased (EPHB1 and THSD7A) or redistributed signals (CLDN5) with shortened SDs in podocytopathy models as compared to controls, which is consistent with greater PDS values indicating greater podocyte damage (Figures S3B and S3C). Furthermore, SD length quantification in three mouse models revealed a significant negative correlation between SD length and the PDS, i.e., models with larger PDS signals exhibited elevated morphological signs of damage (Figure S3D).

In chronic liver disease, a unifying, histologically detectable and locally heterogeneous feature of many disease conditions, including viral hepatitis, MASLD, and MASH, is the ectopic formation and accumulation of lipid droplets in hepatocytes (also called liver steatosis).[51] Therefore, we assessed the local accumulation of lipid droplets in hepatocytes as a proxy for accumulated hepatocyte damage. Using spatial transcriptomic data from a cohort of NAFLD mice and healthy controls,[8,12] we confirmed that the HDS captured within-sample variation of liver tissue damage. First, we observed significantly lower average HDSs in healthy control liver sections when compared to liver sections from NAFLD mice (Figure S4A). Second, within the same tissue section from NAFLD mice, areas with more abundant fat droplets had greater HDSs than less lipid-laden areas (Figures 1G and S4B). Taken together, these results confirm that the PDS and HDS are molecular measures of histologically detectable damage.

To test if the PDS also reflects functional decline of podocytes, we correlated the PDS with albuminuria measurements in mice. The albumin/creatinine ratio (ACR) quantifies excess albumin in the urine and is a quantitative and established measure of podocyte damage.[48,52] We analyzed 12 mouse studies with combined bulk glomerular transcriptomic and ACR measurements and found a significant positive correlation (Spearman = 0.45, $p = 0.017$) between PDS and albuminuria (Figure S3E). The correlation was even stronger (Spearman = 0.78, $p = 1.8 \times 10^{-6}$) for snRNA-seq data generated within this study (Figure 2A; Table S4). Here, we aggregated PDSs across podocytes within each sample and then correlated these pseudo-bulk PDSs with albuminuria (Figure 2A). Taken together, these results indicate that the PDS quantitatively reflects functional decline of podocytes. In some contexts, the PDS may even complement albuminuria as a surrogate measure of podocyte damage because,

(D) Significant negative correlation between slit diaphragm (SD) length as measured by confocal microscopy and PDS measured on identical glomeruli in human Xenium spatial transcriptomics (glomeruli $n = 7$, each measured in 3–7 technical replicates). The blue line indicates regression, and the gray area indicates a 0.95 confidence interval around the regression line. The Spearman correlation coefficient and p-value are provided.

(E) Human Xenium spatial transcriptomic data from a 71-year-old female suffering from FSGS. Top row: H&E histology including overview and high-magnification images of select glomeruli (see boxes in overview). The dashed white lines in high-magnification images indicate areas of potential segmental sclerosis. Second row: immunofluorescence staining signal used for cell segmentation, including nuclei (DAPI, blue), 18S-RNA (yellow), E-cadherin/CD45/ATP1A1 (all magenta), and Vimentin/alpha-smooth muscle actin (SMA; all green). Third row: location of segmented podocytes together with nuclei. The dashed lines indicate glomerular areas devoid of podocytes, indicative of segmental sclerosis. Images in the second and third rows were generated with Xenium Explorer 3. Scale bars are indicated. Fourth row: PDS values (color scale) per single segmented podocyte. Podocytes with insufficient transcriptomic signal depth to calculate PDS were omitted. Bottom row: boxplot shows PDSs of individual podocytes, grouped by glomerular morphology (no sclerosis vs. segmental sclerosis), and the p value of the two-sided Wilcoxon test is shown in the top left; $n = 243$. Violin plots show distribution of single podocyte PDS values (dots) within the glomerulus shown in high-magnification images. Red dot: median; red line: interquartile range.

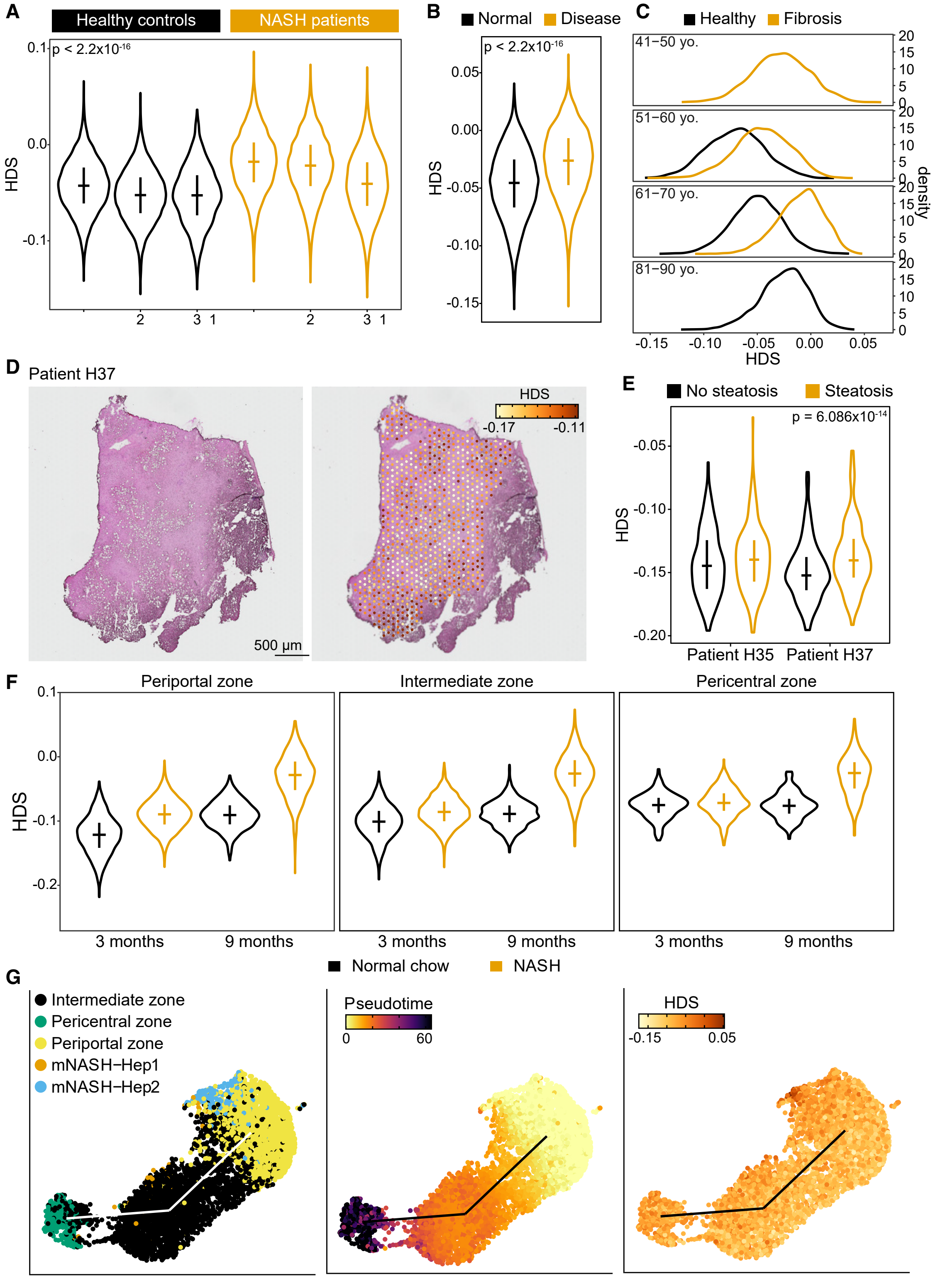

Figure 3. Functional validation of the HDS on mouse and human datasets

(A) Violin plots for HDS distribution in hepatocyte snRNA-seq data of human healthy subjects and patients with NASH (GEO: GSE189600[59]). HDS marker genes were mapped to human ortholog genes. Age and sex of healthy donors are in the same order as shown in the figure: 47-year-old female, 50-year-old male, and 50-year-old male. Age and sex of patients with NASH in the same order as shown in the figure: 49-year-old male, 64-year-old female, and 31-year-old male.

(legend continued on next page)

unlike albuminuria, it is measurable outside of a functioning organism and even in single cells.

As validation of both PDS and HDS showed remarkably robust results at the transcriptomic level, we further tested whether the damage-reporting capacity of our markers could be expanded to the protein level. To this end, we calculated PDSs and HDSs on proteomic data from murine models of podocyte and liver disease, respectively.[14,48,53–55] Without exception, PDSs and HDSs separated diseased from control samples (Figures S3F and S4C). Future application of our scores may hence be extended to proteomics studies.

PDS and HDS are predictive in human data

PDS and HDS were developed using exclusively murine transcriptomic data and revealed very robust gene signatures. Hence, we next asked whether the signature was conserved in and therefore applicable to human data as well. To this end, where available, we identified human orthologs for all murine genes constituting the signatures (see STAR Methods). For the PDS, we reanalyzed publicly available human data from the Kidney Precision Medicine Project (KPMP),[2] including patient clinical characteristics, histology, sc/snRNA-seq data, and spatial transcriptomics. To test the clinical predictive value of the PDS in humans, we correlated human ACR with the PDS and other established clinical predictors of albuminuria such as age, sex, and diabetic status across the KPMP cohort. Only diabetic status and the PDS were significantly correlated with ACR (Figures 2B and S5A), thereby replicating the findings obtained in mice. Within the KPMP Visium spatial transcriptomic dataset, we identified a single biopsy core clearly showing an focal segmental glomerulosclerosis (FSGS) phenotype. Within this sample, PDS correlated with histologic features of FSGS, distinguishing healthy from sclerosed glomeruli and even resolving segmental glomerulosclerosis within a single glomerulus (Figures S5B and S5C).

Motivated by the success with this existing human data, we generated new spatial human transcriptomics data using the Xenium Prime 5K platform, which provides substantially improved spatial resolution compared to the Visium system (spots 55 μm in diameter spaced at 100 μm vs. <50 nm precision for single-transcript localization). Using a customized gene panel that included PDS marker genes, we generated such data for eight patients with minimal change disease or FSGS[56] and combined clinical data with histology, spatial transcriptomics, and confocal microscopy (to measure SD length per glomerulus). First, this analysis reproduced the correlation between podocyte density, as a measure of hyperfiltration stress, and PDS in humans (Figure 2C). Furthermore, using the SD length data and PDSs obtained in identical single glomeruli, we confirmed a significant negative correlation between SD length and PDS (Figure 2D). Finally, leveraging the subcellular resolution and built-in image segmentation information in Xenium spatial transcriptomic data, we analyzed the distribution of single-podocyte PDS values in glomeruli featuring segmental sclerosis. This analysis revealed that PDS values were elevated in sclerosed glomeruli compared to glomeruli without sclerosis (Figure 2E).

In addition, when we computed the PDSs from scRNA-seq data of podocytes recovered from human urine within the NEPTUNE study,[57] PDS correlated with the urine protein/creatinine ratios (UPCRs) at the time of urine collection (Figure S5D). Finally, in microarray, bulk RNA-seq, and snRNA-seq data from human kidney biopsies, the PDS distinguished healthy control subjects from patients suffering from primary FSGS, IgA nephropathy, and CKD, respectively (Figure S5E).[2,3,58] To conclude, a human ortholog-based PDS was readily capable of discriminating functional and morphological features of human podocytopathies.

In a parallel approach to the HDS, using human orthologs of the marker genes, we calculated the HDS for hepatocytes from snRNA-seq human liver samples from two datasets including non-alcoholic steatohepatitis (NASH) and liver fibrosis

Horizontal lines show the median of the distribution, and vertical lines represent the IQR. The p value of the one-sided Wilcoxon rank test (NASH vs. healthy) is shown.

(B) Distribution of HDS in hepatocyte snRNA-seq data from healthy subjects ($n = 3$) and patients ($n = 3$) suffering from liver fibrosis (data from GEO: GSE210077[60]). Violin plots for HDS measurements pooled by health status ($n = 3$). HDS distribution is shifted to higher values in fibrotic individuals. The one-sided Wilcoxon rank-sum test statistic is shown. Horizontal lines show the median of the distribution, and vertical lines represent the IQR.

(C) Same data as in (B). HDS increases with age in healthy human subjects and depending on disease status. Individuals are shown by increasing age range (see the top of the plots) from top to bottom. Each curve represents data from one individual colored by disease state (see legend).

(D) HDS reflects within-sample variance of human liver steatosis. HDS calculation on 10× Visium human spatial transcriptomic data from the Liver Cell Atlas (GEO: GSE192742[8,12]). Only subjects with liver steatosis were analyzed. Left: H&E staining of liver sample; right: HDS per Visium spot overlay. Spots representing hepatocytes were manually annotated. The color scale represents the 10% and 90% quantiles of the HDS distribution across all samples.

(E) HDS separates steatotic from non-steatotic areas within the same human liver. 10× Visium spots of human steatotic livers as shown in (D) were manually subclassified into steatotic and non-steatotic regions based on the H&E staining. Violin plots show HDS distribution in regions classified as non-steatotic (black) vs. steatotic (orange), including the sample from the patient shown in (D) (see also Figure S6A for additional data). Horizontal lines show the median of the distribution, and vertical lines represent the IQR. The p value of the one-sided Wilcoxon rank-sum test on pooled HDS values for steatotic vs. non-steatotic regions is shown at the top of the plot.

(F) HDS in relationship to liver zonation in mice; hepatocyte snRNA-seq data taken from GEO: GSE189600.[59] Male mice were fed with standard or NASH-promoting diet at 4 weeks of age for 3 or 9 months ($n = 1$). HDS distributions for cells from the control (black) and NASH-inducing (orange) diets are shown. Horizontal lines show the median of the distribution, and vertical lines represent the IQR. Statistical comparison was not appropriate due to $n = 1$ per group. Hepatocyte zone annotation was provided by the authors of the dataset (GEO: GSE189600[59]).

(G) HDS vs. pseudo-time trajectories (PTs); same data as in (F). UMAPs show hepatocytes of a 3-month-old mouse fed a NASH-inducing diet. Each dot represents a hepatocyte colored by hepatocyte zone (left), PT (middle), and HDS (right). Hepatocyte zone annotation is from the original publication.[59] mNASH-Hep1/2: classification from the original publication corresponding to damaged hepatocytes whose zone of origin could not be determined according to the authors. PT correlates with zonation, while the HDS is independent of liver zonation. See also Figure S6B for additional data.

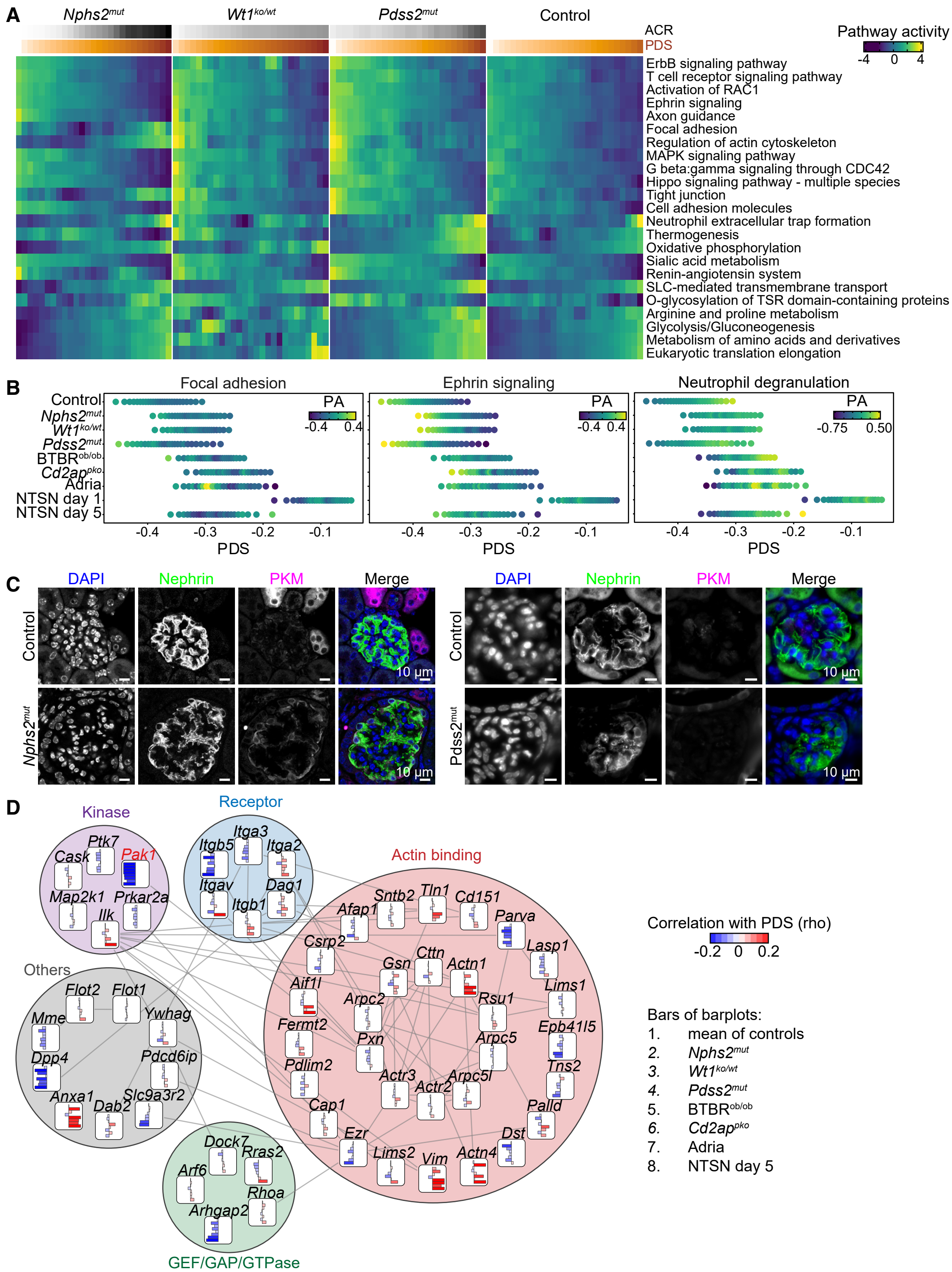

Figure 4. Application of PDS to FSGS datasets reveals model-specific pathway fingerprints

(A) PDS pathway fingerprints for snRNA-seq data from *Nphs2*^{mut}, *Wt1*$^{ko/wt}$, and *Pdss2*^{mut} models of FSGS. "Control" is the average of control samples from all three studies. Nuclei were sorted by increasing PDS (color bar on top). Pathway activities (PAs) for KEGG and Reactome pathways were calculated using AUCell (see STAR Methods) and smoothed using a sliding window of width twice the step size, both adjusted to produce 27 bins in each study. Color shows PAs centered and scaled per pathway. The activity of pathways shown is significantly correlated with PDS in at least one of the three studies (Spearman rank correlation;

(legend continued on next page)

phenotypes.[59,60] The HDS readily differentiated three healthy control subjects from three patients suffering from NASH (Figure 3A), as well as three healthy controls from three patients suffering from liver fibrosis (Figure 3B).[60] Interestingly, the healthy controls represented a wide range of ages, which was reflected by higher HDS levels in older healthy patients (Figure 3C). To test if an increase in HDS was also associated with morphological tissue changes in humans, we analyzed spatial transcriptomic data (10× Visium) from liver sections of two patients with steatosis.[8] Based on histology, spots in the spatial data were manually categorized as areas with or without steatosis (Figure S6A). Upon calculating the HDS, values in tissue areas with steatosis were significantly higher than in tissue areas without steatosis (Figures 3D, 3E, and S6A). Taken together, our findings indicate that conserved cell-type-specific damage scores can be developed from murine transcriptomic data that are subsequently amenable to translational application in humans.

Comparison of HDS and pseudo-time trajectory inference

Pseudo-time trajectories have been proposed as an alternative approach for the computational alignment of single-cell and single-nucleus data along developmental, spatial, and temporal gradients.[61] Compared to the single-cell damage scores presented here, pseudo-time trajectories have potentially two disadvantages: first, they are not readily applicable across different studies, disease models, and species, as pseudo-time trajectories are commonly inferred per dataset or per study after appropriate data integration. Second, pseudo-time trajectories may have a disadvantage if gradients in single-cell data are dominated by signals other than cellular damage. An example is liver zonation: gene expression in hepatocytes is strongly determined by their relative position with respect to periportal and central veins, a signal that potentially "shades" expression changes caused by cellular damage. To test this notion, we compared two established methods for pseudo-time trajectory inference (TSCAN[62] and Monocle3[63]) to the HDS. Applying both algorithms to hepatocyte snRNA-seq data from mice suffering from NASH and controls, for which zonation information was available at the individual hepatocyte level,[59] we confirmed that the HDS separated undamaged from damaged hepatocytes independent of their zone of origin (Figures 3F and S6B), i.e., the HDS is independent of liver zonation. In contrast, the pseudo-time trajectories were dominated by gene expression patterns driven by hepatocyte zonation (Figures 3G, S6C, and S6D). Thus, the damage scores presented here provide information that is distinct from and complementary to pseudo-time trajectories.

Pathway signatures reveal altered metabolism in damaged podocytes

Having validated our scores, we next sought to leverage their potential in addressing unanswered questions in kidney and liver biology. A key feature of the single-cell damage scores developed here is that they facilitate the analysis of progressive molecular changes associated with increasing cellular damage in disease models and even under unperturbed control conditions. To implement this notion, we developed a scheme that first sorts cells *in silico* along a damage trajectory from low to high damage and next identifies regulatory pathways and cellular processes (subsequently collectively called "pathways") whose activity changes progressively along this axis. Cells with similar damage scores were binned (grouped) to reduce noise, and pathway activities per bin were determined using AUCell (see STAR Methods) for all KEGG and Reactome pathways. We note that this approach does not compare gene expression variation between samples, which runs the risk of being affected by batch effects and other inter-individual variation. Instead, this scheme enables the analysis of disease progression within a single sample. Importantly, cells from control samples also displayed a spectrum of damage. Even though the average damage was lower in control samples than in experimental conditions, such samples always contained a small fraction of cells with relatively high damage scores (Figures 1B, 1E, and 4A). This observation motivated us to sort cells by damage in the control samples and identify pathway activity changes along this gradient of otherwise unperturbed cells. Thereby, it became possible to identify pathway activity changes that consistently shifted in the same direction between experimental and control conditions, which can be considered general components of cellular damage and less likely attributable to a specific disease model or trigger. This approach allowed for addressing a key conundrum in the diagnostic approach to glomerular kidney disease, i.e., the contrast between a uniform histologic phenotype of podocyte damage—FSGS—and the multiple triggers, diseases, and molecular pathways resulting in this phenotype. First, this poses a clinical challenge, as histology is often insufficient to provide a conclusive diagnosis. Second, it remained challenging to differentiate common from trigger-specific cellular events leading to the FSGS phenotype.

adjusted $p < 0.05$). Rows are clustered by pathway similarity. Color gradients on top show the average albumin/creatinine ratio (ACR) and the average PDS per bin.

(B) PAs for selected pathways calculated on sn/scRNA-seq data of cumulated control samples and various disease models. Cells/nuclei were grouped in evenly sized bins of 30 cells/nuclei with similar PDSs (*x* axis). Color indicates PA centered and scaled per pathway. 'Control' corresponds to the average PAs across control cells of all models. The range of PDSs per model reflects different damage levels between models and cumulated controls.

(C) Differential regulation of energy metabolism. Immunofluorescence staining of control and experimental kidney tissue from either *Nphs2*^{mut} 8-week-old (left, n = 3) or *Pdss2*^{mut} 21-week-old (right, n = 3) with DAPI (blue), nephrin (green), and PKM (magenta) is shown. Scale bars: 10 μm. Mice of both models reflect similar damage levels as by ACR and SD length (see also Figures 2A and S3C). PKM expression is upregulated in podocytes in *Nphs2*^{mut} mice but not in *Pdss2*^{mut} mice.

(D) Integration of the *in vivo* podocyte adhesome protein-protein interaction network with PDS analysis.[64] Genes are grouped by function, and edges indicate protein-protein interactions of the gene products. Bars within each node show the Spearman rank correlation (rho; see color scale) between PDS and the expression level of the gene per disease model (see legend on the right). Red and blue colors depict positive and negative correlations, respectively. The saturation of the color and the length of the bar reflect the strength of the correlation. The marked upregulation of *Actn1*, *Anxa1*, and *Vim* exclusively in studies that use scRNA-seq with prior glomerular digestion is likely a technical artifact. *Pak1* (marked in red) is a PDS marker gene.

To address this challenge, we obtained published glomerular scRNA-seq data for four podocytopathy models[65] and generated snRNA-seq data for three additional models (Figure S7; Table S4) to reflect a wide range of podocyte damage triggers relevant to important aspects of podocyte biology and clinical settings. Together, these models include mutations in SD proteins (*Nphs2^{mut}* and *Cd2ap^{pko}*), metabolic and oxidative stress (*Pdss2^{mut}* and doxorubicin), antibody-mediated glomerular inflammation (NTN), diabetes (BTBR$^{ob/ob}$), and mutation of a key podocyte TF (*Wt1$^{ko/wt}$*).

To test whether damage scores were quantitatively comparable across disease models and studies, we compared damage scores between control samples from different studies. If the scores are consistent, we would expect (1) control samples across studies to show similar damage distributions and (2) experimental samples to differ according to the degree of induced damage. This was indeed the case (Figure S8A). Pairwise Kolmogorov-Smirnov distances between PDS distributions were on average lower between controls than between experimental PDS distributions (two-sample Wilcoxon test, $p = 2.5 \times 10^{-11}$), arguing in favor of a common PDS baseline in control samples. A similar behavior was noted for the HDS (two-sample Wilcoxon test, $p = 2{,}7 \times 10^{-4}$; Figure S8B), supporting our notion that damage scores are robust and quantitatively comparable across studies. Interestingly, this approach allowed for identifying a small shift in average PDS baseline between scRNA-seq and snRNA-seq studies, suggesting that the additional damage exerted by tissue digestion for scRNA-seq was captured by the PDS (Figure S8A).

Next, we quantified pathway activity changes along the PDS axis as outlined above and focused on pathways that were either already known to be relevant to podocyte biology and/or showed highly dynamic changes with increasing damage. This way, we established "pathway fingerprints" that characterize the regulatory, cell structural, and metabolic changes happening during progressively increasing damage in a specific disease model (Figures 4A, 4B, and S8C). These pathway fingerprints provide a rich source of information, allowing us to discern common and unique patterns of pathway changes across disease models. First, pathways expected to show model-specific behavior, due to the nature of various damage triggers, distinguished fingerprints from each other. For example, mutating the mitochondrial regulator *Pdss2* was reflected by specific activity changes of the closely associated mitochondrial pathway "thermogenesis." Similarly, in a complex with NPHS2 (podocin), tight junction proteins are part of the SD protein complex,[66,67] and the tight junction pathway within the fingerprint of the podocin mutation model (*Nphs2^{mut}*) expectedly distinguished this model from others (Figure 4A). In addition to highlighting model-specific pathways, fingerprints also established pathways commonly modulated with podocyte injury and independent of the trigger. For example, hippo signaling, which was initially found downstream of WT1 transcriptional regulatory function in podocytes,[20] has now been shown to be unequivocally decreased upon podocyte damage in all models. Key pathways in podocyte biology related to the actin cytoskeleton, cell adhesions, or the renin-angiotensin system were also commonly modulated (Figure 4A). In addition to cell structural and regulatory changes, we observed common metabolic changes in podocytes associated with increasing damage. Energy metabolism (glycolysis and oxidative phosphorylation) and protein production (translation) were consistently upregulated with increasing damage across models, suggesting that podocytes are majorly reorganizing their proteome while changing shape and coping with increasing stress. In the clinical context, albuminuria is used as a measure to "align" disease status between patients. Here, we test if the PDS could serve a similar purpose for "aligning" cellular damage states across disease models. Therefore, we integrated damage levels as indicated by the PDS with pathway activity changes across disease models (Figure 4B). This approach allows for the alignment and direct comparison of multiple disease models, samples, or patient biopsies with respect to damage levels and associated signaling pathway behavior. Here, ephrin signaling, which was recently identified as a pathway relevant to the progression of kidney disease,[68] was consistently downregulated upon podocyte damage. To experimentally test the relevance of ephrin signaling in the context of podocyte damage, we performed fluorescent labeling and STED imaging of Ephrin type-B receptor 1 (Eph B1), which is one of the PDS marker genes (Figure S3B). This analysis confirmed the localization of Eph B1 to the SD and showed substantial depletion in damaged podocytes.

To experimentally validate our approach and the alignment of independent datasets based on the PDS, we confirmed the overactivation of the tight junction pathway in *Nphs2^{mut}* compared to *Wt1$^{ko/wt}$* mice. We carried out immunofluorescence staining followed by super-resolution microscopy for the SD marker nephrin (NPHS1) and the tight junction protein claudin-5 in both disease models (Figure S9A). Claudin-5 has been shown to aggregate in the SD upon podocyte damage,[49] and plays a protective role in diabetic nephropathy.[24] Our approach allowed for the parallel quantification of both podocyte damage based on SD length and tight junction activation based on the degree of Claudin-5 aggregation. Hence, we were able to obtain quantitative measures of pathway activation and podocyte damage independent of the PDS. By correlating SD length with Claudin-5 aggregation, we indeed confirmed that in *Nphs2^{mut}* mice, tight junction activation was more pronounced at similar damage levels when compared to *Wt1$^{ko/wt}$* mice, as evidenced by a stronger negative correlation in *Nphs2^{mut}* mice (Figure S9B).

In a second experimental confirmation of our pathway activity analysis, we addressed podocyte energy metabolism represented by the pathways thermogenesis, oxidative phosphorylation, and glycolysis/gluconeogenesis in our fingerprinting analysis (Figure 4A). While the activity of these pathways was generally upregulated with increasing podocyte damage in all models, we noticed that at high damage levels, a pronounced overactivation was evident in *Pdss2^{mut}* mice compared with *Nphs2^{mut}* mice (Figures 4A and 4B). For both models, consistent SD length and albuminuria measurements were available (Figures 2A, S3C, and S3D), allowing for gauging and aligning damage levels independent of the PDS. To evaluate key regulators of energy metabolism in podocytes, we stained kidneys for phosphorylated S6-kinase, which acts downstream of mTORC1 in the mTOR pathway and is a major regulator of podocyte energy metabolism contributing to FSGS pathogenesis.[69,70]

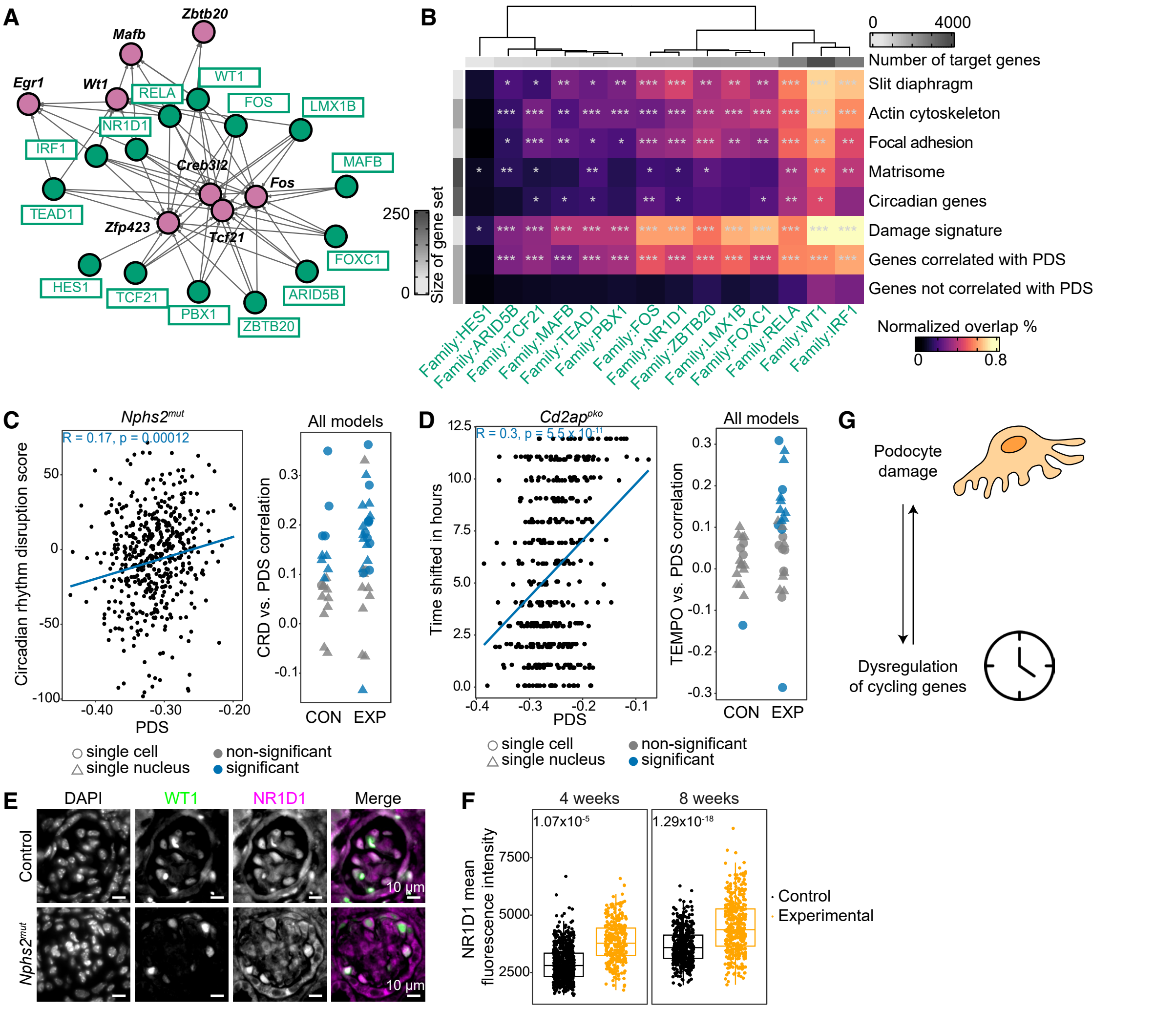

Figure 5. PDS reveals circadian dysregulation as a central mechanism in FSGS

(A) Podocyte transcriptional regulatory network (TRN), limited to transcription factor (TF) genes correlating with PDS in multiple studies (magenta nodes) and TF family regulons (green nodes). Edges indicate regulation of the TF gene by the respective TF family.

(B) TRN analysis reveals the circadian core TF NR1D1 (Rev-Erb alpha) as a key regulator of podocyte genes. Heatmap of TF target gene overlap (color scale) with gene sets is shown on the right. Color intensity shows the fraction of genes in gene sets (rows) regulated by the respective TF families (columns). Significance of the overlap was calculated with Fisher's exact test, false discovery rate (FDR) adjusted, and depicted on the heatmap with asterisks: ***$q < 0.001$, **$q < 0.01$, and *$q < 0.1$. Row and column annotation bars (gray scale) reflect the size of gene sets or target genes, respectively. Bottom row: TF target enrichment for 100 randomly selected genes that are expressed in podocytes but do not correlate with the PDS as a negative control.

(C) Increased podocyte damage correlates with increased circadian disruption. Left: relationship of circadian rhythm disruption (CRD) score[85] and PDS. Each dot represents one nucleus from snRNA-seq data of podocytes isolated from one individual *Nphs2*^{mut} mouse ($n = 357$). The regression line and parameters for the Spearman rank correlation are shown. Right: the *y* axis depicts the Spearman rank correlation coefficients (calculation as exemplified for one individual mouse on the left) for all individual control (CON) and experimental (EXP) samples of seven different mouse models of podocyte damage (*Nphs2*^{mut}, *Pdss2*^{mut}, *Wt1*$^{ko/wt}$, BTBR$^{ob/ob}$, NTN day 5, *Cd2ap*^{pko}, and Adria). Color distinguishes whether the CRD-to-PDS correlation is significant ($p < 0.05$). See the legend in (D) for the meaning of symbols.

(D) Left: relationship of absolute deviation of estimated circadian time from the reference sample time and the PDS. The circadian time of each cell was estimated by the TEMPO algorithm ($n = 323$), using *Clock* as the reference gene. Each dot represents one cell from scRNA-seq data of podocytes isolated from one individual *Cd2ap*^{pko} mouse. The regression line and parameters for Spearman rank correlation are shown. Right: Spearman rank correlation coefficients (calculation as exemplified for one individual mouse on the left) for all individual CON and EXP samples of seven different mouse models of podocyte damage (*Nphs2*^{mut}, *Pdss2*^{mut}, *Wt1*$^{ko/wt}$, BTBR$^{ob/ob}$, NTN day 5, *Cd2ap*^{pko}, and Adria). Color distinguishes whether the TEMPO-to-PDS correlation is significant ($p < 0.05$).

(E) Representative immunofluorescence staining of kidney tissue from 8-week-old *Nphs2*^{mut} and CON mice ($n = 3$) for DAPI, WT1 (green), and NR1D1 (magenta). Scale bars: 10 μm. NR1D1 nuclear localization is disrupted in FSGS glomeruli.

(F) Quantification of NR1D1 mean fluorescence intensity in podocyte nuclei for CON and EXP *Nphs2*^{mut} mice aged 4 weeks ($n = 20$ technical replicates from 4 biological CON animals, $n = 20$ technical replicates from 2 biological EXP animals) and 8 weeks ($n = 20$ technical replicates from 3 biological CON animals, $n = 20$

(legend continued on next page)

Supporting our fingerprinting analysis, we saw differential activation of S6-kinase in podocytes between *Nphs2^{mut}* and *Pdss2^{mut}* mice at similar damage levels (Figure S9C). Podocytes heavily rely on anaerobic glycolysis as an energy source.[69] A key rate-limiting enzyme controlling the balance between glycolysis, (anaerobic) lactate production and (aerobic) oxidative phosphorylation is pyruvate kinase (PKM), which in turn is induced in podocytes by S6-kinase[71] and is a protective factor in diabetic nephropathy.[72,73] PKM function is controlled by two splice isoforms of the *Pkm* gene: the *Pkm1* isoform dominates in tissues with high demand for oxidative phosphorylation (muscle and brain), while the *Pkm2* isoform balances metabolism between anaerobic and aerobic pathways in most tissues, including podocytes (Figure S9D).[72,73] First, we confirmed that *Pkm2* is by far the predominant isoform in podocytes (Figure S9E) and showed that *Pkm* expression was significantly higher in *Nphs2^{mut}*-derived compared to *Pdss2^{mut}*-derived podocytes (Figure S9F). Immunofluorescent staining for PKM in *Nphs2^{mut}* and *Pdss2^{mut}* glomeruli further confirmed the specific upregulation of this key energy metabolism regulatory factor in *Nphs2^{mut}* podocytes at the protein level (Figure 4C). In summary, corresponding to our pathway fingerprinting analysis and assays of two regulators of energy metabolism, we verified the differential activation of energy metabolism pathways that depends on damage triggers and damage levels. This supports the applicability of the PDS in investigating differential molecular changes across podocyte damage models, aligned at similar damage levels.

In summary, pathway fingerprints and damage trajectories identified trigger-specific as well as commonly regulated pathways upon podocyte damage, rendering the PDS suitable as a molecular diagnostic approach for FSGS to resolve its molecular etiology.

Identifying generic druggable targets with the PDS

A curious result of our pathway fingerprint analysis was the aberrant and model-specific behavior of the pathways "focal adhesion" and "regulation of actin cytoskeleton" in the *Nphs2^{mut}* model compared with all other models (Figure 4A). Focal adhesions attach podocytes to the glomerular basement membrane (GBM), making them a crucial organelle for the GBM detachment of podocytes during FSGS.[66,67] Focal adhesions and the SD are further tightly connected to the actin cytoskeleton, which is commonly modulated and rearranged upon podocyte damage.[74] A key morphological feature associated with this rearrangement is foot process effacement. We therefore utilized the PDS to gain deeper insight into the reorganization of the "podocyte adhesome" upon damage. We correlated the expression of members of the podocyte adhesome with the PDS in each of the disease models and control samples (Figure 4D).[75] Selected members of this network were distinctly regulated in the *Nphs2^{mut}* model compared to all other models. For example, two genes encoding actin-binding proteins, dystonin (*Dst*) and palladin (*Palld*), exhibited strong negative correlations with the PDS in the *Nphs2^{mut}* model, whereas in other disease models, they were uncorrelated or even oppositely correlated with the PDS (Figure 4D). These examples underline the importance of distinguishing model-specific changes that potentially act upstream of podocyte damage from alterations that are consistent across models. Therefore, we next identified adhesome components that were consistently downregulated with increasing damage irrespective of the damage trigger. These components included the actin-binding molecules *Parva* and *Epb41l5*, the extracellular matrix receptors integrin beta-5 (*Itgb5*) and *Itga3*, the cell surface peptidase dipeptidyl peptidase-4 (*Dpp4*), and the kinase *Pak1* (RAC1-activated kinase 1). All of these components had previously been identified as relevant to podocyte focal adhesion function.[64,75–77] *Parva* and *Epb41l5* in particular have been shown to prevent podocyte detachment from the GBM in FSGS.[64,76] *Pak1* is a key regulator of focal adhesion and actin cytoskeleton networks and has been shown to be involved in the earliest events of FSGS in a CTCF deletion model.[78] Further, our work surprisingly reveals that downregulation of the cell-surface peptidase *Dpp4* is a hallmark of increasing podocyte damage across disease models, including diabetes. *Dpp4* has been reported to be upregulated in diabetes in several tissues and cell types and to contribute to decreased insulin sensitivity. In podocytes, DPP4 physically interacts with the focal adhesion protein ITGB1,[79] and DPP4 inhibitors decrease albuminuria in mouse models of podocyte damage.[80] These drugs are a mainstay in the treatment of type 2 diabetes mellitus, where their use was associated with beneficial albuminuria outcomes in several clinical trials.[81–84] Taken together, the surprising downregulation of *Dpp4* in seven independent disease models can be interpreted as a repair response amenable to preventative treatment. Thus, our integrative adhesome and PDS network analysis revealed promising targets for the prevention of podocyte GBM detachment in podocytopathies.

Regulators of podocyte gene expression drive cross-model correlates of the PDS

To better understand the regulatory programs driving gene expression changes relevant to the podocyte damage response, we constructed a podocyte-specific transcription regulatory network (TRN) (Figure 5A). To derive this TRN, we acquired podocyte-specific assay for transposase-accessible chromatin using sequencing (ATAC-seq) data by flow cytometry sorting of podocyte nuclei from a nuclear GFP reporter mouse (STAR Methods; Figure S10A). Next, we performed footprinting and TF motif scanning within accessible chromatin regions, using DNA-binding motifs of 110 TFs expressed in podocytes. Finally, we grouped TFs with very similar DNA-binding motifs into 14 TF families, as their binding signatures were difficult to distinguish without further information (Figure S10B). We combined

technical replicates from 3 biological EXP animals). Podocyte nuclei were identified by first segmenting by DAPI-positive and then by WT1-positive fluorescent signals prior to the quantification of NR1D1 fluorescence intensity. *p* values for the F-test are shown. The boxplots show first (Q1), second (Q2), and third (Q3) quartiles of distribution. Whiskers represent Q1 − 1.5× interquartile range (IQR) and Q3 + 1.5× IQR.
(G) Schematic of association between damage and dysregulation of circadian genes in podocytes.

predicted target genes of all motifs within each cluster to form a TF regulon and named each regulon after a representative TF (Figure S10C). This established a network between 14 TF families and 6,360 target genes expressed in podocytes that will serve as a critical resource for future research into podocyte biology.

To systematically identify target genes of these regulons that were involved in FSGS progression across models, we computed how often a gene was correlated with the PDS across snRNA-seq studies of FSGS in both experimental and control conditions. Note that this detection of commonly perturbed genes in experimental and control conditions cannot be achieved with traditional differential expression analysis and necessarily requires a within-sample trajectory analysis.[86] Based on these results, we selected 152 genes that were significantly correlated (Table S5, *t* test, adjusted $p < 0.1$) with the PDS in seven or more tests. Forty of those genes were already members of the PDS marker gene set.

This set of 152 genes contained eight podocyte-expressed TFs, i.e., of the 110 TFs used to create the TRN. Thus, we used the TRN to inspect the regulatory relationships among these eight TFs tightly associated with podocyte damage (Figure 5A). These TF genes included the core podocyte TFs *Wt1*, *Mafb*, and *Tcf21*, TFs known to respond to external damage cues (*Egr1* and *Fos*), and TFs with yet uncharacterized functions in podocytes (*Zbtb20*, *Zfp423*, and *Creb3l2*). The regulators established two groups: one group controlling all eight PDS-associated TFs and another set controlling only four of them (*Egr1*, *Wt1*, *Mafb*, and *Zbtb20*). Consistent with previous studies, we identified the regulons of the WT1, LMX1B, and TEAD1 families as controlling the majority of TF genes correlating with the PDS.[20] Interestingly, in addition to these established podocyte regulons, the IRF1, nuclear receptor subfamily 1 group D (NR1D1), and RELA/p65 families were similarly prominent, indicating that inflammatory signals and processes dependent on nuclear receptor signaling such as the circadian clock may be relevant to podocyte damage (Figure 5A).[86]

Podocyte damage disrupts the circadian clock

Next, we utilized the podocyte TRN to identify potential regulators of genes that were likely involved in podocyte damage either because their own expression correlated with the PDS (i.e., the 152 genes from above) or because they are involved in cellular functions that are known to be relevant for podocytopathies, such as the podocyte adhesome,[75] matrisome,[87] actin cytoskeleton,[88] and SD.[67] Consistent with earlier work, we found that WT1 was predicted to bind regulatory elements of the majority of genes involved in these key podocyte structures as well as of genes correlating with the PDS (Figure 5B). Next to WT1, the TF families containing RELA/p65 and IRF1 (both of which are reacting to inflammatory stress) were prominent regulators of these damage-associated gene sets. Furthermore, all gene sets were co-regulated by a combination of cell-type-specific podocyte TFs (e.g., WT1, LMX1B, FOXC1, MAFB, and TCF21) and generic TFs (e.g., TEAD1 and AP-1 complex/FOS; Figure S10D). The fact that some of the generic regulators were themselves correlated with the PDS (e.g., *Fos*) suggests that the transcriptional response to podocyte damage is coordinated via an intricate network of generic and cell-type-specific TFs (Figure S10C. A striking finding was that NR1D1 (Rev-Erb alpha) was predicted to regulate a wide range of genes that we found to be correlated with the PDS (Figure 5B). Rev-Erb alpha is one of the core TFs that regulate feedback loops within the intracellular circadian clock[89] and is instrumental in establishing circadian gene expression. Recent studies have established that many key podocyte genes are expressed in a circadian fashion and that the disruption of the podocyte circadian clock results in podocyte dysfunction.[90–92] Based on the tight link between NR1D1 and many of the PDS-associated processes across disease models, we speculated that the reverse may also be true, i.e., that podocyte damage interferes with the circadian clock. To experimentally validate this link, we first generated circadian gene expression data from whole murine kidneys by performing transcriptomic profiling in 3-h time intervals over a 48-h period. Using these data, we identified genes with circadian expression oscillation and intersected this gene list with podocyte expression data to obtain a set of genes with circadian expression in podocytes (STAR Methods; Figures S11A and S11B). This gene set was indeed significantly enriched in podocyte NR1D1 target genes as determined in our TRN (Figure 5B).

We then asked whether increasing podocyte damage as measured by the PDS was associated with increased disruption of circadian gene expression. To this end, we employed two different methods to assess disruption of circadian gene expression in single cells—TEMPO,[93] which determines average time shifts between the expression of core clock genes and other circadian genes, and CRD,[85] which measures the variance among circadian genes. Here, we fully exploited the single-cell capacity of our score, as damage-dependent circadian expression heterogeneity was captured within one sample, with the organism normalized for all common factors that potentially affect circadian rhythms, such as cortisol levels, organism redox state, time of day, blood pressure, etc. Both approaches revealed that increased podocyte damage correlated with increased disruption of circadian gene expression within the sample, an effect that was shown across podocytopathy models (Figures 5C and 5D). We further confirmed this result by repeating the analysis using a recently published, podocyte-specific set of circadian genes, yielding an even more pronounced difference between controls and experimental samples (Figures S11C and S11D).[90]

We hypothesized that upon desynchronization of circadian gene expression, even within single glomeruli, the core circadian clock TF NR1D1 (Rev-Erb alpha) would show increased variance in nuclear protein levels and a shift in nuclear vs. cytoplasmic localization. Indeed, immunofluorescence co-staining for NR1D1 and WT1 revealed an increased variance of nuclear-to-cytoplasmic localization of NR1D1 in podocytes of *Nphs2*^{mut} mice compared to controls (Figure 5E). Densitometry for Nr1d1 further showed that the variance in nuclear NR1D1 levels increased with age and hence with progressive podocyte damage (Figure 5F). Of note, the *Nphs2*^{mut} model does not directly interfere with circadian clock components, supporting an effect of podocyte damage on the circadian clock. Similar to our PDS-based transcriptomic approach, this immunohistochemical approach was normalized and controlled for external effects

CellPress
OPEN ACCESS

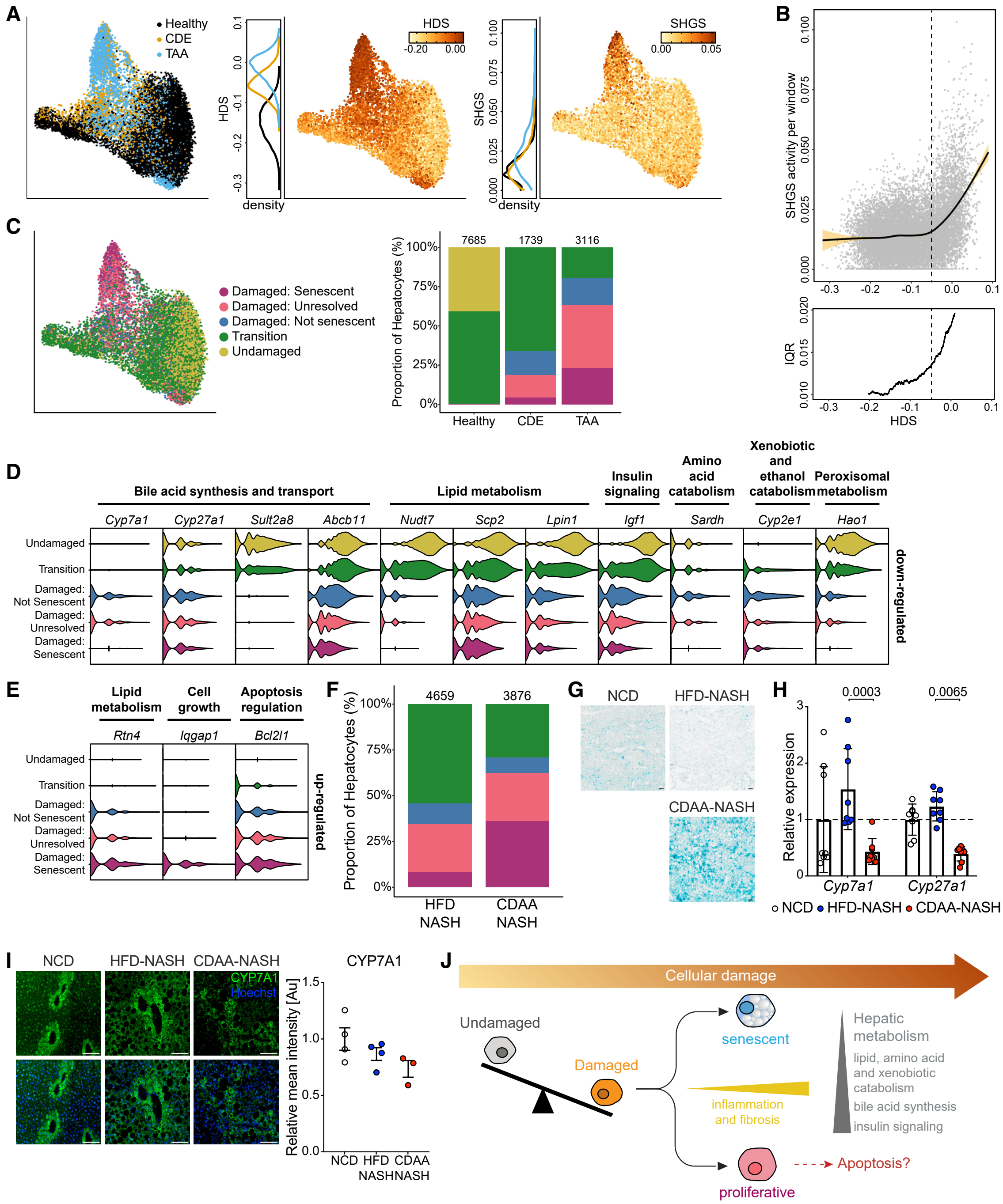

Figure 6. HDS reveals damage-driven cell-state transition of hepatocytes

(A) UMAP of merged snRNA-seq data from mice subjected to three different treatments. Healthy: normal chow; CDE: choline-deficient, ethionine-supplemented (CDE) diet; TAA: thioacetamide (TAA) in the drinking water (GEO: GSE200366,[98] $n = 3$ biological replicates per treatment). Left: UMAP of hepatocytes colored by condition. Middle: hepatocytes colored by HDS (color scale: floor and ceiling values corresponding to 1% and 99% quantiles of the HDS distribution across all samples). Right: hepatocytes colored by senescent hepatocyte gene signature (SHGS). Density plots next to UMAP show HDS and SHGS distribution per condition, respectively (see left UMAP plot for legend). HDS is significantly higher in both CDE and TAA than in controls (one-sided Wilcoxon rank-sum test; for both comparisons, $p < 2.2 \times 10^{-16}$).

(B) SHGS activity score vs. HDS (same data as in A). (Top) Solid line: generalized linear model line shows behavior of SHGS activity with increasing HDS. Orange: confidence interval of the regression line. The data are heteroscedastic (Breusch-Pagan test, $p < 2.2 \times 10^{-16}$). (Bottom) IQR per sliding window of 2,000 hepatocytes (y axis) plotted against mean HDS per sliding window. SHGS increase shows a tipping-point pattern beyond an HDS damage threshold of ~ -0.05.

(legend continued on next page)

on zeitgeber time, as nuclei within single glomeruli from the same animal were assessed.

In conclusion, disruption of circadian gene expression appears to be a common consequence of podocyte damage in response to multiple damage triggers. Given the large number of essential podocyte transcripts exhibiting circadian oscillation,[90] this finding bears significant relevance to understanding podocyte damage. Further, given that disruption of the clock, and hence circadian gene expression, results in podocyte damage[91] and podocyte damage results in disruption of circadian gene expression, positive feedback loops contributing to podocyte damage by means of this mechanism appear likely (Figure 5G). In summary, our integrative analysis of the PDS together with the TRN has yielded important insights into the mechanisms of podocyte damage.

HDS reveals metabolically distinct cell-state trajectories of damaged hepatocytes

Accumulation of damage can drive hepatocytes into highly distinct cellular states, including neoplastic transformation (i.e., establishing hepatocellular carcinoma [HCC]), cell death, and senescence.[94,95] The accumulation of hepatocyte senescence in MASLD has been shown to result in worse outcomes.[96] However, since the pathophysiological role of senescent hepatocytes in chronic liver disease has not been widely investigated nor fully understood, we focused specifically on the transition of damaged hepatocytes into this cell state.[94] To explore this cell-state transition, we used hepatocyte snRNA-seq data from mice either fed a choline-deficient and ethionine-supplemented (CDE) diet or treated with thioacetamide (TAA), as well as controls. While both liver disease models eventually evoke HCC, CDE elicits severe steatosis with little fibrosis at early stages, whereas TAA features only mild steatosis but severe inflammation, necrosis, and fibrosis.[97] Samples used for snRNA-seq were from mice sacrificed after 3 weeks of treatment, when they were precancerous.[98] Upon HDS analysis, greater average hepatocyte damage was seen in TAA hepatocytes compared to CDE or controls (Figure 6A).[59] To assess the senescence status of hepatocytes, we quantified the activity of the senescent hepatocyte gene set (SHGS) per single cell.[96] First, we evaluated the senescence status of cells in relation to the HDS and observed an apparent disease model-specific increase in senescent cells predominant in TAA-exposed hepatocytes (Figures 6A and S12A). Interestingly, with increasing cellular damage, this analysis revealed a "tipping point": until an HDS of about −0.05, we did not observe any noticeable increase in the activity of the senescence gene signature (Figure 6B). Beyond this threshold value, cells increasingly adopted a senescent-like state (Figure 6B). This tipping-point pattern was absent in cells from control animals, as hardly any cells reached the HDS threshold value (Figure S12B). However, the pattern was visible in both disease models (TAA and CDE). These findings indicated that the tipping point mostly depends on damage accumulation in individual hepatocytes (Figure S12B) but is independent of the specific disease model and the average damage level. Furthermore, cells began to adopt divergent cell states as HDS increased. Although a large fraction of cells became increasingly senescent-like, many other cells were able to maintain a low senescence score despite being substantially damaged according to their HDS. To formally quantify this notion, we computed the interquartile range (IQR) of the range of senescence scores as a function of HDS, which revealed a substantial increase in the IQR with increasing damage (Figure 6B). Thus, these findings support the notion that damaged hepatocytes develop along divergent trajectories toward distinct cellular states, one of them being cellular senescence. While cellular damage beyond a certain threshold seems to be a necessary requirement for entry into the senescent state, that cell-state transition does not seem to be fully deterministic, as a large fraction of highly damaged hepatocytes escapes entry into senescence. We

(C) Left: UMAP as shown in (A), colored by cell-state classification. Right: relative abundance of hepatocytes of each cell state per condition. Numbers above stacked bars show the total number of hepatocytes analyzed per condition ($n = 3$ per model).

(D) Gene expression of select genes from a group of 475 genes significantly (Bonferroni-adjusted $p < 0.05$ and log fold change [LFC] < -0.5; see also Table S8) downregulated in damaged senescent hepatocytes compared to damaged non-senescent hepatocytes. Bile acid metabolism is specifically downregulated in damaged senescent cells.

(E) Gene expression of selected genes of a total of 422 significantly (Bonferroni-adjusted $p < 0.05$ and LFC > 0.5; see also Table S7) differentially upregulated genes in damaged senescent hepatocytes compared to damaged non-senescent hepatocytes.

(F) Damaged hepatocyte cell-state assignment similar to (C), based on scRNA-seq data from two murine NASH models ($n = 1$, biological replicates per model): choline-deficient, L-amino acid-defined (CDAA) high-fat diet, and high-fat, high-fructose diet supplemented with trans fat and cholesterol (HFD) (GEO: GSE146049[99]). Cell-state classification was carried out based on HDS and SHGS as in (C). Numbers above stacked bars show the total number of hepatocytes. The color legend is the same as that in (C).

(G) Senescence-associated β-galactosidase staining of liver tissue derived from 26-week-old mice fed a CD, HFD-NASH, or CDAA-NASH diet (same experimental conditions as in F). Representative images of $n = 3$ mice per condition are shown. Scale bars: 100 μm.

(H) RT-qPCR for the expression of bile acid metabolism enzymes *Cyp7a1* and *Cyp27a1* in liver tissues of 26-week-old mice fed a CD, HFD-NASH, or CDAA-NASH diet. Gene expression is shown relative to CD (two-way ANOVA with Tukey's multiple comparisons test, $n = 8$). Whiskers represent ± SD.

(I) Left: representative immunofluorescence (IF) staining of liver tissue from NCD, HFD-NASH, and CDAA-NASH animals stained for CYP7A1 (green) and counterstained with Hoechst (blue). Livers were collected from 12- to 16-week-old NCD-fed mice or 26-week-old NASH-diet-fed mice ($n = 3$–4 biological replicates per condition). Scale bars: 100 μm. Right: quantification of CYP7A1 mean intensity across IF staining of NASH models as shown in (I). Data are represented as the mean of CYP7A1 intensity per mouse and relative to NCD ($n = 3$–4 mice per condition). Whiskers represent ± SEM.

(J) Schematic model of hepatocyte cell-state transitions. Accumulating damage leads to loss of cellular homeostasis, increasing profibrotic and proinflammatory signals. After a critical HDS is reached, hepatocytes begin transitioning into different pathological cell-state trajectories. When transiting in the direction of senescence, hepatocytes strongly downregulate genes involved in defining hepatic metabolic functions such as amino acid and xenobiotic catabolism, insulin signaling, and bile acid synthesis. In parallel, genes involved in lipid catabolism are downregulated, thereby further exacerbating lipid accumulation in senescent cells.

confirmed these results in another independent NASH snRNA-seq murine dataset (Figures S12C–S12E).[59] Furthermore, this tipping point was also preserved in human liver snRNA-seq data from patients suffering from NASH (Figures S12F–S12G). Of note, the value of the HDS tipping point remained consistently in the range of −0.05 between all three datasets (i.e., even across species), corroborating our previous analysis on the ability of the HDS to align independent studies (Figure S8B).

To characterize the transcriptional alterations associated with these cell-state transitions, we arbitrarily divided cells on the continuous HDS and SHGS[96] trajectories into three classes per score, respectively: for the HDS, cells were grouped into undamaged hepatocytes (low HDS), damaged hepatocytes (high HDS), and cells in a "transition" state with intermediate HDS. Damaged hepatocytes were further stratified on the SHGS scale as "senescent," "non-senescent," or "unresolved" depending on their senescence score (see STAR Methods). This grouping allowed us to quantify expression differences between the extremes on both scales. The relative abundances of hepatocytes in each cell-state category varied greatly between the CDE and TAA disease models (Figure 6C). Confirming our uniform manifold approximation and projection (UMAP) and density plots (Figure 6A), we observed a sharp increase in the fraction of damaged hepatocytes in TAA mice when compared to CDE mice: whereas ∼30% of CDE hepatocytes were categorized as damaged, this fraction of hepatocytes more than doubled in TAA-treated mice (∼75%; Figure 6C). Interestingly, despite almost identical HDS tipping-point values between CDE and TAA hepatocytes (Figure S12B), the relative contribution of senescent hepatocytes to all damaged hepatocytes was markedly elevated in TAA mice as compared to CDE mice (Figure 6C).

To better characterize these cell states, we determined genes that were differentially expressed between the senescent and non-senescent damaged hepatocytes (Figures 6D and 6E; Table S6). We found 422 genes with significantly increased expression and 475 genes with significantly decreased expression in senescent damaged hepatocytes (adjusted $p < 0.05$, absolute $\log_2$(fold change) > 0.5, and expressed in at least 10% of senescent or non-senescent damaged hepatocytes). Of the 422 genes significantly upregulated among the senescent cells, 30 were part of the SHGS.

Genes upregulated in senescent damaged hepatocytes were involved in cell adhesion, actin cytoskeleton remodeling, responses to wound healing, as well as apoptosis signaling, and cell growth (Figure S12H; Tables S7). In hepatocytes, the actin cytoskeleton is of great importance for maintaining the polarized cell structure and ensuring proper function of bile canaliculi.[100] A key gene in this context is *Iqgap1*, which encodes an actin-binding scaffold protein and signaling integration factor, which we found to be specifically upregulated in senescent damaged hepatocytes (Figure 6E). Another example is *Rtn4*, whose expression was also highly upregulated in senescent damaged hepatocytes and which has been linked to diet-induced fatty liver disease.[101] Further, we detected upregulation of *Bcl2l1* with increasing damage, reaching its highest levels in the senescent damaged hepatocytes (Figure 6E). *Bcl2l1* encodes an apoptosis inhibitor and exerts protective functions in hepatocytes, providing insight into functional consequences of the senescent cell state.[102] Genes downregulated in the senescent hepatocytes were enriched for hepatic metabolic functions such as lipid metabolism (e.g., *Nudt7*,[103] *Scp2*,[104] and *Lpin1*[105]), bile acid synthesis (e.g., *Cyp7a1*, *Cyp27a1*, *Abcb11*,[106] and *Sult2a8*[107]), amino acid catabolism (e.g., *Sardh*[108]), and xenobiotic breakdown (e.g., *Cyp2e1*[109]), as well as insulin signaling (e.g., *Igf1*[110]) (Figures 6D and S12I; Tables S7). For example, the expression of *Cyp7a1* and *Cyp27a1*, which encode central enzymes of the canonical and alternative bile acid biosynthetic pathways, was first upregulated with damage but then downregulated with entry into a senescent cell state (Figure 6D). Additional effectors of the bile acid synthesis pathway were first downregulated upon damage and displayed an exacerbated downregulation in senescent hepatocytes. This pattern was evident for *Sult2a8*, which encodes a sulfotransferase enzyme responsible for the detoxification of bile acids through sulfonation and the maintenance of a homeostatic bile acid pool,[107,111] and for *Abcb11*, which codes for the canalicular bile salt pump, the primary transporter for bile acid salt (Figure 6D).[106] In summary, liver snRNA-seq of murine NASH models indicated a drastic downregulation of bile acid metabolism in senescent damaged hepatocytes, which differentiated senescent cells from non-senescent hepatocytes at similar damage levels. To confirm this dysregulation of bile acid metabolism in senescent hepatocytes, we carried out additional experiments and analyses in high-fat diet (HFD)-NASH and choline-deficient l-amino acid-defined (CDAA)-NASH mouse models. These models display phenotypes comparable to the CDE and TAA models analyzed previously.[99] First, we reanalyzed snRNA-seq data from HFD-NASH and CDAA-NASH mice by calculating HDS and SHGS activity in hepatocytes.[99] On average, hepatocytes in the CDAA-NASH model had greater SHGS activity and HDS levels compared to HFD-NASH cells (Figures S12J and S12K). Furthermore, this analysis confirmed a relative cell composition between senescence and damage categories similar to CDE and TAA animals (Figure 6F). Importantly, in CDAA-NASH, more than 25% of all hepatocytes realized a senescent cell state (Figure 6F). Next, we assessed the prevalence of senescent cells in CDAA-NASH, HFD-NASH, and control livers using a senescence-associated β-galactosidase (SABG) assay. Indeed, SABG activity was visibly increased in CDAA-NASH samples when compared to HFD-NASH or control livers (Figure 6G), confirming increased levels of hepatocyte senescent cell states in CDAA-NASH mice. Subsequently, we confirmed that enzymes involved in bile acid metabolism were downregulated in CDAA-NASH mice along with increased senescence in this model. RT-qPCR showed that *Cyp7a1* and *Cyp27a1* mRNA levels were significantly downregulated in CDAA-NASH when compared to HFD-NASH mice (Figure 6H). Finally, we stained murine livers for CYP7A1, the key rate-limiting enzyme of canonical bile acid synthesis, by immunofluorescence and measured protein levels by densitometry (Figures 6I and 6J). Again, downregulation of CYP7A1 in CDAA-NASH, compared with HFD-NASH or control livers, respectively, was evident. Taken together, our findings confirm a metabolic shift toward decreased bile acid metabolism in senescent hepatocytes.

In summary, our observations support a model in which the emergence of a senescent phenotype in hepatocytes is in part

damage dependent, as a critical level of damage must be exceeded before an appreciable number of cells embark on a trajectory toward senescence. Yet damage alone does not fully explain why some cells become senescent and others do not (Figure 6K). Further, senescent and non-senescent damaged hepatocytes constitute two distinct cellular states that are metabolically highly divergent. According to our data, senescent hepatocytes may be key contributors to the development of steatosis, particularly due to their dysregulated lipid metabolism and repression of bile acid production.

DISCUSSION

Understanding how cellular damage accumulates and drives chronic metabolic and degenerative diseases remains one of the most pressing challenges in biomedical research. Here, we introduce a novel strategy that quantifies cellular damage at single-cell resolution, providing a powerful tool to dissect disease progression across diverse biological systems. This integrative experimental and computational strategy leverages cell-type-specific molecular markers to systematically stratify cells along a damage continuum, revealing fundamental mechanisms that underlie cellular dysfunction in chronic degenerative diseases. In contrast to conventional transcriptomic analyses that rely on discrete cell-state classifications (e.g., "diseased" vs. "healthy"), our strategy captures progressive molecular deterioration, offering a new paradigm to study the molecular pathogenesis of degenerative disorders. By leveraging information from prior transcriptomic studies, the PDSs and HDSs integrate diverse disease contexts into unified degeneration information. These damage scores allow for generating cellular damage trajectories that carry a defined interpretation and meaning. Damage trajectories stand in contrast to the established pseudotime trajectories, which have failed to detect the damage signal and are subject to speculative interpretations as to what molecular process they might represent. In addition, the possibility to align damage scores across studies enables the direct, quantitative comparison of damage trajectories between new data of a particular study with any earlier (single-cell) transcriptomic study of the same disease. Thereby, our approach also substantially advances mechanistic insight by facilitating the distinction of trigger-specific pathway changes from generic, damage-related changes. Cellular damage scores are not a competing alternative to established notions of senescence, inflammation, or fibrosis. These notions describe partially overlapping programs that can emerge at different positions along a deterioration trajectory. Damage scores instead provide a quantitative axis that allows one to ask when and how such programs are engaged and whether they arise gradually or after a threshold.

The scores provide a robust quantitative readout of cellular damage because marker genes were derived from a great diversity of studies and disease models and had to be reasonably expressed in the target cell type to ensure detectability in single-cell data. Importantly, the scoring is rank based, which makes it resistant to technical variability between cells (e.g., library size). As dozens of genes are integrated, the impact of individual "outlier" genes is reduced. Using a limited gene panel improves robustness to platform differences and provides a realistic path toward standardized measurements in small biopsies and spatially resolved tissue. Robustness of the scores has been confirmed via (1) varying the number of marker genes, which did not have any relevant effect on the results; (2) leave-one-model-out cross-validation; (3) validation using spatial transcriptomic data with matching histological information; (4) correlation of damage scores with clinical and morphological endpoints, such as ACR and SD length; (5) application in a different species (i.e., human); and (6) confirmation of findings with follow-up experiments.

Even though our strategy for selecting marker genes did not require that markers causally contribute to the disease, the composition of marker gene sets revealed exciting insight into the nature of cellular damage. In both cases (PDS and HDS), marker genes were enriched for highly cell-type-specific genes that were consistently repressed under disease conditions (Table S2). This observation is consistent with the notion that the loss of cell-type identity is a hallmark of cellular damage.[45,46,112,113] On the other hand, very few markers were related to the generic stress response, such as the integrated stress response pathway. While this may come as a surprise, generic stress markers are not necessarily good damage markers. Stress responses may be protective, adaptive, or transient. If a cell responds to stress in a physiologically appropriate way, this may not indicate cellular damage. Rather, the opposite may be true: a physiological stress response may be an indicator of high cellular fidelity. Thereby, our work highlights an important distinction between cellular stress and cellular damage.

Applying this concept to kidney and liver disease revealed conserved molecular programs linking metabolic stress to cellular dysfunction and degeneration. In podocytes, progressive injury was associated with a metabolic shift toward oxidative phosphorylation, most likely reflecting the energy demands of cytoskeletal remodeling in response to damage. By mapping the damage continuum, we could differentiate model-specific behavior of energy metabolism and tight junction pathways. In addition, our data established that focal adhesion components exhibit both universal and trigger-specific responses to injury, offering new insight into the structural adaptations of podocytes under stress. While it was already established that changes in the circadian rhythm impact podocytes,[90–92] we now provide the first direct evidence that podocyte damage actively disrupts circadian gene regulation, revealing a bidirectional interplay between circadian control and podocyte health—a critical finding that underscores the role of cell-intrinsic circadian dysregulation in disease progression.

In hepatocytes, we showed that progressive damage determines cell-state transitions, driving cells toward divergent cell states. Notably, we identified a tipping point in hepatocyte damage beyond which cells may enter a senescent state, leading to the repression of lipid and bile acid metabolism. These findings suggest that senescent hepatocytes may serve as drivers of tissue dysfunction, potentially fueling chronic inflammation and HCC development.[96] The ability to pinpoint this tipping point in two species represents a critical step toward defining therapeutic windows for intervention in liver disease progression.[114]

Beyond its biological insights, our damage-scoring framework has direct clinical implications. The fact that the damage scores

were readily applicable to human single-cell and diverse spatial omics platforms underlines the clinical applicability of our concept. As such, the pathway fingerprints derived from our approach can serve as diagnostic tools for the molecular characterization of patient biopsies, enabling subtype-specific stratification of disease states. Furthermore, by performing cross-model analysis, we identified decreased *Dpp4* expression levels as a conserved hallmark of podocyte damage, suggesting that DPP4 inhibitors—currently used in type 2 diabetes—could be repurposed for podocyte disease therapy. We demonstrate the feasibility and utility of this concept for two very distinct diseases: one affecting proliferating, metabolically highly active cells and the other affecting post-mitotic cells that primarily serve a physical function in their organ. Hence, this study establishes a universal blueprint for investigating degenerative diseases at a systems level, offering a scalable approach to quantify cellular damage across species and disease contexts.

The ability to apply our single-cell damage scores across *in vivo* omics modalities and cross-species datasets opens exciting new opportunities for decoding the mechanisms of chronic disease progression beyond the kidney and liver, with potential applications in neurodegeneration, fibrosis, and aging-related disorders. By shifting the focus from static disease endpoints to progressive, single-cell damage trajectories, our approach lays the foundation for a new generation of precision diagnostics and targeted therapies aimed at intercepting degenerative diseases at their earliest stages.

Limitations of the study

The damage scores are RNA based, which makes them readily applicable to a great diversity of platforms and assays, including single-cell and spatial transcriptomics data. However, this approach may be limited in capturing cellular state transitions that do not leave a clear transcriptomic signature, such as the terminal phases during apoptosis.

Especially in the case of single-cell data, the damage scores reflect a composite measure of damage occurring *in vivo* and during sample processing. Cell lysis and single-cell processing in microfluidic devices might additionally damage cells, which potentially contributes to measured damage scores. Indeed, we noted that PDSs obtained from single-cell data were on average slightly greater than PDSs obtained from single-nucleus data. This observation is consistent with the notion that the handling of live cells can lead to extra damage along with respective transcriptional "traces." Yet, as long as all cells are treated equally and in parallel, such a bias would not affect relative differences between cells. Importantly, we found that the small differences between scRNA-seq and snRNA-seq data did not compromise the comparability of scores between studies (Figure S8A).

While this study clearly demonstrated how molecular damage scores can be utilized to obtain insight into disease mechanisms, future work needs to explore clinical applications more deeply. For example, our study did not assess the prognostic value of the damage scores or pathway signatures derived from them. Further, interpretation of the human data is constrained by data availability. For example, cellular damage in humans is expected to result from a combination of the disease (including genetic predisposition), age, and lifestyle. While our study clearly shows the contribution of disease and age, we lacked information on lifestyle factors to also demonstrate their correlation with the PDS or HDS.

Finally, while the PDS and HDS represent a significant step forward, they are likely not yet perfect. As larger training datasets become available, incorporating machine learning-based approaches will be essential. The application of pre-trained foundational models tailored to specific diseases and cell types seems like a powerful and exciting extension of our framework.

RESOURCE AVAILABILITY

Lead contact

Requests for further information and resources should be directed to and will be fulfilled by the lead contact, Andreas Beyer (andreas.beyer@uni-koeln.de).

Materials availability

All unique/stable reagents generated in this study are available from the lead contact without restriction.

Data and code availability

- All sequencing data generated within the study have been deposited at Gene Expression Omnibus (GEO) under the accession numbers GEO: GSE289878, GSE289795, GSE289877 and are publicly available as of the date of publication.
- Xenium spatial transcriptomic data and de-identified clinical metadata have been deposited in the Zenodo database and can be accessed at Zenodo: https://doi.org/10.5281/zenodo.15746500.
- All original code has been deposited at Zenodo and is publicly available at Zenodo: https://doi.org/10.5281/zenodo.19916603 and https://doi.org/10.5281/zenodo.20087796 as of the date of publication.
- Any additional information required to reanalyze the data reported in this paper is available from the lead contact upon request.

ACKNOWLEDGMENTS

We thank Christoph Schell for valuable discussions and for providing protein interaction networks and Martin Höhne for support with image quantification macros. The Wt1 knockout mouse was a gift from Jordan A. Kreidberg. We thank the FACS & Imaging Core Facility at the Max Planck Institute for Biology of Aging, Cologne, and the CECAD Imaging Facility, Cologne, for their support in flow cytometry and imaging acquisition, respectively. We thank Simon Tröder and Branko Zevnik from the *in vivo* Research Facility at CECAD Cologne for technical support in mouse breeding and transgenics. We thank S. Lück, P. Thaben, and A. Kramer for assistance with circadian bulk RNA-seq mouse experiments. We thank Vanessa Künstler for expert technical assistance in acquiring Xenium spatial transcriptomic data and Beate Rygol for expert technical assistance in preparation of tissues for spatial transcriptomics. We thank Life Science Editors for their edits of this manuscript. H.C. and P.U.A. received support from the Cologne Graduate School of Ageing Research. This study was conducted within CRU329, CRC 1678, and CRC TRR422 at the University of Cologne and was supported by grants from the Deutsche Forschungsgemeinschaft (DFG, German Research Foundation; CRC 1678 Project IDs 520471345 to R.-U.M. and A.B.; BE2603/10-1 and BE2603/10-2 to A.B.; KA3217/5-1, KA3217/5-2, and KA3217/6-1 to M.K.; MU3629/4-1 to R.-U.M.; BR6668/2-1 to F.B.; BR2955/8-1 to P.T.B.; SCHE1562/7-1 and SCHE1562/7-2 to B.S.; BE2212/23-1 and BE2212/23-2 to T.B., and CRC TRR 422 to T.B., A.B., F.B., P.T.B., L.B., M.K., R.-U.M., and B.S.). The work was further supported by the German Ministry for Science and Education through research grants 0315899 to P.O.W. and 01EO2106 to F.B. and through the STOP-FSGS consortium 01GM1901E to P.T.B. and T.B.

AUTHOR CONTRIBUTIONS

T.P., P.U.A., and H.C. contributed equally to this work. M.K. and A.B. jointly supervised the project. T.P., P.U.A., H.C., M.K., and A.B. conceptualized the study. T.P. and P.U.A. curated data and performed computational analyses. H.C., M.-K.K., C.V., P.W., L.B., D.U.-J., and C.K. carried out experiments and analyzed data. F.B., P.O.W., and R.-U.M. generated the data for circadian rhythm analyses. C.O., P.T.B., and B.S. generated mouse models. A.-L.S. and K.-F.D. provided expertise in flow cytometry cell sorting and cell sorting resources. H.G. provided expertise in renal pathology and tissue-handling resources and prepared human samples for spatial transcriptomics. C.K. provided expertise in spatial transcriptomics and spatial transcriptomics resources. T.P., P.U.A., H.C., M.K., and A.B. visualized the data and generated the figures. F.T.W. provided expertise on liver biology and validated the results. T.B. provided expertise on podocyte biology. T.P., P.U.A., H.C., T.B., M.K., and A.B. wrote the original manuscript draft and revised and edited the manuscript. All authors approved the manuscript.

DECLARATION OF INTERESTS

The authors declare no competing interests.

STAR★METHODS

Detailed methods are provided in the online version of this paper and include the following:

- KEY RESOURCES TABLE
- EXPERIMENTAL MODEL AND STUDY PARTICIPANT DETAILS
  - Animals
  - Human clinical sample acquisition
- METHOD DETAILS
  - Isolation of glomeruli and nuclei
  - ATAC-seq
  - Single-nuclei RNA-seq
  - Bulk RNA-seq
  - Xenium spatial transcriptomics
  - STED microscopy and quantification
  - Albumin-to-creatinine ratio quantification
  - Immunofluorescence and quantification
  - RT-qPCR
  - Senescence-associated β-gal staining
  - Transcriptomic data retrieval and processing
  - RNA-seq data processing
  - Sn- and sc-RNA-seq data processing
  - ATAC-seq data processing
  - Spatial transcriptomics data analysis
  - Damage score algorithm
  - Damage score validation
  - Circadian clock analysis
- QUANTIFICATION AND STATISTICAL ANALYSIS

SUPPLEMENTAL INFORMATION

Supplemental information can be found online at https://doi.org/10.1016/j.xgen.2026.101337.

Received: February 20, 2026
Revised: April 7, 2026
Accepted: July 23, 2026
Published: August 20, 2026

REFERENCES

1. Hodgin, J.B., Mariani, L.H., Zee, J., Liu, Q., Smith, A.R., Eddy, S., Hartman, J., Hamidi, H., Gaut, J.P., Palmer, M.B., et al. (2022). Quantification of Glomerular Structural Lesions: Associations With Clinical Outcomes and Transcriptomic Profiles in Nephrotic Syndrome. Am. J. Kidney Dis. *79*, 807–819.e1. https://doi.org/10.1053/J.AJKD.2021.10.004.
2. Lake, B.B., Menon, R., Winfree, S., Hu, Q., Melo Ferreira, R., Kalhor, K., Barwinska, D., Otto, E.A., Ferkowicz, M., Diep, D., et al. (2023). An atlas of healthy and injured cell states and niches in the human kidney. Nature *619*, 585–594. https://doi.org/10.1038/S41586-023-05769-3.
3. Menon, R., Otto, E.A., Hoover, P., Eddy, S., Mariani, L., Godfrey, B., Berthier, C.C., Eichinger, F., Subramanian, L., Harder, J., et al. (2020). Single cell transcriptomics identifies focal segmental glomerulosclerosis remission endothelial biomarker. JCI Insight *5*, e133267. https://doi.org/10.1172/JCI.INSIGHT.133267.
4. Mariani, L.H., Eddy, S., AlAkwaa, F.M., McCown, P.J., Harder, J.L., Nair, V., Eichinger, F., Martini, S., Ademola, A.D., Boima, V., et al. (2023). Precision nephrology identified tumor necrosis factor activation variability in minimal change disease and focal segmental glomerulosclerosis. Kidney Int. *103*, 565–579. https://doi.org/10.1016/J.KINT.2022.10.023.
5. McNulty, M.T., Fermin, D., Eichinger, F., Jang, D., Kretzler, M., Burtt, N.P., Pollak, M.R., Flannick, J., Weins, A., Friedman, D.J., et al. (2022). A glomerular transcriptomic landscape of apolipoprotein L1 in Black patients with focal segmental glomerulosclerosis. Kidney Int. *102*, 136–148. https://doi.org/10.1016/J.KINT.2021.10.041.
6. Powell, E.E., Wong, V.W.S., and Rinella, M. (2021). Non-alcoholic fatty liver disease. Lancet *397*, 2212–2224. https://doi.org/10.1016/S0140-6736(20)32511-3.
7. Ma, C., Kesarwala, A.H., Eggert, T., Medina-Echeverz, J., Kleiner, D.E., Jin, P., Stroncek, D.F., Terabe, M., Kapoor, V., ElGindi, M., et al. (2016). NAFLD causes selective CD4(+) T lymphocyte loss and promotes hepatocarcinogenesis. Nature *531*, 253–257. https://doi.org/10.1038/NATURE16969.
8. Guilliams, M., Bonnardel, J., Haest, B., Vanderborght, B., Wagner, C., Remmerie, A., Bujko, A., Martens, L., Thoné, T., Browaeys, R., et al. (2022). Spatial proteogenomics reveals distinct and evolutionarily conserved hepatic macrophage niches. Cell *185*, 379–396.e38. https://doi.org/10.1016/J.CELL.2021.12.018.
9. Mathys, H., Davila-Velderrain, J., Peng, Z., Gao, F., Mohammadi, S., Young, J.Z., Menon, M., He, L., Abdurrob, F., Jiang, X., et al. (2019). Single-cell transcriptomic analysis of Alzheimer's disease. Nature *570*, 332–337. https://doi.org/10.1038/S41586-019-1195-2.
10. Bravo González-Blas, C., De Winter, S., Hulselmans, G., Hecker, N., Matetovici, I., Christiaens, V., Poovathingal, S., Wouters, J., Aibar, S., and Aerts, S. (2023). SCENIC+: single-cell multiomic inference of enhancers and gene regulatory networks. Nat. Methods *20*, 1355–1367. https://doi.org/10.1038/S41592-023-01938-4.
11. Aibar, S., González-Blas, C.B., Moerman, T., Huynh-Thu, V.A., Imrichova, H., Hulselmans, G., Rambow, F., Marine, J.C., Geurts, P., Aerts, J., et al. (2017). SCENIC: single-cell regulatory network inference and clustering. Nat. Methods *14*, 1083–1086. https://doi.org/10.1038/nmeth.4463.
12. Habenicht, L.K.L., Wang, Z., Zhang, X., Li, Y., Mogler, C., Huspenina, J.S., Schmid, R.M., Weber, C., Mohanta, S.K., Ma, Z., and Yin, C. (2022). The C1q-ApoE complex: A new hallmark pathology of viral hepatitis and nonalcoholic fatty liver disease. Front. Immunol. *13*, 970938. https://doi.org/10.3389/FIMMU.2022.970938.
13. Marshall, J.L., Noel, T., Wang, Q.S., Chen, H., Murray, E., Subramanian, A., Vernon, K.A., Bazua-Valenti, S., Liguori, K., Keller, K., et al. (2022). High-resolution Slide-seqV2 spatial transcriptomics enables discovery of disease-specific cell neighborhoods and pathways. iScience *25*, 104097. https://doi.org/10.1016/J.ISCI.2022.104097.
14. Hatje, F.A., Wedekind, U., Sachs, W., Loreth, D., Reichelt, J., Demir, F., Kosub, C., Heintz, L., Tomas, N.M., Huber, T.B., et al. (2021). Tripartite separation of glomerular cell types and proteomes from reporter-free mice. J. Am. Soc. Nephrol. *32*, 2175–2193. https://doi.org/10.1681/ASN.2020091346/-/DCSUPPLEMENTAL.

CellPress
OPEN ACCESS

15. Yamada, H., Shirata, N., Makino, S., Miyake, T., Trejo, J.A.O., Yamamoto-Nonaka, K., Kikyo, M., Empitu, M.A., Kadariswantiningsih, I.N., Kimura, M., et al. (2021). MAGI-2 orchestrates the localization of backbone proteins in the slit diaphragm of podocytes. Kidney Int. *99*, 382–395. https://doi.org/10.1016/J.KINT.2020.09.027.

16. Herwig, J., Skuza, S., Sachs, W., Sachs, M., Failla, A.V., Rune, G., Meyer, T.N., Fester, L., and Meyer-Schwesinger, C. (2019). Thrombospondin Type 1 Domain-Containing 7A Localizes to the Slit Diaphragm and Stabilizes Membrane Dynamics of Fully Differentiated Podocytes. J. Am. Soc. Nephrol. *30*, 824–839. https://doi.org/10.1681/ASN.2018090941.

17. Pisarek-Horowitz, A., Fan, X., Kumar, S., Rasouly, H.M., Sharma, R., Chen, H., Coser, K., Bluette, C.T., Hirenallur-Shanthappa, D., Anderson, S.R., et al. (2020). Loss of Roundabout Guidance Receptor 2 (Robo2) in Podocytes Protects Adult Mice from Glomerular Injury by Maintaining Podocyte Foot Process Structure. Am. J. Pathol. *190*, 799–816. https://doi.org/10.1016/j.ajpath.2019.12.009.

18. Empitu, M.A., Kikyo, M., Shirata, N., Yamada, H., Makino, S.I., Kadariswantiningsih, I.N., Aizawa, M., Patrakka, J., Nishimori, K., and Asanuma, K. (2023). Inhibition of Importin- α -Mediated Nuclear Localization of Dendrin Attenuates Podocyte Loss and Glomerulosclerosis. J. Am. Soc. Nephrol. *34*, 1222–1239. https://doi.org/10.1681/ASN.0000000000000150.

19. Hinkes, B., Wiggins, R.C., Gbadegesin, R., Vlangos, C.N., Seelow, D., Nürnberg, G., Garg, P., Verma, R., Chaib, H., Hoskins, B.E., et al. (2006). Positional cloning uncovers mutations in PLCE1 responsible for a nephrotic syndrome variant that may be reversible. Nat. Genet. *38*, 1397–1405. https://doi.org/10.1038/NG1918.

20. Kann, M., Ettou, S., Jung, Y.L., Lenz, M.O., Taglienti, M.E., Park, P.J., Schermer, B., Benzing, T., and Kreidberg, J.A. (2015). Genome-wide analysis of Wilms' tumor 1-controlled gene expression in podocytes reveals key regulatory mechanisms. J. Am. Soc. Nephrol. *26*, 2097–2104. https://doi.org/10.1681/ASN.2014090940/-/DCSUPPLEMENTAL.

21. Ozaltin, F., Ibsirlioglu, T., Taskiran, E.Z., Baydar, D.E., Kaymaz, F., Buyukcelik, M., Kilic, B.D., Balat, A., Iatropoulos, P., Asan, E., et al. (2011). Disruption of PTPRO causes childhood-onset nephrotic syndrome. Am. J. Hum. Genet. *89*, 139–147. https://doi.org/10.1016/J.AJHG.2011.05.026.

22. Usui, T., Morito, N., Shawki, H.H., Sato, Y., Tsukaguchi, H., Hamada, M., Jeon, H., Yadav, M.K., Kuno, A., Tsunakawa, Y., et al. (2020). Transcription factor MafB in podocytes protects against the development of focal segmental glomerulosclerosis. Kidney Int. *98*, 391–403. https://doi.org/10.1016/J.KINT.2020.02.038.

23. Endlich, N., Kliewe, F., Kindt, F., Schmidt, K., Kotb, A.M., Artelt, N., Lindenmeyer, M.T., Cohen, C.D., Döring, F., Kuss, A.W., et al. (2018). The transcription factor Dach1 is essential for podocyte function. J. Cell Mol. Med. *22*, 2656–2669. https://doi.org/10.1111/JCMM.13544.

24. Sun, H., Li, H., Yan, J., Wang, X., Xu, M., Wang, M., Fan, B., Liu, J., Lin, N., Wang, X., et al. (2022). Loss of CLDN5 in podocytes deregulates WIF1 to activate WNT signaling and contributes to kidney disease. Nat. Commun. *13*, 1600. https://doi.org/10.1038/S41467-022-29277-6.

25. Sippl, C., Zeilbeck, L.F., Fuchshofer, R., and Tamm, E.R. (2014). Optineurin associates with the podocyte Golgi complex to maintain its structure. Cell Tissue Res. *358*, 567–583. https://doi.org/10.1007/S00441-014-1968-8.

26. Kohjima, M., Noda, Y., Takeya, R., Saito, N., Takeuchi, K., and Sumimoto, H. (2002). PAR3beta, a novel homologue of the cell polarity protein PAR3, localizes to tight junctions. Biochem. Biophys. Res. Commun. *299*, 641–646. https://doi.org/10.1016/s0006-291x(02)02698-0.

27. Köttgen, A., Pattaro, C., Böger, C.A., Fuchsberger, C., Olden, M., Glazer, N.L., Parsa, A., Gao, X., Yang, Q., Smith, A.V., et al. (2010). New loci associated with kidney function and chronic kidney disease. Nat. Genet. *42*, 376–384. https://doi.org/10.1038/ng.568.

28. Ishibashi, R., Takemoto, M., Akimoto, Y., Ishikawa, T., He, P., Maezawa, Y., Sakamoto, K., Tsurutani, Y., Ide, S., Ide, K., et al. (2016). A novel podocyte gene, semaphorin 3G, protects glomerular podocyte from lipopolysaccharide-induced inflammation. Sci. Rep. *6*, 25955. https://doi.org/10.1038/SREP25955.

29. Li-Hawkins, J., Lund, E.G., Bronson, A.D., and Russell, D.W. (2000). Expression cloning of an oxysterol 7alpha-hydroxylase selective for 24-hydroxycholesterol. J. Biol. Chem. *275*, 16543–16549. https://doi.org/10.1074/JBC.M001810200.

30. Mattow, J., Demuth, I., Haeselbarth, G., Jungblut, P.R., and Klose, J. (2006). Selenium-binding protein 2, the major hepatic target for acetaminophen, shows sex differences in protein abundance. Electrophoresis *27*, 1683–1691. https://doi.org/10.1002/ELPS.200500703.

31. Zhang, Y., Chen, X., Cao, Y., and Yang, Z. (2021). C8B in Complement and Coagulation Cascades Signaling Pathway is a predictor for Survival in HBV-Related Hepatocellular Carcinoma Patients. Cancer Manag. Res. *13*, 3503–3515. https://doi.org/10.2147/CMAR.S302917.

32. Ahmed, S.M.Q., Sasikumar, J., Laha, S., and Das, S.P. (2024). Multifaceted role of the DNA replication protein MCM10 in maintaining genome stability and its implication in human diseases. Cancer Metastasis Rev. *43*, 1353–1371. https://doi.org/10.1007/S10555-024-10209-3.

33. Krick, S., Shi, S., Ju, W., Faul, C., Tsai, S.Y., Mundel, P., and Böttinger, E.P. (2008). Mpv17l protects against mitochondrial oxidative stress and apoptosis by activation of Omi/HtrA2 protease. Proc. Natl. Acad. Sci. USA *105*, 14106–14111. https://doi.org/10.1073/PNAS.0801146105.

34. Merk, M., Zierow, S., Leng, L., Das, R., Du, X., Schulte, W., Fan, J., Lue, H., Chen, Y., Xiong, H., et al. (2011). The D-dopachrome tautomerase (DDT) gene product is a cytokine and functional homolog of macrophage migration inhibitory factor (MIF). Proc. Natl. Acad. Sci. USA *108*, E577–E585. https://doi.org/10.1073/PNAS.1102941108.

35. Song, J., Baek, I.J., Park, S., Oh, J., Kim, D., Song, K., Kim, M.K., Lee, H.W., Jang, B.K., and Jin, E.J. (2022). Deficiency of peroxisomal NUDT7 stimulates de novo lipogenesis in hepatocytes. iScience *25*, 105135. https://doi.org/10.1016/J.ISCI.2022.105135.

36. Kim, S.C., Kim, C.K., Axe, D., Cook, A., Lee, M., Li, T., Smallwood, N., Chiang, J.Y.L., Hardwick, J.P., Moore, D.D., and Lee, Y.K. (2014). All-trans-retinoic acid ameliorates hepatic steatosis in mice by a novel transcriptional cascade. Hepatology *59*, 1750–1760. https://doi.org/10.1002/HEP.26699.

37. Deng, Y., Zhao, Z., Sheldon, M., Zhao, Y., Teng, H., Martinez, C., Zhang, J., Lin, C., Sun, Y., Yao, F., et al. (2024). LIFR regulates cholesterol-driven bidirectional hepatocyte-neutrophil cross-talk to promote liver regeneration. Nat. Metab. *6*, 1756–1774. https://doi.org/10.1038/S42255-024-01110-Y.

38. Bartoli, D., Piobbico, D., Bellet, M.M., Bennati, A.M., Roberti, R., Della Fazia, M.A., and Servillo, G. (2016). Impaired cell proliferation in regenerating liver of 3 β-hydroxysterol Δ14-reductase (TM7SF2) knock-out mice. Cell Cycle *15*, 2164–2173. https://doi.org/10.1080/15384101.2016.1195939.

39. Hao, J.W., Wang, J., Guo, H., Zhao, Y.Y., Sun, H.H., Li, Y.F., Lai, X.Y., Zhao, N., Wang, X., Xie, C., et al. (2020). CD36 facilitates fatty acid uptake by dynamic palmitoylation-regulated endocytosis. Nat. Commun. *11*, 4765. https://doi.org/10.1038/S41467-020-18565-8.

40. Arai, T., Ono, Y., Arimura, Y., Sayama, K., Suzuki, T., Shinjo, S., Kanai, M., Abe, S.I., Semba, K., and Goda, N. (2017). Type I neuregulin1α is a novel local mediator to suppress hepatic gluconeogenesis in mice. Sci. Rep. *7*, 42959. https://doi.org/10.1038/SREP42959.

41. Verhague, M.A., Cheng, D., Weinberg, R.B., and Shelness, G.S. (2013). Apolipoprotein A-IV expression in mouse liver enhances triglyceride secretion and reduces hepatic lipid content by promoting very low density lipoprotein particle expansion. Arterioscler. Thromb. Vasc. Biol. *33*, 2501–2508. https://doi.org/10.1161/ATVBAHA.113.301948.

42. Zhang, D., Utsumi, T., Huang, H.C., Gao, L., Sangwung, P., Chung, C., Shibao, K., Okamoto, K., Yamaguchi, K., Groszmann, R.J., et al. (2011). Reticulon 4B (Nogo-B) is a novel regulator of hepatic fibrosis. Hepatology *53*, 1306–1315. https://doi.org/10.1002/HEP.24200.

43. Ma, Y., Chang, N., Liu, Y., Liu, F., Dong, C., Hou, L., Qi, C., Yang, L., and Li, L. (2022). Silencing IQGAP1 alleviates hepatic fibrogenesis via blocking bone marrow mesenchymal stromal cell recruitment to fibrotic liver. Mol. Ther. Nucleic Acids *27*, 471–483. https://doi.org/10.1016/J.OMTN.2021.12.020.

44. Zoheir, K.M.A., Abd-Rabou, A.A., Darwish, A.M., Abdelhafez, M.A., and Mahrous, K.F. (2022). Inhibition of induced-hepatic cancer in vivo through IQGAP1-shRNA gene therapy and modulation of TRAIL-induced apoptosis pathway. Front. Oncol. *12*, 998247. https://doi.org/10.3389/FONC.2022.998247.

45. Lu, Y., Ye, Y., Bao, W., Yang, Q., Wang, J., Liu, Z., and Shi, S. (2017). Genome-wide identification of genes essential for podocyte cytoskeletons based on single-cell RNA sequencing. Kidney Int. *92*, 1119–1129. https://doi.org/10.1016/j.kint.2017.04.022.

46. Berasain, C., Arechederra, M., Argemí, J., Fernández-Barrena, M.G., and Avila, M.A. (2023). Loss of liver function in chronic liver disease: An identity crisis. J. Hepatol. *78*, 401–414. https://doi.org/10.1016/J.JHEP.2022.09.001.

47. Wada, Y., Kidokoro, K., Kondo, M., Tokuyama, A., Kadoya, H., Nagasu, H., Kanda, E., Sasaki, T., Cherney, D.Z.I., and Kashihara, N. (2024). Evaluation of glomerular hemodynamic changes by sodium-glucose-transporter 2 inhibition in type 2 diabetic rats using in vivo imaging. Kidney Int. *106*, 408–418. https://doi.org/10.1016/J.KINT.2024.05.006.

48. Butt, L., Unnersjö-Jess, D., Höhne, M., Edwards, A., Binz-Lotter, J., Reilly, D., Hahnfeldt, R., Ziegler, V., Fremter, K., Rinschen, M.M., et al. (2020). A molecular mechanism explaining albuminuria in kidney disease. Nat. Metab. *2*, 461–474. https://doi.org/10.1038/S42255-020-0204-Y.

49. Tesch, F., Siegerist, F., Hay, E., Artelt, N., Daniel, C., Amann, K., Zimmermann, U., Kavvadas, P., Grisk, O., Chadjichristos, C., et al. (2021). Super-resolved local recruitment of CLDN5 to filtration slits implicates a direct relationship with podocyte foot process effacement. J. Cell Mol. Med. *25*, 7631–7641. https://doi.org/10.1111/JCMM.16519.

50. Fukusumi, Y., Zhang, Y., Yamagishi, R., Oda, K., Watanabe, T., Matsui, K., and Kawachi, H. (2018). Nephrin-Binding Ephrin-B1 at the Slit Diaphragm Controls Podocyte Function through the JNK Pathway. J. Am. Soc. Nephrol. *29*, 1462–1474. https://doi.org/10.1681/ASN.2017090993.

51. Gluchowski, N.L., Becuwe, M., Walther, T.C., and Farese, R.V. (2017). Lipid droplets and liver disease: from basic biology to clinical implications. Nat. Rev. Gastroenterol. Hepatol. *14*, 343–355. https://doi.org/10.1038/NRGASTRO.2017.32.

52. Brinkkoetter, P.T., Ising, C., and Benzing, T. (2013). The role of the podocyte in albumin filtration. Nat. Rev. Nephrol. *9*, 328–336. https://doi.org/10.1038/NRNEPH.2013.78.

53. Hengel, F.E., Dehde, S., Lassé, M., Zahner, G., Seifert, L., Schnarre, A., Kretz, O., Demir, F., Pinnschmidt, H.O., Grahammer, F., et al. (2024). Autoantibodies Targeting Nephrin in Podocytopathies. N. Engl. J. Med. *391*, 422–433. https://doi.org/10.1056/NEJMoa2314471.

54. Koehler, S., Kuczkowski, A., Kuehne, L., Jüngst, C., Hoehne, M., Grahammer, F., Eddy, S., Kretzler, M., Beck, B.B., Höhfeld, J., et al. (2020). Proteome Analysis of Isolated Podocytes Reveals Stress Responses in Glomerular Sclerosis. J. Am. Soc. Nephrol. *31*, 544–559. https://doi.org/10.1681/ASN.2019030312.

55. Williams, E.G., Pfister, N., Roy, S., Statzer, C., Haverty, J., Ingels, J., Bohl, C., Hasan, M., Čuklina, J., Bühlmann, P., et al. (2022). Multiomic profiling of the liver across diets and age in a diverse mouse population. Cell Syst. *13*, 43–57.e6. https://doi.org/10.1016/j.cels.2021.09.005.

56. Völker, L.A., Ehren, R., Grundmann, F., Benzing, T., Weber, L.T., and Brinkkötter, P.T. (2019). A newly established clinical registry of minimal change disease and focal and segmental glomerulosclerosis in Germany. Nephrol. Dial. Transplant. *34*, 1983–1986. https://doi.org/10.1093/ndt/gfz046.

57. Latt, K.Z., Heymann, J., Jessee, J.H., Rosenberg, A.Z., Berthier, C.C., Arazi, A., Eddy, S., Yoshida, T., Zhao, Y., Chen, V., et al. (2022). Urine Single-Cell RNA Sequencing in Focal Segmental Glomerulosclerosis Reveals Inflammatory Signatures. Kidney Int. Rep. *7*, 289–304. https://doi.org/10.1016/J.EKIR.2021.11.005.

58. Mitrofanova, A., Molina, J., Varona Santos, J., Guzman, J., Morales, X.A., Ducasa, G.M., Bryn, J., Sloan, A., Volosenco, I., Kim, J.J., et al. (2018). Hydroxypropyl-β-cyclodextrin protects from kidney disease in experimental Alport syndrome and focal segmental glomerulosclerosis. Kidney Int. *94*, 1151–1159. https://doi.org/10.1016/J.KINT.2018.06.031.

59. Xiao, Y., Batmanov, K., Hu, W., Zhu, K., Tom, A.Y., Guan, D., Jiang, C., Cheng, L., McCright, S.J., Yang, E.C., et al. (2023). Hepatocytes demarcated by EphB2 contribute to the progression of nonalcoholic steatohepatitis. Sci. Transl. Med. *15*, eadc9653. https://doi.org/10.1126/SCITRANSLMED.ADC9653.

60. Watson, B.R., Paul, B., Rahman, R.U., Amir-Zilberstein, L., Segerstolpe, Å., Epstein, E.T., Murphy, S., Geistlinger, L., Lee, T., Shih, A., et al. (2025). Spatial transcriptomics of healthy and fibrotic human liver at single-cell resolution. Nat. Commun. *16*, 319. https://doi.org/10.1038/s41467-024-55325-4.

61. Saelens, W., Cannoodt, R., Todorov, H., and Saeys, Y. (2019). A comparison of single-cell trajectory inference methods. Nat. Biotechnol. *37*, 547–554. https://doi.org/10.1038/S41587-019-0071-9.

62. Ji, Z., and Ji, H. (2016). TSCAN: Pseudo-time reconstruction and evaluation in single-cell RNA-seq analysis. Nucleic Acids Res. *44*, e117. https://doi.org/10.1093/NAR/GKW430.

63. Trapnell, C., Cacchiarelli, D., Grimsby, J., Pokharel, P., Li, S., Morse, M., Lennon, N.J., Livak, K.J., Mikkelsen, T.S., and Rinn, J.L. (2014). The dynamics and regulators of cell fate decisions are revealed by pseudotemporal ordering of single cells. Nat. Biotechnol. *32*, 381–386. https://doi.org/10.1038/nbt.2859.

64. Maier, J.I., Rogg, M., Helmstädter, M., Sammarco, A., Schilling, O., Sabass, B., Miner, J.H., Dengjel, J., Walz, G., Werner, M., et al. (2021). EPB41L5 controls podocyte extracellular matrix assembly by adhesome-dependent force transmission. Cell Rep. *34*, 108883. https://doi.org/10.1016/J.CELREP.2021.108883.

65. Chung, J.J., Goldstein, L., Chen, Y.J.J., Lee, J., Webster, J.D., Roose-Girma, M., Paudyal, S.C., Modrusan, Z., Dey, A., and Shaw, A.S. (2020). Single-cell transcriptome profiling of the kidney glomerulus identifies key cell types and reactions to injury. J. Am. Soc. Nephrol. *31*, 2341–2354. https://doi.org/10.1681/ASN.2020020220/-/DCSUPPLEMENTAL.

66. Fukasawa, H., Bornheimer, S., Kudlicka, K., and Farquhar, M.G. (2009). Slit diaphragms contain tight junction proteins. J. Am. Soc. Nephrol. *20*, 1491–1503. https://doi.org/10.1681/ASN.2008101117.

67. Kocylowski, M.K., Aypek, H., Bildl, W., Helmstädter, M., Trachte, P., Dumoulin, B., Wittösch, S., Kühne, L., Aukschun, U., Teetzen, C., et al. (2022). A slit-diaphragm-associated protein network for dynamic control of renal filtration. Nat. Commun. *13*, 6446. https://doi.org/10.1038/S41467-022-33748-1.

68. Schlosser, P., Surapaneni, A.L., Borisov, O., Schmidt, I.M., Zhou, L., Anderson, A., Deo, R., Dubin, R., Ganz, P., He, J., et al. (2024). Association of Integrated Proteomic and Metabolomic Modules with Risk of Kidney Disease Progression. J. Am. Soc. Nephrol. *35*, 923–935. https://doi.org/10.1681/ASN.0000000000000343.

69. Brinkkoetter, P.T., Bork, T., Salou, S., Liang, W., Mizi, A., Özel, C., Koehler, S., Hagmann, H.H., Ising, C., Kuczkowski, A., et al. (2019). Anaerobic Glycolysis Maintains the Glomerular Filtration Barrier Independent of Mitochondrial Metabolism and Dynamics. Cell Rep. *27*, 1551–1566.e5. https://doi.org/10.1016/j.celrep.2019.04.012.

70. Li, F., Fang, Y., Zhuang, Q., Cheng, M., Moronge, D., Jue, H., Meyuhas, O., Ding, X., Zhang, Z., Chen, J.-K., and Wu, H. (2022). Blocking ribosomal protein S6 phosphorylation inhibits podocyte hypertrophy and focal segmental glomerulosclerosis. Kidney Int. *102*, 121–135. https://doi.org/10.1016/j.kint.2022.02.037.

71. Yuan, Q., Miao, J., Yang, Q., Fang, L., Fang, Y., Ding, H., Zhou, Y., Jiang, L., Dai, C., Zen, K., et al. (2020). Role of pyruvate kinase M2-mediated

CellPress
OPEN ACCESS

metabolic reprogramming during podocyte differentiation. Cell Death Dis. *11*, 355. https://doi.org/10.1038/s41419-020-2481-5.

72. Qi, W., Keenan, H.A., Li, Q., Ishikado, A., Kannt, A., Sadowski, T., Yorek, M.A., Wu, I.-H., Lockhart, S., Coppey, L.J., et al. (2017). Pyruvate kinase M2 activation may protect against the progression of diabetic glomerular pathology and mitochondrial dysfunction. Nat. Med. *23*, 753–762. https://doi.org/10.1038/nm.4328.

73. Fu, J., Shinjo, T., Li, Q., St-Louis, R., Park, K., Yu, M.G., Yokomizo, H., Simao, F., Huang, Q., Wu, I.-H., and King, G.L. (2022). Regeneration of glomerular metabolism and function by podocyte pyruvate kinase M2 in diabetic nephropathy. JCI Insight *7*, e155260. https://doi.org/10.1172/jci.insight.155260.

74. Perico, L., Conti, S., Benigni, A., and Remuzzi, G. (2016). Podocyte-actin dynamics in health and disease. Nat. Rev. Nephrol. *12*, 692–710. https://doi.org/10.1038/NRNEPH.2016.127.

75. Schell, C., Rogg, M., Suhm, M., Helmstädter, M., Sellung, D., Yasuda-Yamahara, M., Kretz, O., Küttner, V., Suleiman, H., Kollipara, L., et al. (2017). The FERM protein EPB41L5 regulates actomyosin contractility and focal adhesion formation to maintain the kidney filtration barrier. Proc. Natl. Acad. Sci. USA *114*, E4621–E4630. https://doi.org/10.1073/PNAS.1617004114.

76. Rogg, M., Maier, J.I., Van Wymersch, C., Helmstädter, M., Sammarco, A., Lindenmeyer, M., Zareba, P., Montanez, E., Walz, G., Werner, M., et al. (2022). a-Parvin Defines a Specific Integrin Adhesome to Maintain the Glomerular Filtration Barrier. J. Am. Soc. Nephrol. *33*, 786–808. https://doi.org/10.1681/ASN.2021101319/-/DCSUPPLEMENTAL.

77. Lv, Z., Hu, M., Fan, M., Li, X., Lin, J., Zhen, J., Wang, Z., Jin, H., and Wang, R. (2018). Podocyte-specific Rac1 deficiency ameliorates podocyte damage and proteinuria in STZ-induced diabetic nephropathy in mice. Cell Death Dis. *9*, 342. https://doi.org/10.1038/S41419-018-0353-Z.

78. Clark, A.R., Marshall, J., Zhou, Y., Montesinos, M.S., Chen, H., Nguyen, L., Chen, F., and Greka, A. (2022). Single-Cell Transcriptomics Reveal Disrupted Kidney Filter Cell-Cell Interactions after Early and Selective. Am. J. Pathol. *192*, 281–294. https://doi.org/10.1016/J.AJPATH.2021.11.004.

79. Shi, S., Srivastava, S.P., Kanasaki, M., He, J., Kitada, M., Nagai, T., Nitta, K., Takagi, S., Kanasaki, K., and Koya, D. (2015). Interactions of DPP-4 and integrin β1 influences endothelial-to-mesenchymal transition. Kidney Int. *88*, 479–489. https://doi.org/10.1038/KI.2015.103.

80. Eun Lee, J., Kim, J.E., Lee, M.H., Song, H.K., Ghee, J.Y., Kang, Y.S., Min, H.S., Kim, H.W., Cha, J.J., Han, J.Y., et al. (2016). DA-1229, a dipeptidyl peptidase IV inhibitor, protects against renal injury by preventing podocyte damage in an animal model of progressive renal injury. Lab. Invest. *96*, 547–560. https://doi.org/10.1038/LABINVEST.2016.34.

81. Wexler, D.J., De Boer, I.H., Ghosh, A., Younes, N., Bebu, I., Inzucchi, S.E., McGill, J.B., Mudaliar, S., Schade, D., Steffes, M.W., et al. (2023). Comparative Effects of Glucose-Lowering Medications on Kidney Outcomes in Type 2 Diabetes: The GRADE Randomized Clinical Trial. JAMA Intern. Med. *183*, 705–714. https://doi.org/10.1001/JAMAINTERNMED.2023.1487.

82. Mosenzon, O., Leibowitz, G., Bhatt, D.L., Cahn, A., Hirshberg, B., Wei, C., Im, K., Rozenberg, A., Yanuv, I., Stahre, C., et al. (2017). Effect of Saxagliptin on Renal Outcomes in the SAVOR-TIMI 53 Trial. Diabetes Care *40*, 69–76. https://doi.org/10.2337/DC16-0621.

83. Groop, P.H., Cooper, M.E., Perkovic, V., Emser, A., Woerle, H.J., and Von Eynatten, M. (2013). Linagliptin lowers albuminuria on top of recommended standard treatment in patients with type 2 diabetes and renal dysfunction. Diabetes Care *36*, 3460–3468. https://doi.org/10.2337/DC13-0323.

84. Yoon, S.A., Han, B.G., Kim, S.G., Han, S.Y., Jo, Y.I., Jeong, K.H., Oh, K.H., Park, H.C., Park, S.H., Kang, S.W., et al. (2017). Efficacy, safety and albuminuria-reducing effect of gemigliptin in Korean type 2 diabetes patients with moderate to severe renal impairment: A 12-week, double-blind randomized study (the GUARD Study). Diabetes Obes. Metab. *19*, 590–598. https://doi.org/10.1111/DOM.12863.

85. He, L., Fan, Y., Zhang, Y., Tu, T., Zhang, Q., Yuan, F., and Cheng, C. (2022). Single-cell transcriptomic analysis reveals circadian rhythm disruption associated with poor prognosis and drug-resistance in lung adenocarcinoma. J. Pineal Res. *73*, e12803. https://doi.org/10.1111/JPI.12803.

86. Van den Berge, K., Roux de Bézieux, H., Street, K., Saelens, W., Cannoodt, R., Saeys, Y., Dudoit, S., and Clement, L. (2020). Trajectory-based differential expression analysis for single-cell sequencing data. Nat. Commun. *11*, 1201. https://doi.org/10.1038/S41467-020-14766-3.

87. Randles, M.J., Woolf, A.S., Huang, J.L., Byron, A., Humphries, J.D., Price, K.L., Kolatsi-Joannou, M., Collinson, S., Denny, T., Knight, D., et al. (2015). Genetic Background is a Key Determinant of Glomerular Extracellular Matrix Composition and Organization. J. Am. Soc. Nephrol. *26*, 3021–3034. https://doi.org/10.1681/ASN.2014040419.

88. Rogg, M., Maier, J.I., Dotzauer, R., Artelt, N., Kretz, O., Helmstädter, M., Abed, A., Sammarco, A., Sigle, A., Sellung, D., et al. (2021). SRGAP1 controls small rho GTPases to regulate podocyte foot process maintenance. J. Am. Soc. Nephrol. *32*, 563–579. https://doi.org/10.1681/ASN.2020081126/-/DCSUPPLEMENTAL.

89. Duez, H., and Staels, B. (2009). Rev-erb-alpha: an integrator of circadian rhythms and metabolism. J. Appl. Physiol. *107*, 1972–1980. https://doi.org/10.1152/JAPPLPHYSIOL.00570.2009.

90. Preston, R., Chrisp, R., Dudek, M., Morais, M.R.P.T., Tian, P., Williams, E., Naylor, R.W., Davenport, B., Pathiranage, D.R.J., Benson, E., et al. (2025). The glomerular circadian clock temporally regulates basement membrane dynamics and the podocyte glucocorticoid response. Kidney Int. *107*, 99–115. https://doi.org/10.1016/J.KINT.2024.10.016.

91. Wang, L., Tian, H., Wang, H., Mao, X., Luo, J., He, Q., Wen, P., Cao, H., Fang, L., Zhou, Y., et al. (2024). Disrupting circadian control of autophagy induces podocyte injury and proteinuria. Kidney Int. *105*, 1020–1034. https://doi.org/10.1016/J.KINT.2024.01.035.

92. Ansermet, C., Centeno, G., Nikolaeva, S., Maillard, M.P., Pradervand, S., and Firsov, D. (2019). The intrinsic circadian clock in podocytes controls glomerular filtration rate. Sci. Rep. *9*, 16089. https://doi.org/10.1038/S41598-019-52682-9.

93. Auerbach, B.J., FitzGerald, G.A., and Li, M. (2022). Tempo: an unsupervised Bayesian algorithm for circadian phase inference in single-cell transcriptomics. Nat. Commun. *13*, 6580. https://doi.org/10.1038/S41467-022-34185-W.

94. Sanfeliu-Redondo, D., Gibert-Ramos, A., and Gracia-Sancho, J. (2024). Cell senescence in liver diseases: pathological mechanism and theranostic opportunity. Nat. Rev. Gastroenterol. Hepatol. *21*, 477–492. https://doi.org/10.1038/s41575-024-00913-4.

95. Saul, D., Kosinsky, R.L., Atkinson, E.J., Doolittle, M.L., Zhang, X., LeBrasseur, N.K., Pignolo, R.J., Robbins, P.D., Niedernhofer, L.J., Ikeno, Y., et al. (2022). A new gene set identifies senescent cells and predicts senescence-associated pathways across tissues. Nat. Commun. *13*, 4827. https://doi.org/10.1038/s41467-022-32552-1.

96. Du, K., Umbaugh, D.S., Wang, L., Jun, J.H., Dutta, R.K., Oh, S.H., Ren, N., Zhang, Q., Ko, D.C., Ferreira, A., et al. (2025). Targeting senescent hepatocytes for treatment of metabolic dysfunction-associated steatotic liver disease and multi-organ dysfunction. Nat. Commun. *16*, 3038. https://doi.org/10.1038/s41467-025-57616-w.

97. Köhn-Gaone, J., Dwyer, B.J., Grzelak, C.A., Miller, G., Shackel, N.A., Ramm, G.A., McCaughan, G.W., Elsegood, C.L., Olynyk, J.K., and Tirnitz-Parker, J.E.E. (2016). Divergent Inflammatory, Fibrogenic, and Liver Progenitor Cell Dynamics in Two Common Mouse Models of Chronic Liver Injury. Am. J. Pathol. *186*, 1762. https://doi.org/10.1016/j.ajpath.2016.03.005.

98. Carlessi, R., Denisenko, E., Boslem, E., Köhn-Gaone, J., Main, N., Abu Bakar, N.D.B., Shirolkar, G.D., Jones, M., Beasley, A.B., Poppe, D., et al. (2023). Single-nucleus RNA sequencing of pre-malignant liver

reveals disease-associated hepatocyte state with HCC prognostic potential. Cell Genom. *3*, 100301. https://doi.org/10.1016/j.xgen.2023.100301.

99. Vesting, A.J., Jais, A., Klemm, P., Steuernagel, L., Wienand, P., Fog-Tonnesen, M., Hvid, H., Schumacher, A.-L., Kukat, C., Nolte, H., et al. (2022). NIK/MAP3K14 in hepatocytes orchestrates NASH to hepatocellular carcinoma progression via JAK2/STAT5 inhibition. Mol. Metab. *66*, 101626. https://doi.org/10.1016/j.molmet.2022.101626.

100. Cacho-Navas, C., López-Pujante, C., Reglero-Real, N., Colás-Algora, N., Cuervo, A., Conesa, J.J., Barroso, S., de Rivas, G., Ciordia, S., Paradela, A., et al. (2024). ICAM-1 nanoclusters regulate hepatic epithelial cell polarity by leukocyte adhesion-independent control of apical actomyosin. eLife *12*. https://doi.org/10.7554/eLife.89261.

101. Zhang, S., Guo, F., Yu, M., Yang, X., Yao, Z., Li, Q., Wei, Z., Feng, K., Zeng, P., Zhao, D., et al. (2020). Reduced Nogo expression inhibits diet-induced metabolic disorders by regulating ChREBP and insulin activity. J. Hepatol. *73*, 1482–1495. https://doi.org/10.1016/j.jhep.2020.07.034.

102. Hikita, H., Takehara, T., Shimizu, S., Kodama, T., Li, W., Miyagi, T., Hosui, A., Ishida, H., Ohkawa, K., Kanto, T., et al. (2009). Mcl-1 and Bcl-xL cooperatively maintain integrity of hepatocytes in developing and adult murine liver. Hepatology *50*, 1217–1226. https://doi.org/10.1002/hep.23126.

103. Vickers, S.D., Shumar, S.A., Saporito, D.C., Kunovac, A., Hathaway, Q.A., Mintmier, B., King, J.A., King, R.D., Rajendran, V.M., Infante, A.M., et al. (2023). NUDT7 regulates total hepatic CoA levels and the composition of the intestinal bile acid pool in male mice fed a Western diet. J. Biol. Chem. *299*, 102745. https://doi.org/10.1016/j.jbc.2022.102745.

104. Xu, C., Li, H., and Tang, C.-K. (2022). Sterol carrier protein 2 in lipid metabolism and non-alcoholic fatty liver disease: Pathophysiology, molecular biology, and potential clinical implications. Metabolism *131*, 155180. https://doi.org/10.1016/j.metabol.2022.155180.

105. Schweitzer, G.G., Chen, Z., Gan, C., McCommis, K.S., Soufi, N., Chrast, R., Mitra, M.S., Yang, K., Gross, R.W., and Finck, B.N. (2015). Liver-specific loss of lipin-1-mediated phosphatidic acid phosphatase activity does not mitigate intrahepatic TG accumulation in mice. J. Lipid Res. *56*, 848–858. https://doi.org/10.1194/jlr.M055962.

106. Lam, P., Soroka, C.J., and Boyer, J.L. (2010). The bile salt export pump: clinical and experimental aspects of genetic and acquired cholestatic liver disease. Semin. Liver Dis. *30*, 125–133. https://doi.org/10.1055/s-0030-1253222.

107. Dawson, P.A., and Setchell, K.D.R. (2017). Will the real bile acid sulfotransferase please stand up? Identification of Sult2a8 as a major hepatic bile acid sulfonating enzyme in mice. J. Lipid Res. *58*, 1033–1035. https://doi.org/10.1194/JLR.C077420.

108. Bergeron, F., Otto, A., Blache, P., Day, R., Denoroy, L., Brandsch, R., and Bataille, D. (1998). Molecular cloning and tissue distribution of rat sarcosine dehydrogenase. Eur. J. Biochem. *257*, 556–561. https://doi.org/10.1046/j.1432-1327.1998.2570556.x.

109. García-Suástegui, W.A., Ramos-Chávez, L.A., Rubio-Osornio, M., Calvillo-Velasco, M., Atzin-Méndez, J.A., Guevara, J., and Silva-Adaya, D. (2017). The Role of CYP2E1 in the Drug Metabolism or Bioactivation in the Brain. Oxid. Med. Cell. Longev. *2017*, 4680732. https://doi.org/10.1155/2017/4680732.

110. Clemmons, D.R. (2004). The relative roles of growth hormone and IGF-1 in controlling insulin sensitivity. J. Clin. Investig. *113*, 25–27. https://doi.org/10.1172/JCI20660.

111. Wang, K., Chan, Y.C., So, P.K., Liu, X., Feng, L., Cheung, W.T., Lee, S.S.T., and Au, S.W.N. (2021). Structure of mouse cytosolic sulfotransferase SULT2A8 provides insight into sulfonation of 7α-hydroxyl bile acids. J. Lipid Res. *62*, 100074. https://doi.org/10.1016/J.JLR.2021.100074.

112. Izgi, H., Han, D., Isildak, U., Huang, S., Kocabiyik, E., Khaitovich, P., Somel, M., and Dönertaş, H.M. (2022). Inter-tissue convergence of gene expression during ageing suggests age-related loss of tissue and cellular identity. eLife *11*, e68048. https://doi.org/10.7554/eLife.68048.

113. Yang, J.-H., Hayano, M., Griffin, P.T., Amorim, J.A., Bonkowski, M.S., Apostolides, J.K., Salfati, E.L., Blanchette, M., Munding, E.M., Bhakta, M., et al. (2023). Loss of epigenetic information as a cause of mammalian aging. Cell *186*, 305–326.e27. https://doi.org/10.1016/j.cell.2022.12.027.

114. MacHado, M.V., and Diehl, A.M. (2016). Pathogenesis of Nonalcoholic Steatohepatitis. Gastroenterology *150*, 1769–1777. https://doi.org/10.1053/J.GASTRO.2016.02.066.

115. Matsuda, J., Maier, M., Aoudjit, L., Baldwin, C., and Takano, T. (2020). ARHGEF7 (β-PIX) Is Required for the Maintenance of Podocyte Architecture and Glomerular Function. J. Am. Soc. Nephrol. *31*, 996–1008. https://doi.org/10.1681/ASN.2019090982.

116. Wang, Y., Eng, D.G., Kaverina, N.V., Loretz, C.J., Koirala, A., Akilesh, S., Pippin, J.W., and Shankland, S.J. (2020). Global transcriptomic changes occur in aged mouse podocytes. Kidney Int. *98*, 1160–1173. https://doi.org/10.1016/j.kint.2020.05.052.

117. Schermer, B., Bartels, V., Frommolt, P., Habermann, B., Braun, F., Schultze, J.L., Roodbergen, M., Hoeijmakers, J.H., Schumacher, B., Nürnberg, P., et al. (2013). Transcriptional profiling reveals progeroid Ercc1(-/Δ) mice as a model system for glomerular aging. BMC Genom. *14*, 559. https://doi.org/10.1186/1471-2164-14-559.

118. Zhong, Y., Lee, K., Deng, Y., Ma, Y., Chen, Y., Li, X., Wei, C., Yang, S., Wang, T., Wong, N.J., et al. (2019). Arctigenin attenuates diabetic kidney disease through the activation of PP2A in podocytes. Nat. Commun. *10*, 4523. https://doi.org/10.1038/s41467-019-12433-w.

119. Hinder, L.M., Park, M., Rumora, A.E., Hur, J., Eichinger, F., Pennathur, S., Kretzler, M., Brosius, F.C., and Feldman, E.L. (2017). Comparative RNA-Seq transcriptome analyses reveal distinct metabolic pathways in diabetic nerve and kidney disease. J. Cell Mol. Med. *21*, 2140–2152. https://doi.org/10.1111/jcmm.13136.

120. Long, J., Badal, S.S., Ye, Z., Wang, Y., Ayanga, B.A., Galvan, D.L., Green, N.H., Chang, B.H., Overbeek, P.A., and Danesh, F.R. (2016). Long noncoding RNA Tug1 regulates mitochondrial bioenergetics in diabetic nephropathy. J. Clin. Investig. *126*, 4205–4218. https://doi.org/10.1172/JCI87927.

121. Fu, J., Wei, C., Lee, K., Zhang, W., He, W., Chuang, P., Liu, Z., and He, J.C. (2016). Comparison of Glomerular and Podocyte mRNA Profiles in Streptozotocin-Induced Diabetes. J. Am. Soc. Nephrol. *27*, 1006–1014. https://doi.org/10.1681/ASN.2015040421.

122. Chittka, D., Banas, B., Lennartz, L., Putz, F.J., Eidenschink, K., Beck, S., Stempfl, T., Moehle, C., Reichelt-Wurm, S., and Banas, M.C. (2018). Long-term expression of glomerular genes in diabetic nephropathy. Nephrol. Dial. Transplant. *33*, 1533–1544. https://doi.org/10.1093/ndt/gfx359.

123. Reiniger, N., Lau, K., McCalla, D., Eby, B., Cheng, B., Lu, Y., Qu, W., Quadri, N., Ananthakrishnan, R., Furmansky, M., et al. (2010). Deletion of the receptor for advanced glycation end products reduces glomerulosclerosis and preserves renal function in the diabetic OVE26 mouse. Diabetes *59*, 2043–2054. https://doi.org/10.2337/db09-1766.

124. Sugahara, M., Tanaka, S., Tanaka, T., Saito, H., Ishimoto, Y., Wakashima, T., Ueda, M., Fukui, K., Shimizu, A., Inagi, R., et al. (2020). Prolyl Hydroxylase Domain Inhibitor Protects against Metabolic Disorders and Associated Kidney Disease in Obese Type 2 Diabetic Mice. J. Am. Soc. Nephrol. *31*, 560–577. https://doi.org/10.1681/ASN.2019060582.

125. Xu, J., Zheng, S., Kralik, P.M., Krishnan, L., Huang, H., Hoying, J.B., Cai, L., Carlson, E.C., Tan, Y., and Epstein, P.N. (2016). Diabetes Induced Changes in Podocyte Morphology and Gene Expression Evaluated Using GFP Transgenic Podocytes. Int. J. Biol. Sci. *12*, 210–218. https://doi.org/10.7150/ijbs.13057.

126. Cao, A., Li, J., Asadi, M., Basgen, J.M., Zhu, B., Yi, Z., Jiang, S., Doke, T., El Shamy, O., Patel, N., et al. (2021). DACH1 protects podocytes from experimental diabetic injury and modulates PTIP-H3K4Me3 activity. J. Clin. Investig. *131*, e141279. https://doi.org/10.1172/JCI141279.

127. Naudin, C., Smith, B., Bond, D.R., Dun, M.D., Scott, R.J., Ashman, L.K., Weidenhofer, J., and Roselli, S. (2017). Characterization of the early molecular changes in the glomeruli of Cd151 -/- mice highlights induction of mindin and MMP-10. Sci. Rep. *7*, 15987. https://doi.org/10.1038/s41598-017-15993-3.

128. Inoue, K., Gan, G., Ciarleglio, M., Zhang, Y., Tian, X., Pedigo, C.E., Cavanaugh, C., Tate, J., Wang, Y., Cross, E., et al. (2019). Podocyte histone deacetylase activity regulates murine and human glomerular diseases. J. Clin. Investig. *129*, 1295–1313. https://doi.org/10.1172/JCI124030.

129. Guo, Y., Pace, J., Li, Z., Ma'ayan, A., Wang, Z., Revelo, M.P., Chen, E., Gu, X., Attalah, A., Yang, Y., et al. (2018). Podocyte-Specific Induction of Krüppel-Like Factor 15 Restores Differentiation Markers and Attenuates Kidney Injury in Proteinuric Kidney Disease. J. Am. Soc. Nephrol. *29*, 2529–2545. https://doi.org/10.1681/ASN.2018030324.

130. Mesnard, L., Cathelin, D., Vandermeersch, S., Rafat, C., Luque, Y., Sohier, J., Nochy, D., Garcon, L., Callard, P., Jouanneau, C., et al. (2014). Genetic background-dependent thrombotic microangiopathy is related to vascular endothelial growth factor receptor 2 signaling during anti-glomerular basement membrane glomerulonephritis in mice. Am. J. Pathol. *184*, 2438–2449. https://doi.org/10.1016/j.ajpath.2014.05.020.

131. Bender, S., Ayala, M.V., Bonaud, A., Javaugue, V., Carrion, C., Oblet, C., Rinsant, A., Kaaki, S., Oruc, Z., Boyer, F., et al. (2020). Immunoglobulin light-chain toxicity in a mouse model of monoclonal immunoglobulin light-chain deposition disease. Blood *136*, 1645–1656. https://doi.org/10.1182/blood.2020005980.

132. Sidhom, E.-H., Kim, C., Kost-Alimova, M., Ting, M.T., Keller, K., Avila-Pacheco, J., Watts, A.J., Vernon, K.A., Marshall, J.L., Reyes-Bricio, E., et al. (2021). Targeting a Braf/Mapk pathway rescues podocyte lipid peroxidation in CoQ-deficiency kidney disease. J. Clin. Investig. *131*, e141380. https://doi.org/10.1172/JCI141380.

133. Talyan, S., Filipów, S., Ignarski, M., Smieszek, M., Chen, H., Kühne, L., Butt, L., Göbel, H., Hoyer-Allo, K.J.R., Koehler, F.C., et al. (2021). CALINCA-A Novel Pipeline for the Identification of lncRNAs in Podocyte Disease. Cells *10*, 692. https://doi.org/10.3390/CELLS10030692.

134. Potter, A.S., Drake, K., Brunskill, E.W., and Potter, S.S. (2019). A bigenic mouse model of FSGS reveals perturbed pathways in podocytes, mesangial cells and endothelial cells. PLoS One *14*, e0216261. https://doi.org/10.1371/journal.pone.0216261.

135. Wei, C., Banu, K., Garzon, F., Basgen, J.M., Philippe, N., Yi, Z., Liu, R., Choudhuri, J., Fribourg, M., Liu, T., et al. (2018). SHROOM3-FYN Interaction Regulates Nephrin Phosphorylation and Affects Albuminuria in Allografts. J. Am. Soc. Nephrol. *29*, 2641. https://doi.org/10.1681/ASN.2018060573.

136. Brunskill, E.W., and Potter, S.S. (2015). Pathogenic pathways are activated in each major cell type of the glomerulus in the Cd2ap mutant mouse model of focal segmental glomerulosclerosis. BMC Nephrol. *16*, 71. https://doi.org/10.1186/s12882-015-0063-z.

137. Ettou, S., Jung, Y.L., Miyoshi, T., Jain, D., Hiratsuka, K., Schumacher, V., Taglienti, M.E., Morizane, R., Park, P.J., and Kreidberg, J.A. (2020). Epigenetic transcriptional reprogramming by WT1 mediates a repair response during podocyte injury. Sci. Adv. *6*, eabb5460. https://doi.org/10.1126/sciadv.abb5460.

138. Okabe, M., Motojima, M., Miyazaki, Y., Pastan, I., Yokoo, T., and Matsusaka, T. (2019). Global polysome analysis of normal and injured podocytes. Am. J. Physiol. Renal Physiol. *316*, F241–F252. https://doi.org/10.1152/ajprenal.00115.2018.

139. Ratelade, J., Arrondel, C., Hamard, G., Garbay, S., Harvey, S., Biebuyck, N., Schulz, H., Hastie, N., Pontoglio, M., Gubler, M.-C., et al. (2010). A murine model of Denys-Drash syndrome reveals novel transcriptional targets of WT1 in podocytes. Hum. Mol. Genet. *19*, 1–15. https://doi.org/10.1093/hmg/ddp462.

140. Yang, J., Zhang, D., Motojima, M., Kume, T., Hou, Q., Pan, Y., Duan, A., Zhang, M., Jiang, S., Hou, J., et al. (2021). Super-Enhancer-Associated Transcription Factors Maintain Transcriptional Regulation in Mature Podocytes. J. Am. Soc. Nephrol. *32*, 1323–1337. https://doi.org/10.1681/ASN.2020081177.

141. Lefevre, G.M., Patel, S.R., Kim, D., Tessarollo, L., and Dressler, G.R. (2010). Altering a histone H3K4 methylation pathway in glomerular podocytes promotes a chronic disease phenotype. PLoS Genet. *6*, e1001142. https://doi.org/10.1371/journal.pgen.1001142.

142. Khanova, E., Wu, R., Wang, W., Yan, R., Chen, Y., French, S.W., Llorente, C., Pan, S.Q., Yang, Q., Li, Y., et al. (2018). Pyroptosis by caspase11/4-gasdermin-D pathway in alcoholic hepatitis in mice and patients. Hepatology *67*, 1737–1753. https://doi.org/10.1002/hep.29645.

143. Tsuchida, T., Lee, Y.A., Fujiwara, N., Ybanez, M., Allen, B., Martins, S., Fiel, M.I., Goossens, N., Chou, H.-I., Hoshida, Y., and Friedman, S.L. (2018). A simple diet- and chemical-induced murine NASH model with rapid progression of steatohepatitis, fibrosis and liver cancer. J. Hepatol. *69*, 385–395. https://doi.org/10.1016/j.jhep.2018.03.011.

144. Furuya, S., Argemi, J., Uehara, T., Katou, Y., Fouts, D.E., Schnabl, B., Dubuquoy, L., Belorkar, A., Vadigepalli, R., Kono, H., et al. (2020). A Novel Mouse Model of Acute-on-Chronic Cholestatic Alcoholic Liver Disease: A Systems Biology Comparison With Human Alcoholic Hepatitis. Alcohol Clin. Exp. Res. *44*, 87–101. https://doi.org/10.1111/acer.14234.

145. Umezu, T., Tsuneyama, K., Kanekura, K., Hayakawa, M., Tanahashi, T., Kawano, M., Taguchi, Y.-H., Toyoda, H., Tamori, A., Kuroda, M., and Murakami, Y. (2020). Comprehensive analysis of liver and blood miRNA in precancerous conditions. Sci. Rep. *10*, 21766. https://doi.org/10.1038/s41598-020-78500-1.

146. Li, X., Wang, Z., and Klaunig, J.E. (2019). The effects of perfluorooctanoate on high fat diet induced non-alcoholic fatty liver disease in mice. Toxicology *416*, 1–14. https://doi.org/10.1016/j.tox.2019.01.017.

147. Lewis, K., Valanejad, L., Cast, A., Wright, M., Wei, C., Iakova, P., Stock, L., Karns, R., Timchenko, L., and Timchenko, N. (2017). RNA Binding Protein CUGBP1 Inhibits Liver Cancer in a Phosphorylation-Dependent Manner. Mol. Cell Biol. *37*, e00128. https://doi.org/10.1128/MCB.00128-17.

148. Bird, T.G., M ller, M., Boulter, L., Vincent, D.F., Ridgway, R.A., Lopez-Guadamillas, E., Lu, W.Y., Jamieson, T., Govaere, O., Campbell, A.D., et al. (2018). TGFβ inhibition restores a regenerative response in acute liver injury by suppressing paracrine senescence. Sci. Transl. Med. *10*. https://doi.org/10.1126/SCITRANSLMED.AAN1230.

149. Zhang, G., Byun, H.R., Ying, Z., Blencowe, M., Zhao, Y., Hong, J., Shu, L., Chella Krishnan, K., Gomez-Pinilla, F., and Yang, X. (2020). Differential metabolic and multi-tissue transcriptomic responses to fructose consumption among genetically diverse mice. Biochim. Biophys. Acta. Mol. Basis Dis. *1866*, 165569. https://doi.org/10.1016/j.bbadis.2019.165569.

150. Magee, N., Ahamed, F., Eppler, N., Jones, E., Ghosh, P., He, L., and Zhang, Y. (2022). Hepatic transcriptome profiling reveals early signatures associated with disease transition from non-alcoholic steatosis to steatohepatitis. Liver Res. *6*, 238–250. https://doi.org/10.1016/j.livres.2022.11.001.

151. Bates, J., Vijayakumar, A., Ghoshal, S., Marchand, B., Yi, S., Kornyeyev, D., Zagorska, A., Hollenback, D., Walker, K., Liu, K., et al. (2020). Acetyl-CoA carboxylase inhibition disrupts metabolic reprogramming during hepatic stellate cell activation. J. Hepatol. *73*, 896–905. https://doi.org/10.1016/j.jhep.2020.04.037.

152. Sun, X., Seidman, J.S., Zhao, P., Troutman, T.D., Spann, N.J., Que, X., Zhou, F., Liao, Z., Pasillas, M., Yang, X., et al. (2020). Neutralization of Oxidized Phospholipids Ameliorates Non-alcoholic Steatohepatitis. Cell Metab. *31*, 189–206.e8. https://doi.org/10.1016/j.cmet.2019.10.014.

153. Heintz, M.M., McRee, R., Kumar, R., and Baldwin, W.S. (2020). Gender differences in diet-induced steatotic disease in Cyp2b-null mice. PLoS One *15*, e0229896. https://doi.org/10.1371/journal.pone.0229896.

154. Middleton, S.A., Rajpal, N., Cutler, L., Mander, P., Rioja, I., Prinjha, R.K., Rajpal, D., Agarwal, P., and Kumar, V. (2018). BET Inhibition Improves

NASH and Liver Fibrosis. Sci. Rep. *8*, 17257. https://doi.org/10.1038/s41598-018-35653-4.

155. Schaum, N., Lehallier, B., Hahn, O., Pálovics, R., Hosseinzadeh, S., Lee, S.E., Sit, R., Lee, D.P., Losada, P.M., Zardeneta, M.E., et al. (2020). Ageing hallmarks exhibit organ-specific temporal signatures. Nature *583*, 596–602. https://doi.org/10.1038/S41586-020-2499-Y.

156. Tabula Muris Consortium; Overall coordination; Logistical coordination; Organ collection and processing; Library preparation and sequencing; Computational data analysis; Cell type annotation; Writing group; Supplemental text writing group; Principal investigators (2018). Single-cell transcriptomics of 20 mouse organs creates a Tabula Muris. Nature *562*, 367–372. https://doi.org/10.1038/S41586-018-0590-4.

157. Butt, L., Unnersjö-Jess, D., Höhne, M., Schermer, B., Edwards, A., and Benzing, T. (2021). A mathematical estimation of the physical forces driving podocyte detachment. Kidney Int. *100*, 1054–1062. https://doi.org/10.1016/j.kint.2021.06.040.

158. Schneider, C.A., Rasband, W.S., and Eliceiri, K.W. (2012). NIH Image to ImageJ: 25 years of image analysis. Nat. Methods *9*, 671–675. https://doi.org/10.1038/nmeth.2089.

159. Bankhead, P., Loughrey, M.B., Fernández, J.A., Dombrowski, Y., McArt, D.G., Dunne, P.D., McQuaid, S., Gray, R.T., Murray, L.J., Coleman, H.G., et al. (2017). QuPath: Open source software for digital pathology image analysis. Sci. Rep. *7*, 16878. https://doi.org/10.1038/s41598-017-17204-5.

160. Goldsborough, T., O'Callaghan, A., Inglis, F., Leplat, L., Filby, A., Bilen, H., and Bankhead, P. (2024). A novel channel invariant architecture for the segmentation of cells and nuclei in multiplexed images using InstanSeg. Preprint at bioRxiv. https://doi.org/10.1101/2024.09.04.611150.

161. Schindelin, J., Arganda-Carreras, I., Frise, E., Kaynig, V., Longair, M., Pietzsch, T., Preibisch, S., Rueden, C., Saalfeld, S., Schmid, B., et al. (2012). Fiji: an open-source platform for biological-image analysis. Nat. Methods *9*, 676–682. https://doi.org/10.1038/nmeth.2019.

162. Ewels, P., Magnusson, M., Lundin, S., and Käller, M. (2016). MultiQC: summarize analysis results for multiple tools and samples in a single report. Bioinformatics *32*, 3047–3048. https://doi.org/10.1093/BIOINFORMATICS/BTW354.

163. Durinck, S., Spellman, P.T., Birney, E., and Huber, W. (2009). Mapping identifiers for the integration of genomic datasets with the R/Bioconductor package biomaRt. Nat. Protoc. *4*, 1184–1191. https://doi.org/10.1038/NPROT.2009.97.

164. Durinck, S., Moreau, Y., Kasprzyk, A., Davis, S., De Moor, B., Brazma, A., and Huber, W. (2005). BioMart and Bioconductor: a powerful link between biological databases and microarray data analysis. Bioinformatics *21*, 3439–3440. https://doi.org/10.1093/BIOINFORMATICS/BTI525.

165. Dobin, A., Davis, C.A., Schlesinger, F., Drenkow, J., Zaleski, C., Jha, S., Batut, P., Chaisson, M., and Gingeras, T.R. (2013). STAR: ultrafast universal RNA-seq aligner. Bioinformatics *29*, 15–21. https://doi.org/10.1093/BIOINFORMATICS/BTS635.

166. Yang, S., Corbett, S.E., Koga, Y., Wang, Z., Johnson, W.E., Yajima, M., and Campbell, J.D. (2020). Decontamination of ambient RNA in single-cell RNA-seq with DecontX. Genome Biol. *21*, 57. https://doi.org/10.1186/S13059-020-1950-6.

167. Hao, Y., Hao, S., Andersen-Nissen, E., Mauck, W.M., Zheng, S., Butler, A., Lee, M.J., Wilk, A.J., Darby, C., Zager, M., et al. (2021). Integrated analysis of multimodal single-cell data. Cell *184*, 3573–3587.e29. https://doi.org/10.1016/J.CELL.2021.04.048.

168. Li, H., and Durbin, R. (2009). Fast and accurate short read alignment with Burrows-Wheeler transform. Bioinformatics *25*, 1754–1760. https://doi.org/10.1093/bioinformatics/btp324.

169. Gaspar, J.M. (2020). Genrich: detecting sites of genomic enrichment. Github. https://github.com/jsh58/Genrich.

170. Pebesma, E. (2018). Simple Features for R: Standardized Support for Spatial Vector Data. R J. *10*, 439. https://doi.org/10.32614/RJ-2018-009.

171. Zhang, X., Smits, A.H., van Tilburg, G.B., Ovaa, H., Huber, W., and Vermeulen, M. (2018). Proteome-wide identification of ubiquitin interactions using UbIA-MS. Nat. Protoc. *13*, 530–550. https://doi.org/10.1038/NPROT.2017.147.

172. Carvalho, B.S., and Irizarry, R.A. (2010). A framework for oligonucleotide microarray preprocessing. Bioinformatics *26*, 2363–2367. https://doi.org/10.1093/BIOINFORMATICS/BTQ431.

173. Love, M.I., Huber, W., and Anders, S. (2014). Moderated estimation of fold change and dispersion for RNA-seq data with DESeq2. Genome Biol. *15*, 550. https://doi.org/10.1186/S13059-014-0550-8.

174. Yu, G., Wang, L.G., Han, Y., and He, Q.Y. (2012). clusterProfiler: an R package for comparing biological themes among gene clusters. OMICS *16*, 284–287. https://doi.org/10.1089/OMI.2011.0118.

175. Cao, J., Spielmann, M., Qiu, X., Huang, X., Ibrahim, D.M., Hill, A.J., Zhang, F., Mundlos, S., Christiansen, L., Steemers, F.J., et al. (2019). The single-cell transcriptional landscape of mammalian organogenesis. Nature *566*, 496–502. https://doi.org/10.1038/s41586-019-0969-x.

176. Magno, R., and Maia, A.-T. (2020). gwasrapidd: an R package to query, download and wrangle GWAS catalog data. Bioinformatics *36*, 649–650. https://doi.org/10.1093/bioinformatics/btz605.

177. Bentsen, M., Goymann, P., Schultheis, H., Klee, K., Petrova, A., Wiegandt, R., Fust, A., Preussner, J., Kuenne, C., Braun, T., et al. (2020). ATAC-seq footprinting unravels kinetics of transcription factor binding during zygotic genome activation. Nat. Commun. *11*, 4267. https://doi.org/10.1038/S41467-020-18035-1.

178. Grant, C.E., Bailey, T.L., and Noble, W.S. (2011). FIMO: scanning for occurrences of a given motif. Bioinformatics *27*, 1017–1018. https://doi.org/10.1093/BIOINFORMATICS/BTR064.

179. Sikora-Wohlfeld, W., Ackermann, M., Christodoulou, E.G., Singaravelu, K., and Beyer, A. (2013). Assessing computational methods for transcription factor target gene identification based on ChIP-seq data. PLoS Comput. Biol. *9*, e1003342. https://doi.org/10.1371/JOURNAL.PCBI.1003342.

180. Wu, G., Anafi, R.C., Hughes, M.E., Kornacker, K., and Hogenesch, J.B. (2016). MetaCycle: an integrated R package to evaluate periodicity in large scale data. Bioinformatics *32*, 3351–3353. https://doi.org/10.1093/BIOINFORMATICS/BTW405.

181. Kristensen, K., Nielsen, A., Berg, C.W., Skaug, H., and Bell, B.M. (2016). TMB: Automatic Differentiation and Laplace Approximation. J. Stat. Softw. *70*. https://doi.org/10.18637/jss.v070.i05.

182. Germain, P.-L., Lun, A., Garcia Meixide, C., Macnair, W., and Robinson, M.D. (2021). Doublet identification in single-cell sequencing data using scDblFinder. F1000Res. *10*, 979. https://doi.org/10.12688/f1000research.73600.2.

183. Tröder, S.E., Ebert, L.K., Butt, L., Assenmacher, S., Schermer, B., and Zevnik, B. (2018). An optimized electroporation approach for efficient CRISPR/Cas9 genome editing in murine zygotes. PLoS One *13*, e0196891. https://doi.org/10.1371/JOURNAL.PONE.0196891.

184. Schumacher, V.A., Schlötzer-Schrehardt, U., Karumanchi, S.A., Shi, X., Zaia, J., Jeruschke, S., Zhang, D., Pavenstädt, H., Drenckhan, A., Amann, K., et al. (2011). WT1-dependent sulfatase expression maintains the normal glomerular filtration barrier. J. Am. Soc. Nephrol. *22*, 1286–1296. https://doi.org/10.1681/ASN.2010080860.

185. Moeller, M.J., Sanden, S.K., Soofi, A., Wiggins, R.C., and Holzman, L.B. (2003). Podocyte-specific expression of cre recombinase in transgenic mice. Genesis *35*, 39–42. https://doi.org/10.1002/GENE.10164.

186. Moh, A., Iwamoto, Y., Chai, G.-X., Zhang, S.S.-M., Kano, A., Yang, D.D., Zhang, W., Wang, J., Jacoby, J.J., Gao, B., et al. (2007). Role of STAT3 in liver regeneration: survival, DNA synthesis, inflammatory reaction and liver mass recovery. Lab. Invest. *87*, 1018–1028. https://doi.org/10.1038/labinvest.3700630.

CellPress
OPEN ACCESS

187. Hahn, M., Macht, A., Waisman, A., and Hövelmeyer, N. (2016). NF-κB-inducing kinase is essential for B-cell maintenance in mice. Eur. J. Immunol. *46*, 732–741. https://doi.org/10.1002/eji.201546081.

188. Unnersjö-Jess, D., Butt, L., Höhne, M., Sergei, G., Fatehi, A., Witasp, A., Wernerson, A., Patrakka, J., Hoyer, P.F., Blom, H., et al. (2023). Deep learning-based segmentation and quantification of podocyte foot process morphology suggests differential patterns of foot process effacement across kidney pathologies. Kidney Int. *103*, 1120–1130. https://doi.org/10.1016/j.kint.2023.03.013.

189. Andrews, S. (2010). FastQC: A Quality Control Tool for High Throughput Sequence Data. http://www.bioinformatics.babraham.ac.uk/projects/fastqc/.

190. Baldarelli, R.M., Smith, C.L., Ringwald, M., Richardson, J.E., Bult, C.J., Mouse Genome Informatics Group; Begley, D.A., Bello, S.M., Christie, K., and Finger, J.H. (2024). Mouse Genome Informatics: an integrated knowledgebase system for the laboratory mouse. Genetics *227*, iyae031. https://doi.org/10.1093/GENETICS/IYAE031.

191. Liberzon, A., Birger, C., Thorvaldsdóttir, H., Ghandi, M., Mesirov, J.P., and Tamayo, P. (2015). The Molecular Signatures Database (MSigDB) hallmark gene set collection. Cell Syst. *1*, 417–425. https://doi.org/10.1016/J.CELS.2015.12.004.

192. Bailey, T.L., Johnson, J., Grant, C.E., and Noble, W.S. (2015). The MEME Suite. Nucleic Acids Res. *43*, W39–W49. https://doi.org/10.1093/nar/gkv416.

STAR★METHODS

KEY RESOURCES TABLE

REAGENT or RESOURCE	SOURCE	IDENTIFIER
Antibodies		
Guinea pig polyclonal anti-Nephrin	Fitzgerald Industries International	Cat# 20R-NP002; RRID:AB_1288100
Sheep polyclonal anti-Nephrin	R&D Systems	Cat# AF4269; RRID:AB_2154851
Rabbit polyclonal anti-THSD7A	Sigma	Cat# HPA000923; RRID:AB_1080271
Mouse monoclonal anti-Claudin-5	Thermo Fisher Scientific	Cat#3 5–2500; RRID:AB_2533200
Rabbit polyclonal anti-EPHB1 antibody	Abnova	Cat# PAB3018; RRID:AB_1574714
Rabbit monoclonal anti-Rev-Erbα (Nr1d1) antibody	Cell Signaling Technology	Cat# 13418; RRID:AB_2630359
Goat polyclonal anti-WT1 antibody	R&D Systems	Cat# AF5729; RRID:AB_2216239
Rabbit polyclonal anti-PKM antibody	Proteintech	Cat# 25659-1-AP; RRID:AB_2880181
Rabbit monoclonal anti-Phospho-S6 Ribosomal Protein antibody	Cell Signaling Technology	Cat# 4858; RRID:AB_916156
Rabbit polyclonal anti-CYP7A1 antibody	Thermo Fisher Scientific	Cat# PA5-100892; RRID:AB_2850387
AffiniPure Goat Anti-Guinea Pig IgG (H + L)	Jackson ImmunoResearch Labs	Cat# 106-005-003; RRID:AB_2337394
AffiniPure Donkey Anti-Rabbit IgG (H + L)	Jackson ImmunoResearch Labs	Cat# 711-005-152; RRID:AB_2340585
AffiniPure Goat Anti-Mouse IgG (H + L)	Jackson ImmunoResearch Labs	Cat# 115-005-003; RRID:AB_2338447
Donkey anti-Goat IgG (H + L) Cross-Adsorbed Secondary Antibody, Alexa Fluor™ 488	Thermo Fisher Scientific	Cat# A11055; RRID:AB_2534102
Goat polyclonal anti-Mouse Albumin Antibody	Bethyl Laboratories	Cat# A90-134A; RRID:AB_2891982
Goat polyclonal anti-Mouse Albumin Antibody HRP Conjugated	Bethyl Laboratories	Cat# A90-134P; RRID:AB_67127
Biological samples		
Human kidney FSGS and MCD biopsies	ForMe registry, ClinicalTrials.gov identifier NCT039499728	patient ID 29-10282
Chemicals, peptides, and recombinant proteins		
EF Control to NASH diet (CD)	ssniff Spezialdiäten GmbH	Cat# E15767-040
EF NASH diet (HFD-NASH)	ssniff Spezialdiäten GmbH	Cat# E15766-340
Choline free, lard, 1% cholesterol diet (CDAA-NASH)	ssniff Spezialdiäten GmbH	Cat# S2159-E042
Control diet (CD or NCD)	ssniff Spezialdiäten GmbH	Cat# R/M-H
Dynabeads™ M-450 Tosylactivated	Invitrogen	Cat# 14013
Pronase E	Sigma	Cat# P6911
Collagenase type II	Worthington	Cat# LS004176
Dnase I	Applichem	Cat# A3778,0050
EDTA-free protease inhibitors	Roche	Cat# 5056489001
4′,6-diamidino-2-phenylindole (DAPI)	Sigma	Cat# D9542
Murine RNase inhibitor	New England Biolabs GmbH	Cat# M0314L
Bovine serum albumin (BSA)	Carl Roth	Cat# 3737.2
Fructose	VWR	Cat# USBIF8000
1-Thioglycerol	Sigma	Cat# M6145
Normal donkey serum	Jackson ImmunoResearch	Cat# 17-000-121
Glutaraldehyde	Electron Microscopy Sciences	50-262-23

(Continued on next page)

Continued		
REAGENT or RESOURCE	SOURCE	IDENTIFIER
Critical commercial assays		
Nuclei Isolation Kit: Nuclei EZ Prep	Sigma	Cat# NUC101-1KT
Illumina Tagment DNA Enzyme and Buffer Small Kit	Illumina	Cat# 20034197
DNA Clean & Concentrator-5	Zymo Research	Cat# D4014
Creatinine (urinary) Colorimetric Assay Kit	Cayman Chemical	Cat# 500701
miRNeasy Mini Kit	Qiagen	Cat# 217004
KAPA Library Quantification Kits	Peqlab	Cat# KK4856
RNeasy Mini Kit	Qiagen	Cat# 74106
RNase-free DNase Set	Qiagen	Cat# 79254
High-Capacity cDNA Reverse Transcription Kit	Applied Biosystems	Cat# 4368813
Takyon Low ROX Probe 2x MasterMix dTTP Blue	Eurogentec	UF-LPMT-B0701
Deposited data		
RNA-seq: Conditional podocyte Arhgef7 knockout mice	NCBI GEO	GSE138774[115]
RNA-seq: Aged mice	NCBI GEO	GSE136138[116]
Microarray: Ercc1-deficient hemizygous (Ercc1[-/Δ]) mice	NCBI GEO	GSE43061[117]
RNA-seq: eNOS homozygous deletion (eNos−/−) diabetic mice induced with streptozotocin (datasets: GSM3942312-3: control, GSM3942314-6: streptozotocin, GSM3942320-1: control)	NCBI GEO	GSE134327[118]
RNA-seq: Leptin receptor mutant BKS homozygous db/db diabetic mice (datasets: GSM3514331-6: control, GSM3514343-8: disease)	NCBI GEO	GSE123853[119]
RNA-seq: Leptin receptor mutant BKS homozygous db/db diabetic mice	NCBI GEO	GSE77717[120]
RNA-seq: eNOS homozygous deletion (eNos−/−) diabetic mice induced by streptozotocin	NCBI GEO	GSE79291[121]
Microarray: BTBR ob/ob leptin-deficient diabetic mice (time points: 4, 8, 12, 16 weeks)	NCBI GEO	GSE106841[122]
Microarray: OVE26 diabetic mice	NCBI GEO	GSE20844[123]
Microarray: BTBR ob/ob leptin-deficient diabetic mice (dataset: GSM3768235-40)	NCBI GEO	GSE131266[124]
Microarray: OVE26 diabetic mice	NCBI GEO	GSE36209[125]
RNA-seq: Diabetic mice induced with streptozotocin, and podocyte-specific Dach1 deletion mice	NCBI GEO	GSE168676[126]
Microarray: Conditional podocyte Cd151 knockout mice	NCBI GEO	GSE104624[127]
RNA-seq: Conditional podocyte Parva knockout mice	NCBI GEO	GSE181690[76]
Microarray: Inducible conditional podocyte Tln1 knockout ± valproic acid treatment	NCBI GEO	GSE112116[128]
RNA-seq: Conditional podocyte Cosmc knockout mice	NCBI GEO	GSE126217[125]

(Continued on next page)

Continued

REAGENT or RESOURCE	SOURCE	IDENTIFIER
RNA-seq: HIV-1 transgenic mice induced with adriamycin	NCBI GEO	GSE117987[129]
Microarray: Anti-GBM antibody nephritis	NCBI GEO	GSE56236[130]
RNA-seq: Mice with light chain deposition disease (datasets: _GLO_FRA: disease, _GLO_WT: control, _GLO_DH: control)	NCBI GEO	GSE119049[131]
Single nucleus RNA-seq: Homozygous whole-body Pdss2 mutant (V117M/V117M) mice	NCBI GEO	GSE164273[132]
RNA-seq: Nphs2 compound heterozygous mutant (R231Q/A286V) mice	Talyan et al.[133]	–
RNA-seq: Conditional podocyte Fyn homozygous and Cd2ap heterozygous knockout mice	NCBI GEO	GSE123179[134]
RNA-seq: Conditional podocyte Shroom3 knockdown mice	NCBI GEO	GSE110092[135]
Single nucleus RNA-seq: Nphs2 compound heterozygous mutant (R231Q/A286V) mice (murine model of podocyte damage)	This paper	GEO: GSE289798
Single nucleus RNA-seq: Whole-body Wt1 heterozygous deletion mice (murine model of podocyte damage)	This paper	GEO: GSE289798
Microarray: Cd2ap knockout mice	NCBI GEO	GSE63272[136]
RNA-seq: Adriamycin induced podocytopathy	NCBI GEO	GSE154955[137]
Microarray: Immunotoxin LMB2 induction on podocyte-specific CD25 overexpression (NEP25) mice	NCBI GEO	GSE108629[138]
RNA-seq: Inducible conditional podocyte Mafb knockout mice	NCBI GEO	GSE96044[22]
Single cell RNA-seq: Inducible conditional podocyte Ctcf knockout mice	NCBI GEO	GSE174102[78]
RNA-seq: Whole-body Wt1 heterozygous deletion mice	Talyan et al.[133]	
Microarray: Denys-Drash Wt1 mutant mice	NCBI GEO	GSE18358[139]
Microarray: Inducible conditional podocyte Foxc1/2 knockout mice	NCBI GEO	GSE117571[140]
Microarray: Conditional podocyte Ptip knockout mice	NCBI GEO	GSE17709[141]
Single cell RNA-seq: 4 models: (1) mice induced with nephrotoxic serum, (2) BTBR ob/ob leptin-deficient diabetic mice, (3) adriamycin induced podocytopathy, and (4) CD2AP homozygous deletion mice	NCBI GEO	GSE146912[65]
RNA-seq: Alcoholic hepatitis (AH) and alcoholic steatohepatitis (AHS), 9 total samples = 3 AH liver, 3 ASH liver, 3 control	NCBI GEO	GSE97234[142]
RNA-seq: CCl4-treated mice at 12 weeks and 24 weeks $n = 2$ (pooled biological replicates of 4 individual mice)	NCBI GEO	GSE99010[143]
RNA-seq: Chronic cholestatic liver fibrosis induced by 3,5-diethoxycarbonyl-1,4-dihydrocollidine (DDC), EtOH samples were not included. $N = 4$ at time point 0; $N = 5$ at timepoint 28 days	NCBI GEO	GSE119953[144]

(*Continued on next page*)

Continued

REAGENT or RESOURCE	SOURCE	IDENTIFIER
RNA-seq: Streptozotocin (STZ) induced liver damage and hepatocellular carcinoma. *N* = 3 for each time point, 6 time points	NCBI GEO	GSE153580[145]
RNA-seq: Mice on controlled diet for 16 weeks and treated with perfluoctanic acid (PFOA) or vehicle for 8 weeks *n* = 4 per group	NCBI GEO	GSE119441[146]
RNA-seq: Diethylnitrosamine (DEN) induction of NAFLD and HCC, *n* = 3 per group	NCBI GEO	GSE83240[147]
RNA-seq: Mice exposed to acetaminophen, *n* = 4 (per time point), timepoint 0 considered control	NCBI GEO	GSE111828[148]
RNA-seq: Mice exposed to high fat diet. EtOH and DDC samples in the same study were disregarded	NCBI GEO	GSE119953[144]
RNA-seq: Mice fed with either control or high fat diet for 16 weeks. *N* = 4	NCBI GEO	GSE119441[146]
RNA-seq: Different mouse strains exposed to high fructose diet. *n* = 18 for three strains	NCBI GEO	GSE123894[149]
RNA-seq: Mice exposed to high fat, cholesterol, and fructose (HFCF) diets for 1 or 3 months. *N* = 3 (per diet + diet combinations)	NCBI GEO	GSE135050[150]
RNA-seq: Mice fed an HFCF diet for 12 weeks and 24 weeks. Liver samples were pooled from 4 independent mice in two sequencing samples.	NCBI GEO	GSE99010[143]
RNA-seq: Mice fed normal diet or "fast food diet". Only vehicle treated mice were considered.	NCBI GEO	GSE148849[151]
RNA-seq: 3 mice fed normal chow, 2 mice fed amylin liver NASH (AMLN) diet	NCBI GEO	GSE138419[152]
RNA-seq: Mice fed cholin-deficient, amino acid defined high fat diet (CDAD-HFD) for 8 weeks. *N* = 4 per group	NCBI GEO	GSE137449[153]
RNA-seq: 4 vehicle-treated mice, 4 STAM (streptozotocin followed by high-fat diet) mice	NCBI GEO	GSE114261[154]
RNA-seq: 6, 9, 12-month old mice were grouped as young mice, 18-, 21-, and 24-month old mice as 'old'	NCBI GEO	GSE132040[155,156]
Single nuclei RNA-seq: normal and diseased liver	NCBI GEO	GSE210077[60]
Spatial transcriptomics: human liver (Liver Cell Atlas)	NCBI GEO	GSE192742[8,12]
Single nucleus RNA-seq: human NASH	NCBI GEO	GSE189600[59]
Single nucleus RNA-seq: mouse, 3- and 9-month normal chow (NC) and NASH diet	NCBI GEO	GSE189600[59]
Proteomics: mouse liver	Williams et al.[55]	–
Single cell RNA-seq: HFD-NASH and CDAA-NASH	NCBI GEO	GSE146049[99]
Single nucleus RNA-seq: Pdss2-V117M mutant (murine model of podocyte damage)	This paper	GEO: GSE289798

(*Continued on next page*)

Continued		
REAGENT or RESOURCE	SOURCE	IDENTIFIER
Bulk ATAC-seq: podocyte and non-podocyte cell fraction of murine glomeruli	This paper	GEO: GSE289795
Bulk RNA-seq: circadian experiment in murine kidneys	This paper	GEO: GSE289877
Xenium: Human kidney spatial transcriptomics and de-identified clinical metadata	This paper	Zenodo: https://zenodo.org/records/15746500
Proteomics: mouse glomerular cells	Proteome change DB	PXD016238[14]
Proteomics: podocytes from doxorubicin treated mice	Proteome change DB	PXD012063[54]
Proteomics: mouse kidney glomeruli (Control vs. podocin(R231Q/A268V))	Proteome change DB	PXD018326[157]
Proteomics: Anti-nephrin autoantibodies in minimal change disease and focal segmental glomerulosclerosis	Proteome change DB	PXD047417[53]
Experimental models: Organisms/strains		
Mouse: C57BL/6J	The Jackson Laboratory	JAX#:000664; RRID:IMSR_JAX:000664
Mouse: FVB/NJ	The Jackson Laboratory	JAX#:001800; RRID:IMSR_JAX:001800
Mouse: B6;129S6-Gt(ROSA)26Sortm1(CAG-tdTomato*,-EGFP*)Ees/J (ROSAnT-nG)	The Jackson Laboratory	JAX#:023035; RRID:IMSR_JAX:023035
Mouse: B6.Cg-Tg(NPHS2-cre)295Lbh/J	The Jackson Laboratory	JAX#:008205; RRID:IMSR_JAX:008205
Mouse: B6.129S1-Stat3tm1Xyfu/J (Stat3flox)	The Jackson Laboratory	JAX#:016923; RRID:IMSR_JAX:016923
Oligonucleotides		
Probes: Cyp7a1	Thermo Fisher Scientific	Mm00484150_m1
Probes: Cyp27a1	Thermo Fisher Scientific	Mm00470430_m1
Probes: Tbp	Thermo Fisher Scientific	Mm00446973_m1
Software and algorithms		
Xenium Explorer (version 3.2.0)	10X Genomics	https://www.10xgenomics.com/software
ImageJ (version 1.54)	Schneider, Rasband and Eliceiri.[158]	https://imagej.net/ij/
Huygen's software (version 25.04)	Scientific Volume Imaging	http://svi.nl
QuPath (version 0.6.0)	Bankhead et al.[159]	https://qupath.github.io/
InstanSeg (version 0.1.0-rc1)	Goldsborough et al.[160]	https://github.com/instanseg/instanseg
Fiji (version 2.16.0)	Schindelin et al.[161]	https://imagej.net/software/fiji/
SRA Toolkit (version 2.11.3)	–	https://github.com/ncbi/sra-tools
FastQC (version 0.11.9)	–	https://github.com/s-andrews/FastQC
MultiQC (version 1.1)	Ewels et al.[162]	https://github.com/MultiQC/MultiQC
biomaRt (version 2.50.2)	Durinck et al.[163,164]	https://github.com/Huber-group-EMBL/biomaRt
STAR (version 2.7.3)	Dobin et al.[165]	https://github.com/alexdobin/STAR
decontX (version 1.2.0)	Yang et al.[166]	https://github.com/campbio/decontX
Seurat (version 4.0.1)	Hao et al.[167]	https://github.com/satijalab/seurat
bwa- mem (version 0.7.17)	Li and Durbin[168]	https://github.com/lh3/bwa
Genrich (version 0.6.1)	Gaspar[169]	https://github.com/jsh58/Genrich
sf (version 1.0–20)	Pebesma et al.[170]	https://github.com/cran/sf
Azimuth (version 0.5.0)	Hao and Hao et al.[167]	https://satijalab.github.io/azimuth/index.html
DEP (version 1.4.1)	Zhang et al.[171]	https://bioconductor.org/packages/devel/bioc/vignettes/DEP/inst/doc/DEP.html
oligo (version 1.54.1)	Carvalho and Irizarry[172]	https://github.com/benilton/oligo

(*Continued on next page*)

Continued

REAGENT or RESOURCE	SOURCE	IDENTIFIER
DEseq2 (version 1.34.0)	Love, Huber, and Anders[173]	https://github.com/thelovelab/DESeq2
ClusterProfiler (version 4.10.1)	Yu et al.[174]	https://github.com/YuLab-SMU/clusterProfiler
AUCell (version 1.12.0)	Aibar et al.[11,62]	https://github.com/aertslab/AUCell
TSCAN (version 1.13.0)	Ji and Ji[62]	https://github.com/zji90/TSCAN
Monocle3 (version1.3.7)	Cao et al.[175]	https://github.com/cole-trapnell-lab/monocle3
gwasrapidd (version 0.99.17)	Ramiro et al.[176]	https://github.com/ramiromagno/gwasrapidd
TOBIAS (version 0.14.0)	Bentsen et al.[177]	https://github.com/loosolab/TOBIAS
MEME-suit (version 5.5.0)	Grant, Bailey, and Noble[178]	https://meme-suite.org/meme/
TFtargetCaller (version 0.7)	Sikora-Wohlfeld et al.[179]	https://cellnet-sb.cecad.uni-koeln.de/resources/transcription-factor-target-gene-prediction/
MetaCycle (version 1.2.0)	Wu et al.[180]	https://github.com/gangwug/MetaCycle
GLMM cosinor (version 0.2.0.9)	Kristensen et al.[181]	https://github.com/ropensci/GLMMcosinor
CRDscore (version 0.1.0)	He, Fan, and Zhang et al.[85]	https://github.com/yixianfan/CRDscore
Tempo (version 0.0.1.dev)	Auerbach, FitzGerald, and Li.[93]	https://github.com/bauerbach95/tempo
scDblFinder (version 1.13.6)	Germain et al.[182]	https://github.com/plger/scDblFinder
Programming code used for this work.	–	https://doi.org/10.5281/zenodo.19916603; https://doi.org/10.5281/zenodo.20087796

EXPERIMENTAL MODEL AND STUDY PARTICIPANT DETAILS

Animals

All animals were housed and maintained in the CECAD *in vivo* Research Facility, University of Cologne, or in a barrier-protected facility of the MPI for Metabolism Research, Cologne. All mice were kept under a 12-h light/dark cycle and had *ad libitum* access to food and water. All mice were maintained on C57BL6 (The Jackson Laboratory, Strain#000664) background except for Wt1$^{ko/wt}$ mice, which were kept on FVB/N (The Jackson Laboratory, Strain#001800) background. NPHS2^{mut} mice have been published.[48] Pdss2^{mut} mice were generated as previously published by the CECAD *in vivo* Research Facility (Figure S7A).[183] The Wt1$^{ko/wt}$ mice were previously described.[184] Mice carrying the nuclear fluorescent reporter (nTnG, The Jackson Laboratory, Strain#:023035) were crossed to mice carrying NPHS2 Cre (Pod:cre, The Jackson Laboratory, Strain#: 008205),[185] to generate podocyte-specific expression of nuclear GFP (nTnG;Pod:cre).

To induce liver phenotypes, the following experimental diets were fed to *Stat3*$^{fl/fl}$ (The Jackson Laboratory, Strain#:016923),[186] or *NIK*$^{fl/fl}$ mice[187] (in the context of this study considered as wildtype mice) mice starting at the age of 6 weeks: EF Control to NASH diet (CD: E15767-040, ssniff Spezialdiäten GmbH) containing 65% calories from carbohydrates, 22% of calories from protein, and 13% of calories from fat; EF NASH diet (HFD-NASH: E15766-340, ssniff Spezialdiäten GmbH) containing 42% calories from carbohydrates, 18% of calories from protein, and 40% of calories from fat; Choline free, lard, 1% cholesterol diet (CDAA-NASH: S2159-E042, ssniff Spezialdiäten GmbH) containing 58% calories from carbohydrates, 11% of calories from protein, and 31% of calories from fat. Controls received CD or NCD (R/M-H; ssniff Spezialdiäten GmbH), as indicated.

Animal experiments used age-matched littermates at indicated weeks of age. All mouse experiments were performed with approval from the Animal Care Committee of the University of Cologne and LANUV NRW (Landesamt für Natur, Umwelt und Verbraucherschutz Nordrhein-Westfalen, State Agency for Nature, Environment and Consumer Protection North Rhine-Westphalia) for this study.

To analyze circadian gene expression, two groups of male C57BL/6J mice aged 8 weeks and 78 weeks were acclimated for 2 weeks under 12-h/12-h light–dark cycles before euthanasia at zeitgeber times (ZT): 0, 3, …, 21 h, in accordance with the regional animal ethics committee guidelines. Duplicates of pooled samples consisting of three biological replicates were acquired per zeitgeber time and group, respectively.

Human clinical sample acquisition

Patients included in this study were seen in the Department of Nephrology at the University of Cologne for their first manifestation of the nephrotic syndrome. Kidney biopsies were carried out as part of routine patient care. After diagnosis with either MCD or FSGS and after providing informed consent, patients were enrolled in the ForMe registry (ClinicalTrials.gov identifier NCT039499728)[56]

intended for the study of MCD and FSGS patients. Within this registry, we provide comprehensive genetic analysis, monitor the clinical outcome, and carry out *in vitro* and *in vivo* analysis of biosamples and novel mutations to predict and investigate MCD and FSGS outcomes. The registry itself and its proceedings were approved by the local ethics committee of the University Clinic Cologne, Germany.

Core patient clinical data at the time of kidney biopsy have been made available in de-identified fashion together with human spatial transcriptomics data (see resource availability). Information on gender, ancestry, race, and socioeconomic status were not acquired within the registry and are hence not reported here.

METHOD DETAILS

Isolation of glomeruli and nuclei

Mice were sacrificed by cervical dislocation and kidneys were extracted and perfused *ex vivo* through the renal arteries with magnetic M-450 Tosylactivated Dynabeads (14013, Thermo Fisher) suspension in HBSS (5.4 mM KCl, 0.3 mM Na_2HPO_4, 0.4 mM KH_2PO_4, 4.2 mM $NaHCO_3$, 137 mM NaCl, 5.6 mM D-glucose, 1.3 mM $CaCl_2$, 0.5 mM $MgCl_2$, 0.6 mM $MgSO_4$). Perfused kidneys were minced by scalpel and digested with digestion buffer (1 mg/mL Pronase E (P6911, Sigma), 1 mg/mL collagenase type II (LS004176, Worthington) and 50 U/ml Dnase I (A3778,0050, Applichem), in HBSS) for 15 min at 37°C. The kidney suspension was triturated until homogenous and filtered twice through 100 μm cell strainers (83.3945.100, Sarstedt). The kidney lysates were washed with HBSS and pelleted at 1500 rpm for 5 min at 4°C. The pellet was resuspended in HBSS and washed on the magnet until isolated glomeruli were obtained.

Isolated glomeruli were resuspended in 1 mL of lysis buffer (EZ lysis buffer (NUC101-1KT, Sigma) supplemented with EDTA-free protease inhibitors (5056489001, Roche)) and shaken at 1400 rpm for 10 min at 4°C. After magnetization, the supernatant containing loose nuclei was collected through a 30 μm cell strainer (04-004-2326, Sysmex). The leftover beads were resuspended in 1 mL lysis buffer further lysed 5 times through a 27-G needle. After the loose nuclei were collected, the beads were resuspended in 1 mL of lysis buffer and further lysed through a 30-G needle 5 or 10 times to collect the final aliquots of loose glomerular nuclei. The pooled nuclei were centrifuged at 500 g for 5 min at 4°C. The pellet was resuspended in 300 μL of lysis buffer, transferred to a 1.5 mL tube and placed on the magnet for 3 min to remove residual magnetic beads.

ATAC-seq

50,000 nuclei were isolated from glomeruli of nTnG;Pod:cre mice, stained with DAPI (D9542, Sigma) and cell sorting was performed on the FACSAria IIIu and FACSAria Fusion cell sorters using a 70μm nozzle at 70 psi pressure. The samples and the collection tubes were chilled during sorting. Single nuclei were gated based on DAPI fluorescence, followed by gating on GFP and tdTomato signals. GFP-positive nuclei were subsequently sorted for downstream analysis and collected into lysis buffer. Nuclei were centrifuged at 1000 g for 10 min at 4°C and resuspended in 50 μL of transposition reaction mix (25 μL of transposition buffer, 2.5 μL of TDE1 (20034197, Illumina) and 22.5 μL of nuclease-free water). The reaction was carried out at 37°C for 30 min. Immediately following transposition, DNA was purified using the DNA clean concentrator-5 kit (D4014, Zymo Research) per manufacturer's instructions. Library preparation and sequencing were performed by the Cologne Center of Genomics.

Single-nuclei RNA-seq

Glomeruli were isolated from murine kidneys and subjected to isolation of nuclei using lysis buffer supplemented with 0.1% murine RNase inhibitor (M0314L, New England Biolabs GmbH). Isolated nuclei were counted with a hemocytometer. After centrifugation at 1000*g* for 10 min, nuclei were resuspended in PBS supplemented with 2% BSA (3737.2, Carl Roth) to reach an approximate concentration of 1000 nuclei per μL. Prior to immediate processing, the samples were further filtered through a 10 μm strainer (04-004-2324, Sysmex). For $Wt1^{ko/wt}$ and $Nphs2^{mut}$ mice, glomeruli nuclei were used for snRNA-seq. For $Pdss2^{mut}$ mice, glomeruli nuclei were combined with nuclei extracted from whole kidney (1:1 volume) to additionally capture tubules. Libraries and sequencings were prepared per the manufacturer's instructions supported by 10X Genomics by the Cologne Center of Genomics.

Bulk RNA-seq

To analyze circadian gene expression in kidneys, whole kidneys were dissected, snap-frozen and RNA was prepared using the miRNeasy mini Kit (217004, Qiagen) to isolate total RNA. After removal of ribosomal RNA using biotinylated target-specific oligos combined with Ribo-Zero ribosomal RNA removal beads, RNA was fragmented into small pieces and copied into first-strand complementary DNA (cDNA) followed by second-strand cDNA synthesis. Products were purified and enriched with PCR to create the final cDNA library. After library validation and quantification using the 2100 Bioanalyzer (Agilent), equimolar amounts of five to six pooled libraries were quantified by using the KAPA Library Quantification Kit (KK4856, Peqlab) and the Applied Biosystems 7900HT Sequence Detection System and sequenced on a Hiseq2000 sequencer.

Xenium spatial transcriptomics

Xenium Prime spatial transcriptomics were carried out using the 5K human pan tissues and pathways panel supplemented with a custom 50 gene panel covering PDS genes. For generation of the custom panel, the top 70 ranking genes were provided to the

10X custom panel design tool. Genes predicted to result in optical crowding due to high expression levels and genes predicted to cause technical problems in probe design as by 10X recommendations were excluded from the panel. The remaining top 50 PDS marker genes were included in the final custom panel. In case of genes overlapping between the 5K human panel and the PDS marker gene set, the number of probes per gene was boosted to improve the PDS signal. Full information on the custom panel is available with the Xenium spatial transcriptomics raw data (see section on resource availability).

Kidney biopsy cylinders of four patient biopsies were combined in one FFPE tissue block, sectioned at 5 μm thickness and mounted according to 10X instructions and processed for Xenium spatial transcriptomics according to the manufacturer's instructions. Standard H&E staining was carried out upon completion of spatial transcriptomic data acquisition. Biopsy cylinder attribution to individual patients is ensured by metadata and H&E images uploaded together with Xenium raw data (see section on resource availability). Serial sections were obtained in the same manner for confocal microscopy (see "immunofluorescence and quantification").

Standard built-in segmentation analysis of the Xenium Prime platform was used for single podocyte segmentation. Images were generated using the Xenium Explorer 3 software (version 3.2.0).

STED microscopy and quantification

Murine kidney tissues were fixed in formalin overnight at 4°C and stored in PBS. Kidney cortex was cut into 1–2 mm pieces and immersed in hydrogel solution (4% acrylamide (Carl Roth), 0.25% VA-044 initiator (Sigma) in PBS) overnight at 4°C. The gel was polymerized by incubation at 50°C for 3 h. Excess polymer was removed, and tissues were washed in PBS three times and embedded in 3% agarose (Carl Roth) prior to cutting into 200 μm sections with a vibratome (Leica). Samples were transferred to a clearing solution (200 mM boric acid, 4% SDS, pH 8.5) and incubated at 50°C until cleared. Samples were washed briefly in a wash buffer (10 mM HEPES, 200 mM NaCl, 3% Triton X-100) before incubation with primary antibodies (nephrin: 20R-NP002, Fitzgerald; THSD7A: HPA000923, Sigma; Claudin-5: 35–2500, Thermo Fisher Scientific; EphB1: PAB3018, Abnova) at 1:100 at 37°C overnight. Samples were washed with wash buffer before incubation with secondary antibodies (goat anti-guinea pig: 106-005-003, Jackson ImmunoResearch; donkey anti-rabbit: 711-005-152, Jackson ImmunoResearch; goat anti-mouse: 115-005-003, Jackson ImmunoResearch; each conjugated to fluorophores in-house) at 1:100 at 37°C overnight and embedded in saturated fructose solution (80.2% w/w fructose (USBIF8000, VWR), 0.5% 1-thioglycerol (M6145, Sigma)) before mounting in a glass-bottom dish (Ibidi) for imaging with the SP8 STED microscope (Leica).

To quantify the distribution of Claudin 5 at the podocyte slit diaphragm, the fluorescent intensity of claudin 5 along a manually traced slit was quantified in ImageJ (version 1.54),[158] the area of under the curve was calculated and normalized to slit diaphragm length.

Albumin-to-creatinine ratio quantification

Urines from mice were analyzed to determine albumin and creatinine ratio (ACR). Urinary albumin was determined by ELISA. Briefly, wells were coated for 1 h with 100 μL of anti-mouse albumin coating antibody (A90-134A, Bethyl Laboratories) at 1:10,000 in coating buffer (0.05 M carbonate-bicarbonate, pH 9.6), then washed 5 times with 200 μL of wash solution (50 mM Tris, 0.14 M NaCl, 0.05% Tween 20, pH 8.0) before incubating for 30 min with 200 μL of blocking solution (50 mM Tris, 0.14 M NaCl, 1% BSA, pH 8.0). The wells were washed again 5 times with 200 μL of wash solution before incubation for 1 h with 100 μL of standards or samples diluted in diluent buffer (50 mM Tris, 0.14 M NaCl, 0.05% w/v Tween 20, 1% w/v BSA, pH 8.0). Next, the wells were washed 5 times with 200 μL of wash solution before incubation with 100 μL of HRP detection antibody (A90-134P, Bethyl Laboratories) diluted 1:25,000 in the diluent buffer for 1 h. Finally, the wells were washed 5 times with 200 μL of wash solution and developed with 100 μL of substrate solution (100 μg/ml 3,3′,5,5′-Tetramethylbenzidine (TMB), 48 mM sodium acetate, 0.01% v/v hydrogen peroxide, pH 5.2) for 15 min in the dark. The reaction was stopped by adding 100 μL of stop solution (0.18M sulfuric acid) and the absorbance was measured at 450 nm. Urinary creatinine was measured using a commercial colorimetric kit (500701, Cayman Chemical). Briefly, urines were diluted 1:20 in water and assayed according to the manufacturer's protocol. All samples were measured in triplicates. To obtain the urinary ACR, albumin concentrations were divided by creatinine concentrations.

Immunofluorescence and quantification

Human kidney biopsies were sectioned into 5 μm thickness and mounted on slides for confocal imaging as described in Unnersjö-Jess et al.[188] Briefly, after deparaffinization antigen retrieval was carried out with heated Tris-EDTA at pH 9. Slides were washed in PBS supplemented with Triton X-0.5% and incubated with primary antibody (nephrin: AF4269, R&D Systems) in PBS supplemented with 5% bovine serum albumin and 5% normal donkey serum overnight. Slides were washed in PBS and incubated with Alexa 488-coupled donkey anti-goat secondary antibody (A11055, Thermo Fisher Scientific), washed in PBS supplemented with DAPI and mounted with 80% fructose solution with 0.5% 1-thioglycerol. Slides were imaged on an SP8 3x confocal microscope (Leica) using a 100x lens. Acquired images were deconvoluted using Huygen's software (version 25.04). The slit diaphragm length per area was measured using the nephrin staining using ImageJ (version 1.54)[158] and published macro as described in Butt et al.[48]

Formalin-fixed paraffin-embedded (FFPE) murine kidney samples were sectioned to 5 μm using a microtome and treated by heat-mediated antigen retrieval in Tris-EDTA pH 8. The sections were permeabilized using PBS-T (0.1% Triton X-100) and blocked for 1 h at room temperature using normal donkey serum (017-000-121, Jackson ImmunoResearch) before overnight incubation with primary

antibodies (Nr1d1: 13418, Cell signaling Technology, Wt1: AF5729, R&D Systems, PKM: 25659-1-AP, Proteintech, phosphorylated-S6: 4858, Cell Signaling Technology). Samples were washed and stained with secondary antibodies and Hoechst dye before mounting in ProLong Gold Antifade Mountant (P36934, Invitrogen). Images were acquired using the Zeiss Apotome 3 microscope. Podocyte nuclei were segmented and the expression of Nr1d1 was quantified using QuPath (version 0.6.0),[159] using the InstanSeg extension (version 0.1.0-rc1) and the fluorescence_nuclei_and_cells model.[160] Nphs2^{mut} mice aged 8 weeks and Pdds2^{mut} mice aged 21 weeks were used for phospho-S6-kinase and Pkm staining, as these represented similar damage levels as by SD length and albuminuria. For Pkm staining kidneys were sectioned to 2 μm and heat-mediated antigen retrieval was carried out using citrate buffer.

FFPE murine livers were sectioned to 7 μm using a microtome, deparaffinized, and rehydrated prior to heat-mediated antigen retrieval in citrate buffer pH 6. Sections were treated for 10 min with 0.3% glycine in PBS, washed with PBS, and permeabilized using 0.03% SDS in PBS. Sections were blocked for 1 h with 3% normal donkey serum in PBS-T (0.25% Triton X-100). Primary antibody (CYP7A1: PA5-100892, Thermo Fisher Scientific, 1:100) was applied overnight at 4°C. Slides were washed with PBS-T (0.1% Triton X-100) and stained with secondary antibodies and Hoechst 33342 (H1399, Invitrogen, 1:1000) diluted in PBS-T (0.25% Triton X-100) at RT. After a final wash, slides were mounted using Vectashield Antifade Mounting Medium (Vector Laboratories). Images were acquired with an Olympus Slideview VS200 system (Olympus). For CYP7A1 quantification, regions of interest (ROIs) around bile ducts were preselected in 16-bit.vsi images using QuPath (version 0.6.0). ROIs were further processed using Fiji (version 2.16.0) by measuring the mean intensity of CYP7A1-positive areas. The average CYP7A1 mean intensity per mouse liver is represented.

RT-qPCR

Frozen liver tissue was homogenized in QIAzol (79306, Qiagen), followed by total RNA isolation using the RNeasy Mini Kit (Qiagen, 74106) with DNase treatment using the RNase-free DNase Set (79254, Qiagen) according to the manufacturer's instructions. Reverse transcription was performed using the High-Capacity cDNA Reverse Transcription Kit (4368813, Applied Biosystems) according to the manufacturer's protocol. Subsequently, qPCR was conducted using the QuantStudio 7 Flex Real-Time PCR System with Taqman Real-Time PCR Assays (*Cyp7a1*: Mm00484150_m1, *Cyp27a1*: Mm00470430_m1, *Tbp*: Mm00446973_m1, Thermo Fisher Scientific) and Takyon Low ROX Probe 2x MasterMix dTTP Blue (UF-LPMT-B0701, Eurogentec). Relative target mRNA expression was normalized to expression of *TATA binding protein* (*Tbp*) and analyzed using the $2^{-\Delta\Delta Ct}$ method. Expression levels are presented relative to the control condition.

Senescence-associated β-gal staining

Livers were removed from mice and cryopreserved in Tissue freezing medium (14020108926, Leica). 15 μm cryosections were generated using the Leica CM3050 S cryostat. Sections were fixed in 0.2% glutaraldehyde (50-262-23, Electron Microscopy Sciences) in PBS for 10 min at room temperature. Sections were washed for 3 × 1 min using PBS at RT and incubated in freshly prepared X-gal staining solution (40 mM citrate sodium dihydrogen phosphate buffer pH 5.5, 5 mM potassium hexacyanoferrate(II) trihydrate, 5 mM potassium ferricyanide(III), 150 mM sodium chloride, 2 mM magnesium chloride hexahydrate, and 1 mg/mL 5-bromo-4-chloro-3-indolyl-b-D-galactopyranoside (X-gal)) for 24 h at 37°C. Finally, slides were washed 3 × 1 min with PBS, dehydrated, and embedded using Cytoseal XYL medium (Thermo Fisher Scientific). Images were acquired with an Olympus Slideview VS200 system (Olympus).

Transcriptomic data retrieval and processing

CEL files were downloaded for microarray data. UMI count tables were downloaded for single cell RNA-seq experiments. Unnormalized gene expression count matrices were used, when available, in case of bulk RNA sequencing experiments. If the data was not available in this format, unnormalized count matrices were generated from raw reads, as described below.

Gene IDs were mapped to Mouse Genome Informatics (MGI) gene symbols as a common reference using the R package biomaRt (version 2.50.2).[163,164] On a few occasions, if the expression dataset included counts for different splice variants of the same gene, the average expression across the splice variants was taken as the expression count of that gene before converting to MGI nomenclature, since MGI nomenclature does not account for alternative splicing.

RNA-seq data processing

Raw sequencing files of published datasets available in the 'sra' format were extracted and converted to 'FASTQ' format using the SRA Toolkit (version 2.11.3). Quality control of FASTQ files was done with FastQC (version v0.11.9).[189] MultiQC (version v1.1) was used to summarize the quality statistics of multiple samples from one study into a comprehensive overview.[162] No samples were excluded due to lack of quality.

An index of the *Mus musculus GRCm38 (mm10) and* GRCm39 reference genomes and its annotations were generated using STAR (version 2.7.3) aligner to be used for alignment of raw reads from liver and kidney studies,[165] correspondingly. When STAR was run to align reads, the option -quantMode' was set to the command 'GeneCounts' to generate count matrices as an output. Gene abundances were quantified using both exonic and intronic reads with pre-mRNA full gene models.

Sn- and sc-RNA-seq data processing

STARsolo function of the STAR aligner was used to map reads of single nuclei RNA-seq experiments. Sequence files from 2 sequencing lanes, if present, were pulled in one sample. Ambient RNA was removed from UMI count tables using decontX (version 1.2.0) R package.[166]

The read count (UMI) matrices of all single cell RNA-seq samples were processed in Seurat (version 4.0.1) R package.[167] As individual datasets stem from multiple sources data was handled individually with respect to quality control, filtering, dimensionality reduction, and normalization. The respective parameters have been included in out GitHub repository (see Section on Code availability). Doublets were removed in snRNA-seq datasets with scDblFinder (version 1.13.6) using cluster-based approach.[182]

ATAC-seq data processing

Mapping to the mouse genome mm10 was done using bwa-mem (version 0.7.17) with default parameters.[168] Data quality was assessed by calculating the insert sizes between the R1 and R2 read pairs. Peaks were called using Genrich (version 0.6.1) tool using default parameters.[169]

Spatial transcriptomics data analysis

Mouse visium kidney spatial transcriptomics

Kidney Slide-seq V2 spatial transcriptomics data was retrieved from GSE190094.[13] Podocyte annotation and KNN filtration, glomerular identification and size estimation were performed using the original code from the publication.[13] Samples with low capture rates (below the average of 100 features per bead) and without visually detectable podocyte clusters were removed, which excluded 20 out of 49 original samples from the downstream analysis. For the remaining 29 samples, the number of beads annotated as podocytes was normalized to the glomerular area. Subsequently, the bead density per glomerulus was correlated with the average PDS of the respective glomerulus.

Mouse visium liver spatial transcriptomics

HDS was applied on spatial transcriptomic data from the Liver Cell Atlas dataset.[8,12] Seurat (version 4.0.1) was used to analyze the 10X Visium counts.[167] Uninformative spots located in regions with no or minimal tissue were removed from analysis. Filtering for sequencing quality control was performed using the same parameters as described in the Liver Cell Atlas GitHub repository accessible at https://github.com/guilliottslab/scripts_GuilliamsEtAll_Cell2022. Spots were retained only if they contained more than 1000 detected features (nFeature), total number of counts (nCount) between 5000 and 60 000, and less than 15% mitochondrial RNA. The HDS was computed per spot.

Human kidney spatial transcriptomics

10X Visium spatial transcriptomic data from a human with FSGS, patient ID 29–10282, was retrieved from KPMP and analyzed.[2] The count data and the spots coordinates were loaded and processed with Seurat (version 4.0.1).[167] Glomeruli were annotated manually. The PDS was calculated per spot for all spots within annotated glomeruli using the PDS_calc function (see Github repository – section Code availability) with standard parameters.

Human Xenium data was analyzed with the Seurat package. Slide scans were split in individual samples with sf (version 1.0–20)[170] R package by drawing a polygon around each sample and then cropping all layers of the Seurat object to the selected region. Default cellular segmentation was used for the analysis. Cell types were annotated with Azimuth (version 0.5.0)[167] R package, using the default set of cell-type markers.

For automated glomerular size estimation, coordinates of podocytes, endothelial and mesangial cells were used as input to KNN filtration and clustering (see Method section Mouse kidney spatial transcriptomics), to identify clusters of glomerular cells and filter out scattered individual cells. Glomeruli were segmented by drawing circles around centers of clusters, using distance from the cluster center to the furthest cell of the cluster as glomerular radius. Results were inspected manually and nested glomeruli or clusters containing more than one glomerulus were excluded from downstream analysis. PDS was calculated for all individual podocytes within automatically segmented gloms as detailed for Visium analysis.

Human liver spatial transcriptomics

Human spatial transcriptomic data from patients ‘‘H35’’ and ‘‘H37’’ were retrieved from the Liver Cell Atlas[8,12] and Seurat was used to analyze 10x Visium counts. Spots representing hepatocytes were manually annotated and classified a steatotic or non-steatotic based on histology. The count matrices were subset to include only protein coding genes and only the spots annotated as hepatocytes with more than 250 detected features were included in the analysis (nFeature_Spatial >250). We used the Seurat function NormalizeData() to normalize the counts per spot in each sample and proceeded to calculate the HDS.

Proteomics data analysis

Proteomics datasets PXD016238,[14] PXD018326,[48] PXD047417,[53] and PXD012063[54] were retrieved from proteome exchange. The LFQ data was processed with the DEP (version 1.4.1).[171] Only proteins expressed in more than 50% of the replicates in at least one condition were considered. To calculate PDS in proteomics data, 62 instead of 42 podocyte damage markers were used to ensure at least half of the markers are present in all the studies. For the application of the HDS on murine proteomic data, processed data from Williams et al. provided by the authors were used.[55]

Differential gene expression analysis

CEL files for microarray data were processed and differential expression analysis performed using the oligo (version 1.54.1)[172] R package with the default parameters. Non-normalized bulk RNA-seq count tables were subjected to differential expression analysis with DEseq2 (version 1.34.0),[173] with default parameters. For hepatocyte data, counts were transformed using the variance stabilizing transformation in DESeq2 with the parameter 'blind' set to 'true'. Single cell RNAseq UMI count tables were processed and differential expression analysis performed with Seurat[109] using the FindMarkers() function with the default parameters. For single cell data, only cells of the respective cell type of interest were considered (i.e., podocytes or hepatocytes). Gene set over-representation analysis was performed using ClusterProfiler (version 4.10.1).[174] For details on specific parameters, see our GitHub repository.

Generation of PDS and HDS marker genes

Candidate lists for damage markers were generated to ensure that marker genes were expressed in the targeted cell type (here: podocyte for PDS, hepatocyte for HDS). Data from diseased and control samples were included. The expression of genes included in the damage scores was not required to be exclusive to the targeted cell type, allowing for the inclusion of marker genes that are generically expressed in various cell types.

To create a catalog of genes expressed in podocytes, three single-nuclei RNA-seq kidney datasets generated within this study, and four published single-cell RNA-seq kidney datasets from GSE146912[55] (Table S1) were used, with each of the datasets representing a unique podocyte damage model. Cells annotated as podocytes were subset from all seven datasets and used for the selection.

To create a catalog of genes expressed in hepatocytes, murine gene expression data from the Liver Cell Atlas[8,12] were used and analyzed using Seurat.[167] Cells annotated as hepatocytes by the authors were subsetted from the two experiments available: Standard Diet and Non-Alcoholic-Fatty-Liver-Disease (NAFLD).

The selection procedure for the identification of genes expressed in podocytes and hepatocytes single-cell RNA-seq data was identical. A detection rate for each gene was calculated across cells that fulfilled the following criteria: 'nFeature_RNA' greater than 500 and lower or equal to 6,000, less or equal to 2.5% mitochondrial RNA and 'nCount_RNA' smaller than 30,000. Genes with a detection rate of at least 0.03 (i.e., 3% of the cells) in the target cell type were kept for the subsequent analyses.

Damage score algorithm

To generate the damage scores, we selected and analyzed datasets that represent different disease models and that were partially obtained using different experimental platforms (Table S1), as well as generated additional experimental datasets for the PDS (see above and Table S1). First, each dataset was individually processed and analyzed to obtain differentially expressed genes, always comparing experimental condition versus control. In case of the single cell data, only cells of the respective cell type were considered (i.e., podocytes or hepatocytes). Results of differential expression analysis were integrated across studies in the following way:

Gene must be identified as potentially expressed in podocytes or hepatocytes respectively.

Genes were selected if they were detected in $\geq$75% of the studies.

Out of those, genes were kept if their fold changes were in the same direction (i.e., either positive or negative fold change) in $\geq$75% of the studies.

Selected genes were ranked by their *p*-value of differential expression per study. *p*-values for differential expression may not be comparable between studies, due to variation in sample numbers and/or variable quality of the data. We therefore decided to base the selection process on ranks of *p*-values rather than defining a common *p*-value threshold across studies.

Ranks were combined across studies by averaging the rank of each gene.

Genes were sorted by their mean rank, and the top N ($N = 42$ for the final score) were selected as a universal damage signature. In the calculation of the score, damage signature genes were treated differently depending on whether they were up or downregulated under the disease condition (see below).

Damage scores were computed for each cell/spot/sample using the damage signature genes with the AUCell (version 1.12.0)[11] R package. AUCell scores were calculated separately for up and down regulated genes and combined as follows to get the final podocyte damage score:

$$DS = \frac{n_{up}}{42} DS_{up} - \frac{n_{down}}{42} DS_{down},$$

where DS_{up} and DS_{down} are the respective AUCell scores obtained with the up and downregulated genes. Note that the AUCell score is negative for low-ranking genes. These scores were weighted based on the number of up and down regulated genes (n_{up}, n_{down}). Since in both cases (PDS and HDS) the majority of marker genes were downregulated, DS_{down} contributed more to the final damage score DS.

Damage score validation

Leave-one-model-out cross-validation

Cross validation was performed by excluding all data of one disease model group (Table S1) from the training procedure. Damage signature genes were determined as described above on the remaining training data. Subsequently, damage scores were computed as described above on the samples from disease model left out for testing.

Damage signature randomization test

Robustness of the PDS was tested by randomizing an increasing percentage of studies used to generate the PDS by means of shuffling *p*-values and LFCs of differential expression results. Signatures for partially randomized data were generated and used to calculate deviant damage scores in a test snRNA-seq dataset. As the choice of studies for randomization likely affects the resulting scores, 50 rounds of subsampling of studies were carried out for each level of randomness (i.e., percentage of randomized studies) and results were averaged separately for control and experimental conditions of the test dataset (nephrotoxic serum nephritis, GSE146912).[65]

Damage signature transfer to human data

Damage signature genes in humans were determined by manually mapping mouse genes to their human orthologs using the MGI gene annotation.[190] Signature genes without known human orthologs were omitted. In cases of several human orthologs to a single mouse gene, we kept all human orthologs. In cases of several mouse disease signature genes with a single human ortholog, we included the human ortholog and reduced the number of genes contributing to score calculation.

Pseudo-time trajectory inference

After filtering hepatocytes based on annotation and quality control metrics using Seurat, we performed pseudo-time trajectory (PT) inference on normalized counts of each sample separately. We used TSCAN (version 1.13.0),[62] and Monocle3 (version 1.3.7)[63] with default parameters.

Damage score alignment test

We hypothesized that damage scores of cells from control samples were more similar to each other across studies, as opposed to damage scores from experimental conditions varying stronger between studies, as different interventions affect cellular damage to different degrees. To test our assumption statistically, we first calculated pairwise Kolmogorov-Smirnov (KS) distances between the damage score distributions of all control and experimental samples of all sn/scRNA-seq datasets and then used a one-sided two-sample Wilcoxon test to see if the average KS distance between damage score distributions of individual samples is lower in control than in experimental samples.

Disease association of damage signature genes

To find genomic variants associated with damage signature genes, we extracted the genomic coordinates of the RefSeq canonical isoforms for PDS and HDS marker gene loci and extended them to include 1) a 2 kb promoter; 2) the 5′ upstream DNA sequence spanning from the start of the promoter to the adjacent gene or 250 kb whichever is smaller; and 3) the 3′ downstream sequence spanning from the end of the 3′ UTR to the adjacent gene or 5 kb whichever is smaller. We then used the gwasrapidd (version 0.99.17)[176] R package to search the NHGRI-EBI GWAS catalog for all variants associated with EFO traits related to kidney or liver disease, respectively. Search terms have been deposited in our GitHub repository (see Section on Code availability). Finally, we intersected selected variants with the extended genomic ranges of the podocyte and liver damage marker genes. To test the significance of enrichment of damage signatures in GWAS variants we used Fisher's exact test, where all genes expressed in sc/snRNA-seq kidney and liver datasets were used as the corresponding backgrounds.

Pathway activity analysis

Pathway activity was defined as the AUCell (version 1.12.0)[11] score obtained for the set of genes annotated as pathway members. Pathway membership (i.e., gene annotation) was taken from Reactome, KEGG and 50 hallmarks from MSigDB.[191] This scheme was used to compute relative pathway activities per single cell and for bulk RNA-seq samples. To obtain pathway signatures of the damage models we computed Spearman correlation of single-cell pathway activities with the PDS for each damage model and for the cumulated controls. Per disease model, the top 10 significantly correlating pathways were included in further analyses (adjusted *p*-value <0.05), resulting in a set of 85 pathways (Figure S8C). Of those 85 pathways, 26 pathways were manually selected for visualization in Figure 4A.

Transcription regulatory network construction

To identify transcription factors (TFs) expressed in podocytes, we utilized sc/snRNA-seq data from podocytes as follows: we selected TFs with a detection rate above 5% in at least one of the analyzed sc/snRNA-seq datasets, which resulted in 110 transcription factors contributing to the construction of the podocyte transcription regulatory network (TRN). A TRN is a directed graph representing interactions between transcription factors and their target genes. The TRN was constructed using the podocyte ATAC-seq data and known transcription factor binding motifs in following steps: First, ATAC-seq footprinting was performed on the ATAC-seq peaks with TOBIAS (version 0.14.0)[177] to more precisely locate putative TF binding events.[177] Second, motif scanning was done on ATAC-seq footprints using FIMO from the MEME-suit (version 5.5.0).[192] Motif hits with a *p*-value $<5 \times 10^{-4}$ were kept for the analysis. Third, motif hits (i.e., potential TF binding sites) for each of the 110 TFs were associated with putative target genes using the ClosestGene approach implemented in the TFtargetCaller (version 0.7) R package.[179] Genes with a q-value <0.1 were assigned as targets of the corresponding TF.

TFs of a common family often have very similar or even identical position weight matrices (PWMs) resulting in virtually identical DNA binding motif predictions. To address this ambiguity, PWMs of 110 analyzed TFs were subjected to hierarchical clustering, resulting in 14 TF motif clusters with high similarity of motif sequences and predicted target genes within each TF family. We combined predicted targets of all TFs within each family into one regulon for the downstream analysis.

Circadian clock analysis

Kidney-specific circadian genes analysis

Bulk RNA-seq data from kidneys of young (10 weeks) and old (80 weeks) mice were used to identify kidney-specific circadian genes. Cycling genes were identified with the meta2d(cycMethod = "JT") function from MetaCycle (version 1.2.0).[180] Overall, 317 genes with BH adjusted *p*-value <0.01 in either young or old mice were called 'circadian'. Estimation of acrophase and acrophase uncertainty for circadian genes was done by fitting a cosinor model with the cglmm(Y ~ amp_acro(time_col = Time, period = 24)) function of GLMM cosinor (version 0.2.0.9)[181] R package, where Y are the rlog-normalized expression values of a gene.

Circadian rhythm disruption analysis

Circadian rhythm disruption (CRD) in individual podocytes was calculated using cal_CRDscore() from the CRDscore (version 0.1.0) R package.[85] The set of cycling genes identified from the kidney circadian bulk RNAseq was used to calculate CRD.

Tempo analysis

Circadian time in individual podocytes was estimated by the Tempo (version 0.0.1.dev)[93] algorithm using expression of core clock genes. Estimates of gene acrophases, required by the algorithm, were taken from the analysis of bulk circadian RNA-seq data from the mouse kidney dataset. *Clock* was used as the reference gene.

Hepatocyte cell-state analysis

AUCell was used to calculate the activity score of genes in the SHGS gene set.[96] The HDS and SHGS activity scores were then used to classify hepatocytes in murine snRNA-seq data from Carlessi et al.[98] into five cell state trajectories. The global HDS distribution across all sampleswas assessed to define HDS thresholds: undamaged - HDS smaller than the global 25%-quantile; transition - HDS between the global 25%- and 75%-quantile, damaged - HDS greater than the global 75% quantile. The distribution of the SHGS activity score across samples from the TAA condition was used to set senescence thresholds: senescent – greater than 75% quantile; unresolved – between 25% and 75% quantile, not senescent – lower than 25% quantile.

To find genes differentially expressed in damaged senescent hepatocytes and not-senescent damaged hepatocytes, we used Seurat's FindMarkers function on an SC-transformed merged Seurat object. By default, the function utilizes a Wilcoxon Rank-Sum Test. FindMarkers performs *p*-value adjustment using Bonferroni correction based on the total number of genes expressed. We defined genes significantly differentially expressed if they had an adjusted *p*-value <0.05, an absolute Log2 fold change >0.5 and were expressed in at least 10% of hepatocytes of either senescent-damaged or not-senescent damaged clusters.

Murine scRNA-seq hepatocyte data from Vesting et al.,[99] was provided with quality control, normalization, and cell-type-annotation by the authors. HDS and SHGS scores, activities and distributions were calculated as described above. Damage and cell state classification was carried out in identical fashion.

QUANTIFICATION AND STATISTICAL ANALYSIS

The statistical methods for each experiment were conducted using R scripts. Details regarding datasets were provided in the figure legends, while statistical information regarding experiments and analyses were available in both the figure legends and the main text.

Cell Genomics, Volume *6*

Supplemental information

Cell-type-specific damage scores reveal kidney and liver disease trajectories in single-cell and spatial transcriptomics

Tsimafei Padvitski, Paula Unger Avila, He Chen, Cem Özel, Fabian Braun, Marie-Kristin Kroll, Christian Vossen, Peter Wienand, Linus Butt, David Unnersjö-Jess, Anna-Lena Schumacher, Kat Folz-Donahue, Heike Göbel, Christoph Kuppe, Roman-Ulrich Müller, Pål Olof Westermark, Paul Thomas Brinkkötter, Bernhard Schermer, Thomas Benzing, Frank Thomas Wunderlich, Martin Kann, and Andreas Beyer

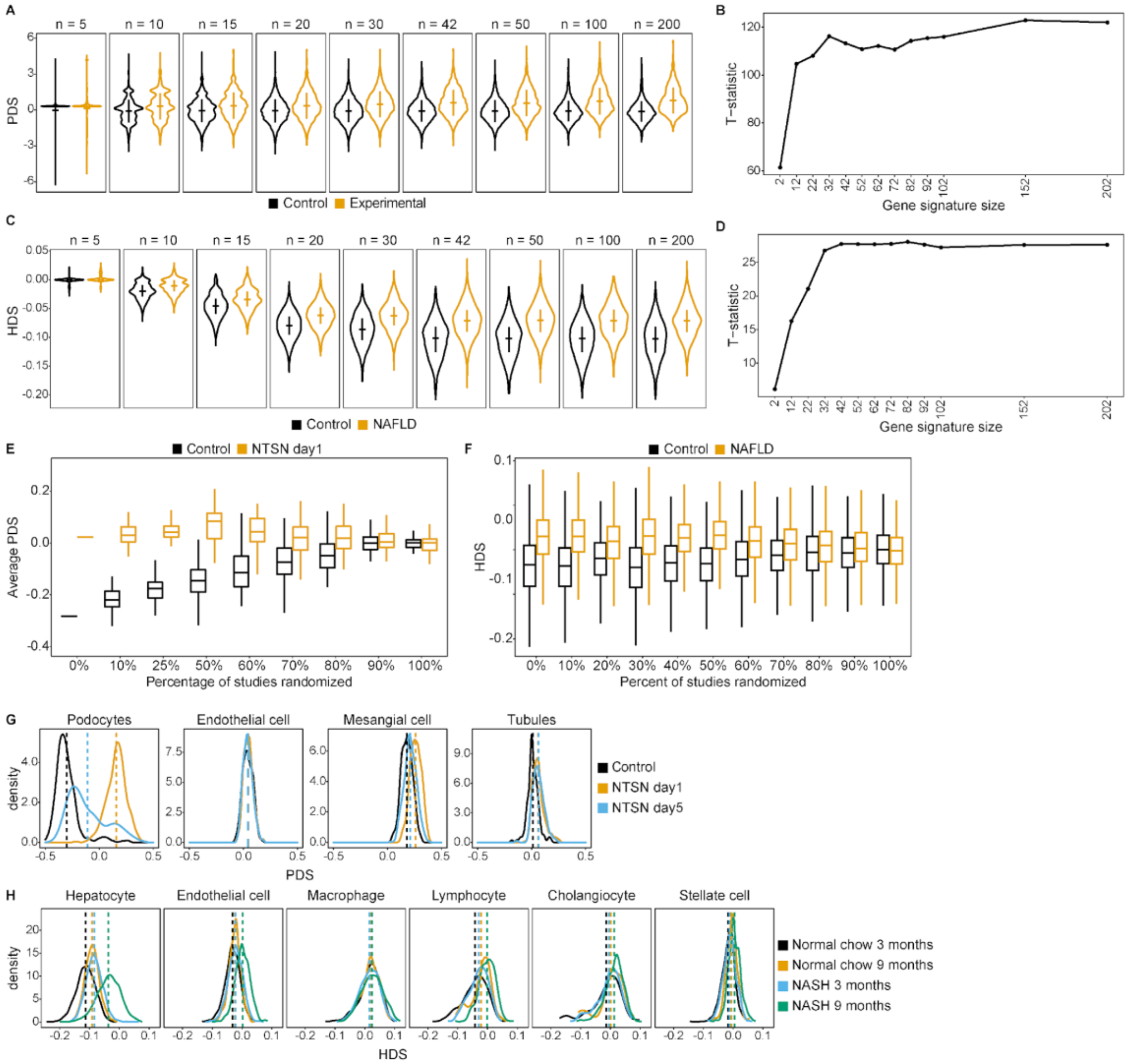

Figure S1: Robustness and specificity of PDS and HDS, related to Figure 1

(A) Signature size test for PDS. Three snRNA-seq (*Wt1*$^{ko/wt}$, *Nphs2*^{mut}, *Pdss2*^{mut}) and four scRNA-seq datasets from Chung et al.[1] were used to test for the number of genes in the PDS needed to robustly differentiate control (black) and experimental (orange) conditions. PDS were scaled per study (Y-axis). Number of top genes (*n*) used per signature is indicated above. Insets in violin plots show the mean plus/minus 2 standard deviations. (B) Signature size test shows absolute t-statistics of PDS comparisons between control and experimental samples in sc/snRNA-seq studies as a function of damage signature size (top *N* genes used; X-axis). Larger absolute t-statistics indicates stronger separation of experimental and control samples. (C) Same as in A for HDS, except HDS is not scaled. Median HDS per distribution are indicated; whiskers represent the inter quartile range (IQR). HDS calculated for snRNA-seq of hepatocytes from 24-week-old mice with and without NAFLD-inducing diet, *n* = 5 (data from Liver Cell Atlas, GSE192742[2,3]). (D) Same as in B for HDS. Signature size test shows absolute t-statistics of HDS comparisons between control and experimental samples in snRNA-seq data as a function of damage signature size (top *N* genes used; X-axis; same data as in C). Larger absolute t-statistics indicates stronger separation of experimental and control samples. (E) PDS robustness test against noise in training data. Varying fractions of the training data were randomized to test how this would reduce the ability of the PDS to successfully distinguish experimental from control conditions in independent test data (see Methods). ScRNA-seq data from nephrotoxic serum nephritis (NTSN) model of podocyte damage from Chung et al.[1] was used for the test. 50 rounds of randomization were conducted for each ‘percentage of studies randomized’. Box plots show first (Q1), second (Q2), and third quartiles (Q3) of the distribution of average PDS per cell across 50 randomization rounds per ‘percentage randomized’. Whiskers represent Q1 - 1.5 x interquartile range (IQR) and Q3 + 1.5 x IQR. (F) Same as in E, but for the HDS. Test was conducted on hepatocyte snRNA-seq (same data as in C). HDS centered per run. Median HDS per distribution is indicated; whiskers represent IQR. (G) Cell-type specificity test for the PDS. Panels show distribution of PDS in four major glomerular cell types (podocytes, endothelium, mesangium and tubules, color of lines reflect experimental condition (control and two damage time points). scRNA-seq data from the nephrotoxic nephritis model[1]. Vertical dashed lines represent the mean. (H) Cell-type specificity test for the HDS. Panels show distribution of HDS in hepatocytes and five major liver cell types from Xiao et al.[4] Cell type annotation was adopted from the authors. Colour of density lines represent the condition of the mouse (*n* = 1). Vertical dashed lines represent the mean.

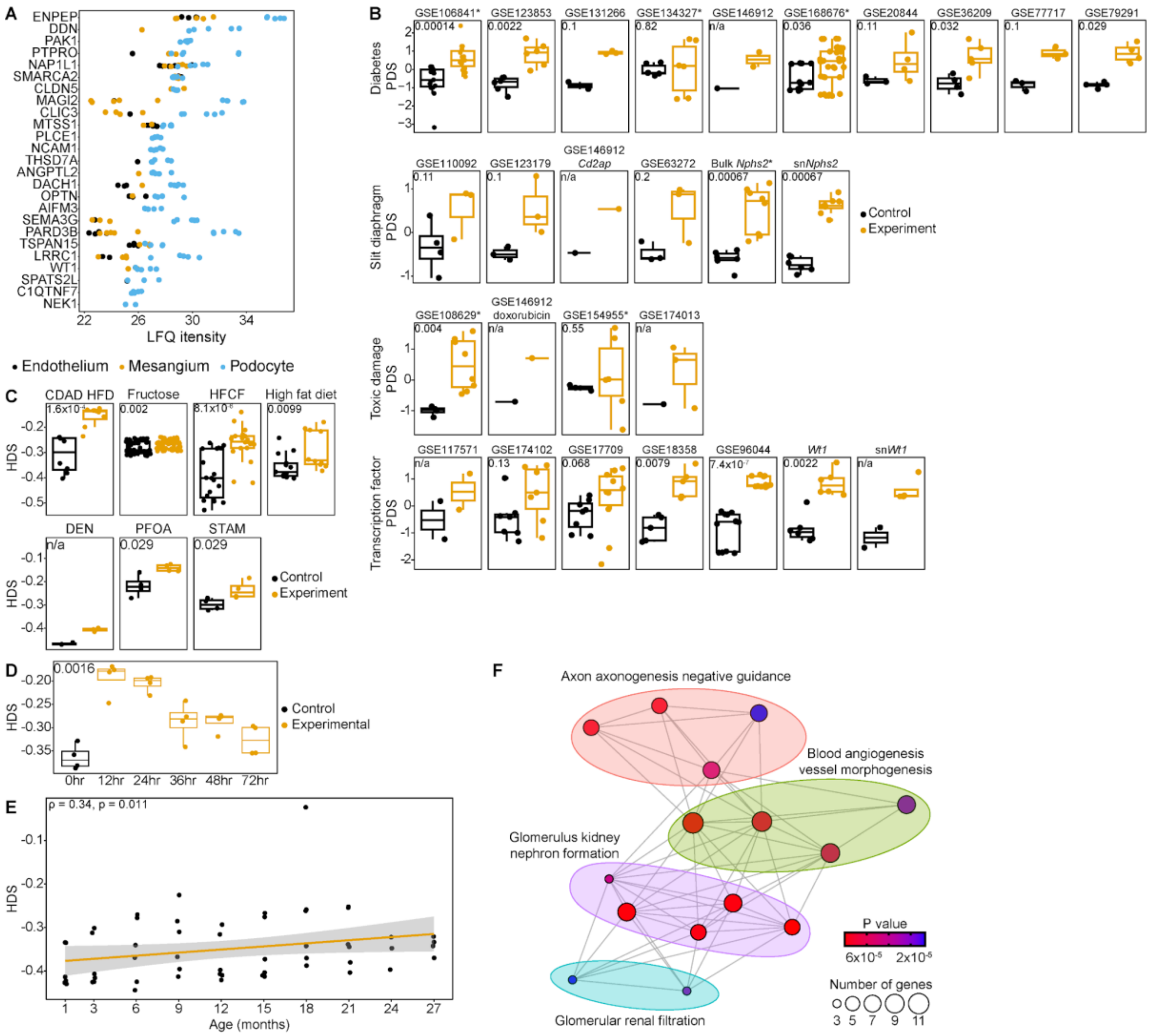

Figure S2: Cross validation of PDS and HDS, related to Figure 1.

(A) Protein expression of PDS marker genes in three types of mouse glomerular cells: podocytes (blue), endothelial cells (black) and mesangial cells (orange). Data is from glomerular proteomics as reported in PXD016238[5]. Non-scaled protein expression values (LFQ) are shown. (B) Leave-one-dataset-out cross validation of the PDS. Each dataset of the indicated group of disease models were left out during the training phase of the PDS and used for testing the indicated disease model afterwards. Each dot represents the mean PDS across all cells of a sample. PDS were scaled and centered per study. Boxplots show first (Q1), second (Q2), and third quartiles (Q3) of the distribution. Whiskers represent Q1 - 1.5 x interquartile range (IQR) and Q3 + 1.5 x IQR. Outlier cells are shown as dots. *P*-value of Wilcoxon test is shown for all studies with at least 3 samples in both conditions. For studies GSE106841[6] (ageing series in diabetes), GSE134327[8], GSE168676[10], Bulk *Nphs2*[14], GSE108629[12], and GSE154955[15] diverse experimental animals (different age, treatments etc.) were summarized as 'experiment'. (C) Leave-one-model-out cross validation of the HDS. Procedure is the same as in B. Each dot represents the average HDS of one sample. CDAD HFD: choline-deficient, L-amino acid-defined, high-fat diet (n = 8), Fructorse (n = 54), HFCF: high fat, cholesterol, and fructose diet (n: control = 19, treatment = 20), High Fat Diet (n: control = 13, treatment = 10), DEN: Diethylnitrosamine (DEN)-induced carcinogenic liver injury (n = 3), PFOA: perfluorooctanoic acid (n = 4), STAM: mouse model that recapitulates human NASH progression to HCC and uses a combination of streptozotocin and high fat diet (n = 4). Boxplots show the first (Q1), second (Q2), and third (Q3) quartiles of distribution. Whiskers show Q1 - 1.5 x IQR and Q3 + 1.5 x IQR. P-values of Wilcoxon rank test (experimental versus control conditions) are shown. (D) Cross validation of the HDS on a time course dataset. Procedure is the same as in B Each dot represents a sample in time series murine liver samples post hepatic injury induced by acetaminophen administration, n = 4 (GSE111828[7]). Labels on x-axis represent the time in hours (hr) after acetaminophen administration. Untreated control samples are labeled with 0hr. *P*-value of Kruskal Wallis test shown. (E) Cross validation of HDS on aging time course in murine liver. Each dot represents the HDS of a murine liver sample of an age group (in months on x-axis) from the Tabula Muris Senis data set, n = 6, except for 24 month (n = 3), and 27 months (n = 4) (GSE132040[9,11]). Regression line (orange with confidence interval as gray shading) shown as visual aid to highlight age-related trend. Correlation coefficient and *p*-value of Spearman correlation are indicated. (F) Functional annotation of the podocyte damage marker set confirms its relations to the key podocyte functions. GO terms were grouped by semantic similarity. Nodes are colored by *p*-value of Fisher's exact test and sized by the number of genes tested.

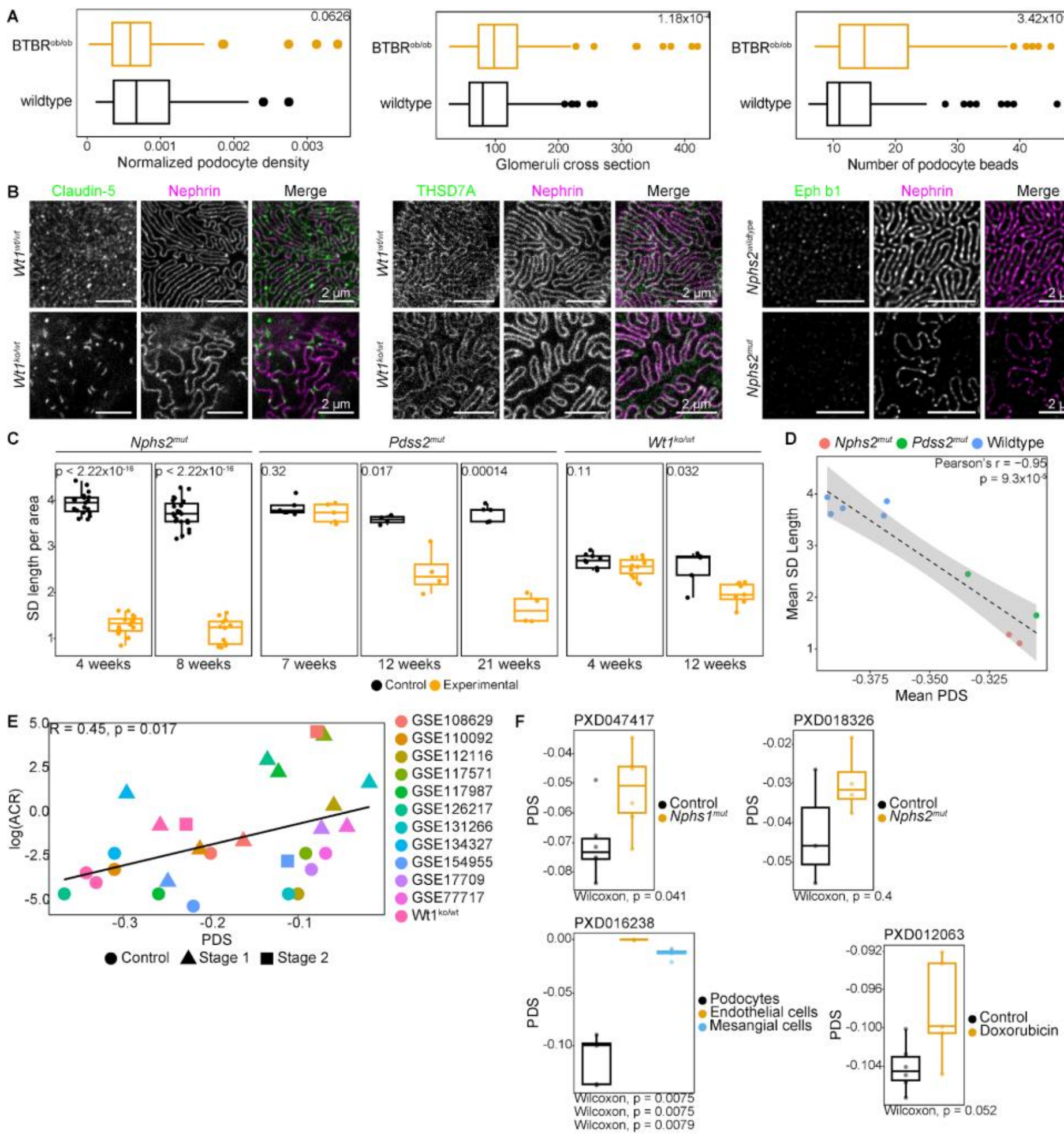

Figure S3: Functional validation of PDS, related to Figure 1.

(A) Boxplots showing glomerular size estimates, podocyte bead number, and normalized podocyte density for diabetic BTBR$^{ob/ob}$ (orange) and control mice (black) as calculated on Slide-seqV2 spatial transcriptomics data provided in GSE190094[13]. Podocyte KNN filtration, glomerular identification and size estimation was performed using the programming code of the original publication[13]. Boxplots show quartiles (Q), with whiskers representing Q1 - 1.5 x interquartile range (IQR) and Q3 + 1.5 x IQR. Outlier cells are represented by dots. Values in top right are *p*-values of Wilcoxon rank sum test of the difference between experimental and control groups. (B) STED of 12-week-old *Wt1*$^{ko/wt}$ or control mice co-stained for nephrin to visualize the slit diaphragm and PDS marker proteins including Claudin-5 and THSD7A. STED of 4-week-old *Nphs2*^{mut} or control mice was co-stained for nephrin and the Eph B1 receptor. All PDS marker proteins localize to the slit diaphragms. In *Wt1*$^{ko/wt}$ mice, Claudin-5 forms aggregates at the slit diaphragms. In *Nphs2*^{mut} mice, the expression of Eph B1 is downregulated upon podocyte damage. (C) Quantification of SD length per area for *Nphs2*^{mut} (*n* = 19 for 4-week control, *n* = 16 for 4-week experimental, *n* = 23 for 8-week control, *n* = 12 for 8-week experimental), *Pdss2*^{mut} (*n* = 10 for 7-week, *n* = 8 for 12-week, *n* = 9 for 21-week) and *Wt1*$^{ko/wt}$ (n = 9 for 4-week control, *n* = 10 for 4-week experimental, *n* = 5 for 12-week control, *n* = 7 for 12-week experimental) control and experimental animals at the indicated age. Boxplots show quartiles (Q), with whiskers representing Q1 - 1.5 x interquartile range (IQR) and Q3 + 1.5 x IQR. *P*-values of t-test are shown between control and experimental groups. (D) Correlation plot of average PDS and average SD length per area for wildtype and experimental *Nphs2*^{mut} (Grouped control: *n* = 2 for 4-week, *n* = 1 for 6-week, *n* = 1 for 8-week, *n* = 2 for 12-week; Grouped experimental: *n* = 2 for each of 4-week, 6-week, 8-week and 12-week) and *Pdss2*^{mut} (Grouped control: *n* = 2 for each of 6-week, 12-week and 21-week; Groupe experimental: *n* = 2 for 6-week, *n* = 2 for 12-week and *n* = 1 for 21-week) mouse groups. Linear regression of PDS correlated with SD length shown in dashed line with 95% confidence interval colored in grey. Coefficient and *p*-value for Pearson's correlation are shown. (E) Correlation of urinary albumin/creatinine ratio (ACR) and PDS in bulk microarray and bulk RNA-seq data. *Wt1*$^{ko/wt}$ represents bulk RNA-seq data published[14]. Stage 1 and 2 indicate datasets, in which longitudinal ACR data was available. Coefficient and *p*-value for Pearson's correlation are shown. (F) Boxplots show PDS calculated for various conditions and genotypes in 4 proteomics datasets. Individual samples are shown as dots. *P*-value of Willcoxon test of condition/genotype effect are provided below each panel. ProteomeXchange identifiers (PXDs) of the corresponding datasets are indicated[5,16–18]. Boxplots show quartiles (Q), with whiskers representing Q1 - 1.5 x interquartile range (IQR) and Q3 + 1.5 x IQR. Outlier cells are represented by dots. *P*-values of Wilcoxon rank sum test of the difference between experimental and control groups are shown.

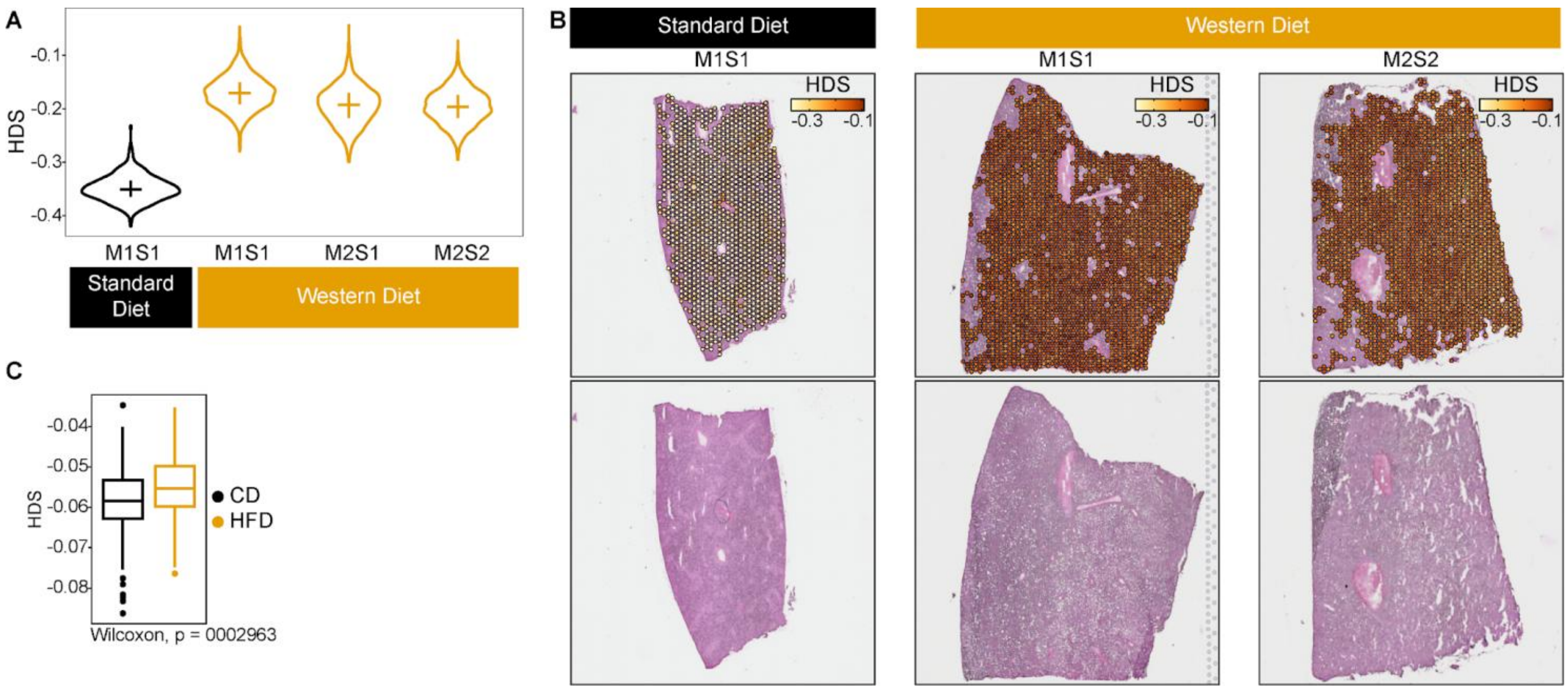

Figure S4: Functional validation of the HDS, related to Figure 1.

(A) Violin plots of HDS distribution in all murine spatial transcriptomic 10X Visium samples from the Liver Cell Atlas (GSE192742[2,3]) comparing western (*n* = 3) to standard diet (*n* = 1). M1S1 = Mouse 1 Sample 1, M2S1 = Mouse 2 Sample 1, M2S2 = Mouse 2 Sample 2. Horizontal lines show the median and vertical lines the IQR of the distributions. Statistical comparison not appropriate due to *n* = 1 per "Standard Diet" group. (B) 10X Visium spatial transcriptomics of murine liver from the Liver Cell Atlas (GSE192742[2,3]). Spatial plots of HDS distribution in spatial transcriptomic not previously shown in Figure 1. Color range: floor = -0.37, ceiling = -0.09. *P*-value for Wilcoxon test shown. (C) Confirmation of HDS on murine liver proteomics data[19]. Mice fed a high fat diet (HFD) compared to mice fed a control diet (CD), (*n* = 315 across conditions, different strains, age range between 157 and 785 days). *P*-value for one-sided Wilcoxon test is shown.

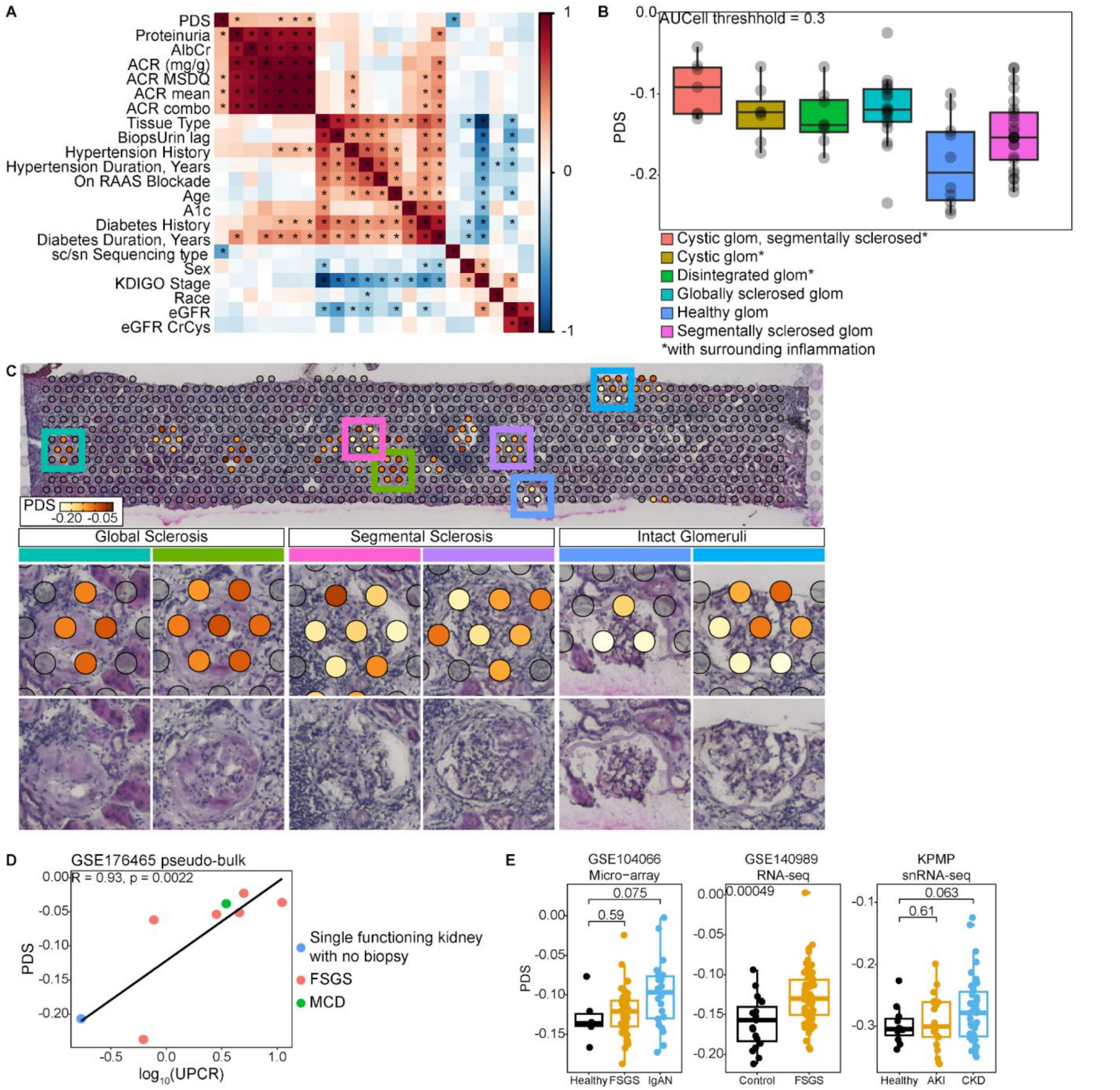

Figure S5: Validation of the PDS on human data, related to Figure 2.
(A) Heatmap of spearman correlation between PDS and detailed clinical traits from patients with acute kidney injury (AKI) and CKD. Anonymized patient data, sc/snRNA-seq data used in the analysis were retrieved from the KPMP database. Stars denote cells with *p*-value of Spearman correlation < 0.05. (B) Boxplot shows PDS in further glomeruli as annotated manually in the histology image shown in the next panel, PDS measurements based on KPMP spatial transcriptomics dataset,[20] patient ID 29-10282, accessed at https://atlas.kpmp.org/. (C) 10X Visium spatial transcriptomic data on a human kidney biopsy from the KPMP registry (patient ID 29-10282)[20] showing an FSGS phenotype. PDS values were computed only for Visium spots with podocyte signals as determined by *WT1* and *NPHS2* expression. Selected glomeruli are marked in the upper panel with respective colors. High magnification images show various degrees of glomerular sclerosis with PDS gradients corresponding to degree of sclerosis. See boxplots for PDS distribution as by glomerular damage in the previous panel. (D) Relationship between PDS calculated on human urinary podocyte snRNA-seq data and urinary protein creatinine ratio (UPCR) in patients suffering from either minimal change disease (MCD) or focal and segmental glomerulosclerosis (FSGS) as reported in GSE176465[21]. Data from 8 patients with at least three measured podocytes each was used. (E) Validation of PDS on human transcriptomic data obtained from healthy patients (CON) and from patients suffering from FSGS, IgA nephropathy (IgAN), acute kidney injury (AKI), and chronic kidney disease (CKD). Samples were acquired and reported in transcriptomic modalities and studies as indicated on top of the plots. Podocytes from snRNA-seq datasets were aggregated in pseudo-bulk samples. *P*-values of Wilcoxon rank sum test comparing experimental and control samples are shown.

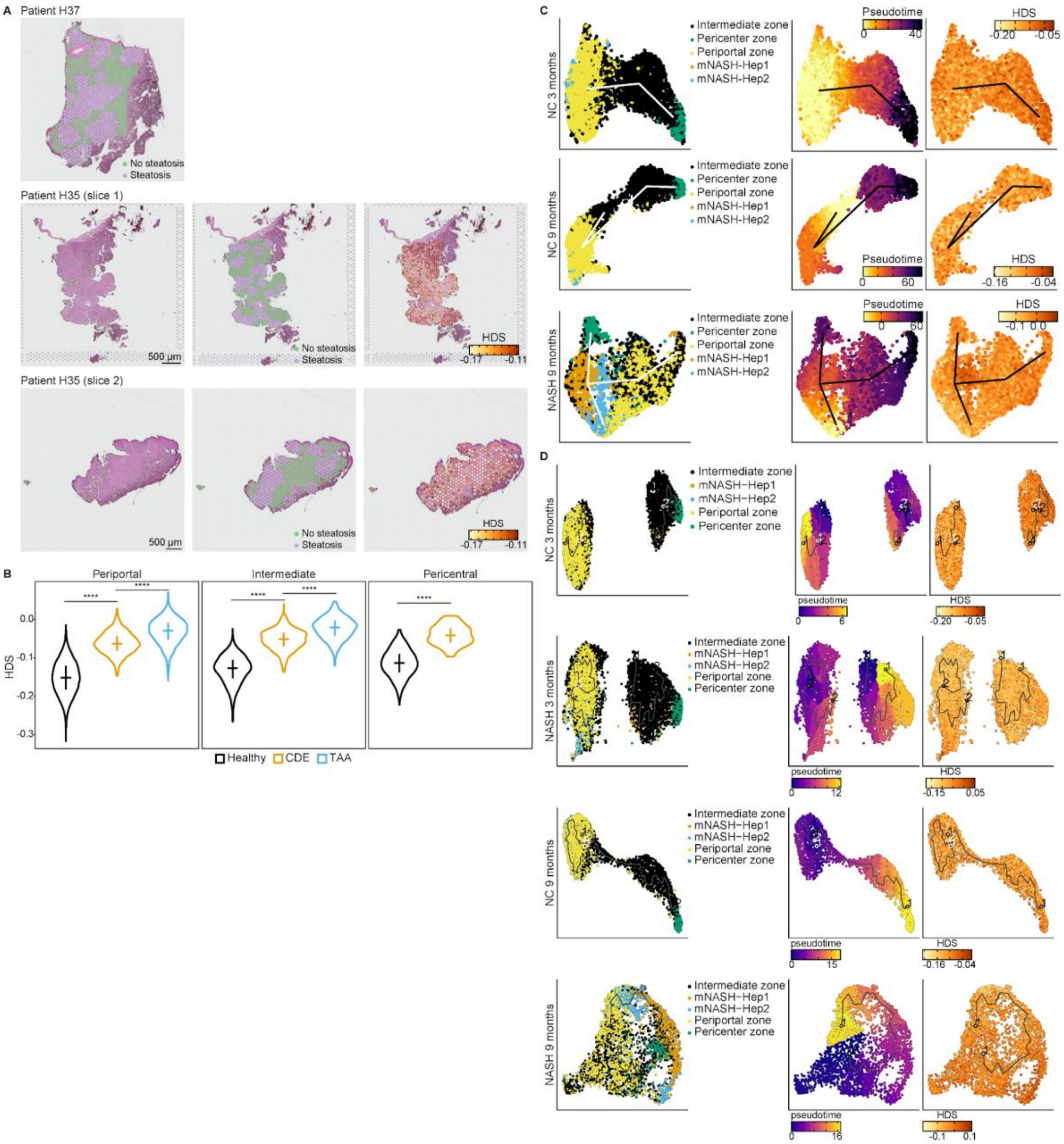

Figure S6: Validation of HDS using single nucleus and spatial transcriptomics, related to Figure 3.
(A) HDS calculation on 10X Visium human spatial transcriptomic data from the Liver Cell Atlas (GSE192742[2,3]). Only subjects with liver steatosis were analyzed. Top panel: manual assignment of steatosis vs non-steatosis areas as used for Figure 3D. Bottom two rows: Left panel: histology, Middle panel: assignment of steatosis vs n-steatosis areas, right panel: HDS per Visium spot overlay. Spots representing hepatocytes were manually annotated. Color scale represents the 10%- and 90%-quantiles of the HDS distribution across all samples. Spots in regions with steatosis: light purple, spots in no-steatosis regions: green. (B) HDS distributions by liver zonation in snRNA-seq data from mice subjected to three different treatments. Healthy: normal chow; CDE: choline-deficient, ethionine-supplemented (CDE) diet; TAA: thioacetamide (TAA) in the drinking water (GSE200366[22]) (n = 3 biological replicates per treatment). Same data is in Figure 6A. Hepatocyte zone annotation as provided by authors of data set. Pericentral hepatocytes were not detected in TAA-treated animals. Horizontal lines show median of the distribution and vertical lines represent the IQR. **** = $p < 2 \times 10^{-16}$ in one sided Wilcoxon rank-sum test. (C) Analysis of HDS versus pseudo-time trajectories with TSCAN. UMAPs for further samples from the same study as shown in Figure 3G. Each dot represents a hepatocyte colored either by liver zone (left), pseudo-time (PT) (middle) and HDS (right). Hepatocyte zone annotation from Xiao et al.[4] MNASH-Hep1/2: classification from original publication corresponding to damaged hepatocytes whose zone of origin could not be determined according to the authors. PT trajectory inference is based on clusters identified using Seurat after quality control cell filtering, normalization with Seurat's SCTransform and dimensionality reduction. PT is dominated by liver zonation, while the HDS is independent of it. Top: Normal Chow 3-month-old, middle: NASH-inducing diet 9-month-old (n = 1), bottom: Normal Chow 9-month-old mouse. (D) Analysis of HDS versus pseudo-time trajectories with Monocle3. Same data and legends as in C. Trajectory inference is based on clusters identified using Monocle3 default processing and clustering pipeline. PT trajectories and clustering are dominated by liver zonation also in Monocle3 analysis. HDS is not reflected by PT trajectories.

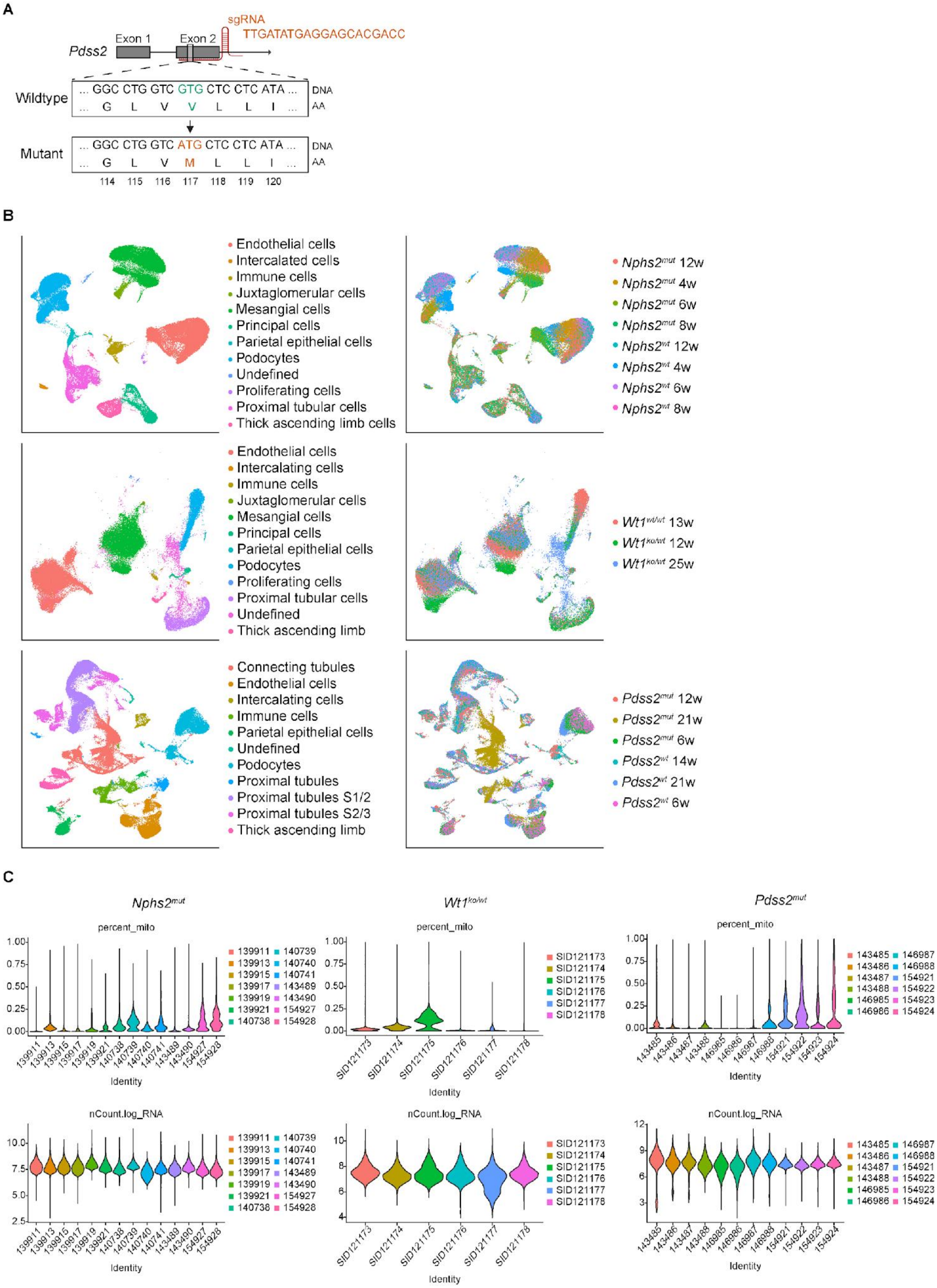

Figure S7: Glomerular snRNA-seq of *Nphs2^mut^*, *Wt1^ko/wt^* and *Pdss2^mut^* mice, related to Figure 4.

(A) CRISPR strategy for the generation of the *Pdss2*^{mut} mice. Exon 2 of *Pdss2* is targeted using single guide RNA (sgRNA) to generate a point mutation which exchanges valine (V) at amino acid (AA) position 117 to methionine (M). (B-C) Overview of snRNA-seq data generated for this study. (B) UMAP of preprocessed snRNA-seq data colored by cell types (left) or condition (right) of *Nphs2*^{mut} (top), *Wt1*$^{ko/wt}$ (middle) or *Pdss2*^{mut} (bottom) datasets. UMI counts corrected for ambient RNA, filtered and normalized (see Methods), are used for this and the next panels. (C) Percent mitochondria (top) and log

transformed number of RNA counts (bottom) for individual snRNA-seq samples of *Nphs2*^{mut} (left), *Wt1*$^{ko/wt}$ (middle) or *Pdss2*^{mut} (right) datasets.

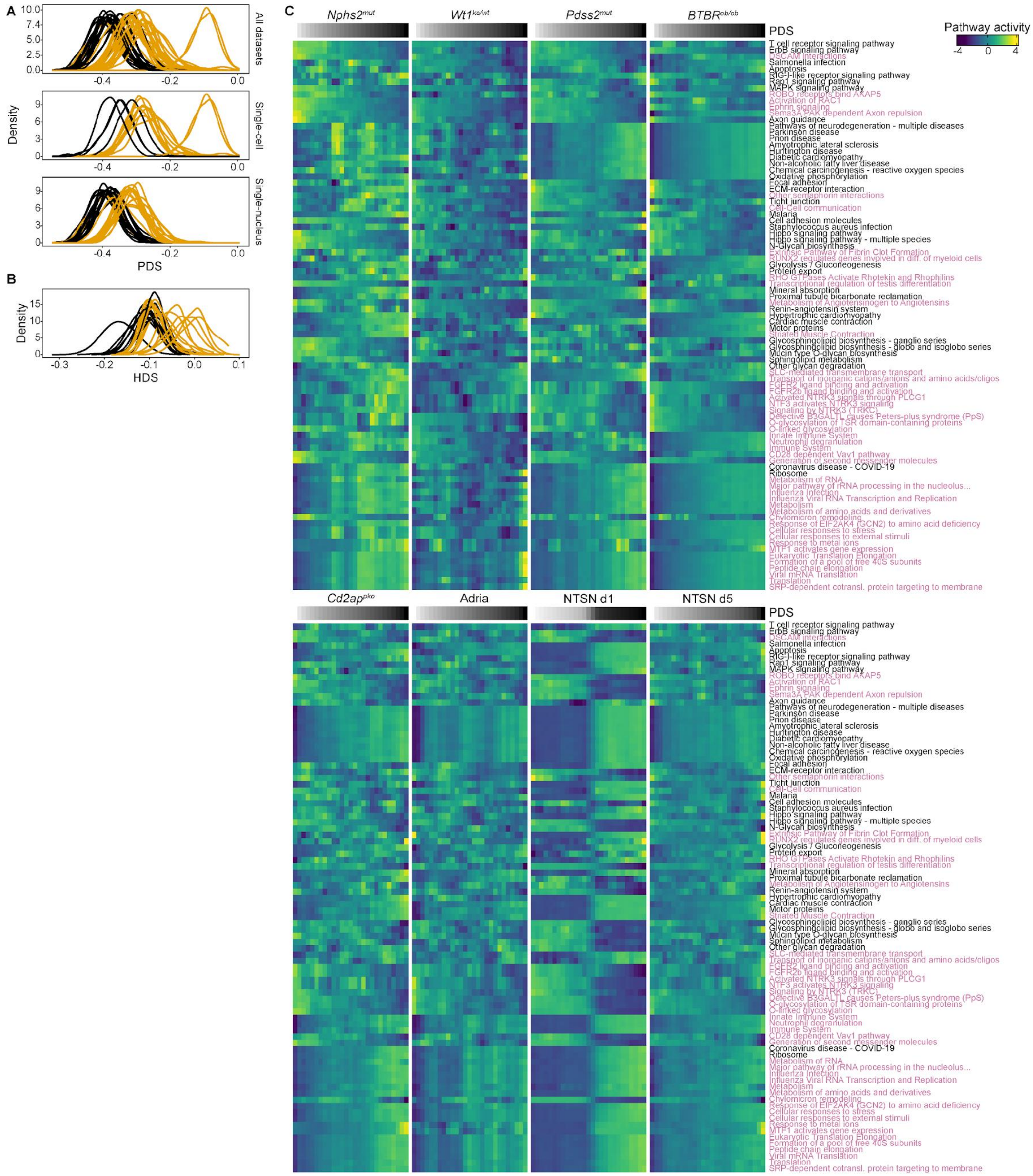

Figure S8: Pathway fingerprints of all FSGS sc/sn-RNA-seq models, related to Figure 4.
(A) Density plots show greater variation of PDS in experimental compared to control samples. PDS densities of individual samples are aligned by x-axis. Black: controls, orange: experimental conditions. Top panel shows results for all sc/snRNA-seq samples, bottom two panels show results for single-cell and single-nuclei datasets separately. Wilcoxon Rank Sum test confirms (p-value = $2.5\text{x}10^{-11}$) that controls are significantly better aligned than experimental samples (see method section Damage score alignment test). (B) Density plots show greater variation of HDS in experimental compared to control samples. HDS densities from three sn/scRNA-seq studies across different liver damage models (NAFLD, NASH, ageing, GSE192742[2,3], GSE200366[22], GSE189600[4]). Black: controls, orange: experimental samples. Wilcoxon rank sum test confirms that control samples are aligned significantly better than experimental samples (p-value = $2.7\text{x}10^{-4}$). (C) Full PDS pathway fingerprints for sn/scRNA-seq data from all studies analyzed. Cells/nuclei were sorted by increasing PDS (left to right, gray color scale). Pathway activities for KEGG (black) and Reactome (magenta) pathways were calculated using AUCell (see Methods). A total of 85 pathways - a union of 10 pathways that most significantly (adjusted p-value < 0.05) correlate with PDS in each model - is used.

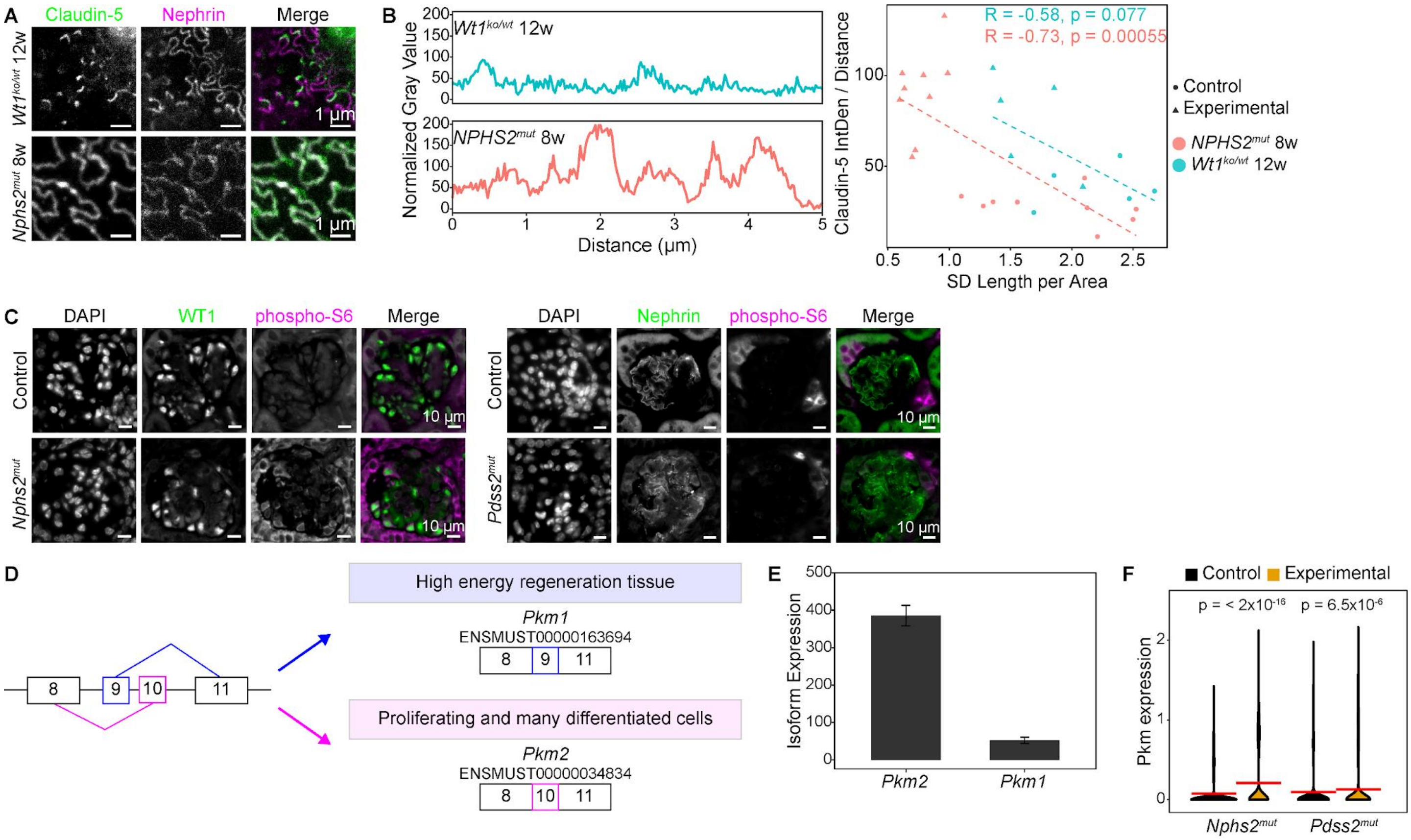

Figure S9: Validation of the PDS pathway fingerprints, related to Figure 4.

(A) Representative STED microscopy of 12-week-old $Wt1^{ko/wt}$ (n = 5 for control, n = 5 for experimental) or 8-week-old $Nphs2^{mut}$ (n = 9 for control, n = 9 for experimental) kidneys co-stained for claudin-5 (green) and nephrin (magenta) to assess the aggregation of claudin-5 at the podocyte slit diaphragm. (B) Representative normalized line profiles of claudin-5 fluorescence intensity along manually traced slit diaphragms, illustrating differences in intensity and focal aggregation of claudin-5 along equal-length segments in $Nphs2^{mut}$ (8-week-old) and $Wt1^{ko/wt}$ (12-week-old) experimental mice. Right: Correlation plot of normalized claudin-5 fluorescence intensity to average SD length per area for 8-week-old $Nphs2^{mut}$ (n = 9 for control, n = 9 for experimental) and 12-week-old $Wt1^{ko/wt}$ (n = 5 for control, n = 5 for experimental) control and experimental animals. Correlation coefficient and p-values in Spearman are shown. (C) Representative immunofluorescence staining of control and experimental kidney tissue from either $Nphs2^{mut}$ 8-week-old (left, n = 3 for control, n = 3 for experimental) or $Pdss2^{mut}$ 21-week-old (right, n = 2 for control, n = 2 for experimental) showing DAPI (blue), nephrin (green) and phospho-S6 (magenta). Phospho-S6 expression is upregulated in podocytes in $Nphs2^{mut}$ mice. (D) Cartoon of *Pkm1* and *Pkm2* isoforms and related dominant isoforms in respective cells or tissues. *Pkm2* is the dominant isoform in podocytes. (E) Barplot of the expression of *Pkm1* and *Pkm2* isoforms in healthy mouse glomeruli, from bulk glomerular RNA-seq of $Nphs2^{mut}$ mice. Error-bars indicate 95% confidence intervals. (F) Violin plot of expression of *Pkm* in murine podocytes from $Nphs2^{mut}$ and $Pdss2^{mut}$ snRNA-seq experiments. Red horizontal lines indicate mean. *P*-value of Wilcoxon test of the genotype effect is displayed.

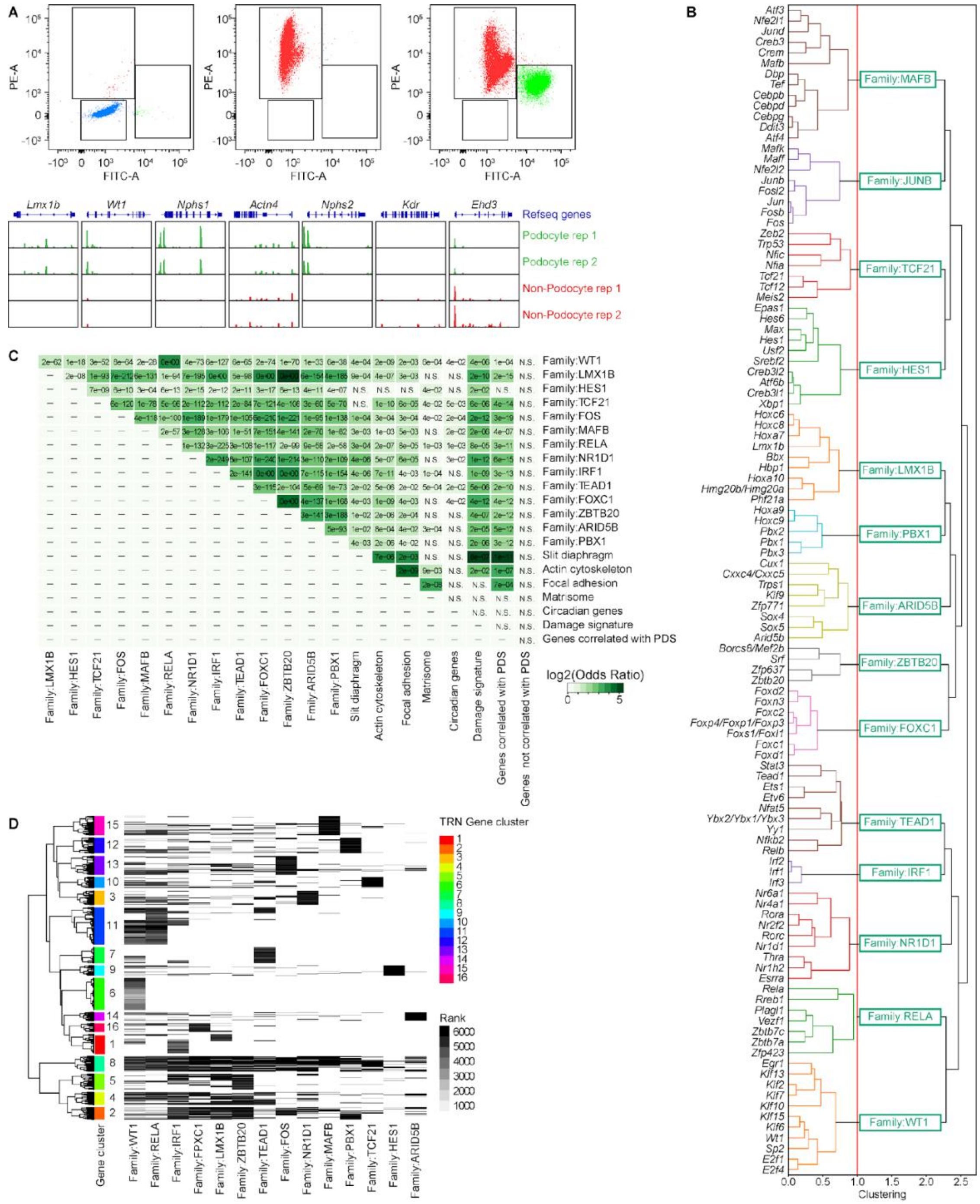

Figure S10: Development of the podocyte-specific transcription regulatory network and identification of podocyte damage pathways, related to Figure 5.

(A) Top: Flow-cytometry bivariate plots of glomerular nuclei extracted from nTnG;Pod:cre mice that express no nuclear fluorescence reporter (left), only tdTomato+ reporter (middle) or both GFP+/tdTomato+ reporter (right). Sorted nuclei were collected and used for the generation of cell type-specific ATAC-seq (see Methods). Bottom: genome browser tracks at podocyte-specific loci (*Lmx1b*, *Wt1*, *Nphs1*, *Actn4*, *Nphs2*) show accessible chromatin in podocytes, whereas those at other glomerular cell type-specific loci (*Kdr*, *Ehd3*) show accessible chromatin in non-podocytes. (B) Dendrogram of hierarchical clustering of TF motifs (PWMs). The dendrogram is cut (red line) to produce 14 TF clusters (families), each one is named by the representative motif. (C) Heatmap shows podocyte specific TRN: 14 regulatory TF-clusters (each representing a group of TFs with similar PWMs) are in columns and 6360 of their target genes expressed in podocytes are in rows. Shades of grey color shows ranks of predicted interaction score (from TFtargetCaller prediction), black and white color means the strongest and no predicted interaction, corresponding dendrogram on the left shows hierarchical clustering of genes. The dendrogram is cut to produce 16 clusters (annotated with rainbow colors), that represent common and unique targets of each of 14 regulatory modules. (D) Heatmap of overlaps of 14 TF regulons and podocyte gene sets used in Figure 5B. Color shows log2 odds ratio of the Fisher's exact test, which represents the strength of association, cell labels show labeled with adjusted P-values of the test (if *padj* < 0.05, otherwise "NS").

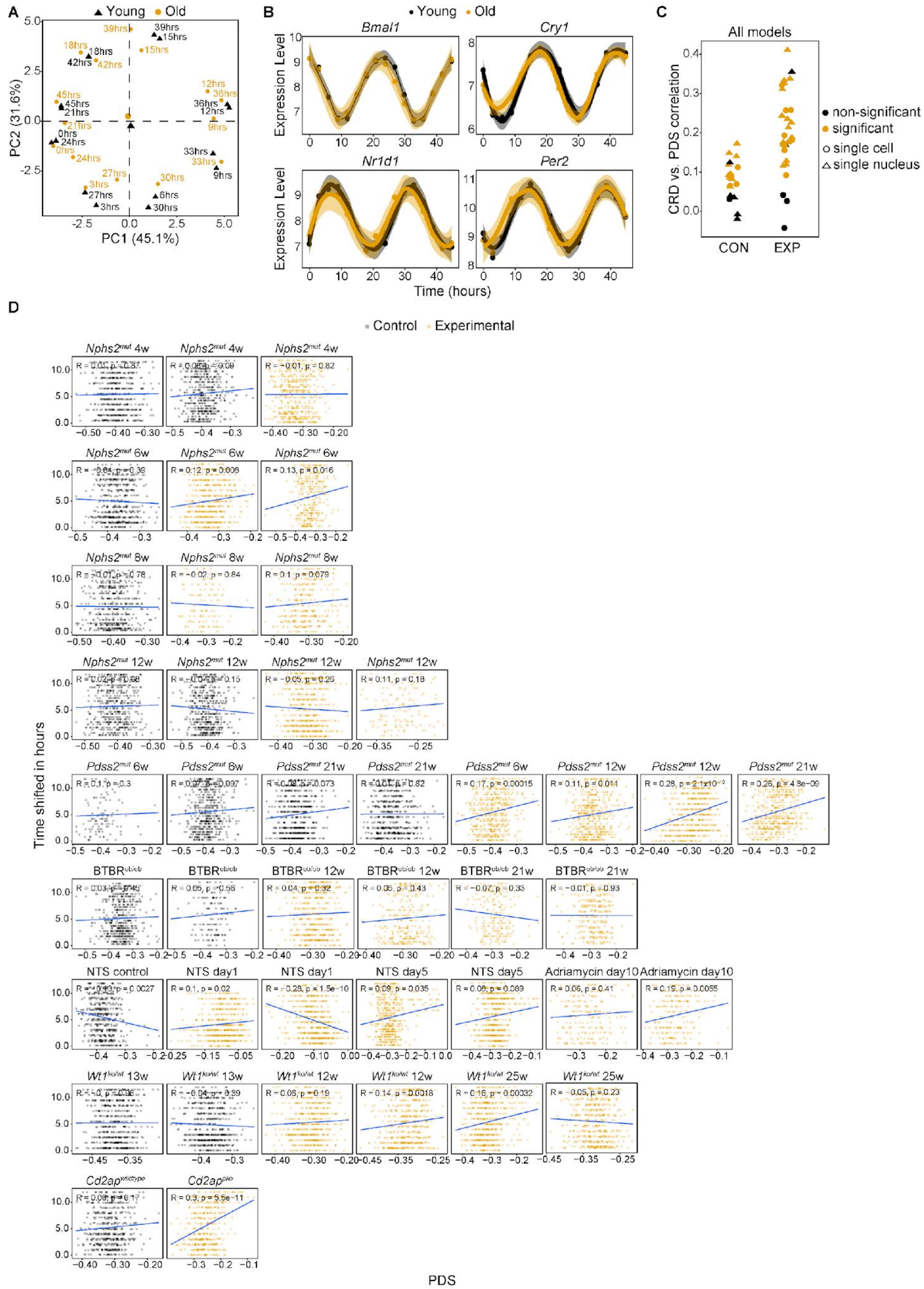

Figure S11: Transcriptome analysis of circadian gene expression, related to Figure 5.
(A) PCA of circadian bulk RNA-seq samples from whole kidneys. Each symbol represents a pooled replicate of 3 individual mice (yellow: age 78 weeks, black: age 8 weeks). Samples were taken at zeitgeber times 0, 3, ... 21 hours during 12h/12h light–dark cycles. Replicates per group (n = 2) are presented in serial fashion resulting in a 48h period. (B) Expression of circadian genes *Bmal1*, *Cry1*, *Nr1d1*, and *Per2* in the same dataset as in panel a. Y-axis - gene expression value normalized by DEseq2 rlog function, X-axis – zeitgeber time, in hours. Replicates per group (n = 2) are represented in serial fashion resulting in a 48h period. Solid line indicates an estimate of the conditional mean function, shades indicate 95% confidence interval of estimates. Yellow: age 78 weeks, black: age 8 wks. (C) Coefficients of Spearman rank correlation between PDS and CRD calculated based on podocyte-specific circadian genes in Preston et al.[23], for all individual control (CON) and experimental (EXP) samples of seven different mouse models of podocyte damage. Color distinguishes if the CRD to PDS correlation was significant (p < 0.05). (D) Absolute deviation between circadian time of each cell from the reference sample time (Y-axis) of the respective sample as a function of the PDS (X-axis). Regression line and parameters for Spearman rank correlation are shown. Black and orange color of dots distinguish cells of control and experimental samples. Circadian time was calculated using a Tempo algorithm,[24] *Clock* was used as a reference gene.

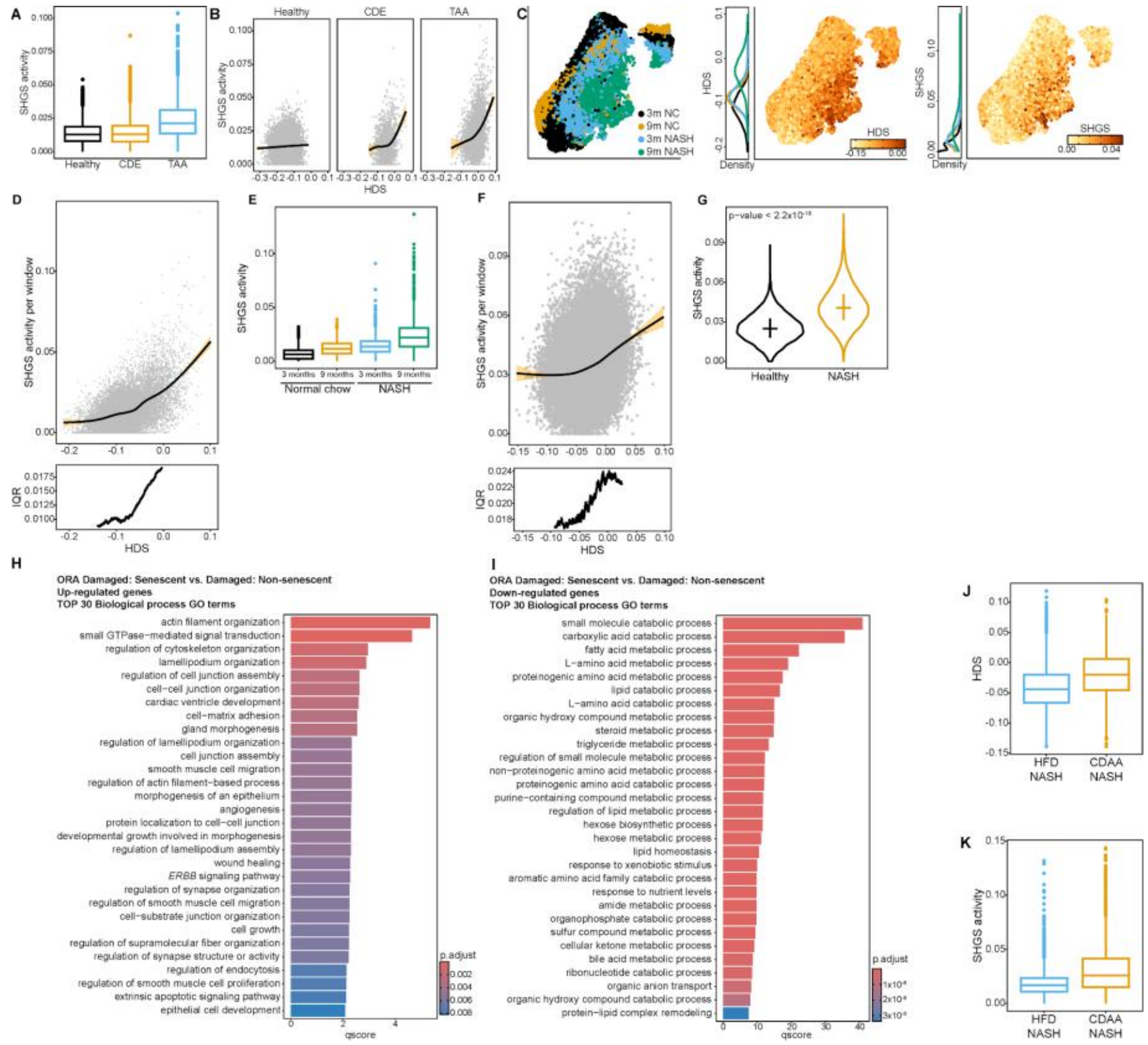

Figure S12: Analysis of hepatocyte cell state transitions, related to Figure 6.
(A) Boxplots showing activity distributions of the Senescent Hepatocyte Gene Signature (SHGS) in snRNA-seq data of mice subjected to three different treatments: Healthy: normal chow; CDE: choline-deficient, ethionine-supplemented (CDE) diet; TAA: thioacetamide (TAA) in the drinking water (GSE200366,[22] n = 3 biological replicates per treatment, same data as in Figure 6A). Wilcoxon rank sum test for both, comparison of TAA to controls and TAA to CDE $p < 2 \times 10^{-16}$. Wilcoxon rank sum test for comparison of CDE to controls was not significant (p-value = 0.052). (B) SHGS activity score versus HDS, separated by model. Same data as in Figure 6A-6B and panel A, but separated by condition. Each dot represents a hepatocyte. Solid line: Generalized linear model line showcases behavior of SHGS activity with increasing HDS. A tipping point pattern with consistent HDS threshold level is evident. Orange: confidence interval of regression line. X-axis represents the same range of HDS for all three plots. (C) UMAP of merged snRNA-seq samples from mice held in four different conditions (n = 1): Black: 3-month-old fed normal chow (3m NC); Orange: 9-month-old fed normal chow (9m NC): Light blue: 3-month-old fed NASH inducing diet (3m NASH); Green: 9-month-old fed NASH inducing diet (9m NASH). Left: UMAP of hepatocytes colored by condition. Middle: hepatocytes colored by HDS. The color range for the HDS has floor and ceiling values corresponding to the 1%- and 99%-quantiles of the HDS distribution across all samples. Right: Hepatocytes colored by SHGS activity score.[25] Density curves of HDS and SHGS activity score are shown per condition, color of line represents condition. Significance tests not appropriate because of sample size (n = 1). (D) SHGS activity score versus HDS in NASH murine snRNA-seq (same data as in panel C). Top: SHGS activity score for each hepatocyte. Solid line: Generalized linear model line showcases behavior of SHGS activity with increasing HDS. Orange: confidence interval of regression line. Bottom: IQR of SHGS activity calculated per 2000-hepatocytes sliding window (y-axis) plotted against mean HDS per sliding window. (E) Boxplots show distribution of SHGS activity per hepatocyte in each condition (n = 1). Same data and legend as in panel C. Statistical significance test not possible due to n = 1. (F) SHGS activity score versus HDS in human snRNA-seq data set of healthy and NASH patients (n = 3, same dataset as in Figure 3A). Top: SHGS activity score for each hepatocyte. Solid line: Generalized linear model line showcases behavior of SHGS activity with increasing HDS. Orange: confidence interval of regression line. Bottom: IQR of SHGS activity calculated per 2000-hepatocytes sliding window (y-axis) plotted against mean HDS per sliding window. (G) Violin plots show distribution of SHGS activity for all hepatocytes per patient health status (black: healthy, orange: NASH, n = 3). Same dataset as in Figure 3A. Horizontal lines mark the median, and vertical lines the IQR. (H) Biological Process (BP) Gene Ontology (GO) over-representation analysis (ORA) of genes significantly differentially up-regulated (p-value < 0.05 and LFC < -0.05) in damaged, senescent vs. Damaged, not-senescent hepatocytes. ORA results were summarized to avoid redundancy using the ClusterProfiler[26] simplify() function with default settings. Bar plot includes top 30 terms kept after filtering for adjusted p-value < 0.01. (I) BP-GO-ORA of genes significantly differentially down-regulated (p-value < 0.05 and LFC > 0.05) in damaged-senescent vs. damaged-not-senescent hepatocytes. ORA results were summarized, and bar plot created, as describe in H. (J) Boxplots of HDS distribution computed on snRNA-seq data from murine samples subject to either HFD-NASH or CDAA-NASH condition (n = 1).[27] (K) Boxplots of SHGS activity distribution computed on snRNA-seq data from murine samples subject to either HFD-NASH or CDAA-NASH condition (n = 1).[27]

Seq names	start	end	width	strand	RefSNP_id	Alleles As ambig	Genome compat	Ref allele	Alt alleles	hit	Gene symbol	Damage signature	withinGeneBody
13	71773564	71773564	1	-	rs626277	N	TRUE	A	C,G,T	TRUE	*DACH1*	PDS	TRUE
4	16155043	16155043	1	-	rs317865	D	TRUE	T	A,G	TRUE	*FGFBP1*	PDS	FALSE
6	53782834	53782834	1	+	rs149051137	H	TRUE	C	A,T	TRUE	*LRRC1*	PDS	FALSE
12	15172097	15172097	1	+	rs10846157	H	TRUE	A	C,T	TRUE	*PTPRO*	PDS	FALSE
3	77398225	77398225	1	+	rs28378063	R	TRUE	A	G	TRUE	*ROBO2*	PDS	FALSE
7	83414458	83414458	1	-	rs143538480	R	TRUE	A	G	TRUE	*SEMA3E*	PDS	FALSE
5	122438887	122438887	1	+	rs149641852	K	TRUE	G	T	TRUE	*SNCAIP*	PDS	TRUE
7	11578298	11578298	1	-	rs74614630	Y	TRUE	T	C	TRUE	*THSD7A*	PDS	TRUE
7	11586025	11586025	1	-	rs17574506	H	TRUE	C	A,T	TRUE	*THSD7A*	PDS	TRUE
11	116830406	116830406	1	-	rs2070669	V	TRUE	C	A,G	TRUE	*APOA4*	HDS	FALSE
11	116832955	116832955	1	-	rs4225	K	TRUE	G	T	TRUE	*APOA4*	HDS	FALSE
11	116937254	116937254	1	-	rs473172	R	TRUE	A	G	TRUE	*APOA4*	HDS	FALSE
7	80545045	80545045	1	+	rs139761834	Y	TRUE	T	C	TRUE	*CD36*	HDS	TRUE
7	80656687	80656687	1	+	rs75326924	Y	TRUE	C	T	TRUE	*CD36*	HDS	TRUE
1	196729333	196729333	1	+	rs6689009	R	TRUE	A	G	TRUE	*CFHR3*	HDS	FALSE
1	196752801	196752801	1	+	rs371960809	W	TRUE	A	T	TRUE	*CFHR3*	HDS	FALSE
1	196706226	196706226	1	+	rs2300429	R	TRUE	G	A	TRUE	*CFHR3*	HDS	FALSE
22	23992517	23992517	1	-	rs140287	V	TRUE	G	A,C	TRUE	*DDT*	HDS	TRUE
22	24126177	24126177	1	-	rs73167538	H	TRUE	A	C,T	TRUE	*DDT*	HDS	TRUE
22	24175884	24175884	1	-	rs75303441	R	TRUE	G	A	TRUE	*DDT*	HDS	TRUE
22	24143401	24143401	1	-	rs117001360	R	TRUE	G	A	TRUE	*DDT*	HDS	TRUE
22	24066229	24066229	1	-	rs112234981	R	TRUE	A	G	TRUE	*DDT*	HDS	TRUE
22	24132091	24132091	1	-	rs735863	Y	TRUE	T	C	TRUE	*DDT*	HDS	TRUE
22	24087164	24087164	1	-	rs78365374	D	TRUE	G	A,T	TRUE	*DDT*	HDS	TRUE
22	24072436	24072436	1	-	rs17854874	R	TRUE	G	A	TRUE	*DDT*	HDS	TRUE
22	24178086	24178086	1	-	rs144524781	R	TRUE	G	A	TRUE	*DDT*	HDS	TRUE
22	24008132	24008132	1	-	rs405597	M	TRUE	A	C	TRUE	*DDT*	HDS	TRUE
22	24114019	24114019	1	-	rs111736007	Y	TRUE	C	T	TRUE	*DDT*	HDS	TRUE
22	24142922	24142922	1	-	rs116954205	Y	TRUE	C	T	TRUE	*DDT*	HDS	TRUE
22	24209757	24209757	1	-	rs139650685	R	TRUE	A	G	TRUE	*DDT*	HDS	TRUE
22	24169000	24169000	1	-	rs149418007	R	TRUE	G	A	TRUE	*DDT*	HDS	TRUE
22	24101661	24101661	1	-	rs4820582	S	TRUE	G	C	TRUE	*DDT*	HDS	TRUE
22	24080848	24080848	1	-	rs529389498	Y	TRUE	T	C	TRUE	*DDT*	HDS	TRUE
22	24152950	24152950	1	-	rs531196227	D	TRUE	G	A,T	TRUE	*DDT*	HDS	TRUE

22	24099093	24099093	1	-	rs552616617	B	TRUE	T	C,G	TRUE	*DDT*	HDS	TRUE
22	24111668	24111668	1	-	rs78960173	Y	TRUE	T	C	TRUE	*DDT*	HDS	TRUE
13	113849020	113849020	1	-	rs6602909	B	TRUE	T	C,G	TRUE	*GAS6*	HDS	TRUE
13	113842344	113842344	1	-	rs9577924	R	TRUE	A	G	TRUE	*GAS6*	HDS	TRUE
13	113862741	113862741	1	-	rs6602904	Y	TRUE	C	T	TRUE	*GAS6*	HDS	TRUE
16	81826596	81826596	1	+	rs76424446	S	TRUE	C	G	TRUE	*HSD17B2*	HDS	FALSE
15	90228773	90228773	1	+	rs11547962	V	TRUE	G	A,C	TRUE	*IQGAP1*	HDS	FALSE
15	90211624	90211624	1	+	rs1533325	Y	TRUE	C	T	TRUE	*IQGAP1*	HDS	FALSE
15	90275237	90275237	1	+	rs12595409	Y	TRUE	C	T	TRUE	*IQGAP1*	HDS	FALSE
17	48065680	48065680	1	-	rs7209484	B	TRUE	T	C,G	TRUE	*SCRN2*	HDS	FALSE
5	172438076	172438076	1	-	rs10067250	S	TRUE	G	C	TRUE	*STK10*	HDS	FALSE
17	1921011	1921011	1	-	rs4790292	M	TRUE	C	A	TRUE	*TLCD2*	HDS	FALSE

Table S3: Kidney and liver disease GWAS associations of podocyte and hepatocyte damage marker genes, related to Figure 1. PDS and HDS marker gene loci were intersected with kidney- and liver-disease-associated variants from the NHGRI-EBI GWAS Catalog using canonical RefSeq gene bodies and extended regulatory regions. The table reports overlapping disease-associated variants, corresponding traits, affected marker genes, and enrichment statistics relative to expressed kidney or liver background genes.

Abbreviation in figure	Explanation in figure caption	Full description for methods
Nphs2^{mut}	*Nphs2*$^{R231Q/A286V}$ mutant	*Nphs2*$^{R231Q/A286V}$ compound heterozygous mutant mice (Butt et al.[16])
Wt1$^{ko/wt}$	*Wt1* heterozygous	*Wt1* heterozygous whole-body knockout (Kann et al.[28])
Pdss2^{mut}	*Pdss2*$^{V117M/V117M}$ mutant	*Pdss2*$^{V117M/V117M}$ homozygous mutant mice generated by CRISPR strategy.
BTBR$^{ob/ob}$	Diabetic nephropathy	BTBR.Cg-*Lep*^{ob}/WiscJ, Data taken from Chung et al. (Chung et al.[1])
Cd2ap^{pko}	Conditional podocyte *Cd2ap* knock-out	*Nphs2*^{Cre} x *Cd2ap*$^{fl/fl}$, data taken from Chung et al. (Chung et al.[1])
Adria	Adriamycin toxicity model	Data taken from Chung et al. (Chung et al.[1])
NTSN	Nephrotoxic serum nephritis	Data taken from Chung et al. (Chung et al.[1])

Table S4: Description of FSGS damage models and corresponding scRNA-seq datasets used in the manuscript, related to Figure 4.

Supplemental references

[S1] Chung, J.J., Goldstein, L., Chen, Y.J.J., Lee, J., Webster, J.D., Roose-Girma, M., Paudyal, S.C., Modrusan, Z., Dey, A., and Shaw, A.S. (2020). Single-cell transcriptome profiling of the kidney glomerulus identifies key cell types and reactions to injury. Journal of the American Society of Nephrology *31*, 2341–2354. https://doi.org/10.1681/ASN.2020020220/-/DCSUPPLEMENTAL.

[S2] Guilliams, M., Bonnardel, J., Haest, B., Vanderborght, B., Wagner, C., Remmerie, A., Bujko, A., Martens, L., Thoné, T., Browaeys, R., et al. (2022). Spatial proteogenomics reveals distinct and evolutionarily conserved hepatic macrophage niches. Cell *185*, 379-396.e38. https://doi.org/10.1016/J.CELL.2021.12.018.

[S3] Habenicht, L.K.L., Wang, Z., Zhang, X., Li, Y., Mogler, C., Huspenina, J.S., Schmid, R.M., Weber, C., Mohanta, S.K., Ma, Z., et al. (2022). The C1q-ApoE complex: A new hallmark pathology of viral hepatitis and nonalcoholic fatty liver disease. Front. Immunol. *13*. https://doi.org/10.3389/FIMMU.2022.970938.

[S4] Xiao, Y., Batmanov, K., Hu, W., Zhu, K., Tom, A.Y., Guan, D., Jiang, C., Cheng, L., McCright, S.J., Yang, E.C., et al. (2023). Hepatocytes demarcated by EphB2 contribute to the progression of nonalcoholic steatohepatitis. Sci. Transl. Med. *15*. https://doi.org/10.1126/SCITRANSLMED.ADC9653.

[S5] Hatje, F.A., Wedekind, U., Sachs, W., Loreth, D., Reichelt, J., Demir, F., Kosub, C., Heintz, L., Tomas, N.M., Huber, T.B., et al. (2021). Tripartite separation of glomerular cell types and proteomes from reporter-free mice. Journal of the American Society of Nephrology *32*, 2175–2193. https://doi.org/10.1681/ASN.2020091346/-/DCSUPPLEMENTAL.

[S6] Chittka, D., Banas, B., Lennartz, L., Putz, F.J., Eidenschink, K., Beck, S., Stempfl, T., Moehle, C., Reichelt-Wurm, S., and Banas, M.C. (2018). Long-term expression of glomerular genes in diabetic nephropathy. Nephrol. Dial. Transplant *33*, 1533–1544. https://doi.org/10.1093/ndt/gfx359.

[S7] Bird, T.G., M ller, M., Boulter, L., Vincent, D.F., Ridgway, R.A., Lopez-Guadamillas, E., Lu, W.Y., Jamieson, T., Govaere, O., Campbell, A.D., et al. (2018). TGFβ inhibition restores a regenerative response in acute liver injury by suppressing paracrine senescence. Sci. Transl. Med. *10*. https://doi.org/10.1126/SCITRANSLMED.AAN1230.

[S8] Zhong, Y., Lee, K., Deng, Y., Ma, Y., Chen, Y., Li, X., Wei, C., Yang, S., Wang, T., Wong, N.J., et al. (2019). Arctigenin attenuates diabetic kidney disease through the activation of PP2A in podocytes. Nat. Commun. *10*, 4523. https://doi.org/10.1038/s41467-019-12433-w.

[S9] Schaum, N., Karkanias, J., Neff, N.F., May, A.P., Quake, S.R., Wyss-Coray, T., Darmanis, S., Batson, J., Botvinnik, O., Chen, M.B., et al. (2018). Single-cell transcriptomics of 20 mouse organs creates a Tabula Muris. Nature *562*, 367–372. https://doi.org/10.1038/S41586-018-0590-4.

[S10] Cao, A., Li, J., Asadi, M., Basgen, J.M., Zhu, B., Yi, Z., Jiang, S., Doke, T., El Shamy, O., Patel, N., et al. (2021). DACH1 protects podocytes from experimental diabetic injury and modulates PTIP-H3K4Me3 activity. J. Clin. Invest. *131*. https://doi.org/10.1172/JCI141279.

[S11] Schaum, N., Lehallier, B., Hahn, O., Pálovics, R., Hosseinzadeh, S., Lee, S.E., Sit, R., Lee, D.P., Losada, P.M., Zardeneta, M.E., et al. (2020). Ageing hallmarks exhibit organ-specific temporal signatures. Nature *583*, 596–602. https://doi.org/10.1038/S41586-020-2499-Y.

[S12] Okabe, M., Motojima, M., Miyazaki, Y., Pastan, I., Yokoo, T., and Matsusaka, T. (2019). Global polysome analysis of normal and injured podocytes. Am. J. Physiol. Renal Physiol. *316*, F241–F252. https://doi.org/10.1152/ajprenal.00115.2018.

[S13] Marshall, J.L., Noel, T., Wang, Q.S., Chen, H., Murray, E., Subramanian, A., Vernon, K.A., Bazua-Valenti, S., Liguori, K., Keller, K., et al. (2022). High-resolution Slide-seqV2 spatial transcriptomics enables discovery of disease-specific cell neighborhoods and pathways. iScience *25*. https://doi.org/10.1016/J.ISCI.2022.104097.

[S14] Talyan, S., Filipów, S., Ignarski, M., Smieszek, M., Chen, H., Kühne, L., Butt, L., Göbel, H., Hoyer-Allo, K.J.R., Koehler, F.C., et al. (2021). CALINCA-A Novel Pipeline for the Identification of lncRNAs in Podocyte Disease. Cells *10*, 1–17. https://doi.org/10.3390/CELLS10030692.

[S15] Ettou, S., Jung, Y.L., Miyoshi, T., Jain, D., Hiratsuka, K., Schumacher, V., Taglienti, M.E., Morizane, R., Park, P.J., and Kreidberg, J.A. (2020). Epigenetic transcriptional reprogramming by WT1 mediates a repair response during podocyte injury. Sci. Adv. *6*, eabb5460. https://doi.org/10.1126/sciadv.abb5460.

[S16] Butt, L., Unnersjö-Jess, D., Höhne, M., Edwards, A., Binz-Lotter, J., Reilly, D., Hahnfeldt, R., Ziegler, V., Fremter, K., Rinschen, M.M., et al. (2020). A molecular mechanism explaining albuminuria in kidney disease. Nat. Metab. *2*, 461–474. https://doi.org/10.1038/S42255-020-0204-Y.

[S17] Hengel, F.E., Dehde, S., Lassé, M., Zahner, G., Seifert, L., Schnarre, A., Kretz, O., Demir, F., Pinnschmidt, H.O., Grahammer, F., et al. (2024). Autoantibodies Targeting Nephrin in Podocytopathies. N. Engl. J. Med. *391*, 422–433. https://doi.org/10.1056/NEJMoa2314471.

[S18] Koehler, S., Kuczkowski, A., Kuehne, L., Jüngst, C., Hoehne, M., Grahammer, F., Eddy, S., Kretzler, M., Beck, B.B., Höhfeld, J., et al. (2020). Proteome Analysis of Isolated Podocytes Reveals Stress Responses in Glomerular Sclerosis. J. Am. Soc. Nephrol. *31*, 544–559. https://doi.org/10.1681/ASN.2019030312.

[S19] Williams, E.G., Pfister, N., Roy, S., Statzer, C., Haverty, J., Ingels, J., Bohl, C., Hasan, M., Čuklina, J., Bühlmann, P., et al. (2022). Multiomic profiling of the liver across diets and age in a diverse mouse population. Cell Syst. *13*, 43-57.e6. https://doi.org/10.1016/j.cels.2021.09.005.

[S20] Lake, B.B., Menon, R., Winfree, S., Hu, Q., Ferreira, R.M., Kalhor, K., Barwinska, D., Otto, E.A., Ferkowicz, M., Diep, D., et al. (2023). An atlas of healthy and injured cell states and niches in the human kidney. Nature *619*, 585–594. https://doi.org/10.1038/S41586-023-05769-3.

[S21] Latt, K.Z., Heymann, J., Jessee, J.H., Rosenberg, A.Z., Berthier, C.C., Arazi, A., Eddy, S., Yoshida, T., Zhao, Y., Chen, V., et al. (2021). Urine Single-Cell RNA Sequencing in Focal Segmental Glomerulosclerosis Reveals Inflammatory Signatures. Kidney Int. Rep. *7*, 289–304. https://doi.org/10.1016/J.EKIR.2021.11.005.

[S22] Carlessi, R., Denisenko, E., Boslem, E., Köhn-Gaone, J., Main, N., Abu Bakar, N.D.B., Shirolkar, G.D., Jones, M., Beasley, A.B., Poppe, D., et al. (2023). Single-nucleus RNA sequencing of pre-malignant liver reveals disease-associated hepatocyte state with HCC prognostic potential. Cell genomics *3*, 100301. https://doi.org/10.1016/j.xgen.2023.100301.

[S23] Preston, R., Chrisp, R., Dudek, M., Morais, M.R.P.T., Tian, P., Williams, E., Naylor, R.W., Davenport, B., Pathiranage, D.R.J., Benson, E., et al. (2024). The glomerular circadian clock temporally regulates basement membrane dynamics and the podocyte glucocorticoid response. Kidney Int. https://doi.org/10.1016/J.KINT.2024.10.016.

[S24] Auerbach, B.J., FitzGerald, G.A., and Li, M. (2022). Tempo: an unsupervised Bayesian algorithm for circadian phase inference in single-cell transcriptomics. Nat. Commun. *13*. https://doi.org/10.1038/S41467-022-34185-W.

[S25] Du, K., Umbaugh, D.S., Wang, L., Jun, J.H., Dutta, R.K., Oh, S.H., Ren, N., Zhang, Q., Ko, D.C., Ferreira, A., et al. (2025). Targeting senescent hepatocytes for treatment of metabolic dysfunction-associated steatotic liver disease and multi-organ dysfunction. Nat. Commun. *16*, 3038. https://doi.org/10.1038/s41467-025-57616-w.

[S26] Yu, G., Wang, L.G., Han, Y., and He, Q.Y. (2012). clusterProfiler: an R package for comparing biological themes among gene clusters. OMICS *16*, 284–287. https://doi.org/10.1089/OMI.2011.0118.

[S27] Vesting, A.J., Jais, A., Klemm, P., Steuernagel, L., Wienand, P., Fog-Tonnesen, M., Hvid, H., Schumacher, A.-L., Kukat, C., Nolte, H., et al. (2022). NIK/MAP3K14 in hepatocytes orchestrates NASH to hepatocellular carcinoma progression via JAK2/STAT5 inhibition. Mol. Metab. *66*, 101626. https://doi.org/10.1016/j.molmet.2022.101626.

[S28] Kann, M., Ettou, S., Jung, Y.L., Lenz, M.O., Taglienti, M.E., Park, P.J., Schermer, B., Benzing, T., and Kreidberg, J.A. (2015). Genome-wide analysis of Wilms' tumor 1-controlled gene expression in podocytes reveals key regulatory mechanisms. Journal of the American Society of Nephrology *26*, 2097–2104. https://doi.org/10.1681/ASN.2014090940/-/DCSUPPLEMENTAL.
